# Iron catalyzed methylation and ethylation of vinyl arenes†
†Electronic supplementary information (ESI) available. See DOI: 10.1039/c6sc04274k



**DOI:** 10.1039/c6sc04274k

**Published:** 2016-12-02

**Authors:** Nengbo Zhu, Jianguo Zhao, Hongli Bao

**Affiliations:** a State Key Laboratory of Structural Chemistry , Key Laboratory of Coal to Ethylene Glycol and Its Related Technology , Fujian Institute of Research on the Structure of Matter , University of Chinese Academy of Sciences , 155 Yangqiao Road West , Fuzhou , 350002 , P. R. China . Email: hlbao@fjirsm.ac.cn

## Abstract

Short alkyl chain Heck (type) reactions, especially methyl Heck reactions, are a difficult aspect of the alkyl Heck reaction. To provide a solution to this problem, iron-catalyzed methyl, ethyl and propyl Heck reactions were developed using readily available alkyl peroxides as alkyl sources. The reaction conditions were mild, clean, and easy to handle. No additive was needed, and no hazardous waste was generated. The products were obtained in up to 99% yield of one isomer for most situations. This reaction works for many types of olefin and tolerates a variety of functional groups. Several late-stage functionalizations of natural products and drug molecules were conducted to demonstrate the synthetic applications of this reaction.

## Abstract 2

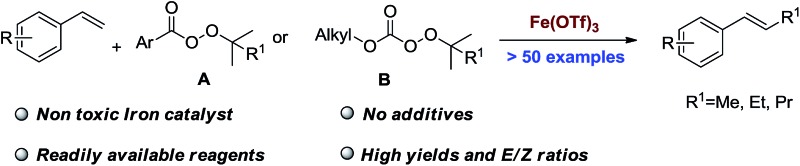
Iron-catalyzed methyl, ethyl and propyl Heck reactions were developed using readily available alkyl peroxides as alkyl sources.

The methyl group, which is so often considered to be chemically inert, is able to significantly alter the pharmacological properties of a molecule.^1^ The methyl group affects micromolecules and biomacromolecules through stereoelectronic effects to increase their potency against enzyme metabolism or changes in the physiochemical properties of bioactive compounds.^2^ The importance of the methyl group on arenes or heteroarenes is widely recognized, whereas the importance of the methyl group in alkyl or vinyl chains is less well understood. In fact, methyl groups in the alkyl chain or vinyl chain can profoundly affect a bioactive compound's properties.^2,3^

The Heck reaction is a powerful tool in chemical synthesis.^4–12^ Aryl Heck reactions are well established whereas alkyl Heck and alkyl Heck type reactions (for brevity, all Heck reactions including Heck-type reactions are referred to as Heck reactions), especially methyl Heck reactions are still challenging.^13–23^ Methyl hydrazine,^24^ methyl halides,^13,14,25^ and silanes^26^ have been applied to solve this problem (Scheme 1). Unfortunately, the methyl Heck reaction remains challenging and there is no practical solution to this problem using readily available methylation reagents.

**Scheme 1:**
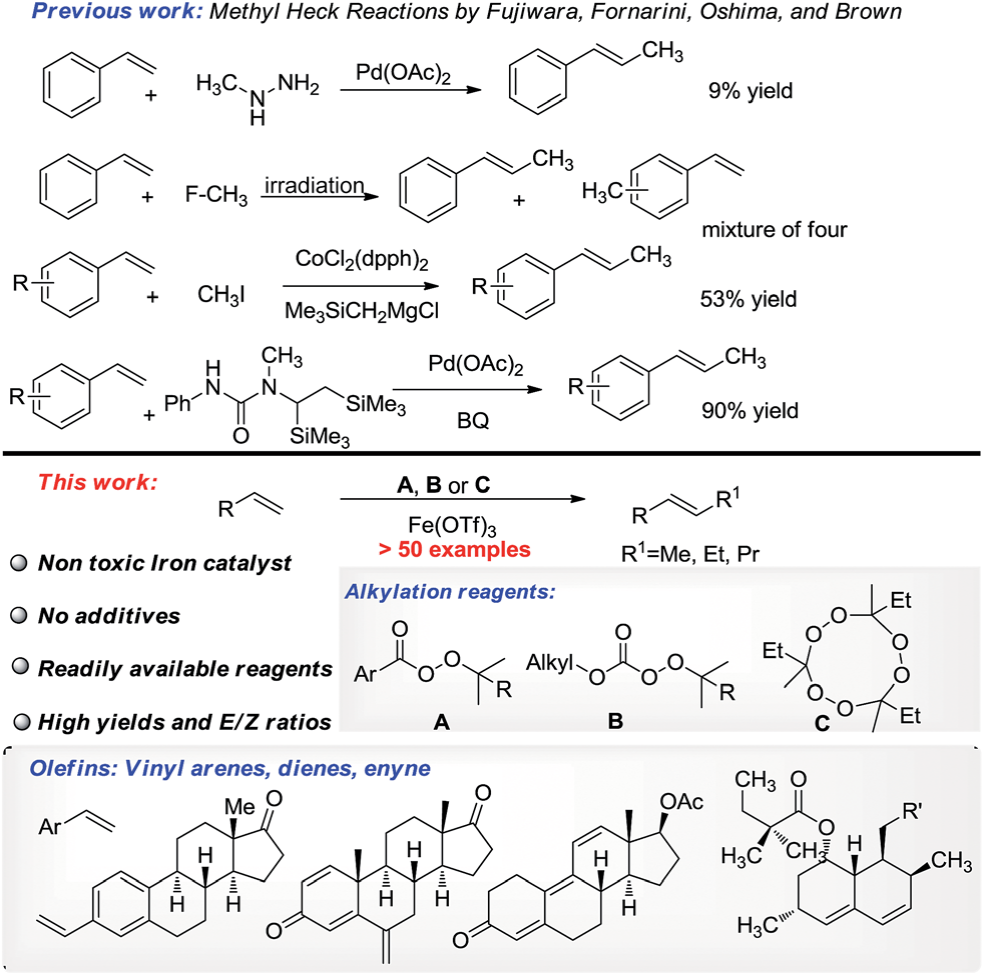
Short chain alkylation of olefins.

Perester (**A**) and *OO-tert*-butyl (or pentyl) *O*-alkyl monopercarbonate (**B**) are inexpensive chemical feedstocks commonly used in industry as oxidants or radical initiators. These peroxides are rarely seen as alkylation reagents.^27–33^ Li and co-workers^29^ developed a procedure for the methylation of aryl C–H bonds using dicumyl peroxide as the methylation reagent, and DiRocco and co-workers developed a procedure for the late-stage methylation of heterocycles with peresters as methyl sources.^31^ Both examples mentioned above worked on the methylation of arenes or heterocycles,^34,35^ rather than double bonds.

Many peresters and *OO-tert*-butyl (or pentyl) *O*-alkyl monopercarbonates (**B**) are commercially available. It would be synthetically useful if a methyl Heck reaction based on these non-ordinary methylation reagents was developed. Iron is one of the most important metals in nature.^36–41^ Many iron compounds are observed to be essential components of biological systems and have low toxicity in living systems. The application of nontoxic iron catalysts in pharmaceutical related chemistry is highly desired as it could reduce the risk of contamination by metal catalysts. Here, we report our work on the methylation of double bonds with an iron catalyst. This iron catalyzed unprecedented short alkyl chain Heck reaction of olefins applies readily available peroxides (**A**, **B**, **C**) as the alkylation reagents (Scheme 1). Type **B** peroxides are shown to be valuable alkyl electrophiles for the first time.

We commenced our study by screening metal catalysts for the reaction of *p-tert*-butyl-styrene **1** with commercially available *tert*-butyl peroxybenzoate (**2**) as the methylation reagent. Copper, nickel and palladium catalysts did not deliver the methylation product in an effective way. Styrene **1** and benzoic acid were the two major peaks observed using GC-MS (Table 1, entries 1–4). To our delight, Fe(OTf)_3_ (10 mol%) promoted the reaction and afforded the product in 85% yield (entry 8). Study of the reaction temperature revealed that 80 °C was optimal (entries 8–12). Solvent screening showed that THF and dioxane were both suitable for this reaction (entry 16). When reducing the usage of the catalyst from 10 mol% to 5 mol% in dioxane, the yield increased slightly (entry 17, 90% yield). The yield dropped to 36% when one equivalent of the catalyst was employed (entry 18). Fe(OTf)_2_ was examined under the optimal reaction conditions and 65% of the isolated product was obtained (entry 19). The product was obtained in 34% yield in the presence of HOTf (entry 20). There was no product generated without Fe(OTf)_3_ or under photoredox catalysis conditions (entries 21–22). The reaction conditions were mild, clean and easy to handle, used a nontoxic catalyst and required no additives. This method is potentially useful due to its low cost and environmental advantages.

**Table 1:** Reaction condition screening^*a*^

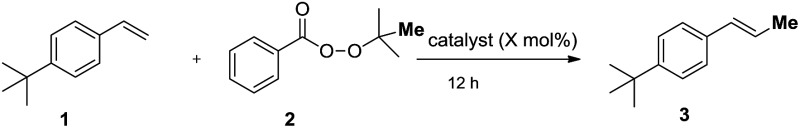				
Entry	Metal (mol%)	Solvent	Temp. (°C)	Yield^*b*^ (%)
1	Cu(OTf)_2_/10	THF	80	Trace
2	CuBr/10	THF	80	Trace
3	Pd(TFA)_2_/10	THF	80	Trace
4	NiCl_2_/10	THF	80	Trace
5	FeSO_4_·7H_2_O (10)	THF	80	Trace
6	Fe(OAc)_2_ (10)	THF	80	Trace
7	Fe(acac)_3_ (10)	THF	80	Trace
8	Fe(OTf)_3_ (10)	THF	80	85
9	Fe(OTf)_3_ (10)	THF	rt	46
10	Fe(OTf)_3_ (10)	THF	40	56
11	Fe(OTf)_3_ (10)	THF	60	69
12	Fe(OTf)_3_ (10)	PhCH_3_	80	6
13	Fe(OTf)_3_ (10)	CH_3_CN	80	—
14	Fe(OTf)_3_ (10)	DMF	80	21
15	Fe(OTf)_3_ (10)	DME	80	70
16	Fe(OTf)_3_ (10)	Dioxane	80	80
17	Fe(OTf)_3_ (5)	Dioxane	80	90 (85)^*c*^
18	Fe(OTf)_3_ (100)	Dioxane	80	36
19	Fe(OTf)_2_(5)	Dioxane	80	65^*c*^
20	HOTf (15)	Dioxane	80	34
21	—	Dioxane	80	Trace
22^*d*^	White LED light	Dioxane	rt	Trace

^*a*^Reactions were conducted with styrene (0.5 mmol), **2** (1.0 mmol), catalyst and solvent (2 mL) at 80 °C for 12 h.

^*b*^Yield of product detected using GC.

^*c*^Yield of isolated product.

^*d*^5 mol% of RuCl_3_(bpy)_3_ 6H_2_O as photocatalyst.

Having identified the optimal conditions, we investigated the reactivity of thirteen methylation reagents (Table 2, **6–18**). The methylation product was obtained in up to an 87% yield. Effective methylation reagent **13** is commercially available, and reagents **10–12** and **14–18** were easily made from *t*-butyl hydroperoxide. Encouraged by these results, six ethylation reagents (**19–24**) and one propylation reagent (**26**) were synthesized and examined. Up to a 99% yield for ethylation and a 90% yield for propylation were obtained. Interestingly, an 81% yield of an ethylation product was achieved with commercially available reagent **26** (TMTETPN). It is worth noting that *trans* olefin was the only isomer observed. We found that peresters (**A**) are more reactive, while monopercarbonates (**B**) are milder and thus important for sensitive substrates (Table 3).

**Table 2:** Scope of methylation, ethylation and propylation reagents^*a*^

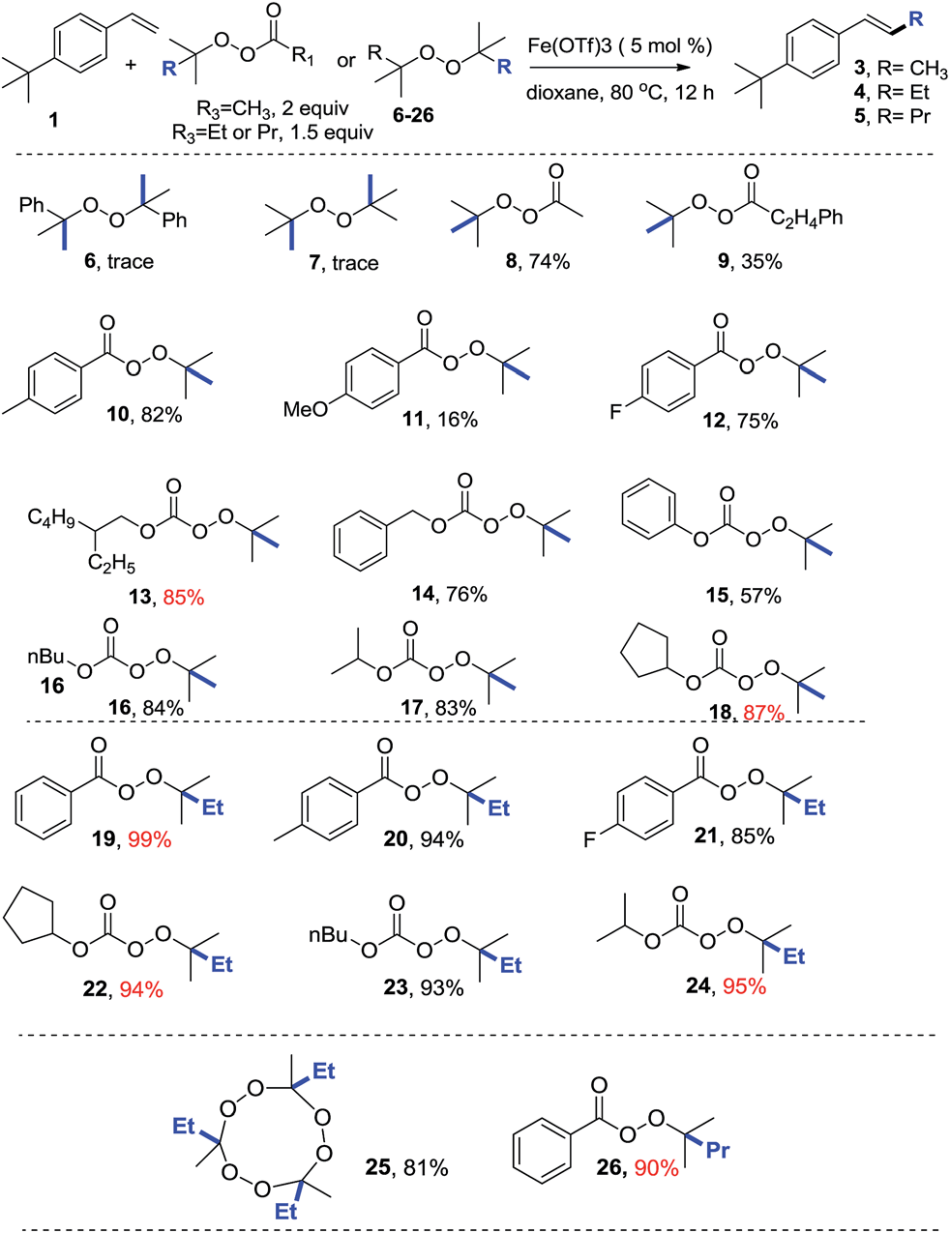

^*a*^Yield of isolated product.

**Table 3:** Scope of olefins^*a*^

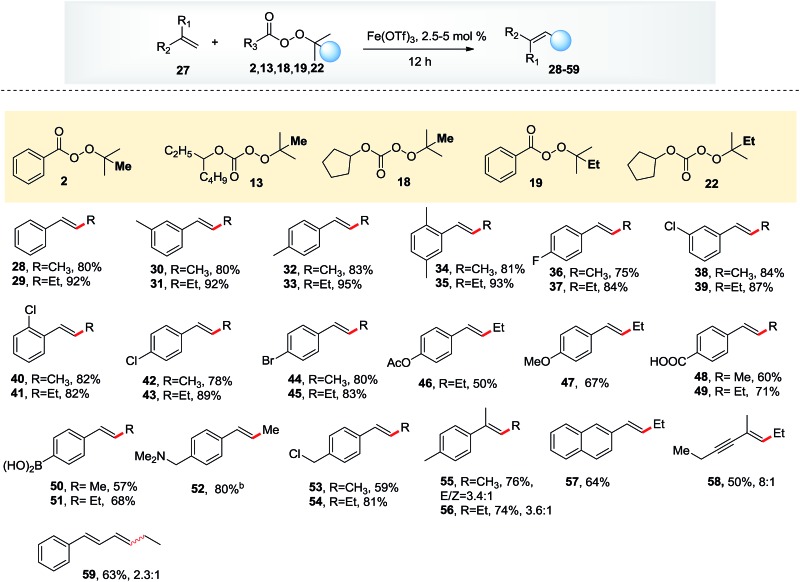

^*a*^Reactions were conducted on a 0.5 mmol scale with Fe(OTf)_3_ (2.5–5 mol%) and THF or dioxane (2 mL) at 80 °C for 12 h. Please see ESI for more details.

^*b*^3 equiv. of HOTf was added as an additive.

Having established the reactivity of these three types of peroxides in the presence of iron, we investigated the scope of the olefins, which proved to be broad. Alkyl substituents on the phenyl ring increased the yield to 80–95%, whereas halogen substituents decreased the yield to 70–88%. Functional groups such as acetate, methoxyl, free carboxylic acid, boronic acid and amine were all tolerated. 2-Vinylnaphthalene afforded products in reasonable yields. Importantly, dienes and enynes were suitable substrates for this reaction, delivering the corresponding products in moderate yields.

To exemplify the synthetic application of this method we examined this reaction with regard to natural products and drug molecules. The late-stage modification of exemestane **60**, an aromatase inhibitor used in the treatment of breast cancer, delivered the double bond shift product **61a** and the normal product **61b** in 61% yield (Scheme 2). The functionalization of trenbolone acetate **62** afforded derivative **63** in a 41% yield. Remarkably, vinylestrone **64** delivered the ethyl Heck product **65** in a 91% yield. Simvastatin derivative **67** was examined in this reaction. The site selectivity was good and the ethylation product **68** was obtained in 48% yield. These examples demonstrate that this reaction is a useful method to functionalize complex molecules in the late stages.

**Scheme 2:**
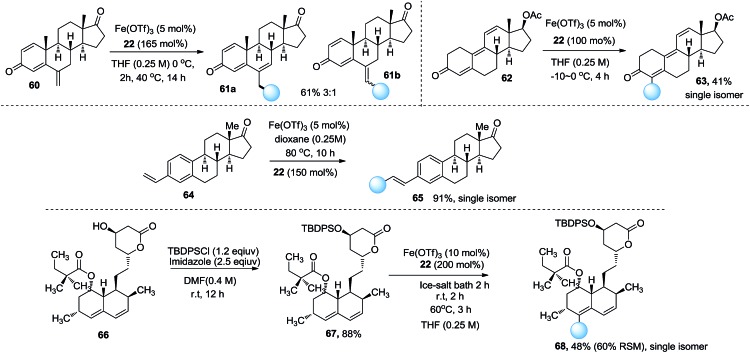
Synthetic applications of natural products and drug molecules.

To learn more about the reactivity information of this reaction with non-conjugated olefins, natural product betulin **69** was examined (Scheme 3). The reaction did not reach full conversion and it delivered ethyl Heck product **70** as a mixture of *E*/*Z* isomers (*E*/*Z* = 1.9 : 1) in 30% yield (25% of the starting material was recovered). Other simple olefins suffer with the same conversion and *E*/*Z* selectivity problems. This reaction is less ideal for non-conjugated olefins.

**Scheme 3:**
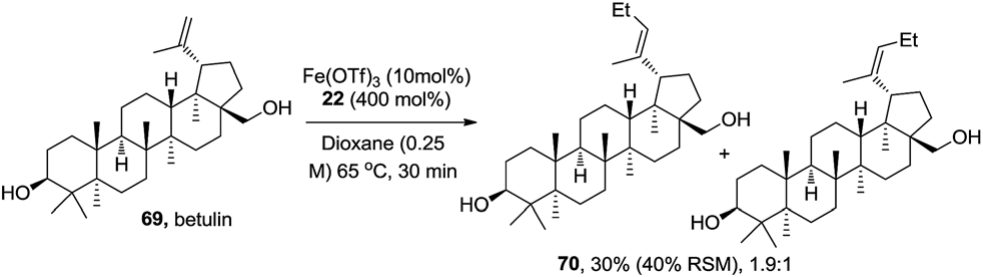
Example of a non-conjugated olefin.

Inspired by Fenton's reaction^42^ and the work of Bedford, Nagashima, Nakamura, and Neidig,^43–46^ a single electron transfer catalytic cycle involving iron complexes, that differs only in one oxidation state, is proposed. Fe(OTf)_3_ is reduced to iron(ii). Iron(ii) complex **T-A** transfers an electron to the perester to form an iron(iii) compound (**T-B**) and a methyl radical. The methyl radical reacts with styrenes to form a benzylic radical (**T-C**). The benzylic radical (**T-C**) is oxidized by the iron(iii) species (**T-B**) to a carbocation (**T-D**) and reforms the iron(ii) species (**T-A**). The carbocation (**T-D**) then undergoes deprotonation to deliver the desired product. To support this proposed mechanism a radical trapping experiment was conducted using NHPI (*N*-hydroxyphthalimide). The reaction was stopped and no desired product was observed, while radical adduct **71** was observed using GC-MS. Thereafter, MeOH was added to the reaction to see whether it would capture the carbon cation. Fortunately, methyl ether **72** was isolated from this reaction in a 30% yield (Scheme 4).

**Scheme 4:**
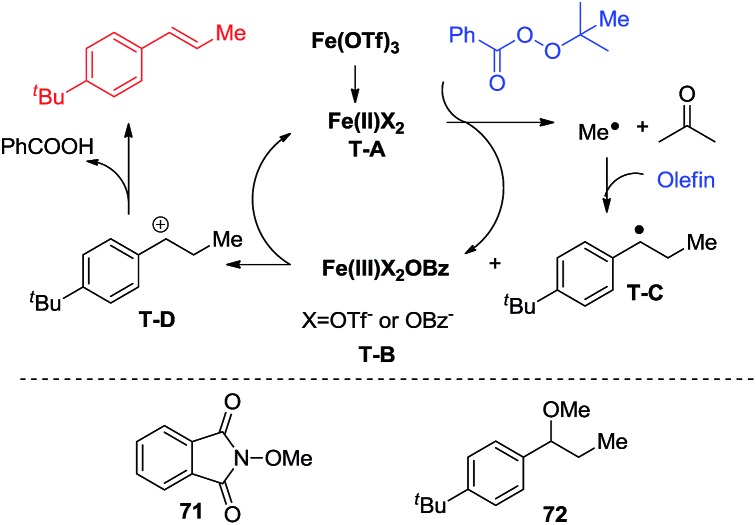
Proposed catalytic cycle.

## Conclusions

In summary, we have developed a practical method for the short chain alkylation of olefins using readily available peresters and monopercarbonates as alkylation reagents. Many of these reagents are commercially available and the others are easy to synthesize. The simple reaction conditions and environmental benefits of this catalytic system open up the possibility of further synthetic applications. This reaction is efficient and selective, and works for vinyl arenes, dienes, enynes, and complicated natural products or drug molecules.
