# Supplementary material for: Iron catalyzed methylation and ethylation of vinyl arenes
Source: Chem Sci. 2016 Dec 2;8(3):2081–5. doi: 10.1039/c6sc04274k (PMC5399631; doi:10.1039/c6sc04274k)

Electronic Supplementary Material (ESI) for Chemical Science.
This journal is © The Royal Society of Chemistry 2016

Iron Catalyzed Methylation and Ethylation of Vinyl Arenes

Nengbo Zhu, Jianguo Zhao and Hongli Bao *

State Key Laboratory of Structural Chemistry, Key Laboratory of Coal to Ethylene Glycol and Its Related Technology, Fujian Institute of Research on the Structure of Matter, University of Chinese Academy of Sciences, 155 Yangqiao Road West, Fuzhou, 350002, P.R. China.

Table of contents:

Materials and methods..S-2

Synthesis of peresters and peroxycarbonates..S-3

Synthesis of alkenes………………………...……S-9

General procedure for the Methylation and Ethylation of Vinyl Arenes…....S-11

Characterization data for products……………………………………………..S-15

Mechanistic study…………………………………………………………….....S-27

Reference………………………………………………………………………….S-29

NMR spectra……………………………………….....................................S-31

Materials and methods

All reactions were carried out under an atmosphere of nitrogen in flame-dried glassware with magnetic stirring unless otherwise indicated. Commercially obtained reagents were used as received. Solvents were dried by Innovative Technology Solvent Purification System. Liquids and solutions were transferred via syringe. All reactions were monitored by thin-layer chromatography. GC and GC-MS data were recorded on Thermo Trace 1300 and Thermo ISQ QD, respectively. ^{1}H and ^{13}C NMR spectra were recorded on Bruker-BioSpin AVANCE III HD. Data for ^{1}H NMR spectra are reported relative to chloroform (7.26 ppm) or dimethyl sulfoxide (3.36 ppm) as an internal standard and are reported as follows: chemical shift (ppm), multiplicity, coupling constant (Hz), and integration. Data for ^{13}C NMR spectra are reported relative to chloroform (77.23 ppm) or dimethyl sulfoxide (39.98 ppm) as an internal standard and are reported in terms of chemical shift (ppm). HRMS data were recorded on Waters Micromass GCT.

Synthesis of peresters and peroxycarbonates

Peresters and peroxycarbonates have potentials to explode. Any peresters and peroxycarbonates involved reaction (as product or substrate) should be carried out with precautions!

Perester **2** was purchased from Admas; **6** was purchased from Amquar; **7** was purchased from Energy; **8** and **13** from *J&K*; **25** from Acros. Other peresters and peroxycarbonates were prepared according to the corresponding method.

Method A:

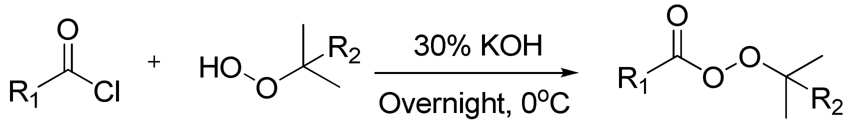

A solution of corresponding hydroperoxide (5.5 mmol) was cooled to 0 °C and corresponding acyl chloride (5 mmol) was added dropwise. The mixture was rapidly stirred in 30 minutes. The KOH solution (30% in water) (6.5 mmol) was then added dropwise. The mixture was then stirred for overnight at 0 °C. The reaction mixture was washed by water and CH_2Cl_2. the organic layer was dried over anhydrous magnesium sulfate and concentrated by rotary evaporator under reduced pressure. The residue was purified by flash chromatography (hexanes/ethyl acetate = 20/1~8/1) to afford the desired product.

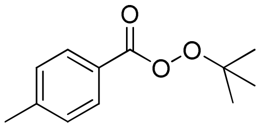

10

Following the method A, 925.6 mg, 89% yield, clear oil. ^{1}H NMR (400 MHz, $CDCl_3$) δ 7.84 (d, *J* = 8.0 Hz, 2H), 7.25 (d, *J* = 8.0 Hz, 2H), 2.42 (s, 3H), 1.42 (s, 9H). ^{13}C NMR (400 MHz, $CDCl_3$) δ 164.57, 144.20, 129.35, 129.17, 124.84, 83.91, 26.28, 21.75.

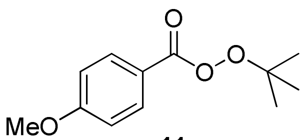

11

Following the method A, 930.9 mg, 83% yield, clear oil. ^{1}H NMR (400 MHz, $CDCl_3$) δ 7.91 (d, *J* = 16.0 Hz, 2H), 6.93 (d, *J* = 8.0 Hz, 2H), 3.87 (s, 3H), 1.41 (s, 9H). ^{13}C NMR (400 MHz, $CDCl_3$) δ 164.28, 163.63, 131.21, 119.79, 113.92, 83.82, 55.50, 26.28.

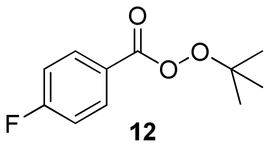

12

Following the method B, 922.2 mg, 87% yield, clear oil. ^{1}H NMR (400 MHz, $CDCl_3$) δ 8.04−7.94 (m, 2H), 7.15 (t, *J* = 8.0 Hz, 2H), 1.42 (s, 9H). ^{13}C NMR (400 MHz, $CDCl_3$) δ 167.17, 164.64, 163.50, 131.79, 131.70, 123.89, 123.86, 116.04, 115.82, 84.14, 26.25. ^{19}F (376 MHz, $CDCl_3$) δ -104.45.

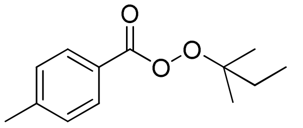

20

Following the method A, 954.8 mg, 86% yield, clear oil.^{1}H NMR (400 MHz, $CDCl_3$) δ 7.83 (d, *J* = 8.0 Hz, 2H), 7.24 (d, *J* = 8.0 Hz, 2H), 2.41 (s, 3H), 1.75 (q, *J* = 8.0 Hz, 2H), 1.36 (s, 6H), 0.99 (t, *J* = 8.0 Hz, 3H). ^{13}C NMR (400 MHz, $CDCl_3$) δ 164.44, 144.11, 129.31, 129.11, 124.91, 86.19, 31.63, 23.87, 21.71, 8.35.

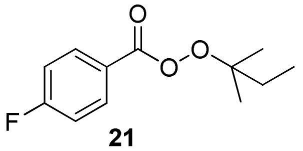

21

Following the method A, 892 mg, 79% yield, clear oil. ^{1}H NMR (400 MHz, $CDCl_3$) δ 8.03−7.93 (m, 2H), 7.18−7.11 (m, 2H), 1.75 (q, *J* = 8.0 Hz, 2H), 1.36 (s, 6H), 0.99 (t, *J* = 8.0 Hz, 3H). ^{13}C NMR (400 MHz, $CDCl_3$) δ 167.13, 164.60, 163.41, 131.75, 131.65, 123.96, 116.01, 115.79, 86.46, 31.62, 23.86, 8.35. ^{19}F (376 MHz, $CDCl_3$) δ -104.52.

Method B:

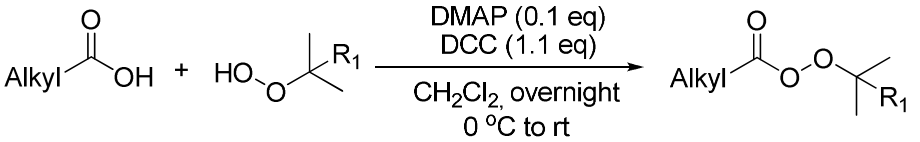

A solution of the corresponding carboxylic acid (5 mmol) and DMAP (0.5 mmol) in CH_2Cl_2 (4 mL) was cooled to 0 °C and hydroperoxide (5.5 mmol) was added dropwise. The reaction mixture was stirred for 10 min. DCC (5.5 mmol) was added to the reaction mixture and the resulting mixture was stirred at 0 °C for 30 min, then at room temperature overnight. The reaction solution was filtered. The filtrate was concentrated by rotary evaporator under reduced pressure and purified by flash

column chromatography (petroleum ether/ethyl acetate = 20/1~8/1) to afford the desired product.

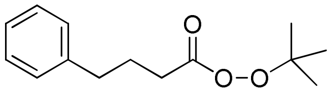

9

Following the method B, 944 mg, 80% yield, clear oil. ^{1}H NMR (400 MHz, $CDCl_3$) δ ^{1}H NMR (400 MHz, $CDCl_3$) δ 7.31 (t, *J* = 8.0 Hz, 2H), 7.21-7.17 (m, 3H), 2.68 (t, *J* = 8.0 Hz, 2H), 2.32 (t, *J* = 8.0 Hz, 2H), 2.06-1.96 (m, 2H), 1.32 (s, 9H). ^{13}C NMR (100 MHz, $CDCl_3$) δ 170.83, 140.98, 128.48, 126.14, 83.32, 35.02, 30.56, 26.52, 26.17.

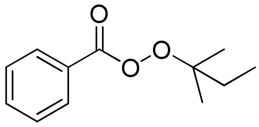

19

Following the method B, 821.9 mg, 79% yield, clear oil. ^{1}H NMR (400 MHz, $CDCl_3$) δ 7.94 (d, *J* = 8.0 Hz, 2H), 7.59 (t, *J* = 8.0 Hz, 1H), 7.46 (t, *J* = 8.0 Hz, 2H), 1.75 (q, *J* = 8.0 Hz, 2H), 1.37 (s, 6H), 0.99 (t, *J* = 8.0 Hz, 3H). ^{13}C NMR (400 MHz, $CDCl_3$) δ 164.35, 133.33, 129.10, 128.62, 127.76, 86.33, 31.62, 23.88, 8.36.

Method C:

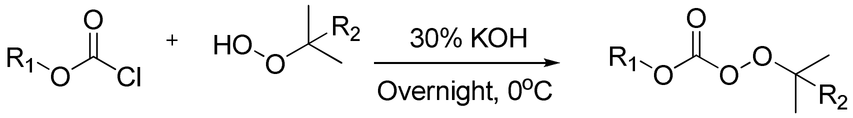

A solution of the corresponding hydroperoxide (5.5 mmol) was cooled to 0 ^{o}C and chloroformate (5 mmol) was added dropwise. The mixture was rapidly stirred in 30 minutes. The KOH solution (30% in water) (6.5 mmol) was then added dropwise. The mixture was then stirred for overnight at 0 ^{o}C. The reaction mixture was washed by water and CH_2Cl_2. the organic layer was dried over anhydrous magnesium sulfate and concentrated by rotary evaporator under reduced pressure. The residue was purified by flash chromatography (hexanes/ethyl acetate = 20/1~8/1) to afford the desired product.

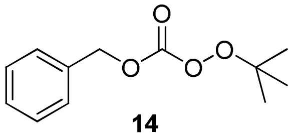

14

Following the method C, 1008.2 mg, 90% yield, clear oil. ^{1}H NMR (400 MHz, $CDCl_3$) δ 7.45−7.32 (m, 5H), 5.24 (s, 2H), 1.32 (s, 9H). ^{13}C NMR (400 MHz, $CDCl_3$) δ 154.77, 134.57, 128.87, 128.69, 128.60, 84.29, 70.61, 25.93.

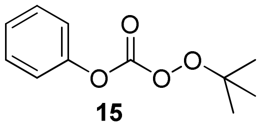

15

Following the method C, 945.3 mg, 90% yield, clear oil. ^{1}H NMR (400 MHz, $CDCl_3$) δ 7.39 (t, J = 8.0 Hz, 2H), 7.26 (t, J = 4.0 Hz, 1H), 7.19 (d, J = 12.0 Hz, 2H), 1.39 (s, 9H). ^{13}C NMR (400 MHz, $CDCl_3$) δ 153.09, 150.80, 129.66, 126.40, 120.79, 84.79, 25.97.

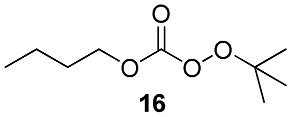

16

Following the method C, 845.5 mg, 89% yield, clear oil. ^{1}H NMR (400 MHz, $CDCl_3$) δ 4.24 (t, J = 8.0 Hz, 2H), 1.73−1.65 (m, 2H), 1.45−1.38 (m, 2H), 1.34 (s, 9H), 0.95 (t, J = 4.0 Hz, 3H). ^{13}C NMR (400 MHz, $CDCl_3$) δ 154.93, 84.10, 69.07, 30.63, 25.94, 18.83, 13.65.

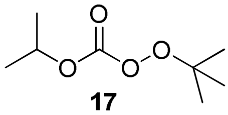

17

Following the method C, 589.5 mg, 67% yield, clear oil. ^{1}H NMR (400 MHz, $CDCl_3$) δ 5.03−4.91 (m, 1H), 1.34 (s, 6H), 1.33 (s, 9H). ^{13}C NMR (400 MHz, $CDCl_3$) δ 154.31, 83.96, 73.68, 25.92, 21.72.

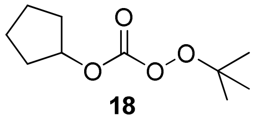

18

Following the method C, 898.9 mg, 89% yield, clear oil. ^{1}H NMR (400 MHz, $CDCl_3$) δ 5.23−5.12 (m, 1H), 1.90−1.59 (m, 8H), 1.33 (s, 9H). ^{13}C NMR (400 MHz, $CDCl_3$) δ 154.54, , 83.92, 82.65, 32.60, 25.92, 23.50.

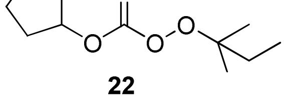

22

Following the method C, 864.5 mg, 80% yield, clear oil. ^{1}H NMR (400 MHz, $CDCl_3$) δ 5.20−5.13 (m, 1H), 1.95−1.70 (m, 6H), 1.69−1.58 (m, 4H), 1.27 (s, 6H), 0.93 (t, J =

8.0 Hz, 3H). ^{13}C NMR (400 MHz, $CDCl_3$) δ 154.51, 86.27, 82.62, 32.62, 31.29, 23.52, 8.22.

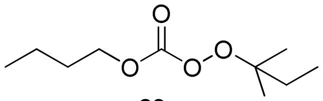

23

Following the method C, 816.5 mg, 80% yield, clear oil. ^{1}H NMR (400 MHz, $CDCl_3$) δ 4.23 (t, J = 8.0 Hz, 2H), 1.72−1.62 (m, 4H), 1.44−1.37 (m, 2H), 1.28 (s, 6H), 0.99−0.90 (m, 6H). ^{13}C NMR (400 MHz, $CDCl_3$) δ 154.85, 86.39, 68.99, 31.27, 30.62, 23.52, 18.81, 13.61, 8.20.

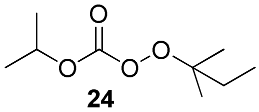

24

Following the method C, 636.8 mg, 67% yield, clear oil. ^{1}H NMR (400 MHz, $CDCl_3$) δ 5.01−4.91 (m, 1H), 1.66 (q, J = 8.0 Hz, 2H), 1.32 (d, J = 8.0 Hz, 6H), 1.27 (s, 6H), 0.93 (t, J = 8.0 Hz, 3H). ^{13}C NMR (400 MHz, $CDCl_3$) δ 154.22, 86.24, 73.57, 31.25, 23.49, 21.68, 8.16.

Method D:

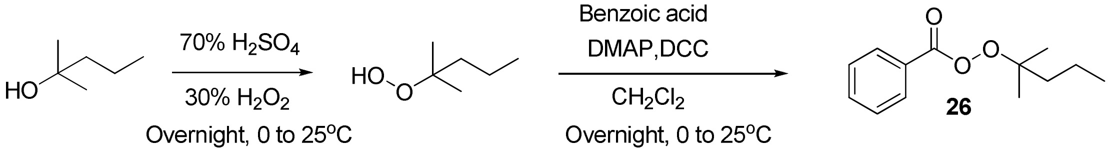

A solution of 70% H_2SO_4 (5 mmol) was cooled to 0 °C and tert-butanol (5 mmol) was added. The H_2O_2 solution (30% in water) (5 mmol) was then added dropwise in 30 minutes. The mixture was then stirred for overnight at 25°C. The reaction mixture was washed by water and CH_2Cl_2. the organic layer was dried over anhydrous magnesium sulfate and concentrated by rotary evaporator under reduced pressure. 2-hydroperoxy-2-methylpentane was prepared.

A solution of Benzoic acid (5 mmol) in CH_2Cl_2 (10 mL) was cooled to 0°C and DMAP (0.5 mmol) was added. 2-hydroperoxy-2-methylpentane (5.5 mmol) was added dropwise and the DCC (5.5 mmol) was added. The mixture was rapidly stirred at 0°C in 30 minutes and overnight at 25°C. After the reaction finished, the reaction solution was filtered. The solution was dried over anhydrous magnesium sulfate and concentrated by rotary evaporator under reduced pressure. The residue was purified

by flash chromatography (hexanes/ethyl acetate = 20/1~8/1) to afford the desired product .

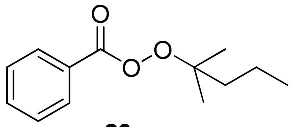

26

Following the method D, 577 mg, 52% total yield, clear oil. ^{1}H NMR (400 MHz, $CDCl_3$) δ 7.94 (d, *J* = 8.0 Hz, 2H), 7.59 (t, *J* = 8.0 Hz, 1H), 7.46 (t, *J* = 8.0 Hz, 2H), 1.68 (t, *J* = 8.0 Hz, 2H), 1.51−1.41 (m, 2H), 1.37 (s, 6H), 0.95 (t, *J* = 8.0 Hz, 3H). ^{13}C NMR (400 MHz, $CDCl_3$) δ 164.35, 133.32, 129.10, 128.62, 127.78, 86.12, 41.36, 24.35, 17.29, 14.60.

Synthesis of alkenes

(*E*)-buta-1,3-dien-1-ylbenzene (**SI-19**) were synthesized by the reference.[1] Other alkenes were purchased from following companies:

Alkenes	Company	Alkenes	Company	Alkenes	Company
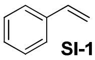 SI-1	Energy	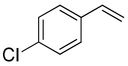 SI-8	Energy	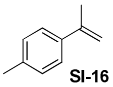 SI-16	Energy
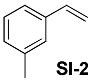 SI-2	Adamas	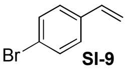 SI-9	Energy	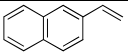 SI-17	Meryer
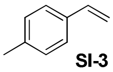 SI-3	Energy	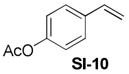 SI-10	Energy	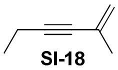 SI-18	Sigma
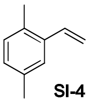 SI-4	Alfa	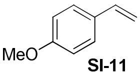 SI-11	Energy	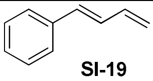 SI-19	Reference[1]
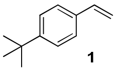 1	Energy	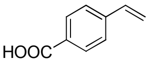 SI-12	Energy	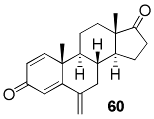 60	Energy
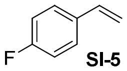 SI-5	Adamas	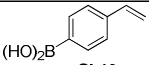 SI-13	Bide	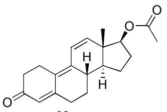 62	Heowns
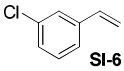 SI-6	Energy	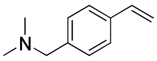 SI-14	*J&K*	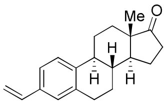 64	Reference[2]
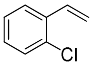 SI-7	Energy	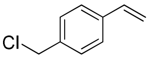 SI-15	Meryer	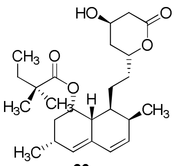 66	Energy
			Energy	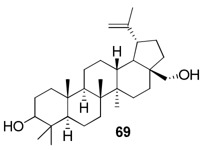 69	Energy

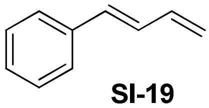

SI-19

The method was following the reference.[1] ^{1}H NMR (400 MHz, $CDCl_3$) δ 7.41 (d, *J* = 8.0 Hz, 2H), 7.31 (t, *J* = 8.0 Hz, 2H), 7.22 (t, *J* = 8.0 Hz, 1H), 6.82 (dd, J_1 = 12.0 Hz,

J_2 = 12.0 Hz, 1H), 6.60-6.43 (m, 2H), 5.35 (d, J = 16.0 Hz, 1H), 5.18 (d, J = 12.0 Hz, 1H). ^{13}C NMR (400 MHz, $CDCl_3$) δ 137.21, 137.16, 132.89, 129.66, 128.64, 127.66, 126.47, 117.64.

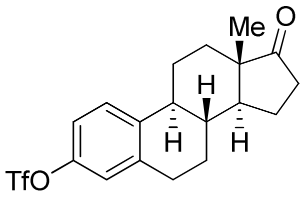

SI-20

The method was following the reference.[2] ^{1}H NMR (400 MHz, $CDCl_3$) δ 7.35 (d, J = 8.0 Hz, 1H), 7.04 (d, J = 8.0 Hz, 1H), 7.00 (s, 1H), 2.99-2.89 (m, 2H), 2.53 (dd, J_1 = 8.0 Hz, J_2 = 8.0 Hz, 1H), 2.45-2.36 (m, 1H), 2.36-2.26 (m, 1H), 2.23-1.96 (m, 4H), 1.71-1.47 (m, 6H), 0.92 (s, 3H). ^{13}C NMR (400 MHz, $CDCl_3$) δ 220.40, 147.60, 140.30, 139.32, 127.20, 121.23, 118.29, 50.38, 47.85, 44.10, 37.75, 35.79, 31.49, 29.38, 26.91, 26.08, 25.68, 21.56, 13.78. ^{19}F (376 MHz, $CDCl_3$) δ -73.01.

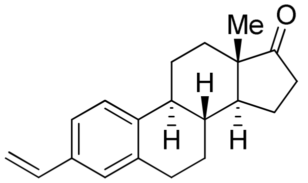

64

The method was following the reference.[2] ^{1}H NMR (400 MHz, $CDCl_3$) δ 7.29-7.17 (m, 2H), 7.14 (s, 1H), 6.67 (dd, J_1 = 12.0 Hz, J_2 = 8.0 Hz, 1H), 5.72 (d, J = 16.0 Hz, 1H), 5.20 (d, J = 8.0 Hz, 1H), 2.98-2.85 (m, 2H), 2.52 (dd, J_1 = 8.0 Hz, J_2 = 8.0 Hz, 1H), 2.46-2.39 (m, 1H), 2.34-2.25 (m, 1H), 2.19-1.93 (m, 4H), 1.65-1.38 (m, 6H), 0.91 (s, 3H). ^{13}C NMR (400 MHz, $CDCl_3$) δ 220.89, 139.55, 136.59, 135.22, 126.89, 125.56, 123.62, 123.62, 113.20, 50.52, 48.01, 44.46, 38.18, 35.88, 31.61, 29.40, 26.52, 25.74, 21.61, 13.87.

General procedure for Methylation and Ethylation of Vinyl Arenes

General procedure for Table 2:

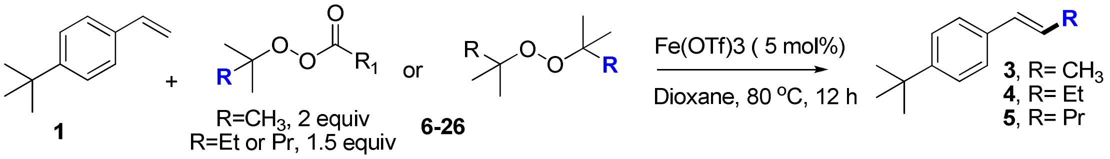

4-Tert-butylstyrene **1** (80 mg, 0.5 mmol, 1 equiv), peroxide **6-26** (0.75 mmol, 1.5 equiv) or 1 mmol (2 equiv) respectively), Fe(OTf)$_3$ (12.6 mg, 5 mol%) and 1,4-dioxane (2 mL) were added into a flame dried schlenk tube with a stirring bar. The reaction mixture was heated to 80 ^{o}C for 12 hours. Then, the reaction mixture was cooled to ambient temperature. The reaction solution was diluted with 30 mL CH_2Cl_2 and filtrated through the diatomite. The filtrate was concentrated by rotary evaporator under reduced pressure and the residue was purified by column chromatography on silica gel (n-pentane) to yield **3**, **4** and **5**.

When different peroxides were used as methylation agent, different isolated yield was given. Details are in **SI-table 1**.

SI-table 1. The isolated yield of methylation product when different peroxide were used

Entry	Olefins	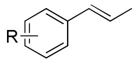	
		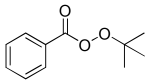	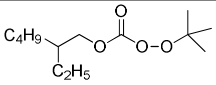
1	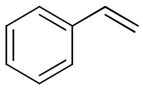	80%	79%
2	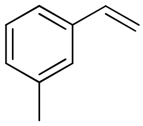	80%	74%
3	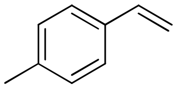	83%	83%

4	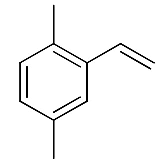	79%	81%
5	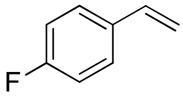	71%	75%
6	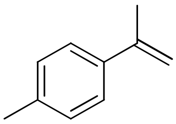	67% E/Z=3.8:1	76% E/Z=3.4:1
7	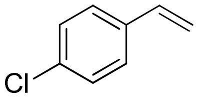	78%	76%
8	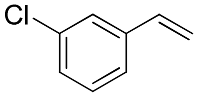	84%	80%
9	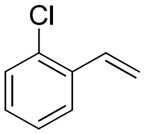	82%	78%[a]
10	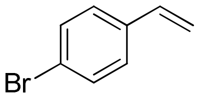	79%	80%
11	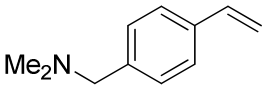	80%	77%
12	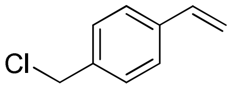	53%	59%

a: at 100 ^{o}C

SI-table 2. The result for the different methods of methylaton or ethylation system.

	Reacton system	Product / GC yield (%)
1 [a]	CH_3I(Co)	**3**/49
2 [a]	C_2H_5I(Co)	**4**/57
3 [b]	CH_3I (Pd)	**3**/trace
4 [b]	C_2H_5(Pd)	**4**/41
5 [c]	CH_3I (Pd+LiI)	**3**/trace
6 [c]	C_2H_5I(Pd+LiI)	**4**/ 62

[a] See detailed procedure in reference 3.

[b] See detailed procedure in reference 4.

[c] See detailed procedure in reference 5.

General procedure for Table 3:

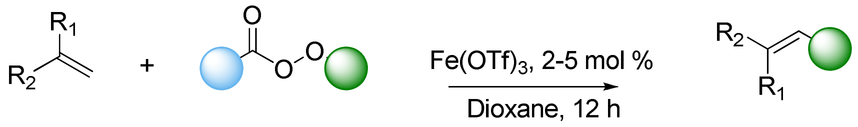

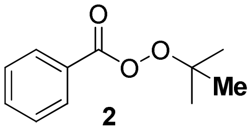

2

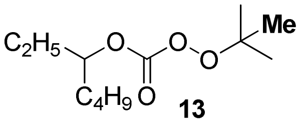

13

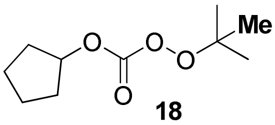

18

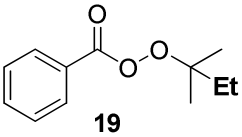

19

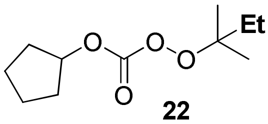

22

Olefin **1** (0.5 mmol, 1 equiv), peroxide (1mmol , 2 equiv), $Fe(OTf)_3$ (12.6 mg, 5 mol%) and 1,4-dioxane (2 mL) were added into a flame dried schlenk tube with a stirring bar. The reaction mixture was heated to 80 °C for 12 hours. Then, the reaction mixture was cooled to ambient temperature. The reaction solution was diluted with 30 mL CH_2Cl_2 and filtrated through the diatomite. The filtrate was concentrated by rotary evaporation under reduced pressure and the residue was purified by column chromatography on silica gel (n-pentane/ethyl acetate = 100/0.1~3/1) to yield **28-71**.

Characterization data for products

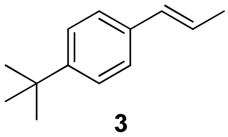

3

Following the general procedure for Table 2 (74 mg, 85% yield, clear oil, **2** as the methylation reagent (2 equiv)). ^{1}H NMR (400 MHz, $CDCl_3$) δ 7.32-7.22 (m, 4H), 6.40 (d, J = 20.0 Hz, 1H), 6.23-6.14 (m, 1H), 1.87 (d, J = 4.0 Hz, 3H), 1.31 (s, 9H). ^{13}C NMR (100 MHz, $CDCl_3$) δ 149.71, 135.21, 130.72, 125.53, 125.40, 124.91, 34.50, 31.35, 18.53.

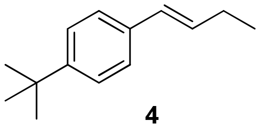

4

Following the general procedure for Table 2 (93 mg, 99% yield, clear oil, **19** as the ethylation reagent (1.5 equiv)). ^{1}H NMR (400 MHz, $CDCl_3$) δ 7.35-7.27 (m, 4H), 6.37 (d, J = 16.0 Hz, 1H), 6.26−6.19 (m, 1H), 2.25-2.16 (m, 2H), 1.31 (d, J = 4.0 Hz, 3H), 1.31 (s, 9H), 1.08 (t, J = 8.0 Hz, 3H). ^{13}C NMR (100 MHz, $CDCl_3$) δ 149.75, 135.20, 131.93, 128.48, 125.60, 125.40, 34.50, 31.34, 26.11, 13.78.

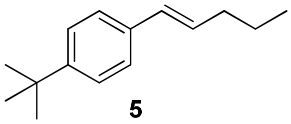

5

Following the general procedure for Table 2 (91 mg, 90% yield, clear oil, **26** as the propylation reagent (1.5 equiv)). ^{1}H NMR (400 MHz, $CDCl_3$) δ 7.40−7.26 (m, 4H), 6.34 (d, J = 16.0 Hz, 1H), 6.27−6.12 (m, 1H), 2.18 (q, J = 8.0 Hz, 2H), 1.49 (q, J = 8.0 Hz, 2H), 1.31 (s, 9H), 0.94 (t, J = 8.0 Hz, 3H). ^{13}C NMR (400 MHz, $CDCl_3$) δ 149.77, 135.21, 130.24, 129.60, 125.62, 125.40, 35.17, 34.50, 31.35, 22.64, 13.75.

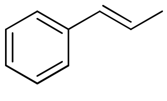

28

Following the general procedure for Table 3 (48 mg, 80% yield, clear oil, **2** as the methylation reagent (2 equiv)). ^{1}H NMR (400 MHz, $CDCl_3$) δ 7.37−7.14 (m, 5H), 6.38 (d, J = 16.0 Hz, 1H), 6.29−6.19 (m, 1H), 1.87 (d, J = 8.0 Hz, 3H). ^{13}C NMR (400 MHz, $CDCl_3$) δ 137.94, 131.02, 128.50, 126.76, 125.83, 125.74, 18.55.

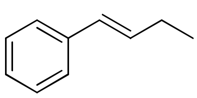

29

Following the general procedure for Table 3 (61 mg, 92% yield, clear oil, **19** as the ethylation reagent (1.5 equiv)). ^{1}H NMR (400 MHz, $CDCl_3$) δ 7.33 (d, *J* = 8.0 Hz, 2H), 7.28 (d, *J* = 8.0 Hz, 2H), 7.18 (t, *J* = 8.0 Hz, 1H), 6.36 (d, *J* = 12.0 Hz, 1H), 6.32−6.22 (m, 1H) , 2.27−2.14 (m, 2H), 1.09 (t, *J* = 8.0 Hz, 3H). ^{13}C NMR (400 MHz, $CDCl_3$) δ 137.96, 132.67, 128.80, 128.50, 126.78, 125.93, 26.10, 13.69.

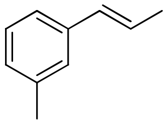

30

Following the general procedure for Table 3 (53 mg, 80% yield, clear oil, **2** as the methylation reagent (2 equiv)).^{1}H NMR (400 MHz, $CDCl_3$) δ 7.18−7.12 (m, 3H), 7.01 (d, *J* = 8.0 Hz, 1H), 6.39 (d, *J* = 16.0 Hz, 1H), 6.26-6.18 (m, 1H), 2.33 (s, 3H), 1.86 (d, *J* = 8.0 Hz, 3H). ^{13}C NMR (100 MHz, $CDCl_3$) δ 137.99, 137.89, 131.07, 128.39, 127.55, 126.60, 125.51, 122.96, 21.45, 18.54.

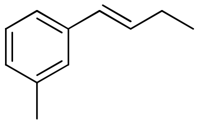

31

Following the general procedure for Table 3 (68 mg, 92% yield, clear oil, **19** as the ethylation reagent (1.5 equiv)).^{1}H NMR (400 MHz, $CDCl_3$) δ 7.20−7.13 (m, 3H), 7.01 (d, *J* = 8.0 Hz, 1H), 6.36 (d, *J* = 16.0 Hz, 1H), 6.28-6.21 (m, 1H), 2.33 (s, 3H), 2.25-2.18 (m, 2H), 1.08 (t, *J* = 8.0 Hz, 3H). ^{13}C NMR (100 MHz, $CDCl_3$) δ 138.00, 137.91, 132.48, 128.86, 128.41, 127.58, 126.68, 123.09, 26.13, 21.46, 13.74. HRMS (EI+) calcd for $[C_{11}H_{14}]^+$ ($[M]^+$): 146.1096, found: 146.1090.

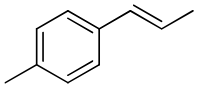

32

Following the general procedure for Table 3 (55 mg, 83% yield, clear oil, **2** as the methylation reagent (2 equiv)). ^{1}H NMR (400 MHz, $CDCl_3$) δ 7.23 (d, *J* = 8.0 Hz, 2H), 7.12 (d, *J* = 8.0 Hz, 2H), 6.38 (d, *J* = 16.0 Hz, 1H), 6.22-6.14 (m, 1H), 2.32 (s, 3H), 1.87 (d, *J* = 4.0 Hz, 3H). ^{13}C NMR (100 MHz, $CDCl_3$) δ 136.42, 135.15, 130.82, 129.19, 125.71, 124.66, 21.16, 18.51.

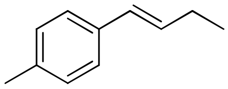

33

Following the general procedure for Table 3 (70 mg, 95% yield, clear oil, **19** as the ethylation reagent (1.5 equiv)). ^{1}H NMR (400 MHz, $CDCl_3$) δ 7.25 (d, *J* = 8.0 Hz, 2H), 7.10 (d, *J* = 8.0 Hz, 2H), 6.36 (d, *J* = 16.0 Hz, 1H), 6.24-6.17 (m, 1H), 2.32 (s, 3H), 2.25-2.16 (m, 2H), 1.08 (t, *J* = 8.0 Hz, 3H). ^{13}C NMR (100 MHz, $CDCl_3$) δ 136.43, 135.17, 131.64, 129.18, 128.60, 125.81, 26.07, 21.15, 13.74.

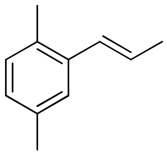

34

Following the general procedure for Table 3 (59 mg, 81% yield, clear oil, **13** as the methylation reagent (2 equiv)). ^{1}H NMR (400 MHz, $CDCl_3$) δ 7.22 (s, 1H), 7.02 (d, *J* = 8.0 Hz, 1H), 6.94 (d, *J* = 8.0 Hz, 1H), 6.58 (d, *J* = 16.0 Hz, 1H), 6.12-6.07 (m, 1H), 2.30 (s, 3H), 2.28 (s, 3H), 1.90 (d, *J* = 4.0 Hz, 3H). ^{13}C NMR (100 MHz, $CDCl_3$) δ 136.79, 135.31, 131.75, 130.06, 128.92, 127.49, 126.69, 126.07, 21.07, 19.37, 18.86.

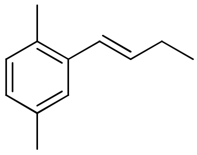

35

Following the general procedure for Table 3 (75 mg, 93% yield, clear oil, **19** as the ethylation reagent (1.5 equiv)). ^{1}H NMR (400 MHz, $CDCl_3$) δ 7.23 (s, 1H), 7.02 (d, *J* = 8.0 Hz, 1H), 6.93 (d, *J* = 4.0 Hz, 1H), 6.56 (d, *J* = 16.0 Hz, 1H), 6.14-6.10 (m, 1H), 2.30 (s, 3H), 2.28 (s, 3H), 2.24-2.22 (m, 2H), 1.10 (t, *J* = 8.0 Hz, 3H). ^{13}C NMR (100 MHz, $CDCl_3$) δ 136.78, 135.31, 133.73, 131.87, 130.08, 127.50, 126.68, 126.08, 26.44, 21.06, 19.36, 13.90. HRMS (EI+) calcd for $[C_{12}H_{16}]^+$ ($[M]^+$): 160.1252, found: 160.1245.

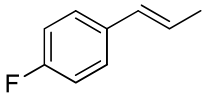

36

Following the general procedure for Table 3 (51 mg, 75% yield, clear oil, **13** as the methylation reagent (2 equiv)). ^{1}H NMR (400 MHz, $CDCl_3$) δ 7.29 (t, *J* = 8.0 Hz, 2H), 6.97 (t, *J* = 8.0 Hz, 2H), 6.37 (d, *J* = 12.0 Hz, 1H), 6.19-6.10 (m, 1H), 1.87 (d, *J* = 4.0 Hz, 3H). ^{13}C NMR (100 MHz, $CDCl_3$) δ 163.05, 160.61, 134.09, 134.05, 129.84,

127.24, 127.16, 125.46, 125.44, 115.42, 115.21, 18.46. ^{19}F (376 MHz, $CDCl_3$) δ -155.99.

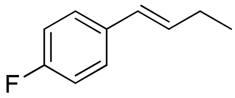

37

Following the general procedure for Table 2 (63 mg, 84% yield, clear oil, **19** as the ethylation reagent (1.5 equiv)). ^{1}H NMR (400 MHz, $CDCl_3$) δ 7.31 (t, *J* = 8.0 Hz, 2H), 6.99 (t, *J* = 8.0 Hz, 2H), 6.35 (d, *J* = 16.0 Hz, 1H), 6.21-6.14 (m, 1H), 2.25−2.18 (m, 2H), 1.08 (d, *J* = 8.0 Hz, 3H). ^{13}C NMR (100 MHz, $CDCl_3$) δ 163.08, 160.64, 134.10, 132.37, 127.63, 127.33, 127.26, 115.41, 115.20, 26.02, 13.65. ^{19}F (376 MHz, $CDCl_3$) δ -155.98.

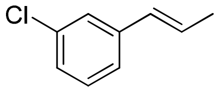

38

Following the general procedure for Table 3 (64 mg, 84% yield, clear oil, **2** as the methylation reagent (2 equiv)). ^{1}H NMR (400 MHz, $CDCl_3$) δ 7.31 (s, 1H), 7.21-7.14 (m, 3H), 6.36-6.23 (m, 2H), 1.89 (d, *J* = 4.0 Hz, 3H). ^{13}C NMR (100 MHz, $CDCl_3$) δ 139.80, 134.40, 129.80, 129.69, 127.41, 126.68, 125.77, 124.04, 18.52.

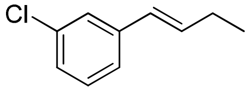

39

Following the general procedure for Table 3 (73 mg, 87% yield, clear oil, **19** as the ethylation reagent (1.5 equiv)). ^{1}H NMR (400 MHz, $CDCl_3$) δ 7.33 (s, 1H), 7.21-7.14 (m, 3H), 6.34−6.28 (m, 2H), 2.24-2.20 (m, 2H), 1.09 (t, *J* = 8.0 Hz, 3H). ^{13}C NMR (100 MHz, $CDCl_3$) δ 139.85, 134.41, 134.26, 129.68, 127.59, 126.69, 125.85, 124.16, 26.05, 13.51.

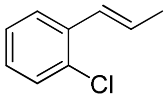

40

Following the general procedure for Table 3 (62 mg, 82% yield, clear oil, **2** as the methylation reagent (2 equiv), at 100 ^{o}C for 12 h). ^{1}H NMR (400 MHz, $CDCl_3$) δ 7.49 (d, *J* = 8.0 Hz, 1H), 7.33 (d, *J* = 8.0 Hz, 1H), 7.21 (d, *J* = 8.0 Hz, 1H), 7.14 (d, *J* = 8.0 Hz, 1H), 6.79 (d, *J* = 16.0 Hz, 1H), 6.29-6.18 (m, 1H), 1.94 (d, *J* = 8.0 Hz, 3H). ^{13}C

NMR (100 MHz, $CDCl_3$) δ 135.96, 132.38, 129.58, 128.77, 127.82, 127.29, 126.76, 126.59, 18.81.

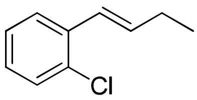

41

Following the general procedure for Table 3 (69 mg, 82% yield, clear oil, **19** as the ethylation reagent (1.5 equiv)). ^{1}H NMR (400 MHz, $CDCl_3$) δ 7.51 (d, *J* = 8.0 Hz, 1H), 7.33 (d, *J* = 8.0 Hz, 1H), 7.18 (t, *J* = 8.0 Hz, 1H), 7.12 (t, *J* = 8.0 Hz, 1H), 6.73 (d, *J* = 16.0 Hz, 1H), 6.28-6.21 (m, 1H), 2.31−2.24 (m, 2H), 1.11 (t, *J* = 8.0 Hz, 3H). ^{13}C NMR (100 MHz, $CDCl_3$) δ 135.98, 135.58, 132.55, 129.59, 127.82, 126.75, 126.59, 125.11, 26.32, 13.57.

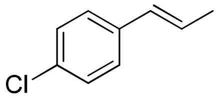

42

Following the general procedure for Table 3 (60 mg, 78% yield, clear oil, **2** as the methylation reagent (2 equiv)). ^{1}H NMR (400 MHz, $CDCl_3$) δ 7.24 (d, *J* = 4.0 Hz, 4H), 6.33 (d, *J* = 16.0 Hz, 1H), 6.29−6.16 (m, 1H), 1.86 (d, *J* = 8.0 Hz, 3H). ^{13}C NMR (400 MHz, $CDCl_3$) δ 136.41, 132.25, 129.87, 128.60, 127.02, 126.49, 18.51.

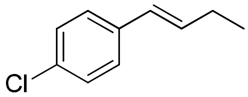

43

Following the general procedure for Table 3 (74 mg, 89% yield, clear oil, **19** as the ethylation reagent (1.5 equiv)). ^{1}H NMR (400 MHz, $CDCl_3$) δ 7.25 (s, 4H), 6.38−6.18 (m, 2H), 2.28−2.16 (m, 2H), 1.09 (t, *J* = 8.0 Hz, 3H). ^{13}C NMR (400 MHz, $CDCl_3$) δ 136.44, 133.38, 132.26, 128.58, 127.64, 127.11, 26.04, 13.54.

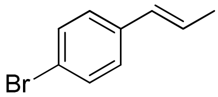

44

Following the general procedure for Table 3 (78 mg, 80% yield, clear oil, **13** as the methylation reagent (2 equiv)). ^{1}H NMR (400 MHz, $CDCl_3$) δ 7.41 (d, *J* = 8.0 Hz, 2H), 7.17 (d, *J* = 12.0 Hz, 2H), 6.35 (d, *J* = 16.0 Hz, 1H), 6.27-6.18 (m, 1H), 1.88 (d, *J* = 8.0 Hz, 3H). ^{13}C NMR (100 MHz, $CDCl_3$) δ 136.85, 131.54, 129.92, 127.38, 126.66, 120.35, 18.54.

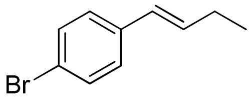

45

Following the general procedure for Table 3 (87 mg, 83% yield, clear oil, **19** as the ethylation reagent (1.5 equiv)). ^{1}H NMR (400 MHz, $CDCl_3$) δ 7.41 (d, *J* = 12.0 Hz, 2H), 7.20 (d, *J* = 8.0 Hz, 2H), 6.33-6.23 (m, 1H), 2.25-2.21 (m, 2H), 1.08 (d, *J* = 8.0 Hz, 3H). ^{13}C NMR (100 MHz, $CDCl_3$) δ 136.89, 133.53, 131.54, 127.70, 120.48, 26.08, 13.53.

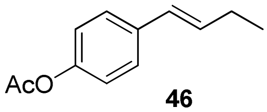

46

Following the general procedure for Table 3 (47 mg, 50% yield, clear oil, **19** as the ethylation reagent (1.5 equiv), at -15 °C~25 °C for 5 h). ^{1}H NMR (400 MHz, $CDCl_3$) δ 7.34 (d, *J* = 8.0 Hz, 2H), 7.01 (d, *J* = 8.0 Hz, 2H), 6.37 (d, *J* = 16.0 Hz, 1H), 6.15-6.17 (m, 1H), 2.28 (s, 3H), 2.25-2.18 (m, 2H), 1.08 (t, *J* = 8.0 Hz, 3H). ^{13}C NMR (100 MHz, $CDCl_3$) δ 168.51, 148.39, 134.76, 131.89, 126.81, 125.75, 120.48, 24.99, 20.09, 12.58. HRMS (EI+) calcd for $[C_{12}H_{14}O_2]^+$ ($[M]^+$): 190.0994, found: 190.0986.

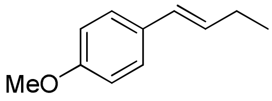

47

Following the general procedure for Table 3 (55 mg, 67% yield, clear oil, **19** as the ethylation reagent (1.2 equiv), $Fe(OTf)_3$ (2.5 mol%), at -15 °C~65 °C for 9 h). ^{1}H NMR (400 MHz, $CDCl_3$) δ 7.28 (d, *J* = 8.0 Hz, 2H), 6.84 (d, *J* = 8.0 Hz, 2H), 6.34 (d, *J* = 16.0 Hz, 1H), 6.16-6.09 (m, 1H), 3.79 (s, 3H), 2.24-2.17 (m, 2H), 1.08 (t, *J* = 8.0 Hz, 3H). ^{13}C NMR (100 MHz, $CDCl_3$) δ 158.61, 130.82, 130.55, 128.13, 126.97, 113.92, 55.29, 26.04, 13.81.

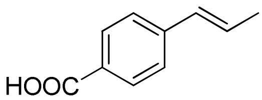

48

Following the general procedure for Table 3 (49 mg, 60% yield, white solid, **18** as the methylation reagent (2 equiv)). ^{1}H NMR (400 MHz, DMSO-d^6) δ 7.88 (d, *J* = 8.0 Hz, 2H), 7.50 (d, *J* = 8.0 Hz, 2H), 6.52-6.42 (m, 2H), 1.88 (d, *J* = 4.0 Hz, 3H). ^{13}C NMR (100 MHz, DMSO-d^6) δ 167.60, 142.20, 130.57, 130.14, 129.37, 129.20, 126.17, 18.90. HRMS (EI+) calcd for $[C_{10}H_{10}O_2]^+$ ($[M]^+$): 162.0681, found: 162.0685.

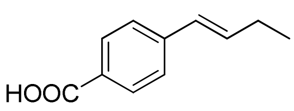

49

Following the general procedure for Table 3 (62 mg, 70% yield, white solid, **22** as the ethylation reagent (1.5 equiv)). ^{1}H NMR (400 MHz, DMSO-d^6) δ 7.88 (d, *J* = 8.0 Hz, 2H), 7.51 (d, *J* = 8.0 Hz, 2H), 6.55-6.44 (m, 2H), 2.27-2.20 (m, 2H), 1.06 (t, *J* = 8.0 Hz, 3H). ^{13}C NMR (100 MHz, DMSO-d^6) δ 167.62, 142.20, 135.82, 130.14, 128.36, 126.27, 26.05, 13.77. HRMS (EI+) calcd for $[C_{11}H_{12}O_2]^+$ ($[M]^+$): 176.0837, found: 176.0840.

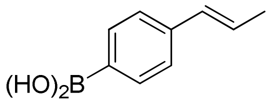

50

Following the general procedure for Table 3 (47 mg, 57% yield, white solid, **18** as the methylation reagent, (2 equiv)). ^{1}H NMR (400 MHz, DMSO-d^6) δ 7.97 (s, 2H), 7.73 (d, *J* = 8.0 Hz, 2H), 7.34 (d, *J* = 8.0 Hz, 2H), 6.43-6.30 (m, 2H), 1.86 (t, *J* = 8.0 Hz, 3H). ^{13}C NMR (100 MHz, DMSO-d^6) δ 139.38, 134.90, 131.45, 126.59, 125.20, 18.82. HRMS (ESI) calcd for $[C_9H_{10}BO_2]^-$ ($[M\text{-}H]^-$): 161.0779, found: 161.0781.

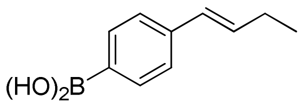

51

Following the general procedure for Table 3 (60 mg, 68% yield, white solid, **22** as the ethylation reagent (1.5 equiv)). ^{1}H NMR (400 MHz, DMSO-d^6) δ 7.97 (s, 2H), 7.74 (d, *J* = 8.0 Hz, 2H), 7.35 (d, *J* = 8.0 Hz, 2H), 6.41-6.35 (m, 2H), 2.21-2.20 (m, 2H), 1.05 (t, *J* = 8.0 Hz, 3H). ^{13}C NMR (100 MHz, DMSO-d^6) δ 139.35, 134.91, 133.34, 129.21, 125.29, 26.00, 13.96. HRMS (ESI) calcd for $[C_{10}H_{12}BO_2]^-$ ($[M\text{-}H]^-$): 175.0938, found: 175.0931.

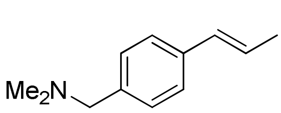

52

Following the general procedure for Table 3 (70 mg, 80% yield, light yellow liquid, **2** as the methylation reagent (2 equiv)). ^{1}H NMR (400 MHz, $CDCl_3$) δ 7.29 (d, *J* = 8.0 Hz, 2H), 7.23 (d, *J* = 8.0 Hz, 2H), 6.41 (d, *J* = 16.0 Hz, 1H), 6.27-6.16 (m, 1H), 3.39 (s, 2H), 2.23 (s, 6H), 1.86 (d, *J* = 8.0 Hz, 3H). ^{13}C NMR (100 MHz, $CDCl_3$) δ 137.34, 136.81, 130.76, 129.31, 125.69, 125.41, 64.13, 45.35, 18.51. HRMS (EI+) calcd for $[C_{12}H_{17}N]^+$ ($[M]^+$): 175.1361, found: 175.1355.

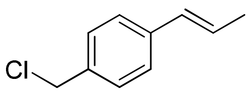

53

Following the general procedure for Table 3 (49 mg, 59% yield, clear oil, **13** as the methylation reagent (2 equiv)). ^{1}H NMR (400 MHz, $CDCl_3$) δ 7.30 (s, 4H), 6.41 (d, *J* = 16.0 Hz, 1H), 6.30-6.21 (m, 1H), 4.57 (s, 2H), 1.88 (d, *J* = 8.0 Hz, 3H). ^{13}C NMR (100 MHz, $CDCl_3$) δ 138.20, 135.79, 130.42, 128.86, 126.63, 126.14, 46.27, 18.56.

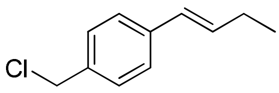

54

Following the general procedure for Table 3 (73 mg, 81% yield, clear oil, **19** as the ethylation reagent (1.5 equiv)). ^{1}H NMR (400 MHz, $CDCl_3$) δ 7.34-7.29 (m, 4H), 6.39 (d, *J* = 16.0 Hz, 1H), 6.32-6.25 (m, 1H), 4.57 (s, 2H), 2.27-2.20 (m, 2H), 1.09 (t, *J* = 8.0 Hz, 3H). ^{13}C NMR (100 MHz, $CDCl_3$) δ 138.23, 135.81, 133.54, 128.86, 128.18, 126.23, 46.28, 26.10, 13.59. HRMS (EI+) calcd for $[C_{11}H_{13}Cl]^+$ ($[M]^+$): 180.0706, found: 180.0702.

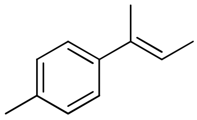

55

Following the general procedure for Table 3 (55 mg, 76% yield, clear oil, E/Z=3.4:1, **13** as the methylation reagent (2 equiv)). ^{1}H NMR (400 MHz, $CDCl_3$) δ 7.28 (d, *J* = 8.0 Hz, 2H), 7.12 (d, *J* = 8.0 Hz, 2H), 5.85−5.80 (m, 1H), 2.33 (s, 3H), 2.01 (s, 3H), 1.79 (d, *J* = 8.0 Hz, 3H). ^{13}C NMR (100 MHz, $CDCl_3$) δ 141.18, 136.02, 135.29, 128.86, 125.40, 121.65, 21.03, 15.51, 14.33.

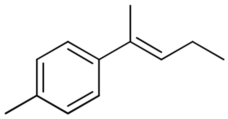

56

Following the general procedure for Table 3 (60 mg, 74% yield, clear oil, E/Z=3.6:1, **19** as the ethylation reagent (1.1 equiv)). ^{1}H NMR (400 MHz, $CDCl_3$) δ 7.27 (d, *J* = 8.0 Hz, 2H), 7.10 (d, *J* = 8.0 Hz, 2H), 5.74 (t, *J* = 8.0 Hz, 1H), 2.33 (s, 3H), 2.23−2.17 (m, 2H), 2.01 (s, 3H), 1.05 (t, *J* = 8.0 Hz, 3H). ^{13}C NMR (400 MHz, $CDCl_3$) δ 141.12, 136.05, 133.82, 129.49, 128.84, 125.45, 22.04, 21.03, 15.64, 14.17.

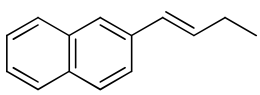

57

Following the general procedure for Table 3 (59 mg, 64% yield, clear oil, **19** as the ethylation reagent (1.5 equiv)). ^{1}H NMR (400 MHz, $CDCl_3$) δ 7.77 (t, *J* = 8.0 Hz, 3H), 7.67 (s, 1H), 7.59 (d, *J* = 8.0 Hz, 1H), 7.46−7.38 (m, 2H), 6.56 (d, *J* = 16.0 Hz, 1H), 6.44-6.37(m, 1H), 2.32-2.26 (m, 2H), 1.13 (d, *J* = 8.0 Hz, 3H). ^{13}C NMR (100 MHz, $CDCl_3$) δ 135.42, 133.74, 133.17, 132.65, 128.92, 128.05, 127.83, 127.64, 126.11, 125.42, 125.32, 123.58, 26.22, 13.71.

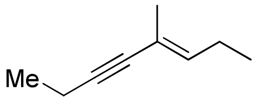

58

Following the general procedure for Table 3 (31 mg, 50% yield, clear oil, E/Z=8:1, **2** as the ethylation reagent (1.1 equiv), $Fe(OTf)_3$ (2.5 mol%), THF (2 mL), at -15 ^{o}C~25 ^{o}C for 6 h). ^{1}H NMR (400 MHz, $CDCl_3$) δ 5.56 (d, *J* = 8.0 Hz, 1H), 2.36 (q, *J* = 8.0 Hz, 2H), 2.26−2.17 (m, 2H), 1.80 (s, 3H), 1.18 (t, *J* = 8.0 Hz, 3H), 0.97 (t, *J* = 8.0 Hz, 3H). ^{13}C NMR (100 MHz, $CDCl_3$) δ 138.21, 117.46, 94.92, 79.09, 23.79, 23.39, 14.21, 13.66, 13.16. HRMS (EI+) calcd for $[C_9H_{14}]^+$ ($[M]^+$): 122.1096, found: 122.1091.

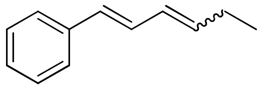

59

Following the general procedure for Table 3 (50 mg, 63% yield, clear oil, E/Z=2.3:1, **22** as the ethylation reagent (1.0 equiv), $Fe(OTf)_3$ (2.5 mol%), THF (2 mL), at -15 ^{o}C for 4 h). ^{1}H NMR (400 MHz, $CDCl_3$) δ 7.44-7.34 (m, 2H), 7.33-7.26 (m, 2H), 7.23-7.16 (m, 1H), 7.11-7.01 (m, 0.34H), 6.80-6.70 (m, 0.68H), 6.56-6.41 (m, 1.03H), 6.26-6.07 (m, 0.93H), 5.94-5.79 (m, 0.65H), 5.60-5.45 (m, 0.28H), 2.35-2.27 (m, 0.63H), 2.22-2.11 (m, 1.47H), 1.05 (t, *J* = 8.0 Hz, 3H). ^{13}C NMR (100 MHz, $CDCl_3$) δ 137.72, 137.42, 134.89, 131.98, 129.99, 129.57, 128.59, 128.55, 128.11, 127.34, 127.07, 126.33, 126.14, 124.39, 25.89, 21.36, 14.34, 13.55.

Synthetic applications of natural products and drug molecules

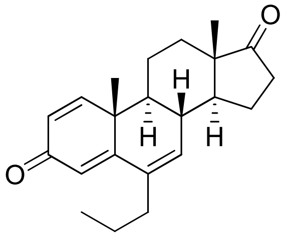

61a

Following the general procedure for Table 3 (99 mg, 61% yield, light yellow oil, **22** as the ethylation reagent (1.65 equiv), THF (2 mL), at 0 °C for 2 h and 40 °C for 14 h). ^{1}H NMR (400 MHz, $CDCl_3$) δ 7.09 (d, *J* = 12.0 Hz, 1H), 6.30 (d, *J* = 12.0 Hz, 1H), 6.25 (s, 1H), 5.87 (s, 1H), 2.56-2.50 (m, 1H), 2.41 (d, *J* = 8.0 Hz, 2H), 2.16-2.13 (m, 3H), 1.93-1.90 (m, 2H), 1.75-1.64 (m, 2H), 1.52-1.43 (m, 4H), 1.35−1.32 (m, 1H), 1.19 (s, 3H), 1.00 (s, 3H), 0.92 (t, *J* = 8.0 Hz, 3H). ^{13}C NMR (100 MHz, $CDCl_3$) δ 219.29, 186.53, 162.08, 153.66, 136.79, 131.87, 127.74, 121.72, 49.08, 48.80, 47.79, 41.28, 37.27, 35.60, 34.68, 31.30, 21.70, 21.44, 21.40, 20.24, 13.91, 13.86. HRMS (EI+) calcd for $[C_{22}H_{28}O_2]^+$ ($[M]^+$): 324.2089, found: 324.2085.

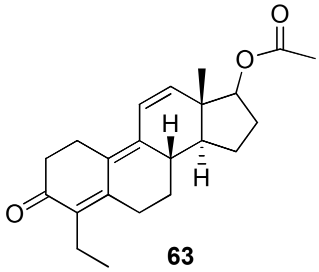

63

Following the general procedure for Table 3 (70 mg, 41% yield, light yellow oil, **22** as the ethylation reagent (1 equiv), THF (2 mL), at -15 °C for 4 h). ^{1}H NMR (400 MHz, $CDCl_3$) δ 6.46 (d, *J* = 8.0 Hz, 1H), 6.32 (d, *J* = 12.0 Hz, 1H), 4.81 (t, *J* = 8.0 Hz, 1H), 2.91−2.79 (m, 2H), 2.76−2.64 (m, 1H), 2.52−2.42 (m, 2H), 2.42−2.26 (m, 5H), 2.09 (s, 3H), 1.97−1.89 (m, 1H), 1.83−1.67 (m, 2H), 1.64−1.48 (m, 3H), 1.29−1.25 (m, 2H), 1.01−0.92 (m, 6H). ^{13}C NMR (400 MHz, $CDCl_3$) δ 198.23, 171.16, 149.87, 140.26, 139.16, 135.83, 128.49, 123.83, 78.64, 47.67, 44.77, 37.02, 36.84, 27.70, 27.51, 26.75, 24.62, 23.24, 21.13, 18.83, 14.50, 13.01. HRMS (EI+) calcd for $[C_{22}H_{28}O_3]^+$ ($[M]^+$): 340.2038, found: 340.2044.

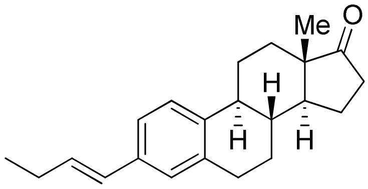

65

Following the general procedure for Table 3 (70 mg, 91% yield, white solid, experiment run on 0.25 mmol scale**, 22** as the ethylation reagent (1.5 equiv), $Fe(OTf)_3$ (5 mol%), dioxane (1 mL), at 80 °C for 10 hours). ^{1}H NMR (400 MHz, $CDCl_3$) δ 7.22 (d, J = 8.0 Hz, 1H), 7.14 (d, J = 8.0 Hz, 1H), 7.07 (s, 1H), 6.38-6.13 (m, 2H), 2.96-2.83 (m, 2H), 2.51 (dd, J_1 = 8.0 Hz, J_2= 8.0 Hz, 1H), 2.56-2.44 (m, 1H), 2.44-2.36 (m, 1H), 2.31-1.93 (m, 7H), 1.66-1.36 (m, 6H), 1.08 (t, J = 8.0 Hz, 3H), 0.90 (s, 3H). ^{13}C NMR (400 MHz, $CDCl_3$) δ 220.96, 138.39, 136.46, 135.61, 132.10, 128.45, 126.53, 125.49, 123.39, 50.52, 48.02, 44.42, 38.24, 35.89, 31.62, 29.43, 26.57, 26.09, 25.76, 21.62, 13.87, 13.75. HRMS (ESI) calcd for $[C_{22}H_{29}O]^+$ ($[M+H]^+$): 309.2213, found: 309.2214.

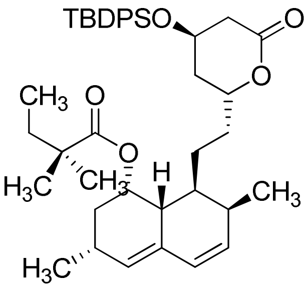

67

The method was following the reference.[6] Experiment run on 4 mmol scale (2309 mg, 88% yield, white solid). ^{1}H NMR (400 MHz, $CDCl_3$) δ 7.67−7.59 (m, 4H), 7.47−7.37 (m, 6H), 5.98 (d, J = 8.0 Hz, 1H), 5.81−5.72 (m, 1H), 5.51 (s, 1H), 5.32 (d, J = 4.0 Hz, 1H), 4.78-4.65 (m, 1H), 4.27 (d, J = 4.0 Hz, 1H), 2.62-2.22 (m, 4H), 2.06-1.61 (m, 6H), 1.60-1.44 (m, 4H), 1.30-1.23 (m, 2H), 1.14-1.06 (m, 18H), 0.87 (d, J = 8.0 Hz, 3H), 0.82 (t, J = 8.0 Hz, 3H). ^{13}C NMR (400 MHz, $CDCl_3$) δ 177.54, 170.16, 135.66, 133.20, 133.10, 132.89, 131.63, 130.08, 129.71, 128.45, 127.92, 68.02, 64.50, 42.96, 38.95, 37.48, 36.75, 36.46, 33.21, 33.04, 32.74, 30.68, 27.35, 26.92, 24.82, 24.72, 24.31, 23.02, 19.10, 13.90, 9.34.

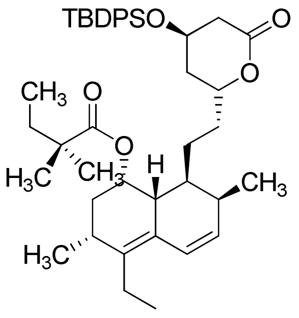

68

Following the general procedure for Table 3 (82 mg, 48% (60% BRSM) yield, clear oil, experiment run on 0.25 mmol scale, **22** as the ethylation reagent (2 equiv), $Fe(OTf)_3$ (10 mol%), THF (2 mL), at ice-salt bath for 2 h, r.t for 2 h, 60 ^oC for 3 h). ^1H NMR (400 MHz, $CDCl_3$) δ 7.65-7.59 (m, 5H), 7.43-7.37 (m, 5H), 6.40 (d, J = 8.0 Hz, 1H), 5.85−5.69 (m, 1H), 5.24 (d, J = 4.0 Hz, 1H), 4.74-4.66 (m, 1H), 4.28-4.22 (m, 1H), 2.53-2.30 (m, 5H), 2.27-2.07 (m, 3H), 1.94-1.71 (m, 6H), 1.57-1.53 (m, 2H), 1.40-1.33 (m, 2H), 1.16-1.14 (m, 2H), 1.13-1.10 (m, 4H), 1.08-1.01 (m, 15H), 0.88 (d, J = 8.0 Hz, 3H), 0.82 (t, J = 8.0 Hz, 3H). ^{13}C NMR (400 MHz, $CDCl_3$) δ 177.69, 170.21, 138.75, 135.65, 133.20, 133.10, 132.86, 130.06, 127.91, 124.89, 123.52, 68.63, 64.55, 64.48, 42.94, 38.94, 38.10, 37.07, 36.53, 33.52, 33.19, 32.98, 30.89, 30.40, 29.70, 26.91, 26.87, 24.84, 24.61, 24.26, 23.79, 22.07, 19.10, 14.34, 13.94, 9.30. HRMS (ESI) calcd for $[C_{43}H_{60}O_5SiNa]^+$ ($[M+Na]^+$): 707.4102, found: 707.4106.

Example of un-conjugated olefin

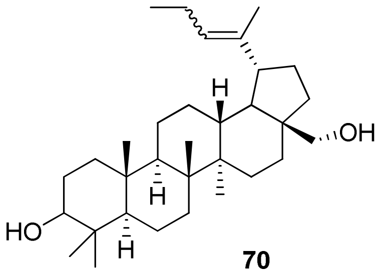
70

Following the general procedure for Table 3 (94 mg, 30% yield (40% RSM), E/Z=1.9:1, white solid, **22** as the ethylation reagent (4 equiv), $Fe(OTf)_3$ (10 mol%), dioxane (2 mL), at 65 °C for 30 min). ^{1}H NMR (400 MHz, $CDCl_3$) δ 5.11 (t, *J* = 8.0 Hz, 0.64H), 4.99 (t, *J* = 8.0 Hz, 0.36H), 4.34−4.33 (m, 1H), 3.80 (d, *J* = 8.0 Hz, 1H), 3.39-3.31 (m, 1H), 3.21-3.17 (m, 1H), 2.78-2.72 (m, 0.33H), 2.34-2.28 (m, 0.67H), 2.00-1.93 (m, 4H), 1.80-1.73 (m, 4H), 1.69-1.64 (m, 3H), 1.62 (s, 3H), 1.60-1.57 (m, 4H), 1.52 (s, 3H), 1.42-1.37 (m, 5H), 1.26 (s, 3H), 1.03-1.02 (m, 4H), 0.97 (s, 6H), 0.94-0.88 (m, 4H), 0.82 (s, 3H), 0.76 (s, 3H). ^{13}C NMR (400 MHz, $CDCl_3$) δ 138.74, 138.51, 126.32, 126.29, 79.01, 78.99, 74.08, 60.70, 60.65, 55.30, 50.47, 50.37, 49.70, 48.25, 47.83, 47.57, 42.76, 42.73, 40.92, 40.90, 40.06, 38.87, 38.77, 38.72, 37.49, 37.29, 37.17, 37.15, 35.61, 34.23, 34.05, 29.71, 29.25, 29.21, 29.03, 27.99, 27.38, 27.08, 27.06, 24.84, 24.67, 23.29, 21.15, 20.89, 20.84, 20.72, 19.53, 18.32, 16.13, 16.10, 16.02, 15.97, 15.37, 14.79, 14.76, 14.57, 13.97. HRMS (ESI) calcd for $[C_{32}H_{55}O_2]^+$ ($[M+H]^+$): 471.4197, found: 471.4191.

Mechanistic study I

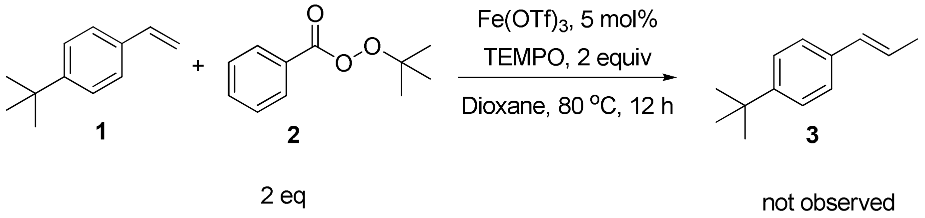

4-tert-butylstyrene **1** (80 mg, 0.5 mmol), Peroxide **2** (194 mg, 1.0 mmol), $Fe(OTf)_3$ (12.6 mg, 5 mol%), TEMPO (156 mg, 1.0 mmol) and 1,4-dioxane (2 mL) were added into a flame dried schlenk tube with a stirring bar and the reaction mixture was heated to 80 °C for 12 hours. The reaction mixture was cooled to ambient temperature and filtered through the diatomite. Then, the solution was detected by GC-MS and the product **3** was not observed.

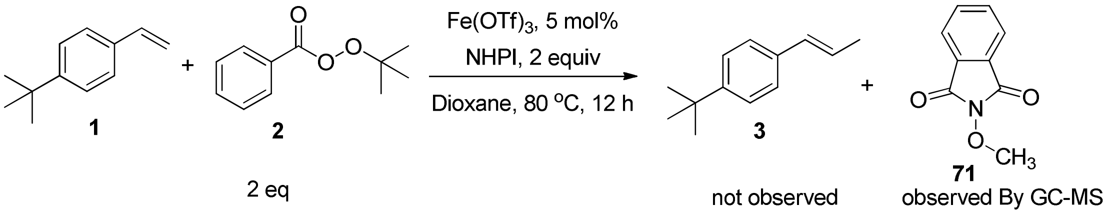

4-tert-butylstyrene **1** (80 mg, 0.5 mmol), Peroxide **2** (194 mg, 1.0 mmol), $Fe(OTf)_3$ (12.6 mg, 5 mol%), NHPI (163 mg, 1.0 mmol) and 1,4-dioxane (2 mL) were added into a flame dried schlenk tube with a stirring bar and the reaction mixture was heated to 80 °C for 12 hours. The reaction mixture was cooled to ambient temperature and filtered through the diatomite. Then, the solution was detected by GC-MS and the product **3** was not observed**. 69** was observed by GC-MS.

Mechanistic study II

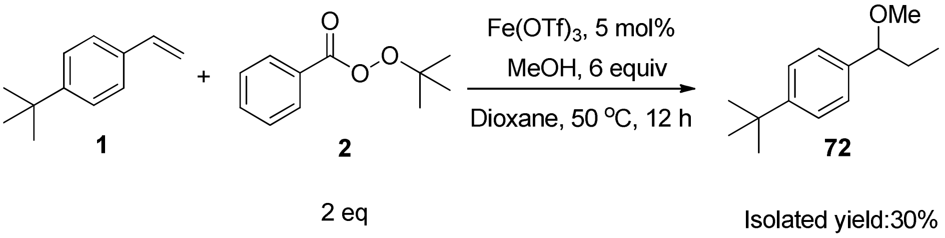

4-tert-butylstyrene **1** (80 mg, 0.5 mmol), Peroxide **2** (194 mg, 1.0 mmol), $Fe(OTf)_3$ (12.6 mg, 5 mol%), CH_3OH (96 mg, 3 mmol) and 1,4-dioxane (2 mL) were added into a flame dried schlenk tube with a stirring bar and the reaction mixture was heated to 50 °C for 12 hours. Then, the reaction mixture was cooled to ambient temperature. The reaction solution was diluted with ethyl acetate and filtrated through the diatomite. The filtrate was concentrated by rotary evaporation under reduced pressure and the residue was purified by column chromatography on silica gel (hexanes/ethyl acetate = 20/1~3/1) to yield **72** (31 mg, 30%).

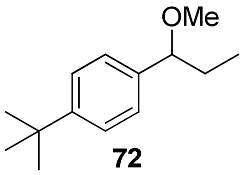

72

^{1}H NMR (400 MHz, $CDCl_3$) δ 7.36 (d, J = 8.0 Hz, 2H), 7.21 (d, J = 8.0 Hz, 2H), 3.99 (t, J = 8.0 Hz, 1H), 3.21 (s, 3H), 1.85-1.78 (m, 1H), 1.70-1.62 (m, 1H), 1.32 (s, 9H), 0.88 (t, J = 8.0 Hz, 3H). ^{13}C NMR (400 MHz, $CDCl_3$) δ 150.25, 139.09, 126.43, 125.13, 85.31, 56.65, 34.50, 31.41, 30.84, 10.31.

Reference

1. X.-J. Wei, D.-T. Yang, L. Wang, T. Song, L.-Z. Wu, Q. Liu, *Org. Lett.* **2013**, 15, 6054.

2. C. Y. Huang, A. G. Doyle, *J. Am. Chem. Soc.* **2012,** 134 (23), 9541–9544.

3. Y. Ikeda, T. Nakamura, H. Yorimitsu; K. Oshima. *J. Am. Chem. Soc.* **2002**, 124, 6514-6515.

4. C.M. Mcmahon, E.J. Alexnian. *Angew. Chem., Int. Ed.* **2014**, 53, 5974-5977.

5. Y. Zou; J. zhou. *Chem. Commun.* **2014**, 50, 3725-3728.

6. R. F. Cunico; L.Bedell. *J. Org. Chem.* **1980**, 45, 4798-4801.

NMR spectra

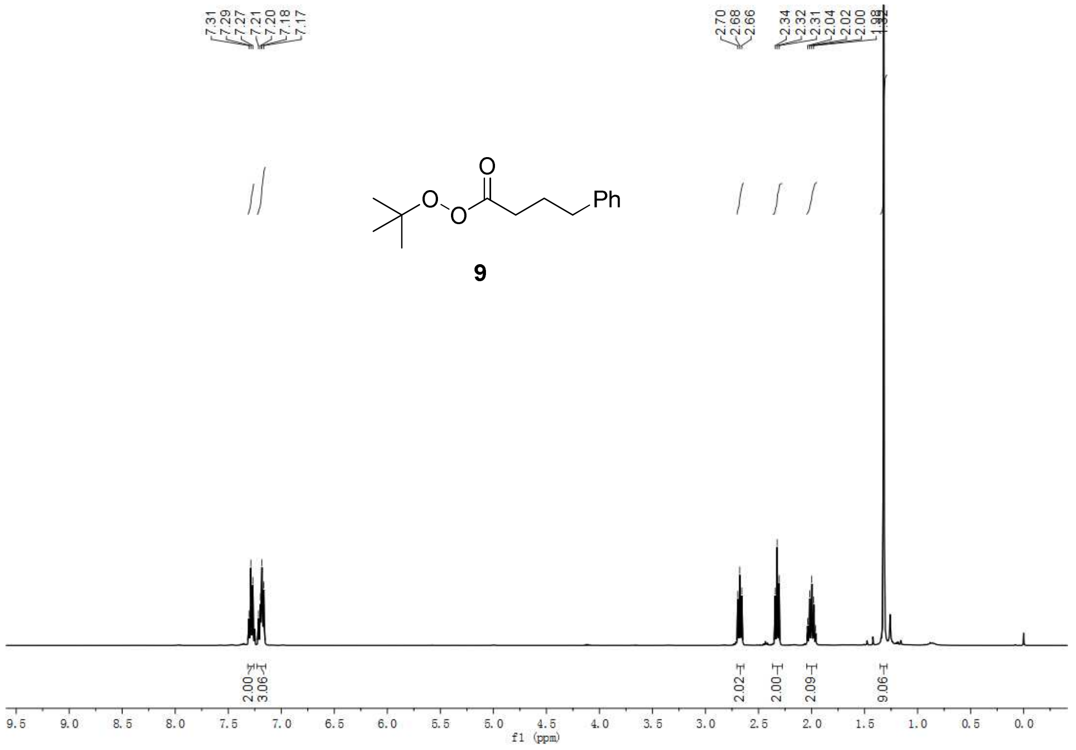
7.31 7.29 7.27 7.21 7.20 7.18 7.17
2.70 2.68 2.66 2.34 2.32 2.31 2.04 2.02 2.00 1.98
O
O
O
Ph
9
2.00 3.06 2.02 2.00 2.09 9.06
9.5 9.0 8.5 8.0 7.5 7.0 6.5 6.0 5.5 5.0 4.5 4.0 3.5 3.0 2.5 2.0 1.5 1.0 0.5 0.0
f1 (ppm)

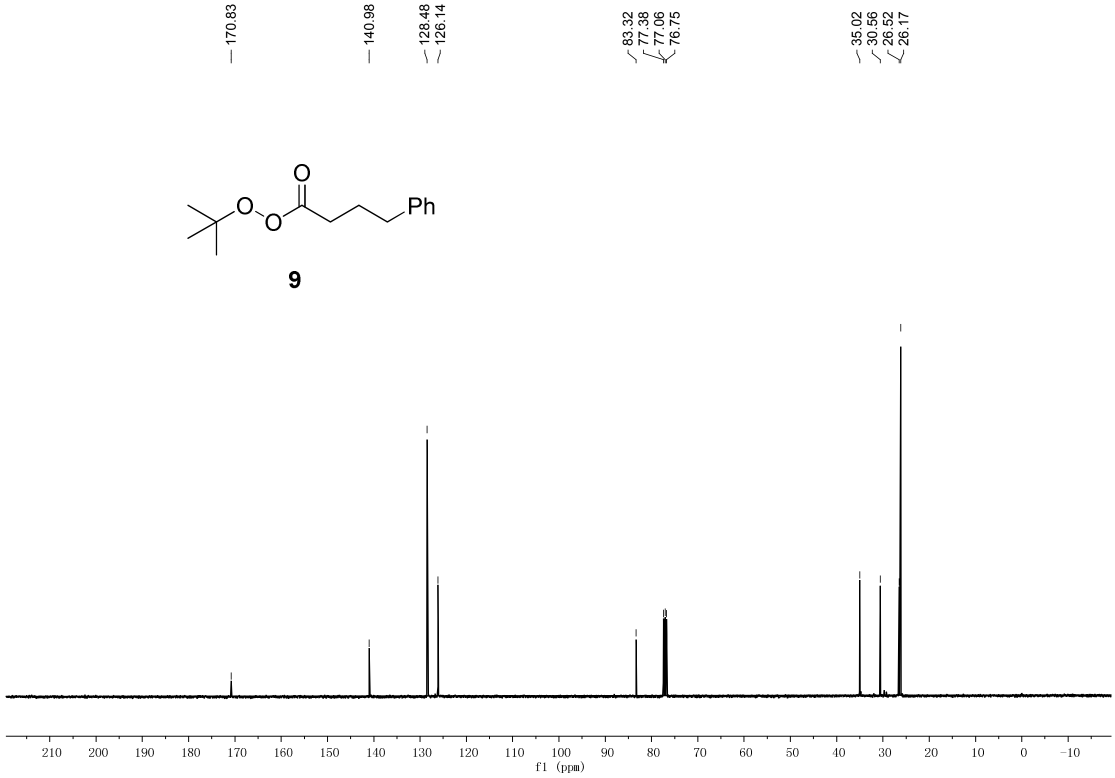
170.83
140.98
128.48 126.14
83.32 77.38 77.06 76.75
35.02 30.56 26.52 26.17
O
O
O
Ph
9
210 200 190 180 170 160 150 140 130 120 110 100 90 80 70 60 50 40 30 20 10 0 -10
f1 (ppm)

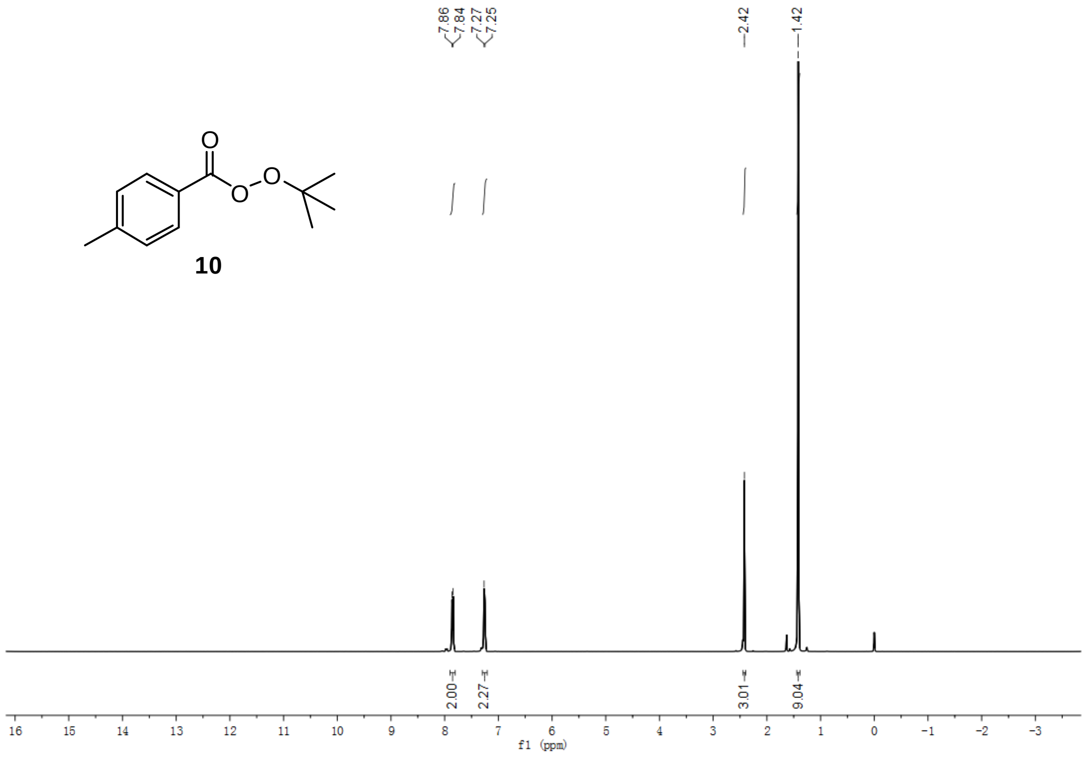
7.86
7.84
7.27
7.25
2.42
1.42
10
2.00
2.27
3.01
9.04
16 15 14 13 12 11 10 9 8 7 6 5 4 3 2 1 0 −1 −2 −3
f1 (ppm)

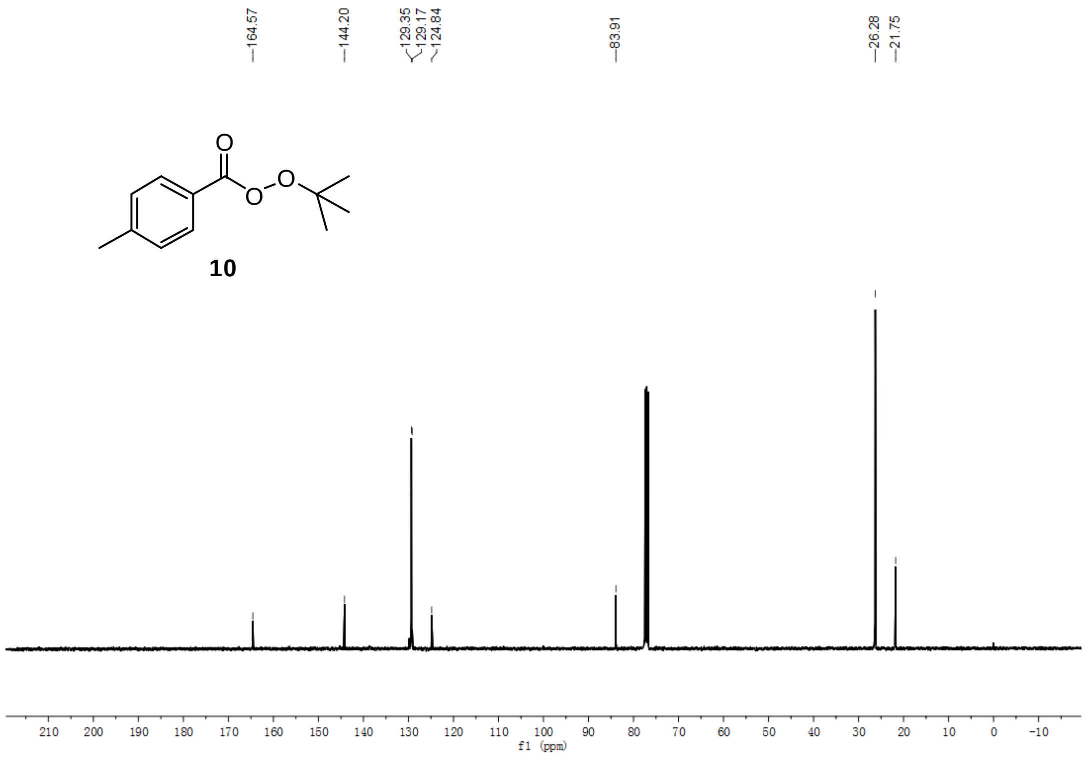
164.57
144.20
129.35
129.17
124.84
83.91
26.28
21.75
10
210 200 190 180 170 160 150 140 130 120 110 100 90 80 70 60 50 40 30 20 10 0 −10
f1 (ppm)

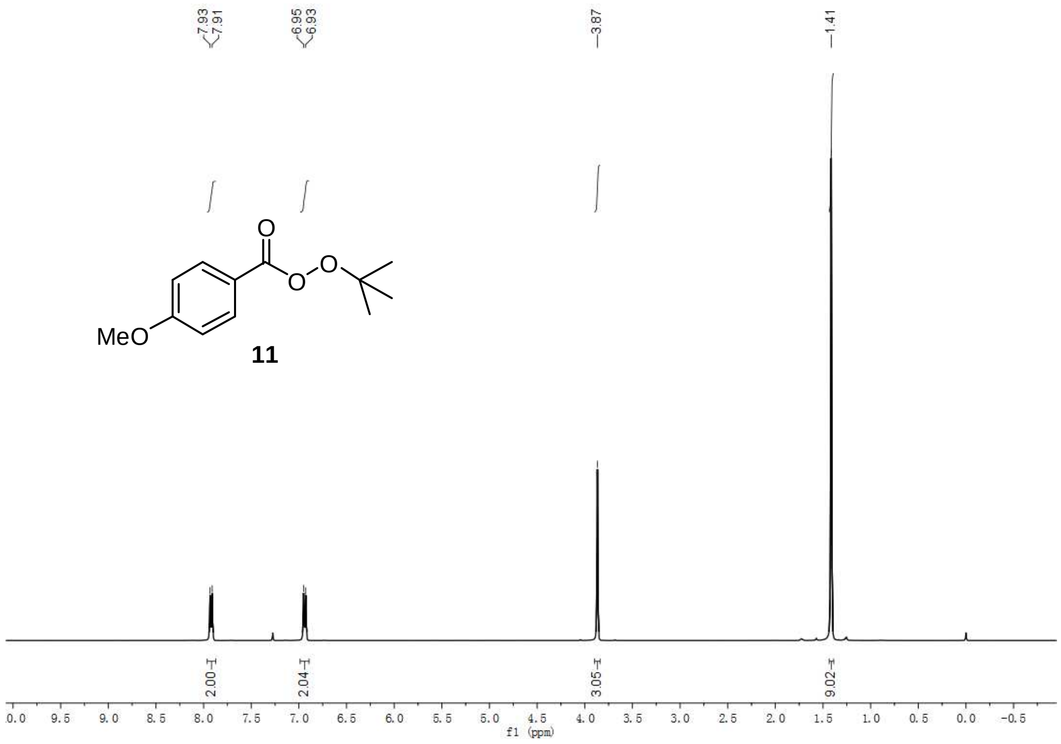
7.93
7.91
6.95
6.93
3.87
1.41
MeO
O
O
O
11
2.00
2.04
3.05
9.02
f1 (ppm)

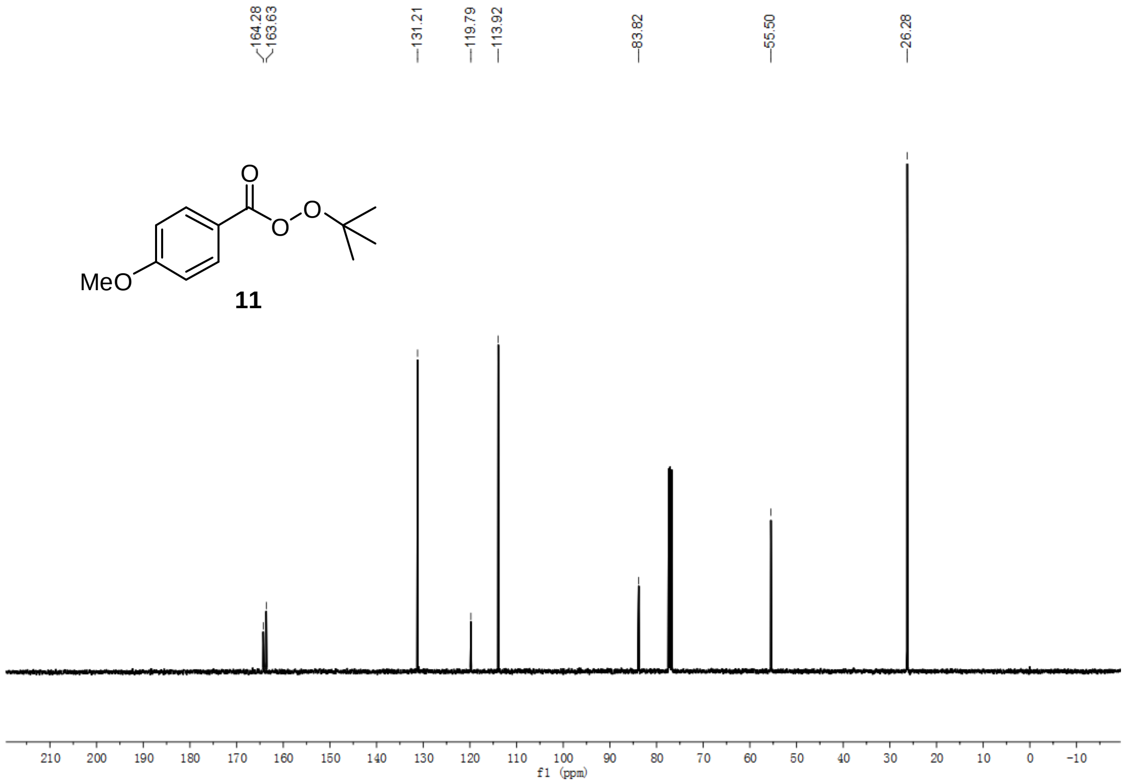
164.28
163.63
131.21
119.79
113.92
83.82
55.50
26.28
MeO
O
O
O
11
f1 (ppm)

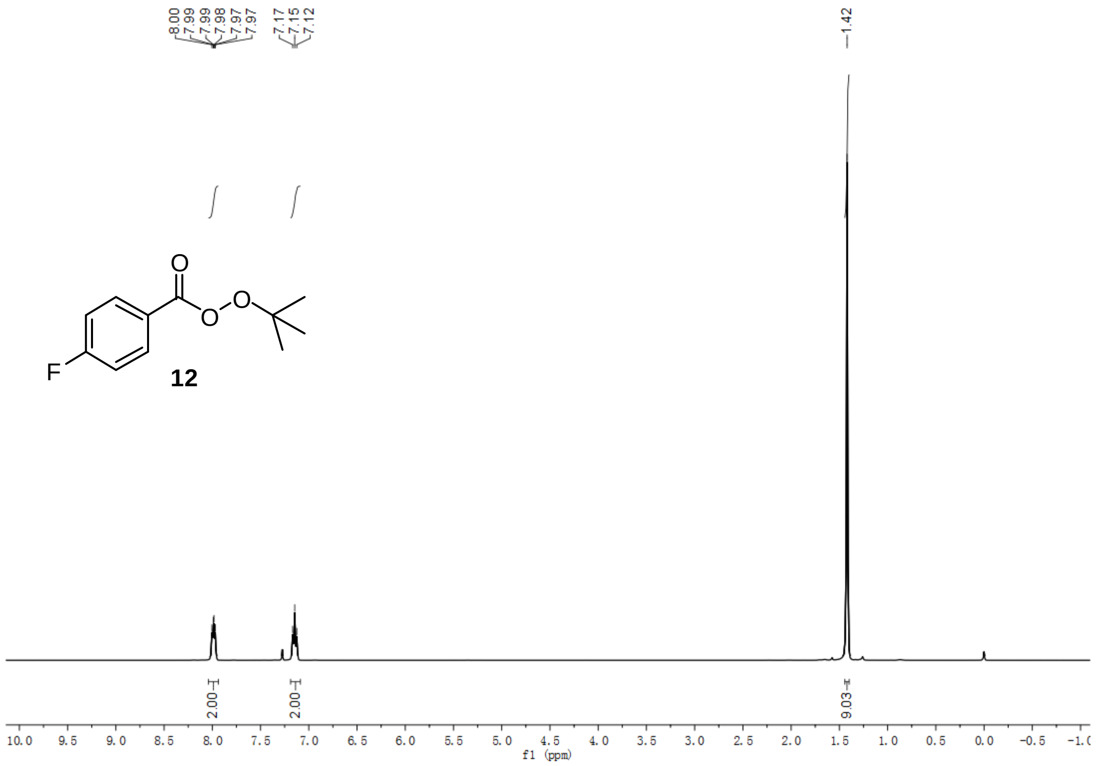
8.00
7.99
7.99
7.98
7.97
7.97
7.17
7.15
7.12
1.42
2.00
2.00
9.03
f1 (ppm)

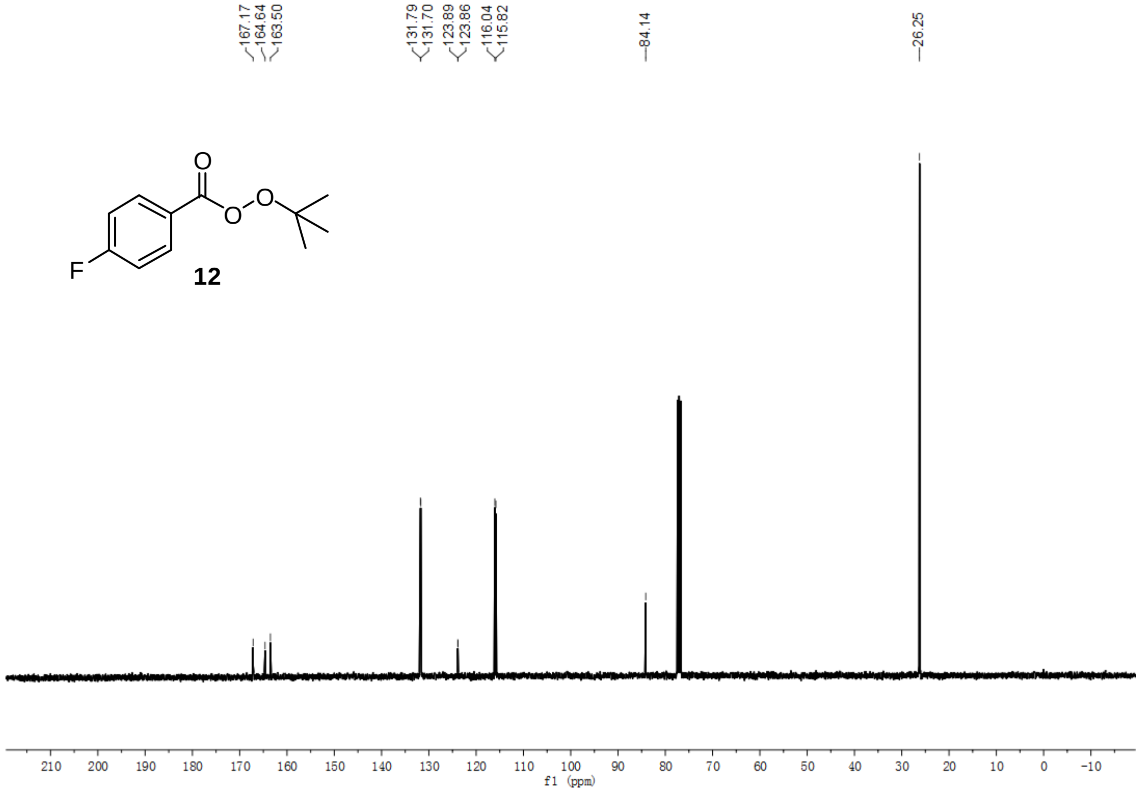
167.17
164.64
163.50
131.79
131.70
123.89
123.86
116.04
115.82
84.14
26.25
f1 (ppm)

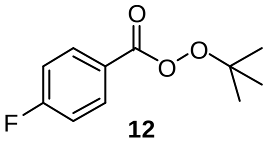
O
O
O
F

12

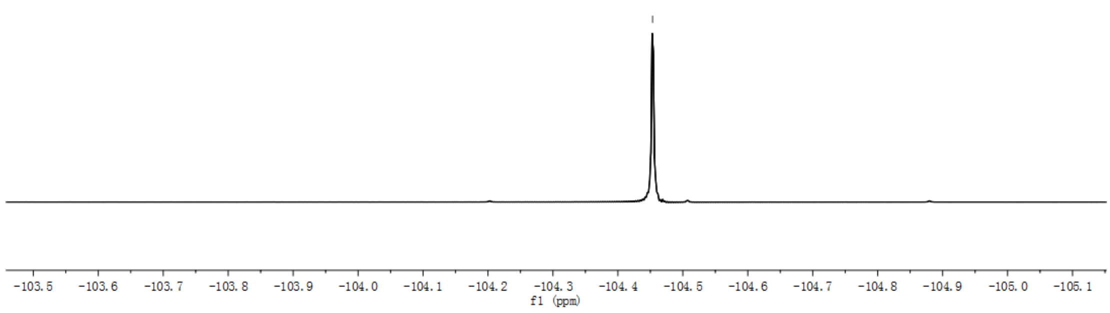
-104.45
-103.5 -103.6 -103.7 -103.8 -103.9 -104.0 -104.1 -104.2 -104.3 -104.4 -104.5 -104.6 -104.7 -104.8 -104.9 -105.0 -105.1
f1 (ppm)

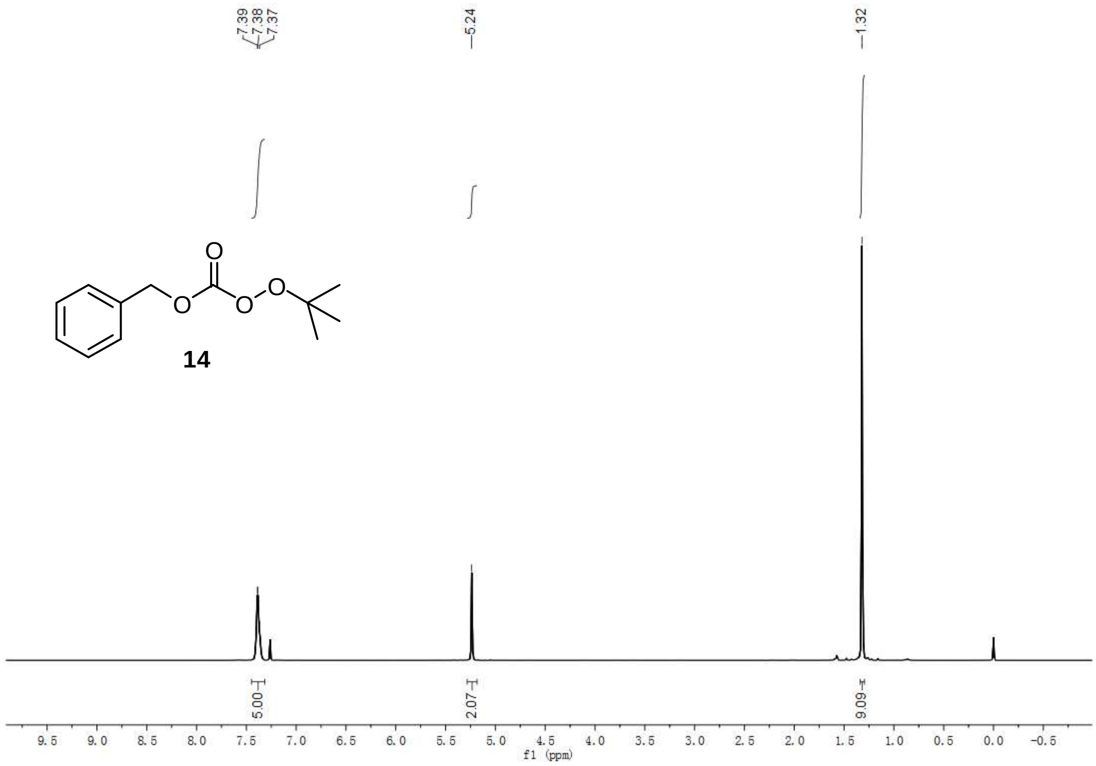
7.39
7.38
7.37
5.24
1.32
14
5.00
2.07
9.09
9.5 9.0 8.5 8.0 7.5 7.0 6.5 6.0 5.5 5.0 4.5 4.0 3.5 3.0 2.5 2.0 1.5 1.0 0.5 0.0 -0.5
f1 (ppm)

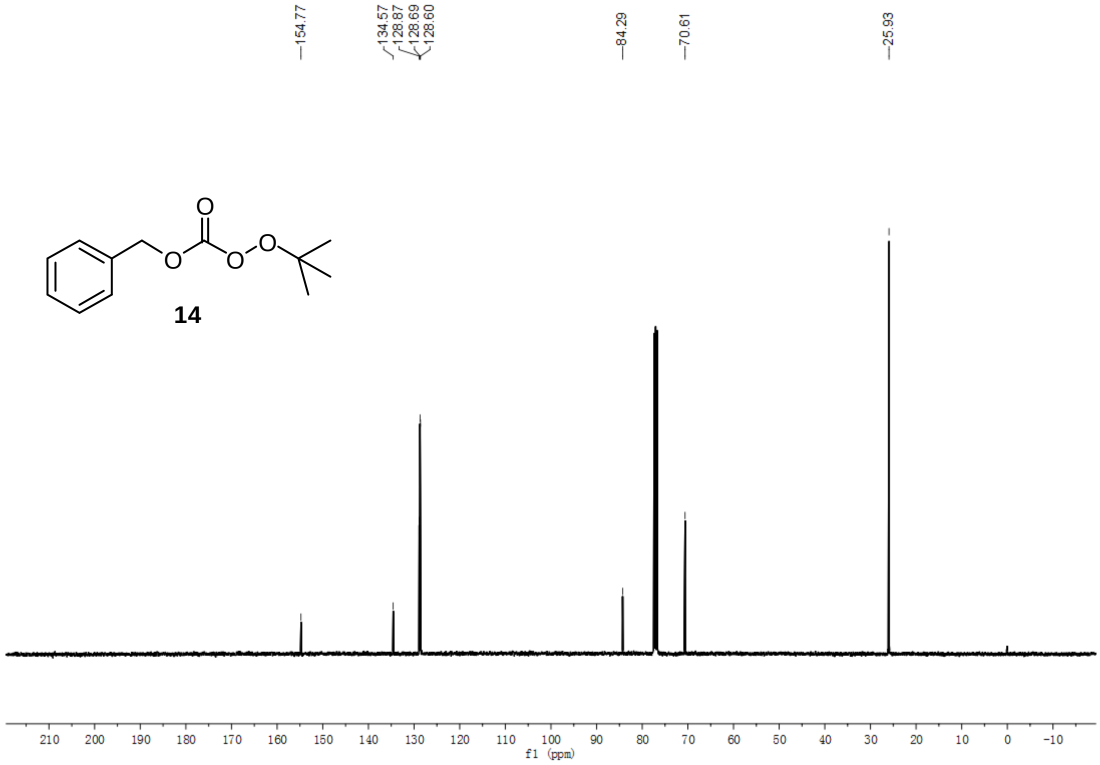
154.77
134.57
128.87
128.69
128.60
84.29
70.61
25.93
14
210 200 190 180 170 160 150 140 130 120 110 100 90 80 70 60 50 40 30 20 10 0 -10
f1 (ppm)

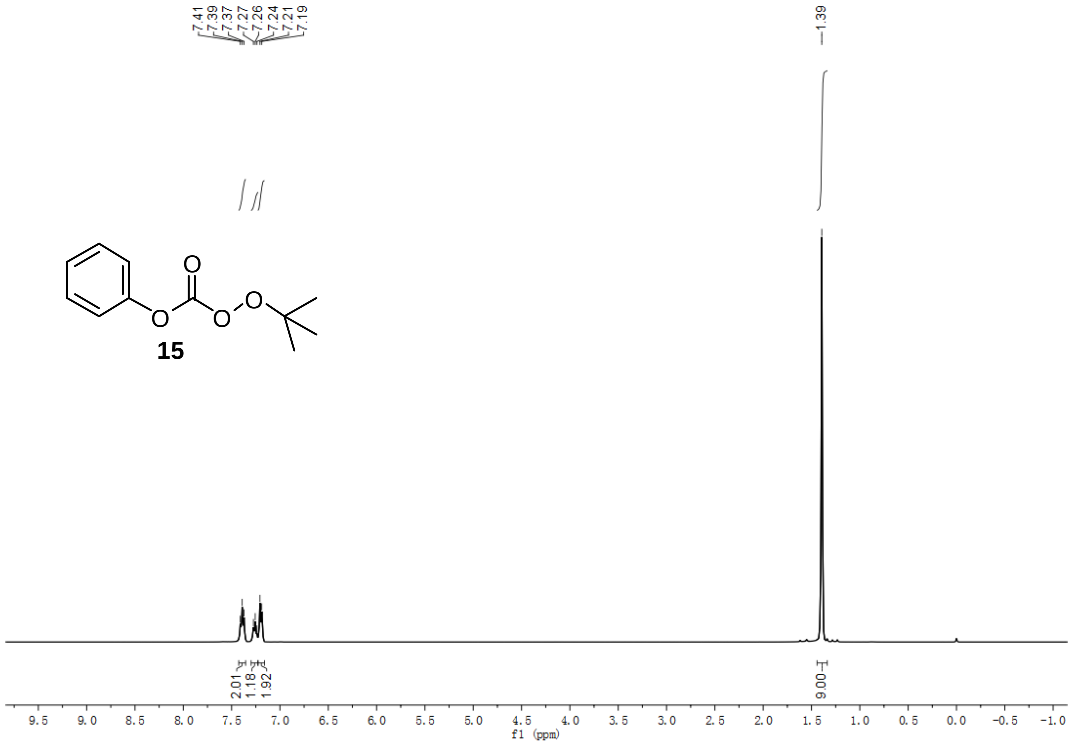
7.41
7.39
7.37
7.27
7.26
7.24
7.21
7.19
1.39
O
O
O
O
O
15
2.01
1.18
1.92
9.00
9.5 9.0 8.5 8.0 7.5 7.0 6.5 6.0 5.5 5.0 4.5 4.0 3.5 3.0 2.5 2.0 1.5 1.0 0.5 0.0 -0.5 -1.0
f1 (ppm)

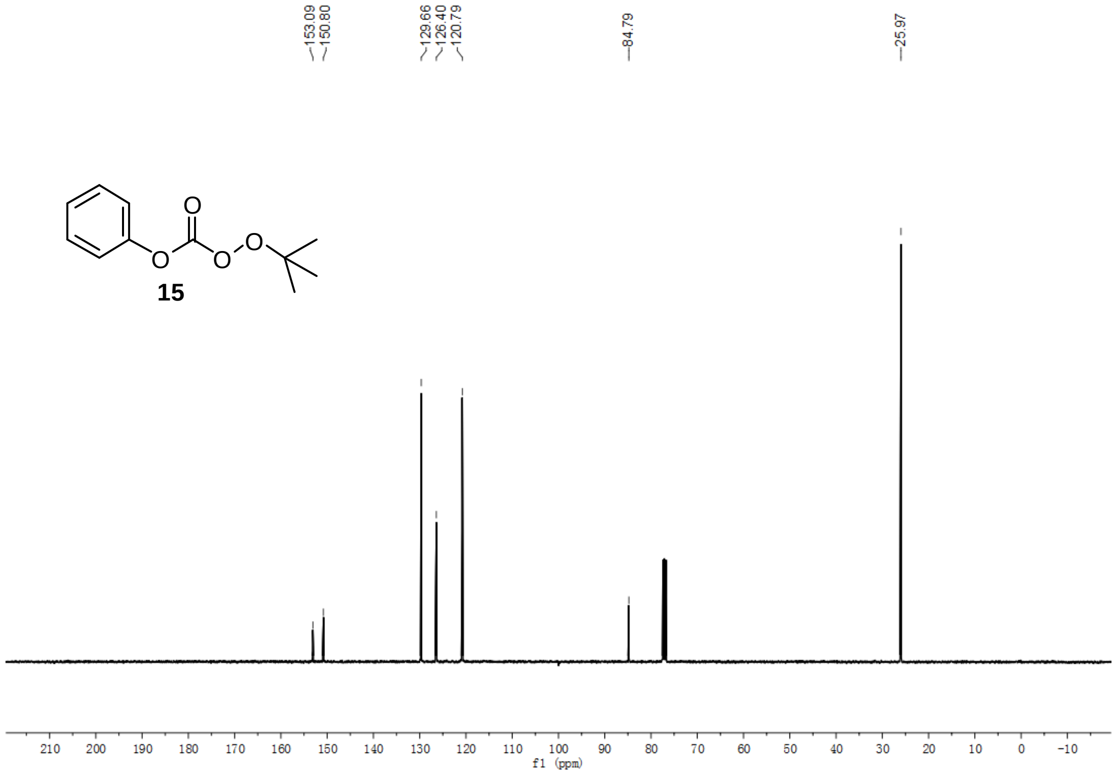
153.09
150.80
129.66
126.40
120.79
84.79
25.97
O
O
O
O
O
15
210 200 190 180 170 160 150 140 130 120 110 100 90 80 70 60 50 40 30 20 10 0 -10
f1 (ppm)

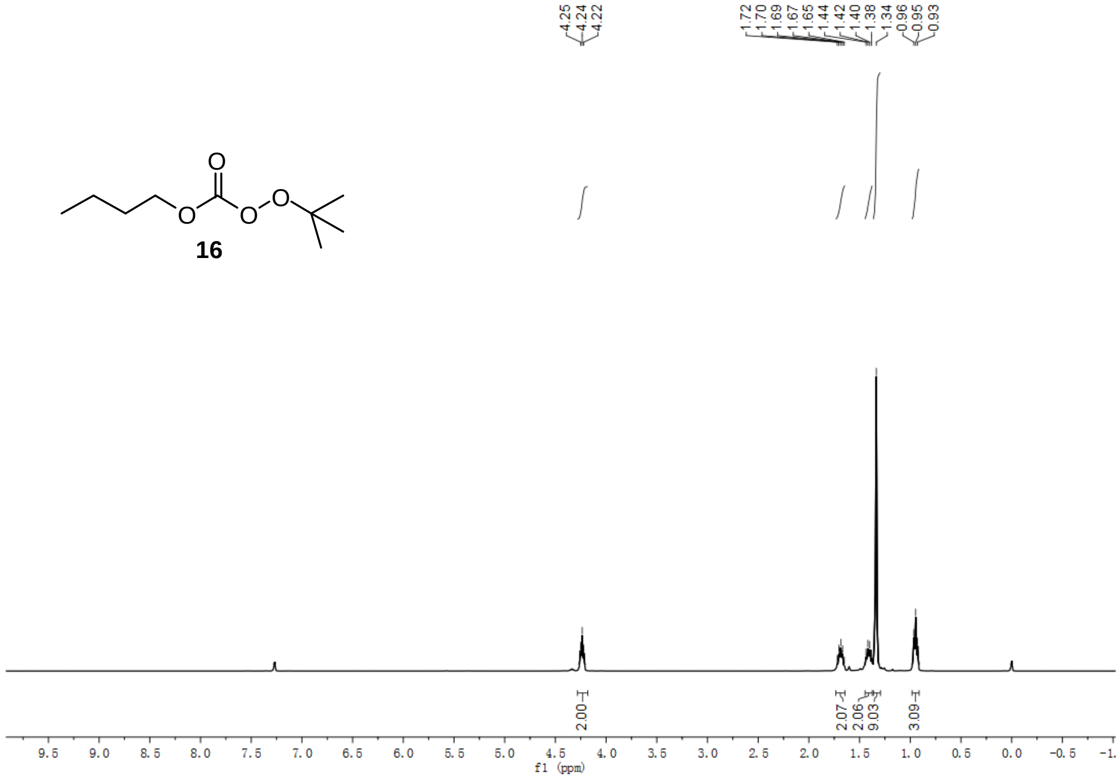
16
4.25
4.24
4.22
1.72
1.70
1.69
1.67
1.65
1.44
1.42
1.40
1.38
1.34
0.96
0.95
0.93
2.00
2.07
2.06
9.03
3.09
f1 (ppm)

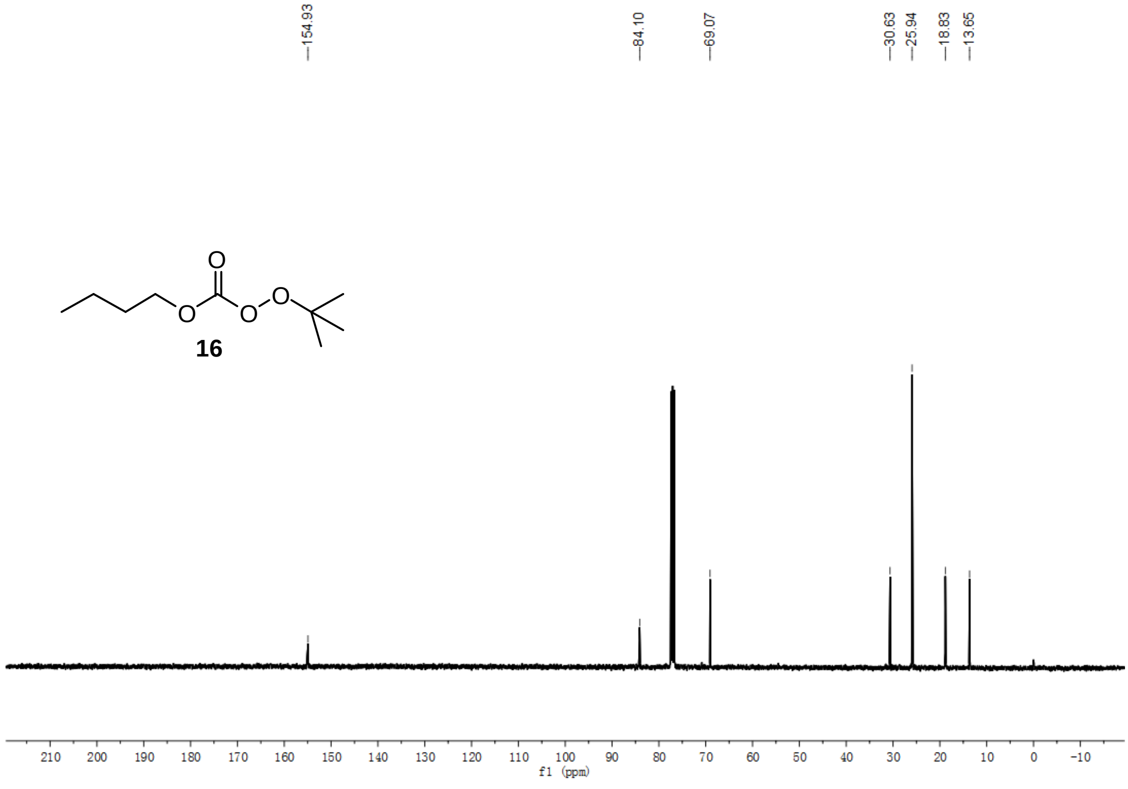
16
154.93
84.10
69.07
30.63
25.94
18.83
13.65
f1 (ppm)

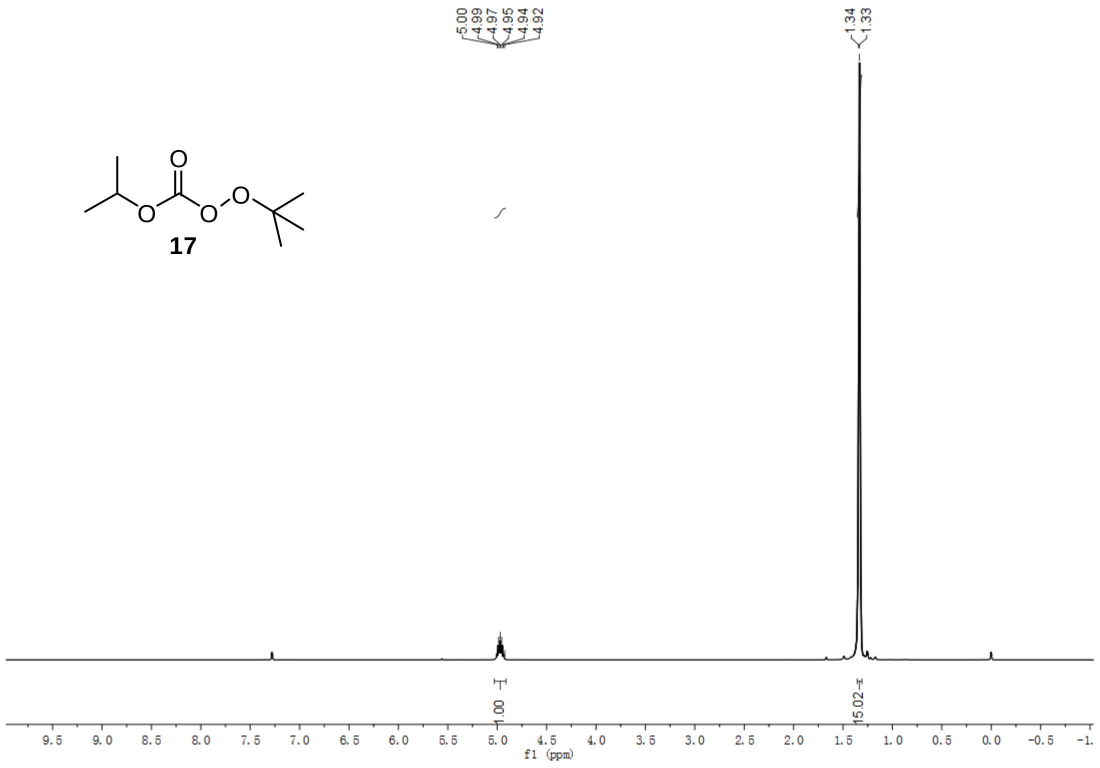

5.00
4.99
4.97
4.95
4.94
4.92
1.34
1.33
17
1.00
15.02
9.5 9.0 8.5 8.0 7.5 7.0 6.5 6.0 5.5 5.0 4.5 4.0 3.5 3.0 2.5 2.0 1.5 1.0 0.5 0.0 −0.5 −1.
f1 (ppm)

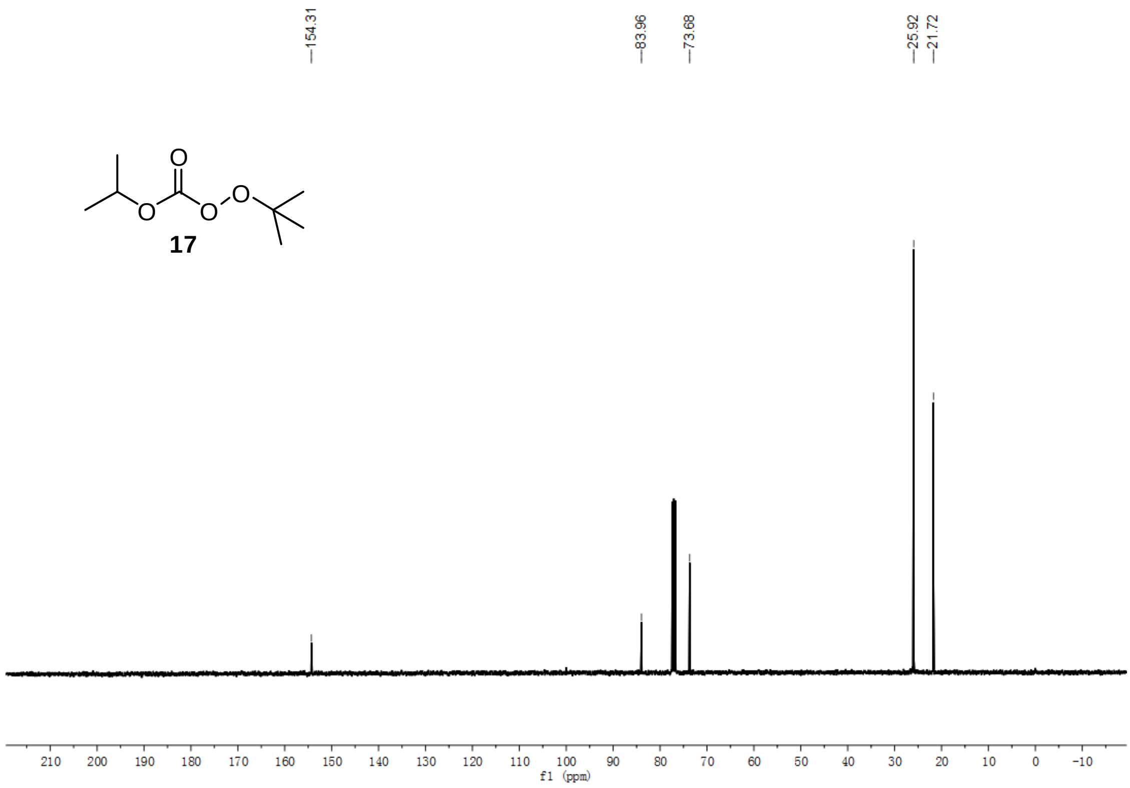

154.31
83.96
73.68
25.92
21.72
17
210 200 190 180 170 160 150 140 130 120 110 100 90 80 70 60 50 40 30 20 10 0 −10
f1 (ppm)

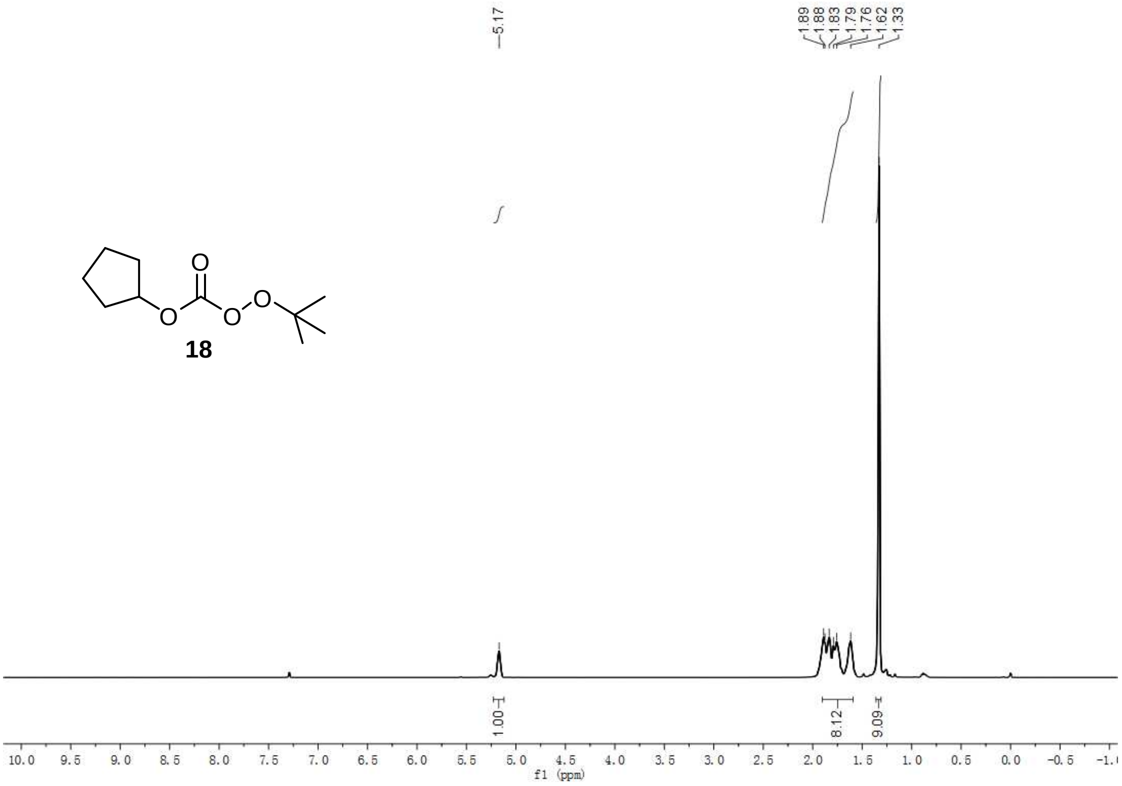

18
5.17
1.89
1.88
1.83
1.79
1.76
1.62
1.33
1.00
8.12
9.09
f1 (ppm)

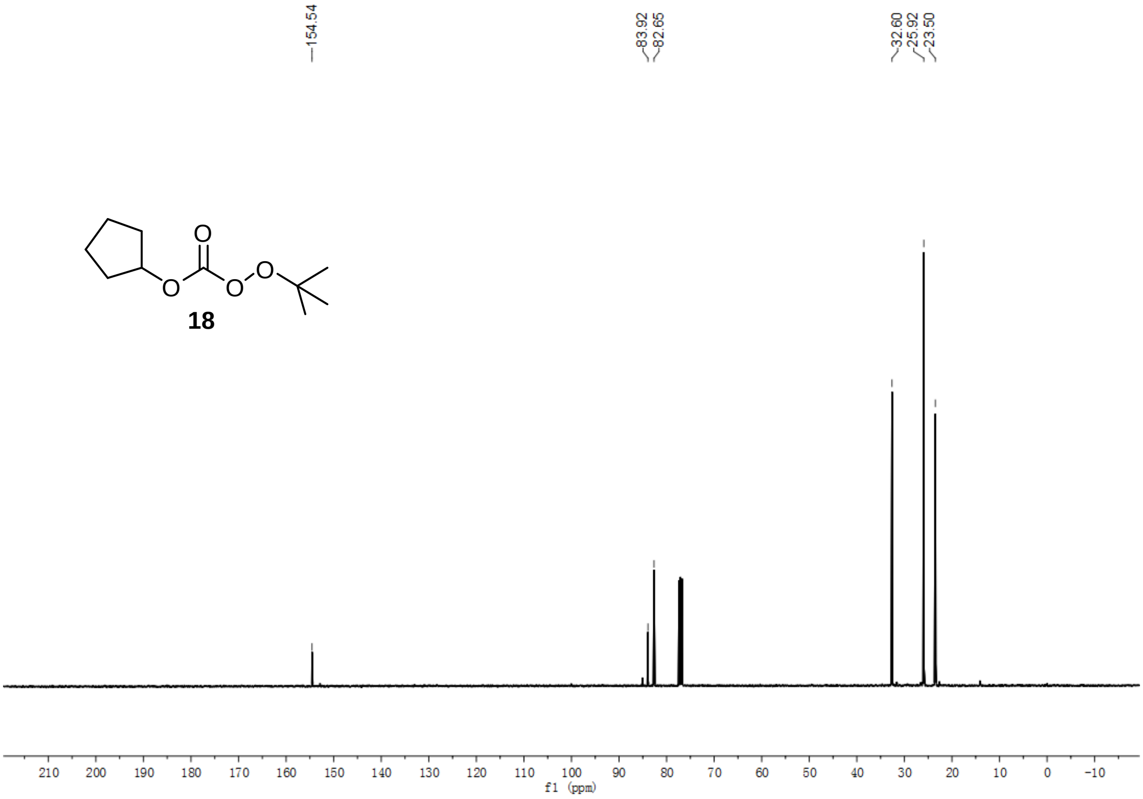

18
154.54
83.92
82.65
32.60
25.92
23.50
f1 (ppm)

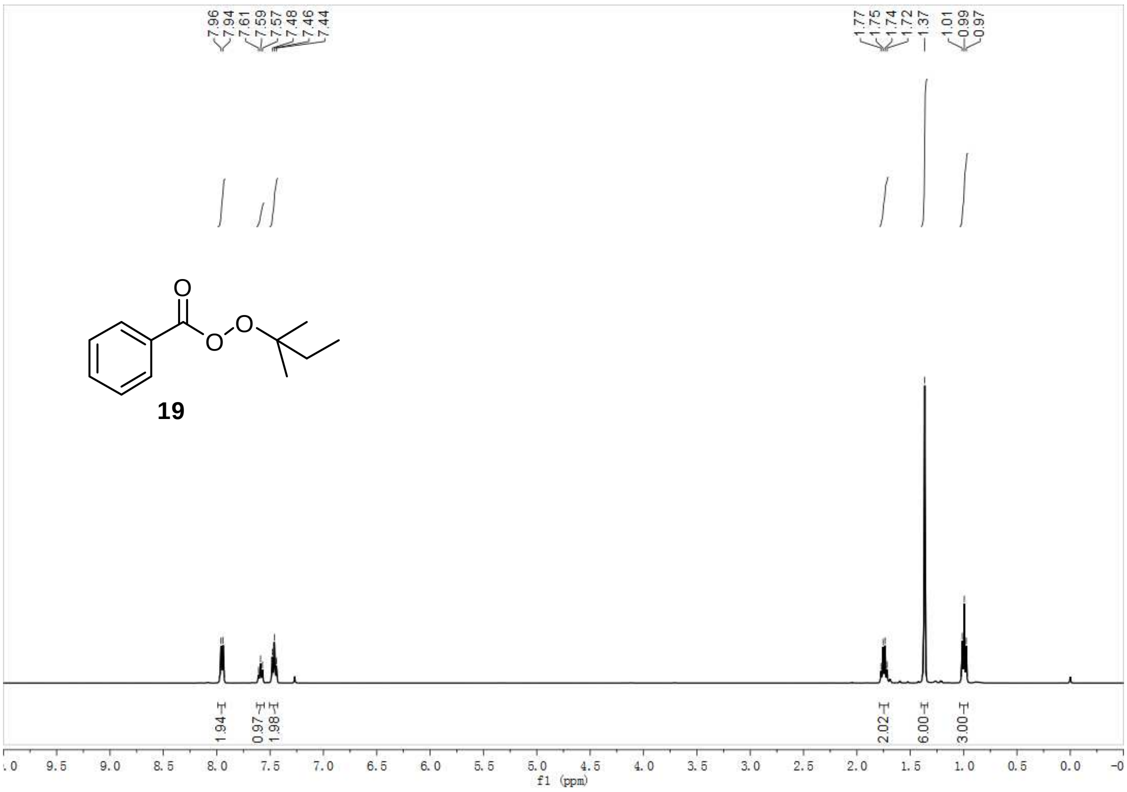
7.96
7.94
7.61
7.59
7.57
7.48
7.46
7.44
1.77
1.75
1.74
1.72
1.37
1.01
0.99
0.97
1.94
0.97
1.98
2.02
6.00
3.00
f1 (ppm)

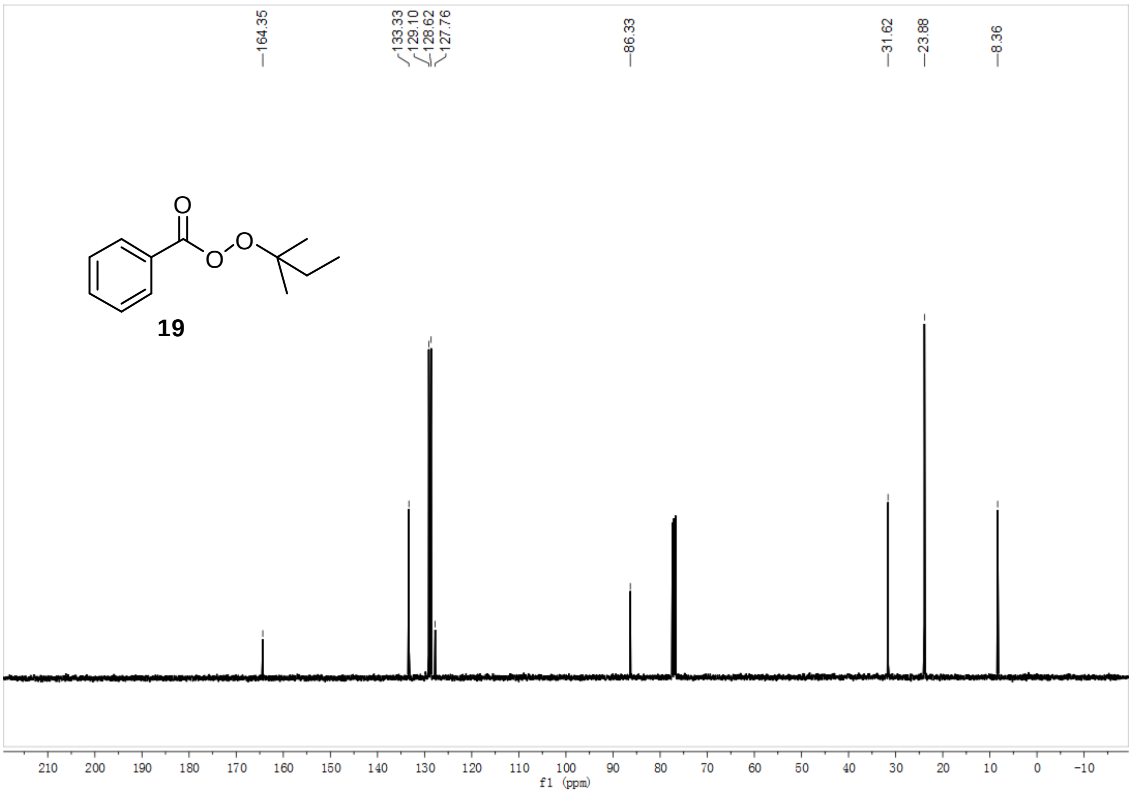
164.35
133.33
129.10
128.62
127.76
86.33
31.62
23.88
8.36
f1 (ppm)

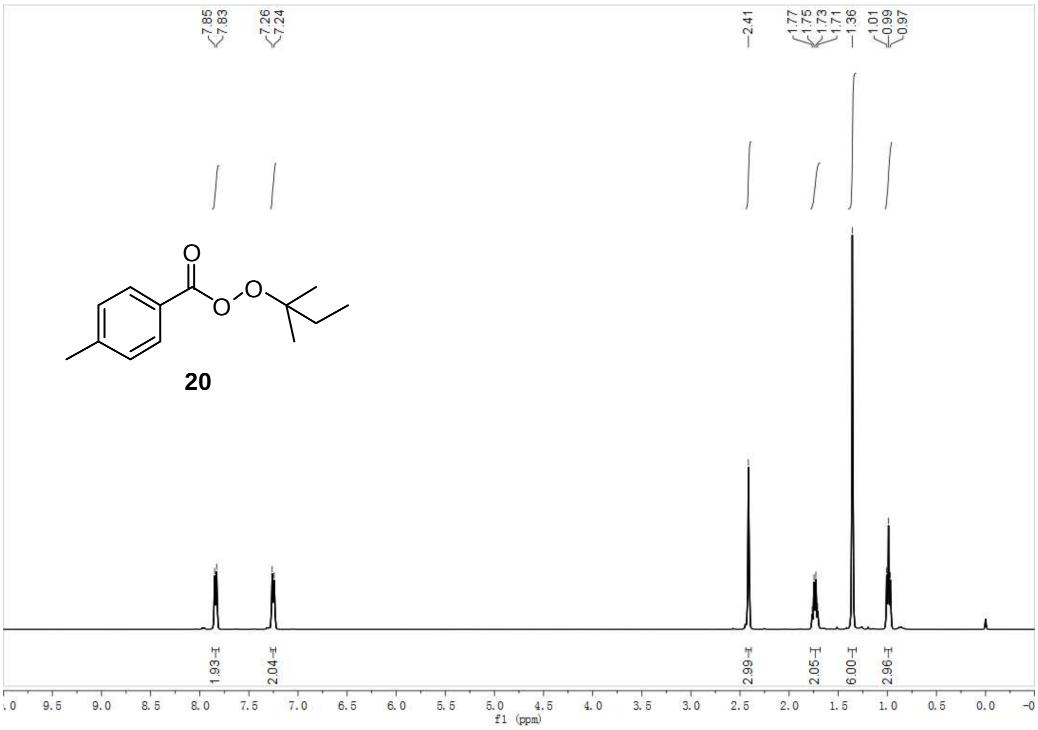
7.85
7.83
7.26
7.24
2.41
1.77
1.75
1.73
1.71
1.36
1.01
0.99
0.97
O
O
O
1.93
2.04
2.99
2.05
6.00
2.96
f1 (ppm)

20

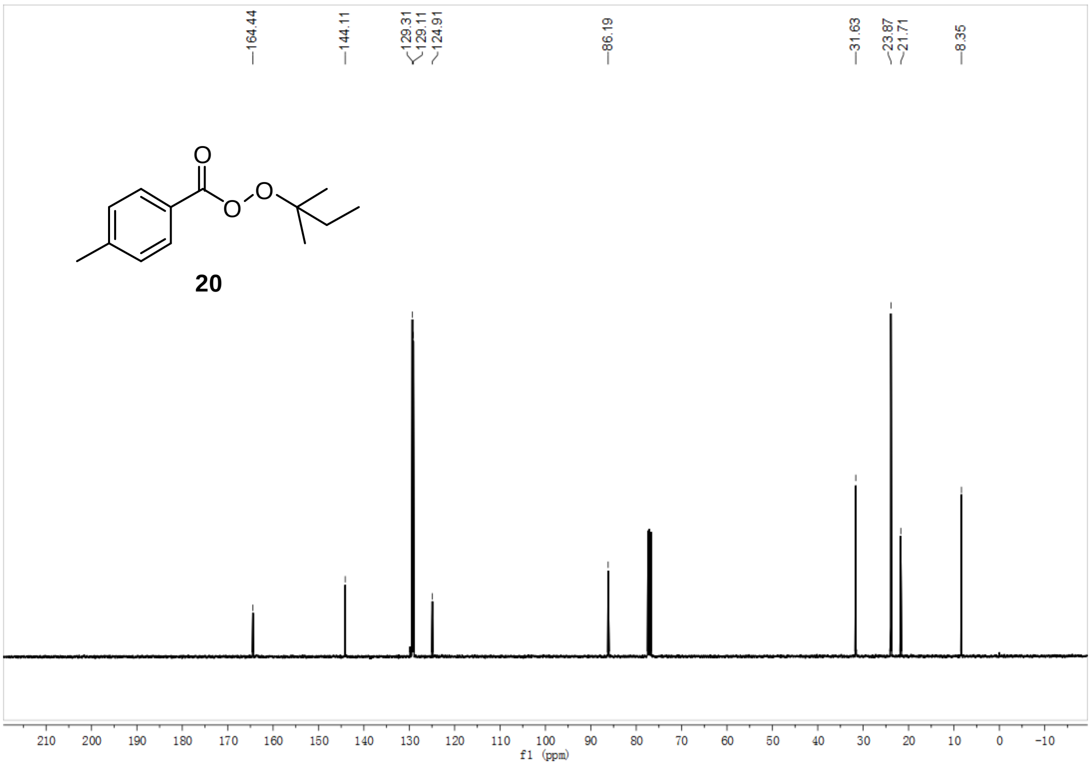
164.44
144.11
129.31
129.11
124.91
86.19
31.63
23.87
21.71
8.35
O
O
O
f1 (ppm)

20

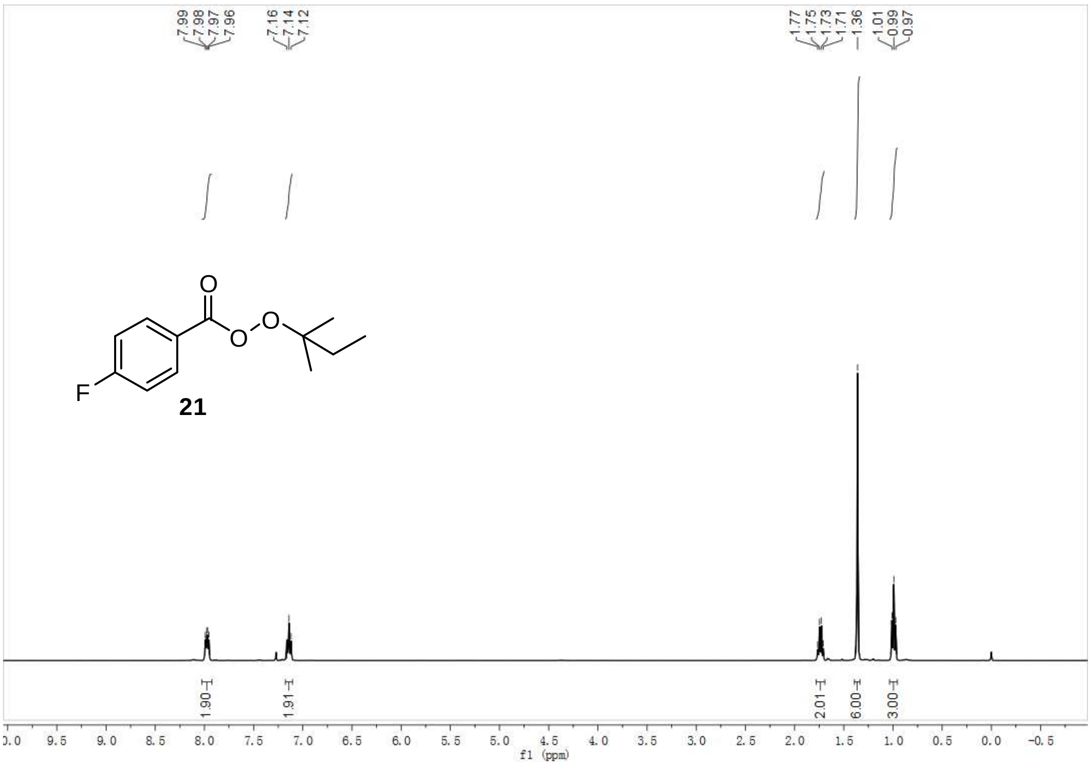
7.99
7.98
7.97
7.96
7.16
7.14
7.12
1.77
1.75
1.73
1.71
1.36
1.01
0.99
0.97
O
O
O
F
21
1.90
1.91
2.01
6.00
3.00
f1 (ppm)

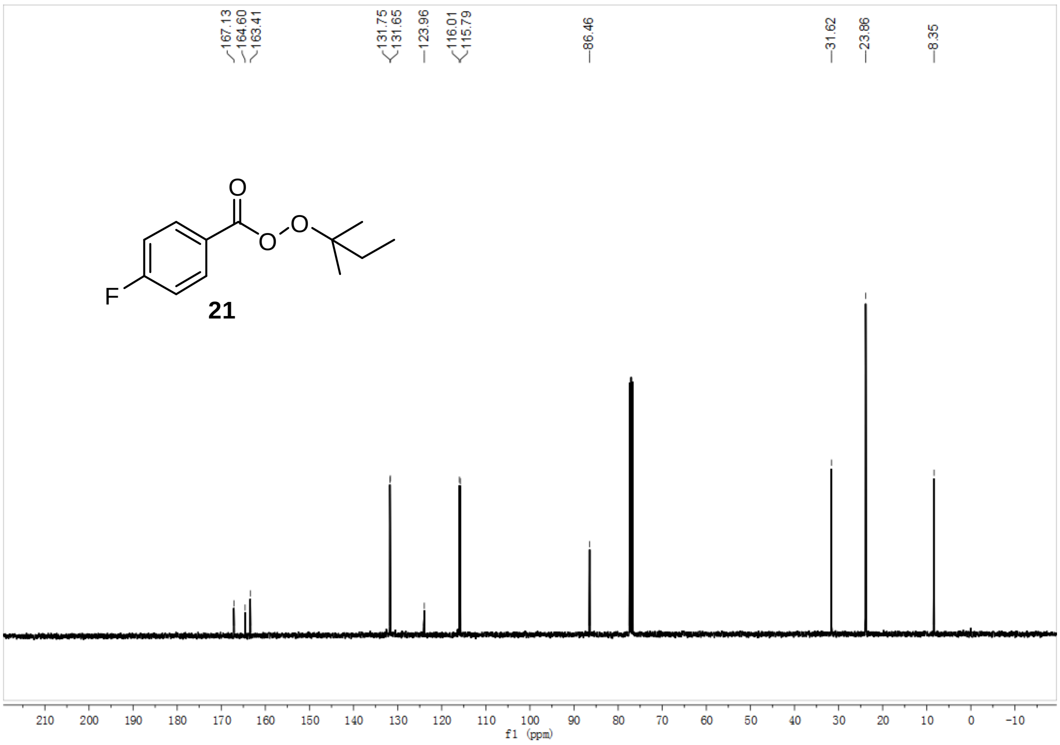
167.13
164.60
163.41
131.75
131.65
123.96
116.01
115.79
86.46
31.62
23.86
8.35
O
O
O
F
21
f1 (ppm)

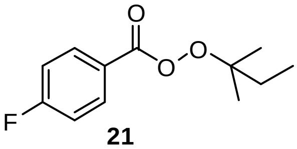
O
O
O
F

21

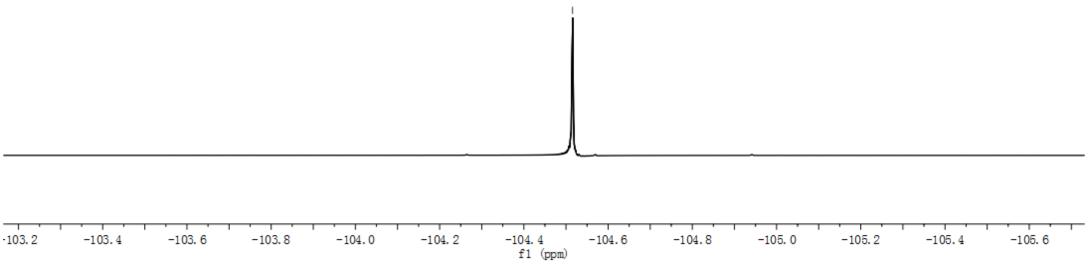
-104.52
-103.2
-103.4
-103.6
-103.8
-104.0
-104.2
-104.4
-104.6
-104.8
-105.0
-105.2
-105.4
-105.6
f1 (ppm)

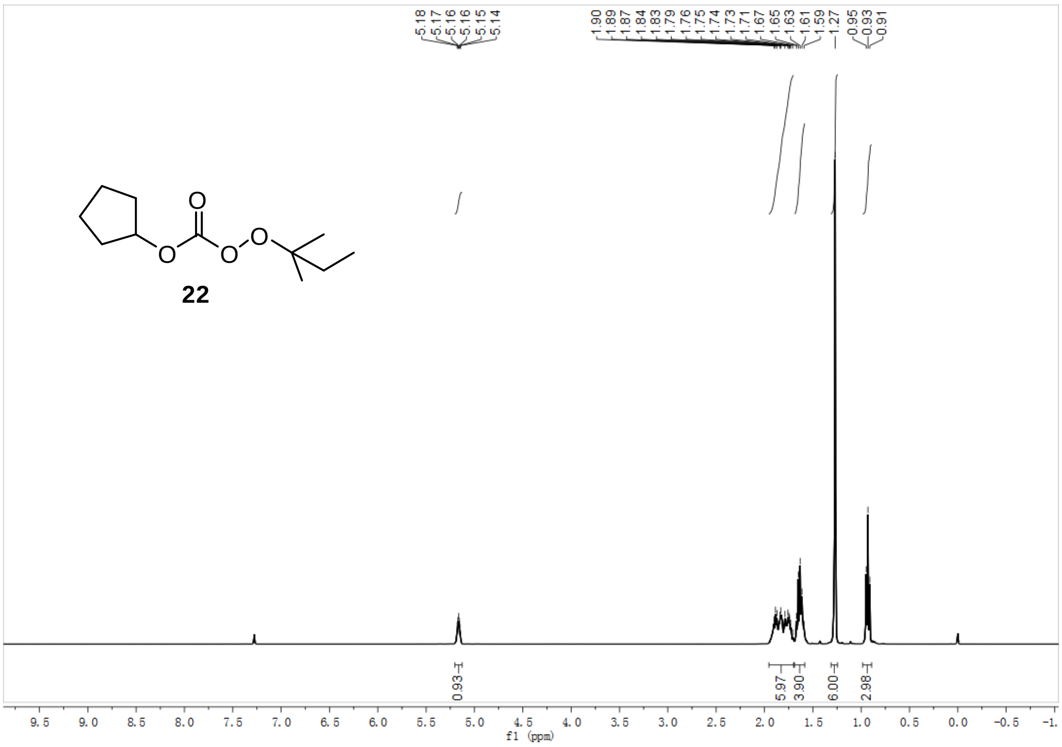

22

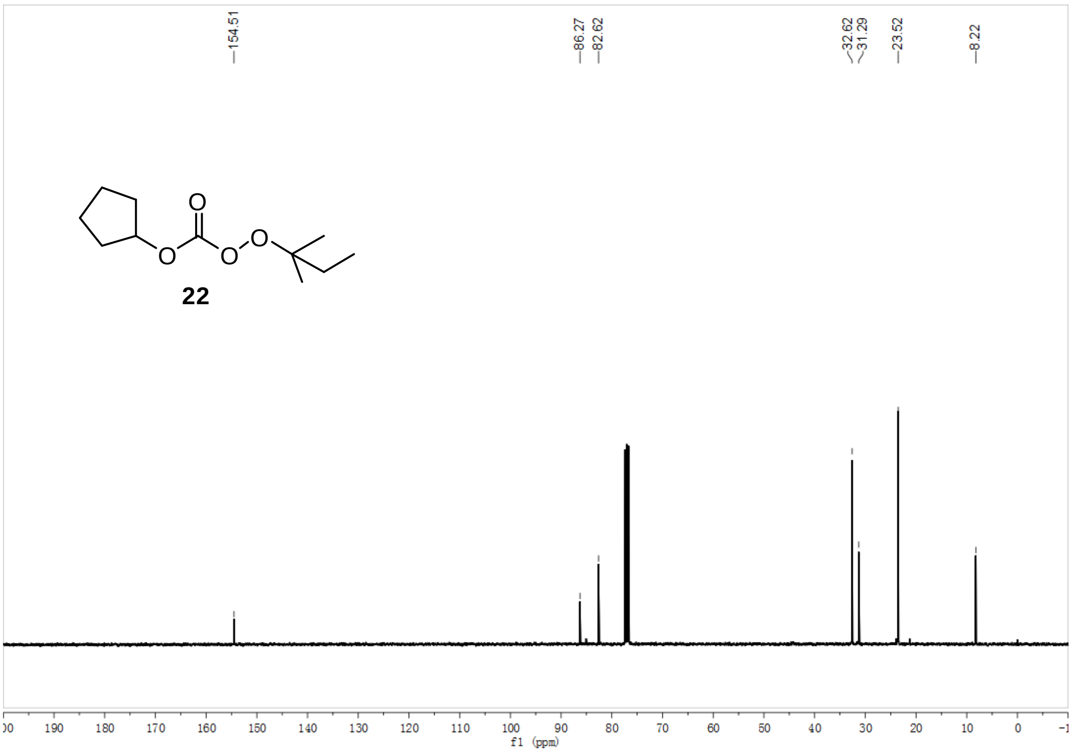

22

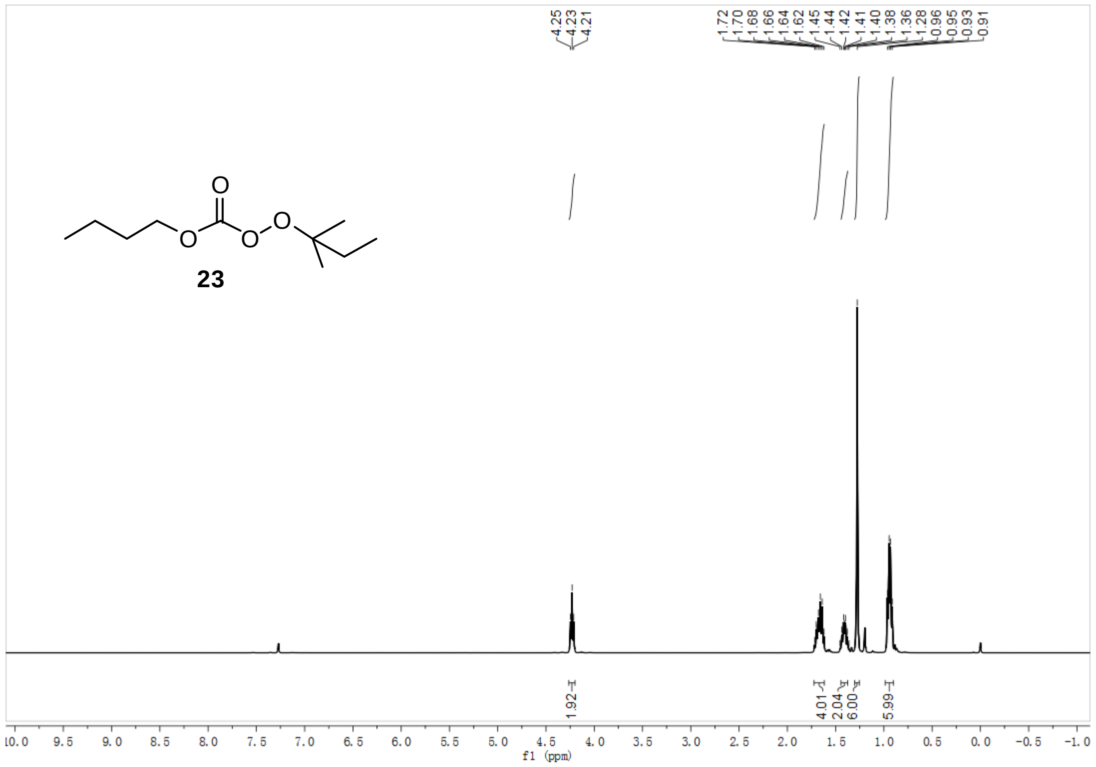
4.25
4.23
4.21
1.72
1.70
1.68
1.66
1.64
1.62
1.45
1.44
1.42
1.41
1.40
1.38
1.36
1.28
0.96
0.95
0.93
0.91
23
1.92
4.01
2.04
6.00
5.99
f1 (ppm)

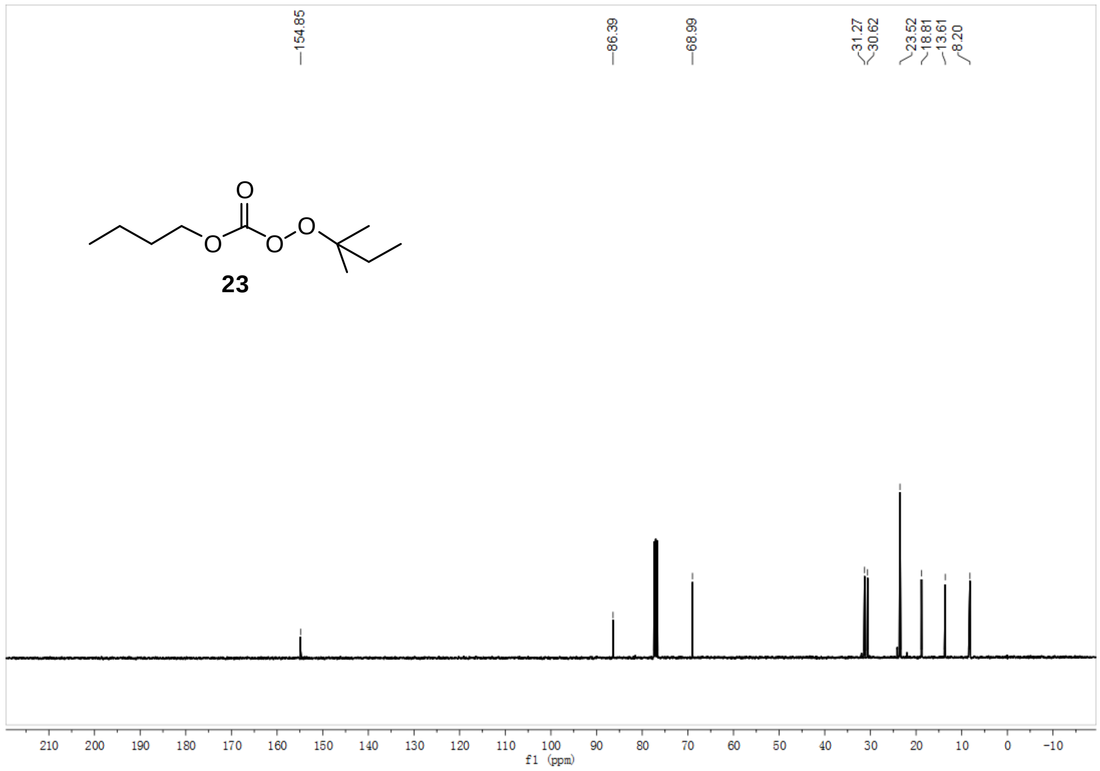
154.85
86.39
68.99
31.27
30.62
23.52
18.81
13.61
8.20
23
f1 (ppm)

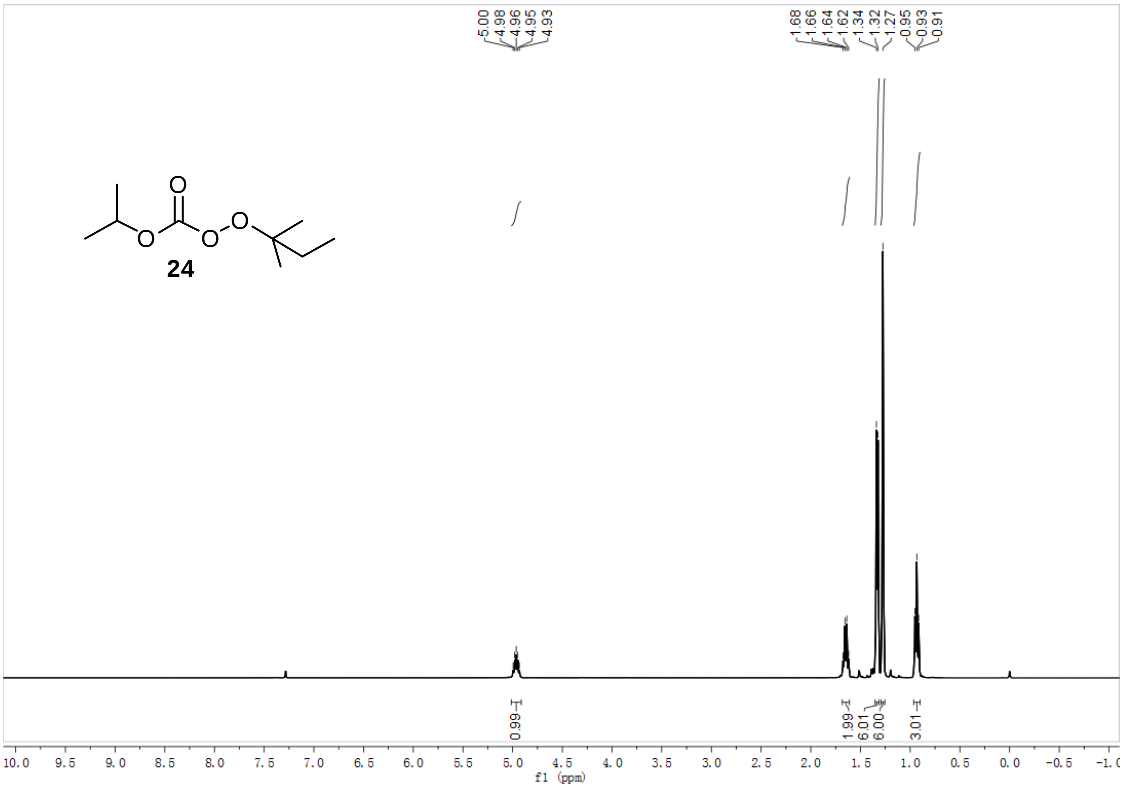
24
f1 (ppm)

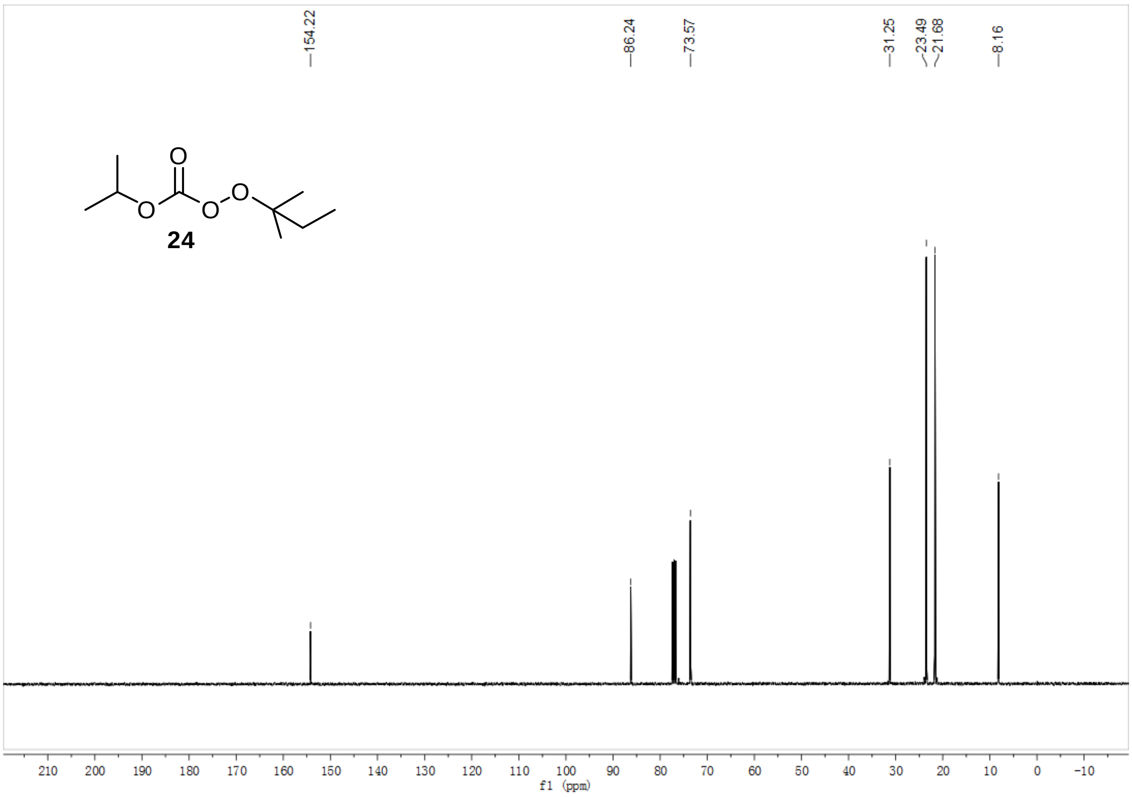
24
f1 (ppm)

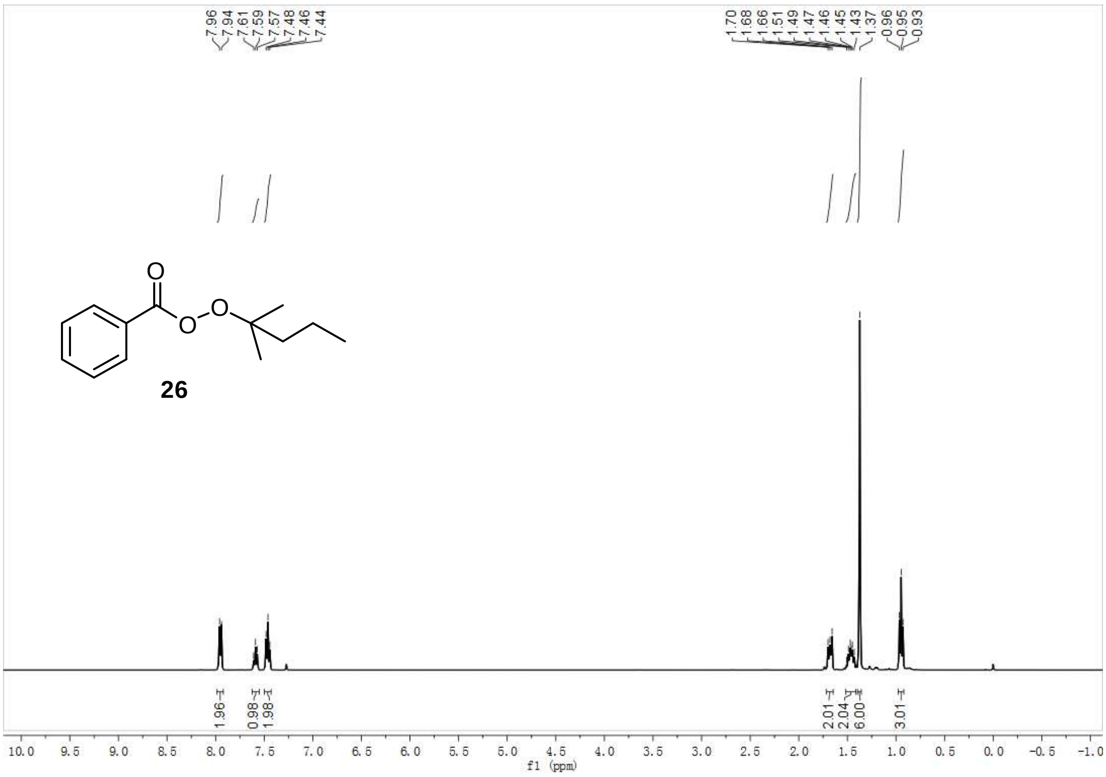
7.96
7.94
7.61
7.59
7.57
7.48
7.46
7.44
1.70
1.68
1.66
1.51
1.49
1.47
1.46
1.45
1.43
1.37
0.96
0.95
0.93
26
1.96
0.98
1.98
2.01
2.04
6.00
3.01
f1 (ppm)

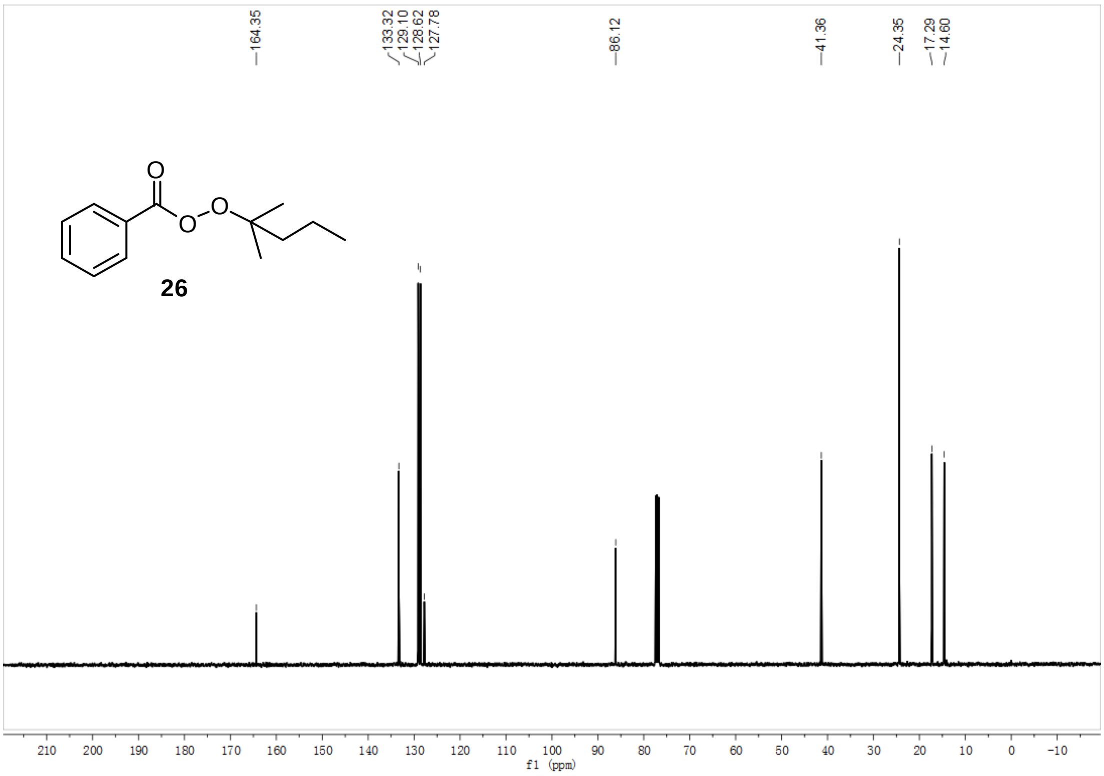
164.35
133.32
129.10
128.62
127.78
86.12
41.36
24.35
17.29
14.60
26
f1 (ppm)

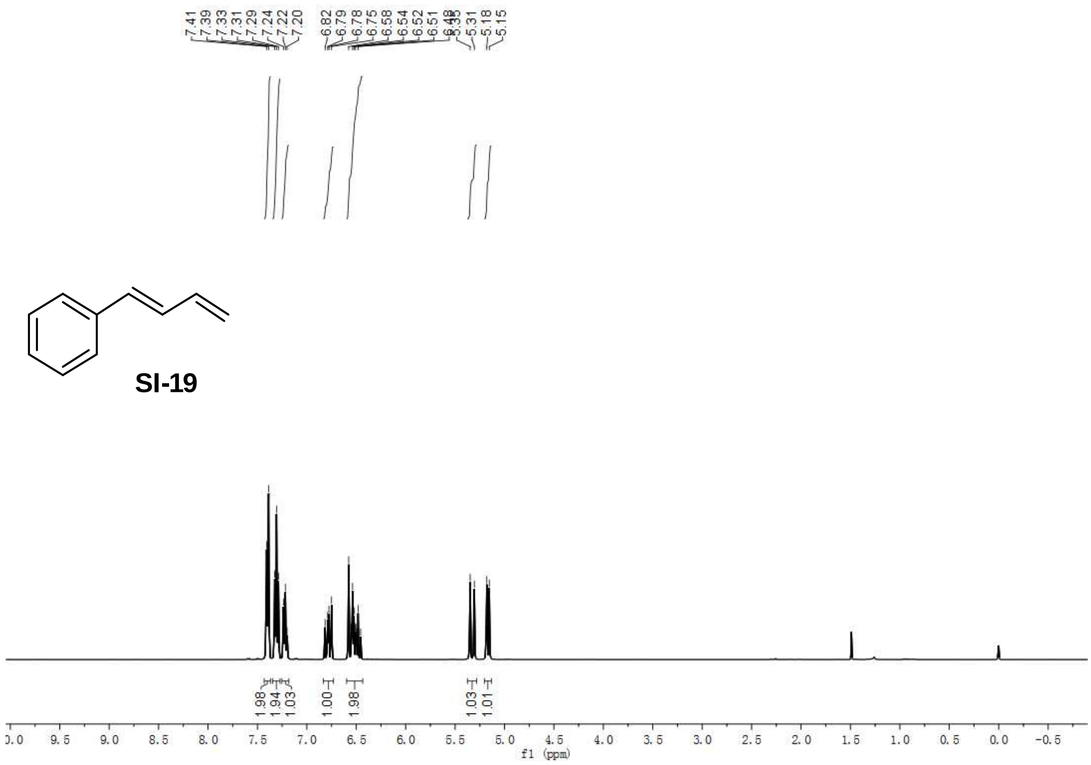
7.41
7.39
7.33
7.31
7.29
7.24
7.22
7.20
6.82
6.79
6.78
6.75
6.58
6.54
6.52
6.51
5.31
5.18
5.15
1.98
1.94
1.03
1.00
1.98
1.03
1.01
f1 (ppm)

SI-19

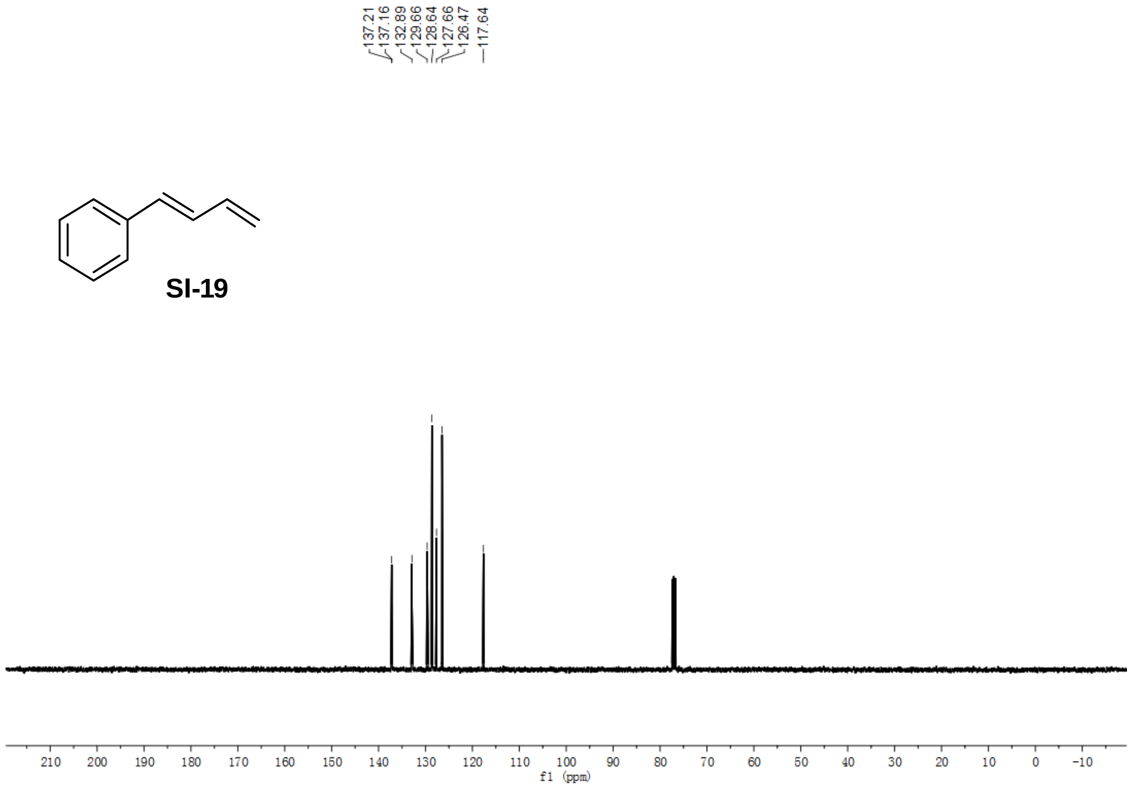
137.21
137.16
132.89
129.66
128.64
127.66
126.47
117.64
f1 (ppm)

SI-19

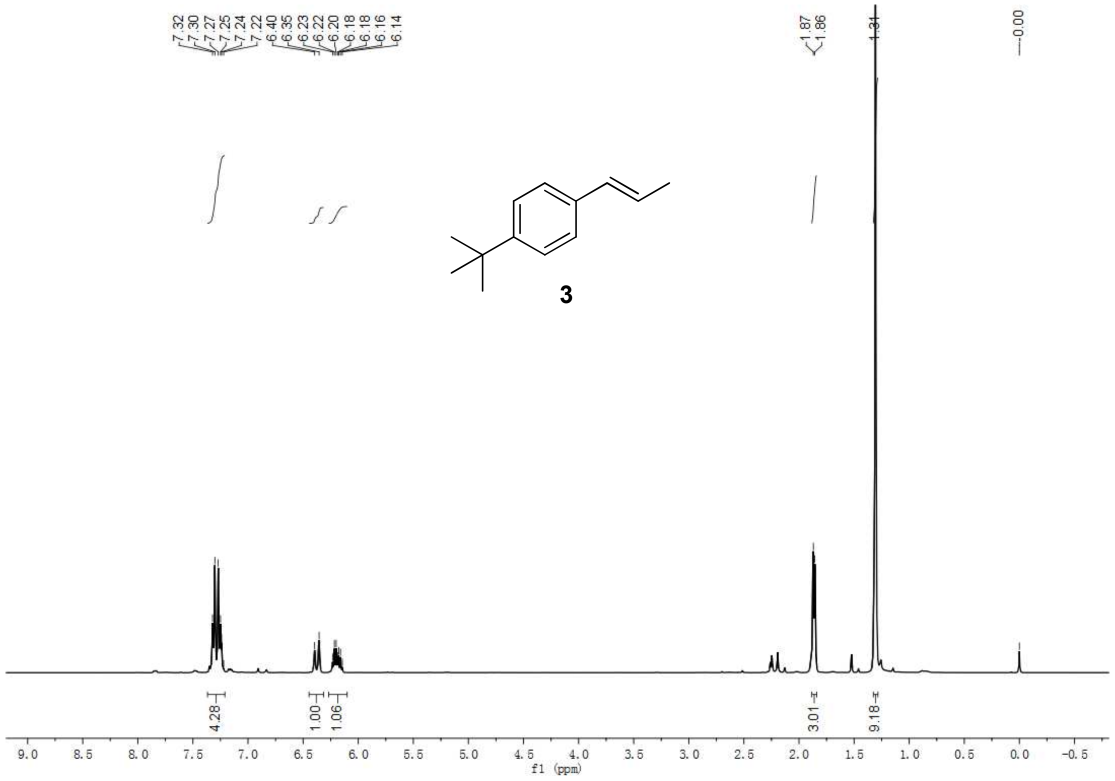
7.32
7.30
7.27
7.25
7.24
7.22
6.40
6.35
6.23
6.22
6.20
6.18
6.18
6.16
6.14
1.87
1.86
1.31
0.00
3
4.28
1.00
1.06
3.01
9.18
f1 (ppm)

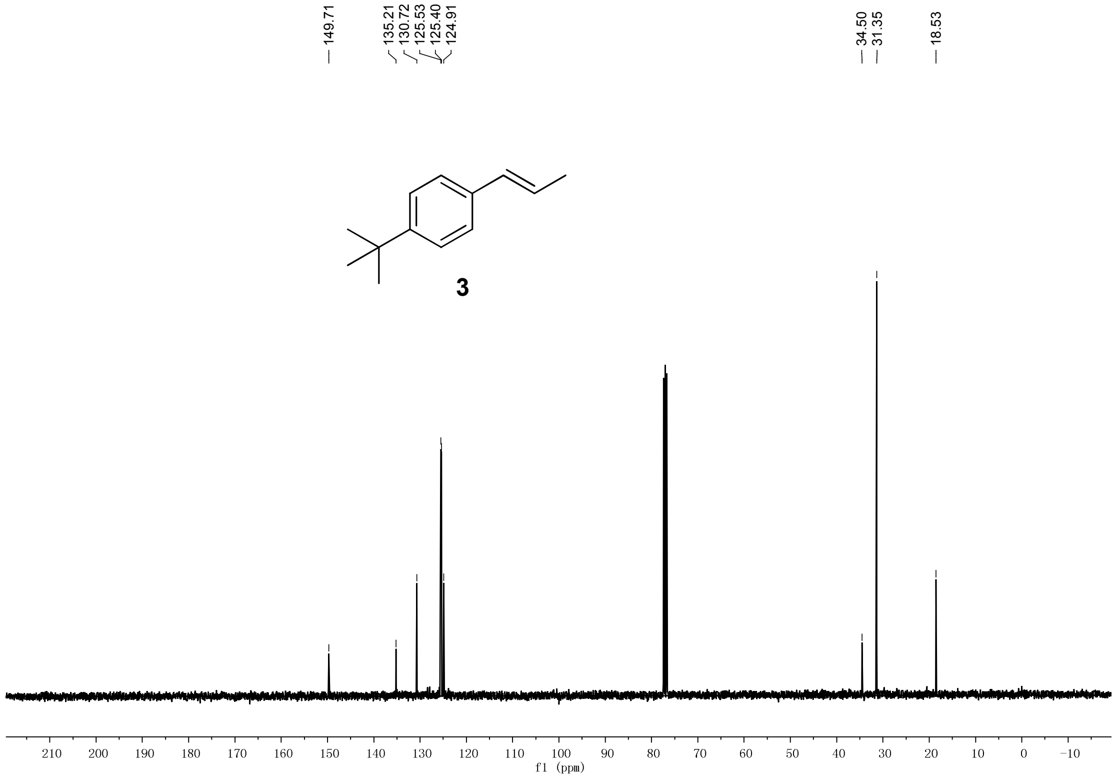
149.71
135.21
130.72
125.53
125.40
124.91
34.50
31.35
18.53
3
f1 (ppm)

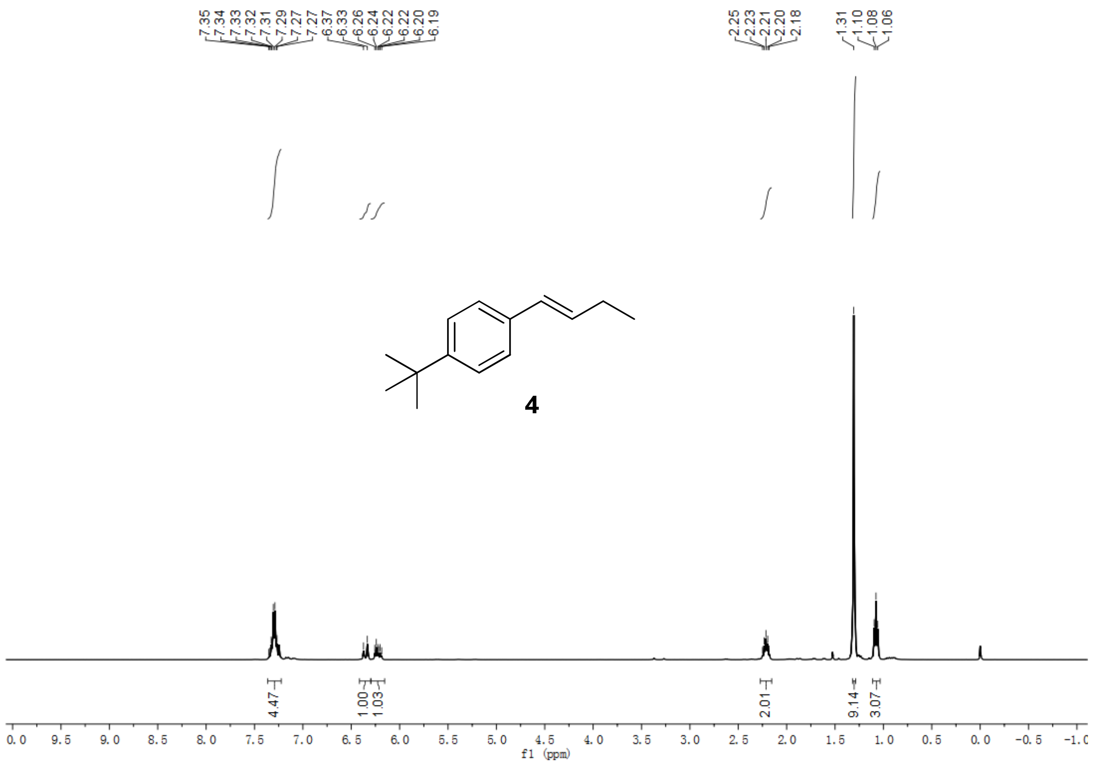
7.35
7.34
7.33
7.32
7.31
7.29
7.27
7.27
6.37
6.33
6.26
6.24
6.22
6.22
6.20
6.19
2.25
2.23
2.21
2.20
2.18
1.31
1.10
1.08
1.06
4
4.47
1.00
1.03
2.01
9.14
3.07
f1 (ppm)

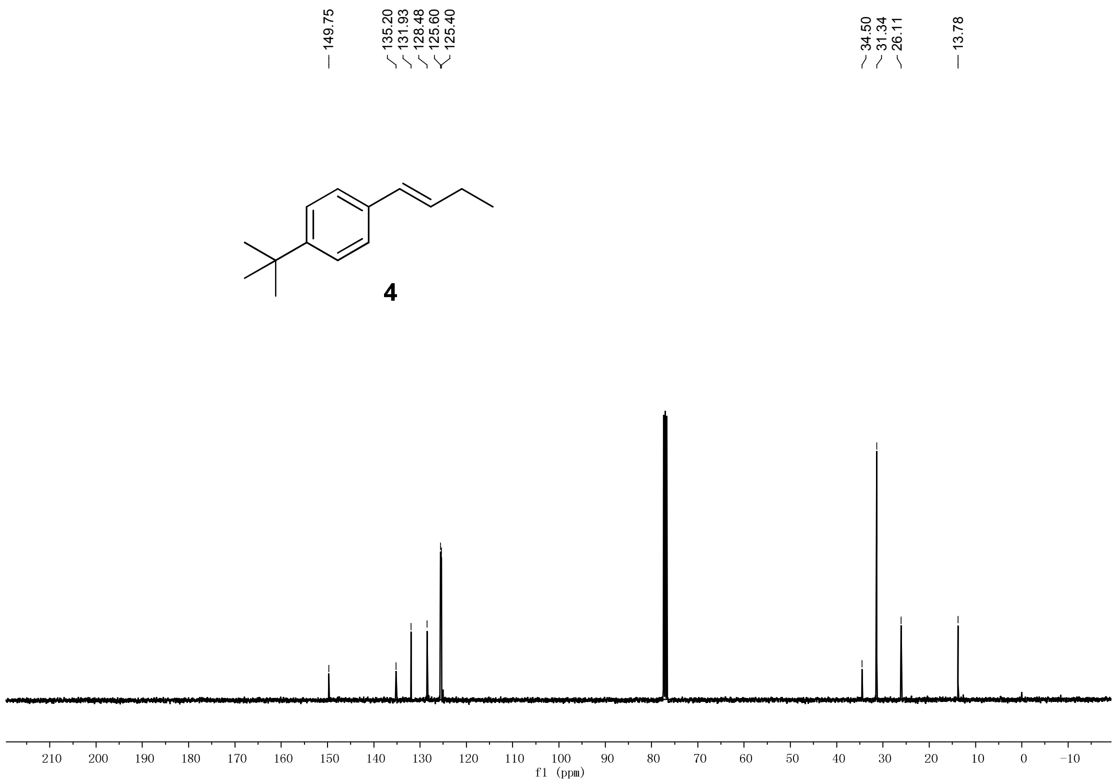
149.75
135.20
131.93
128.48
125.60
125.40
34.50
31.34
26.11
13.78
4
f1 (ppm)

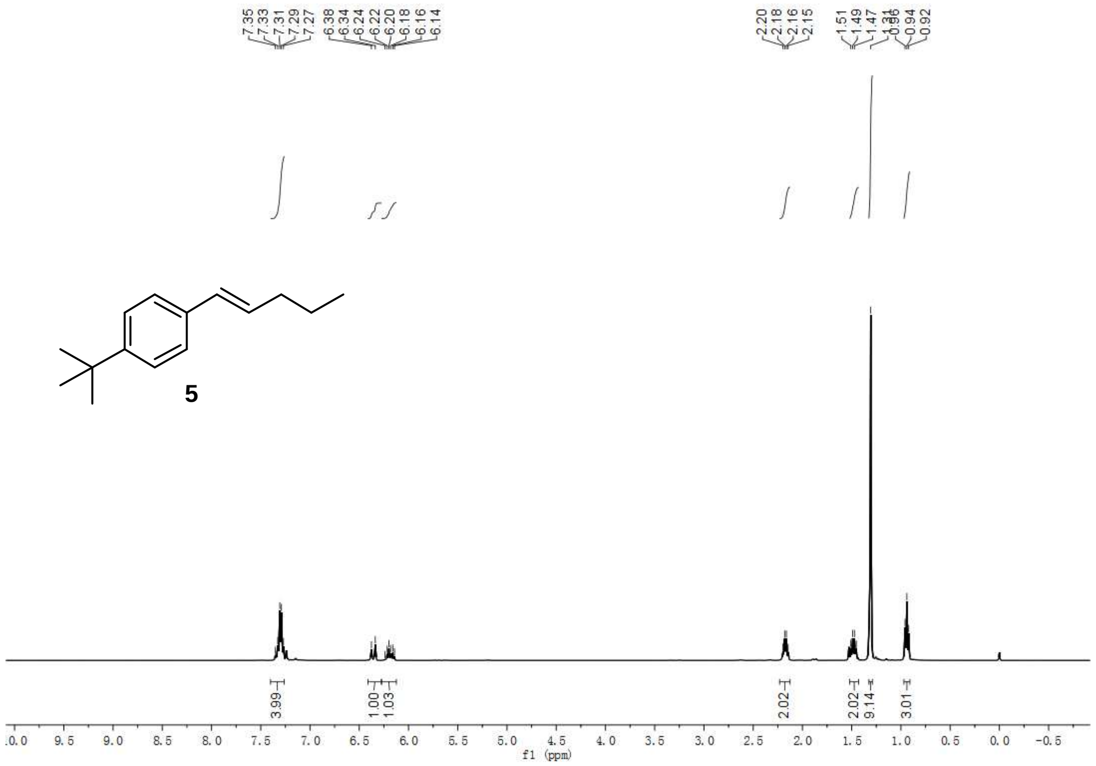

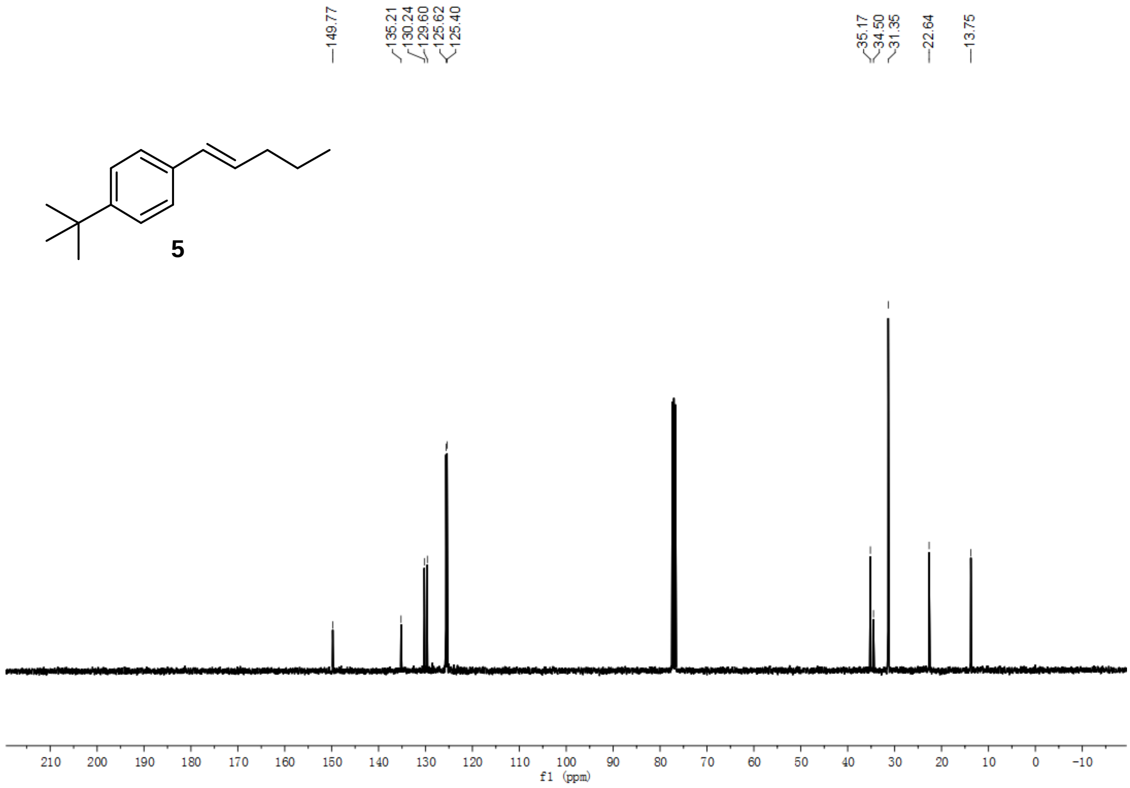

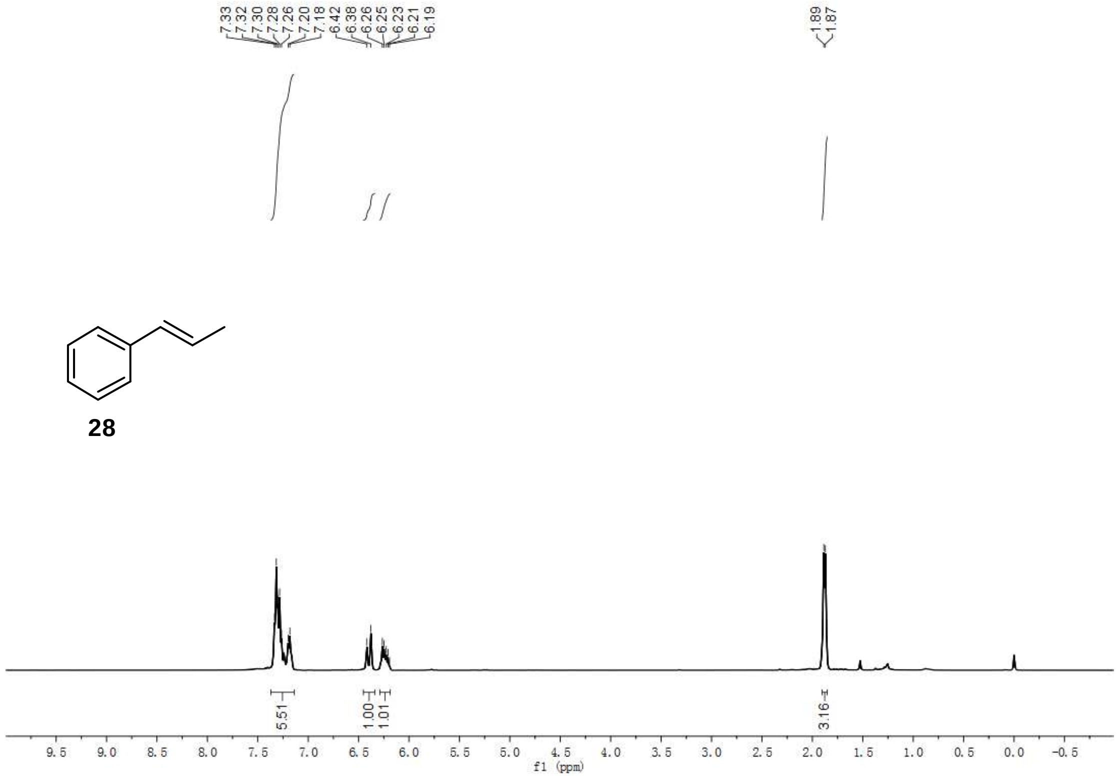
7.33
7.32
7.30
7.28
7.26
7.20
7.18
6.42
6.38
6.26
6.25
6.23
6.21
6.19
1.89
1.87
28
5.51
1.00
1.01
3.16
f1 (ppm)

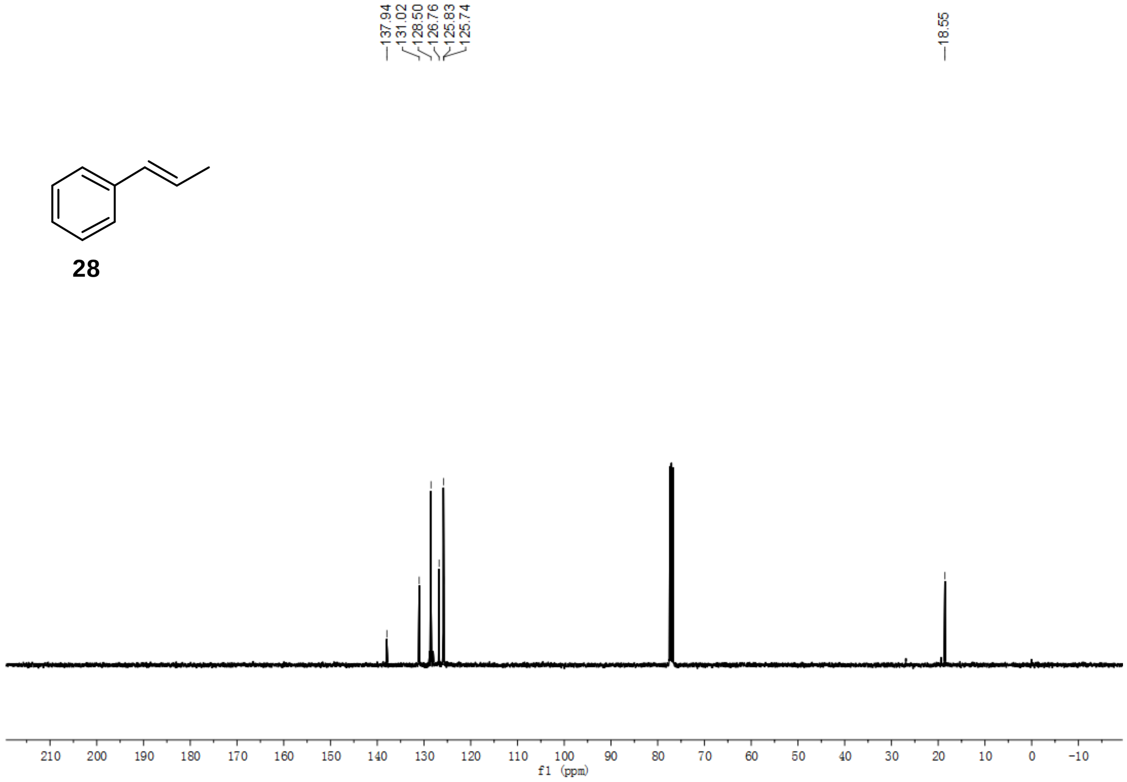
137.94
131.02
128.50
126.76
125.83
125.74
18.55
28
f1 (ppm)

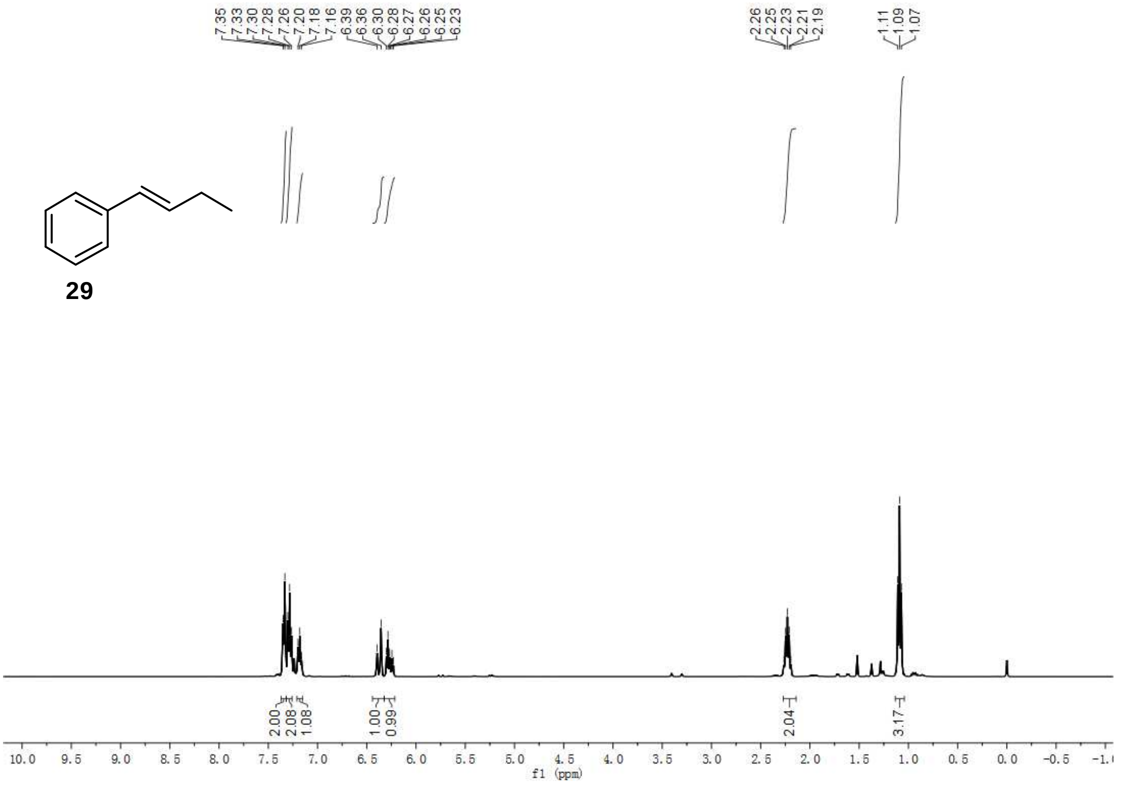
7.35
7.33
7.30
7.28
7.26
7.20
7.18
7.16
6.39
6.36
6.30
6.28
6.27
6.26
6.25
6.23
2.26
2.25
2.23
2.21
2.19
1.11
1.09
1.07
29
2.00
2.08
1.08
1.00
0.99
2.04
3.17
10.0 9.5 9.0 8.5 8.0 7.5 7.0 6.5 6.0 5.5 5.0 4.5 4.0 3.5 3.0 2.5 2.0 1.5 1.0 0.5 0.0 -0.5
f1 (ppm)

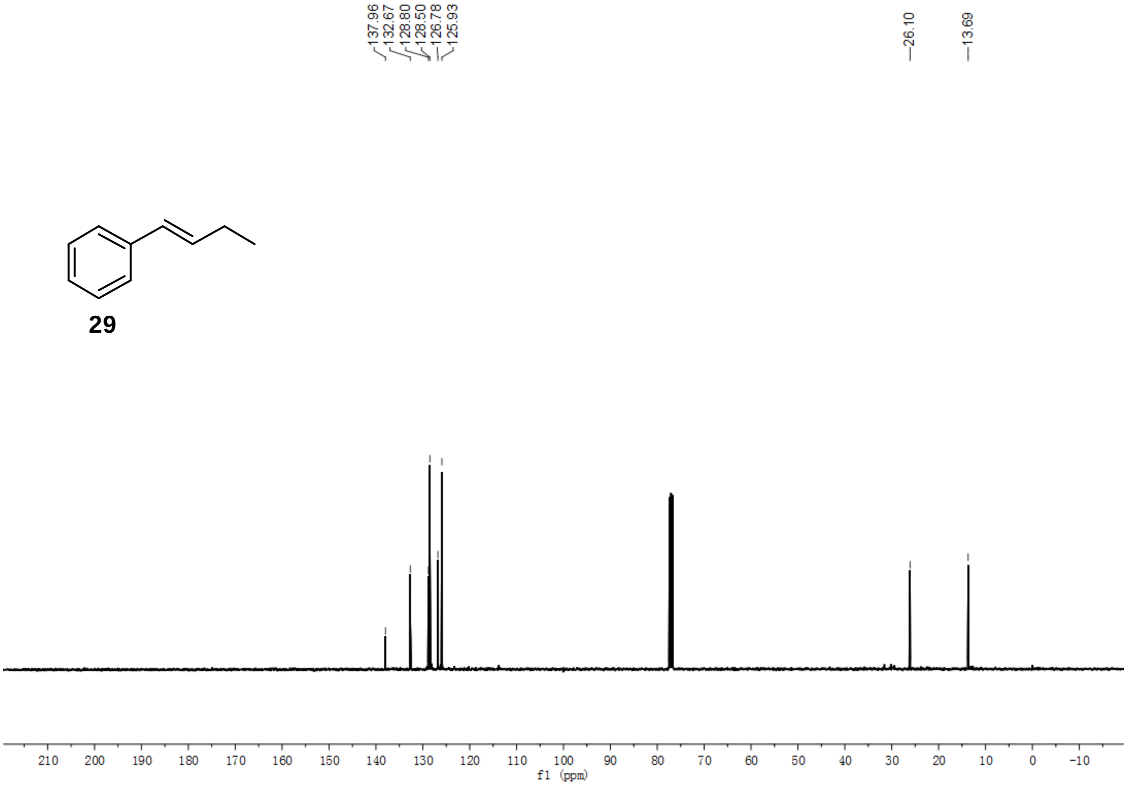
137.96
132.67
128.80
128.50
126.78
125.93
26.10
13.69
29
210 200 190 180 170 160 150 140 130 120 110 100 90 80 70 60 50 40 30 20 10 0 -10
f1 (ppm)

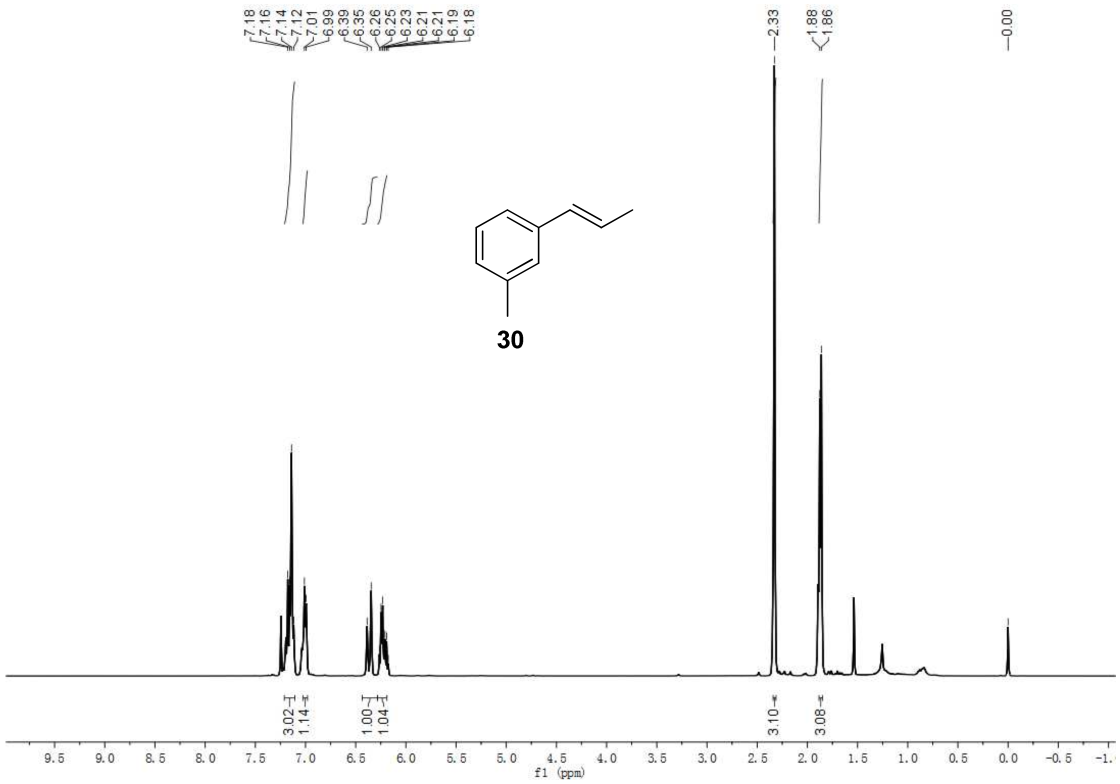
7.18
7.16
7.14
7.12
7.01
6.99
6.39
6.35
6.26
6.25
6.23
6.21
6.21
6.19
6.18
2.33
1.88
1.86
0.00
30
3.02
1.14
1.00
1.04
3.10
3.08
9.5 9.0 8.5 8.0 7.5 7.0 6.5 6.0 5.5 5.0 4.5 4.0 3.5 3.0 2.5 2.0 1.5 1.0 0.5 0.0 -0.5 -1.
f1 (ppm)

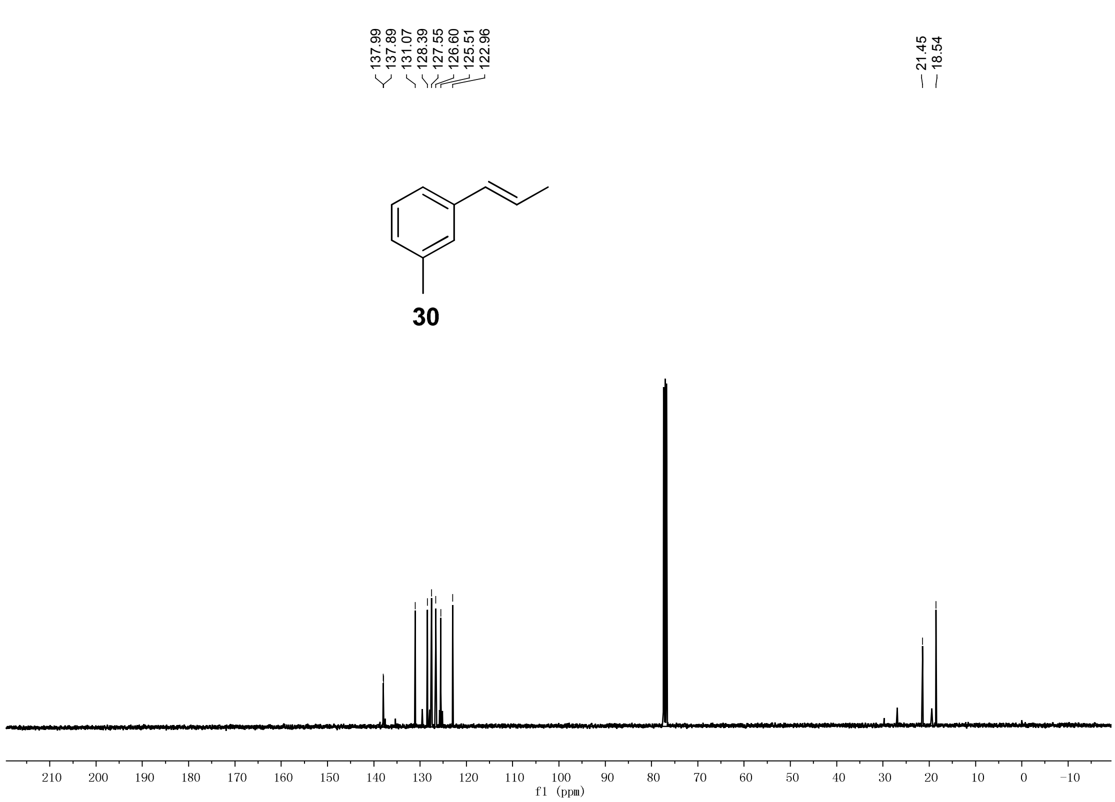
137.99
137.89
131.07
128.39
127.55
126.60
125.51
122.96
21.45
18.54
30
210 200 190 180 170 160 150 140 130 120 110 100 90 80 70 60 50 40 30 20 10 0 -10
f1 (ppm)

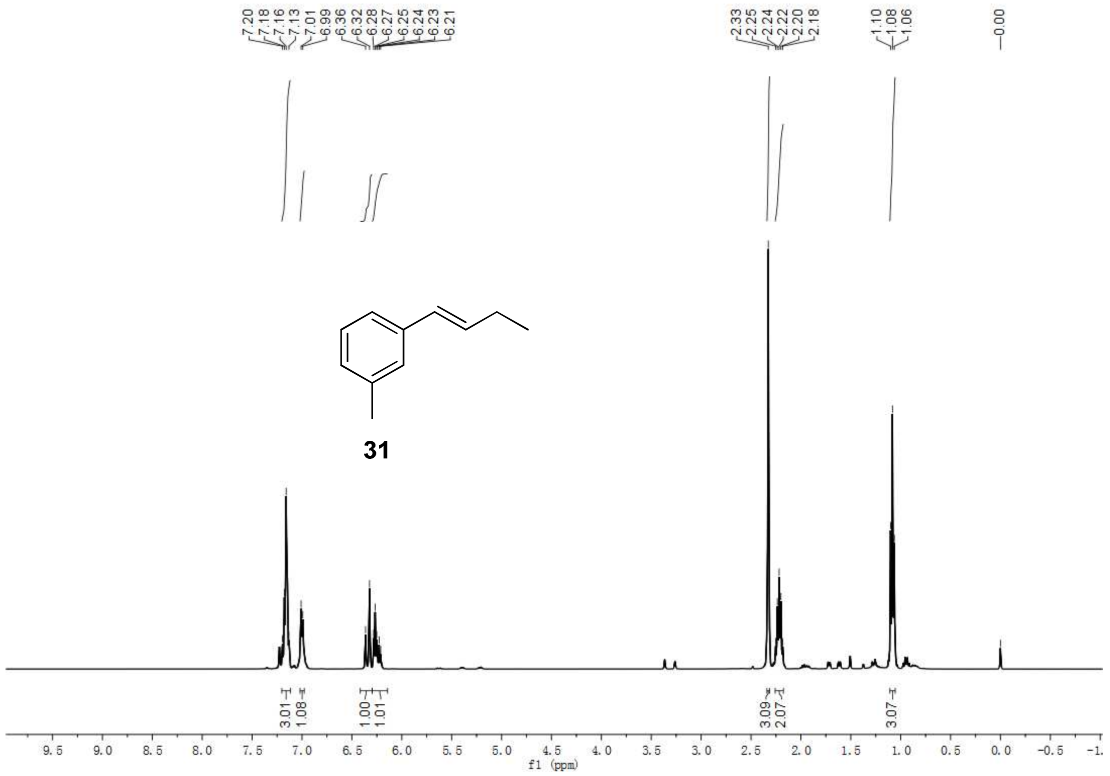
7.20
7.18
7.16
7.13
7.01
6.99
6.36
6.32
6.28
6.27
6.25
6.24
6.23
6.21
2.33
2.25
2.24
2.22
2.20
2.18
1.10
1.08
1.06
0.00
31
3.01
1.08
1.00
1.01
3.09
2.07
3.07
f1 (ppm)

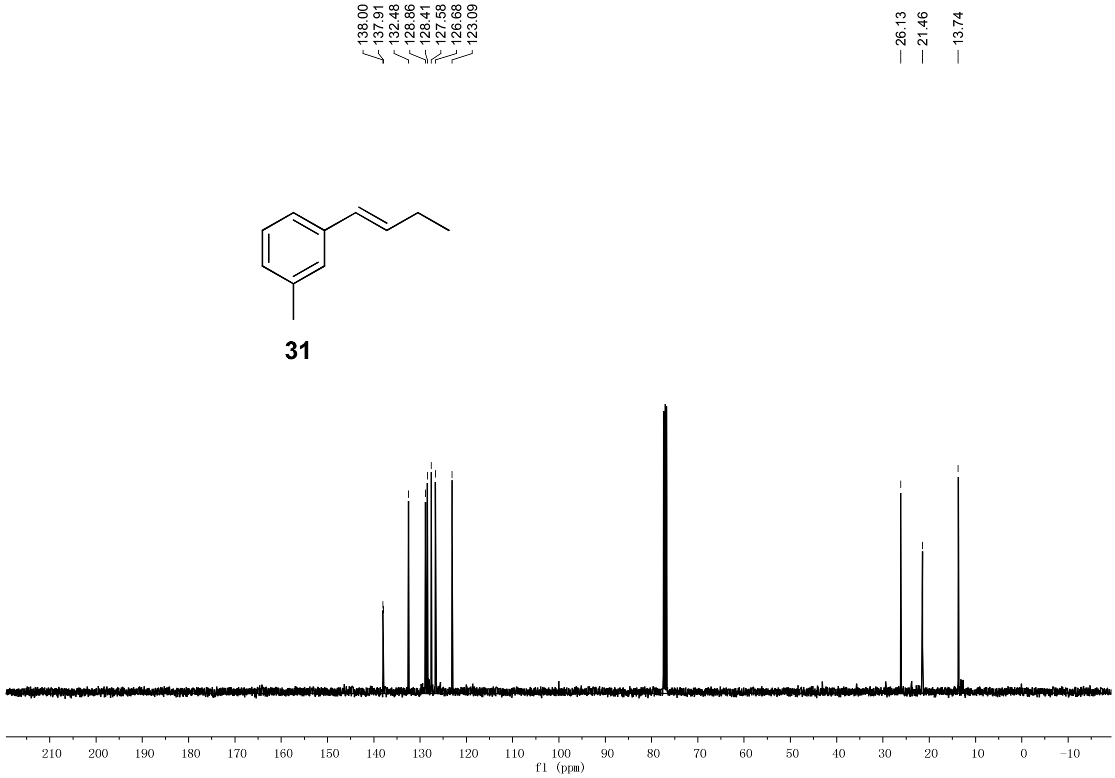
138.00
137.91
132.48
128.86
128.41
127.58
126.68
123.09
26.13
21.46
13.74
31
f1 (ppm)

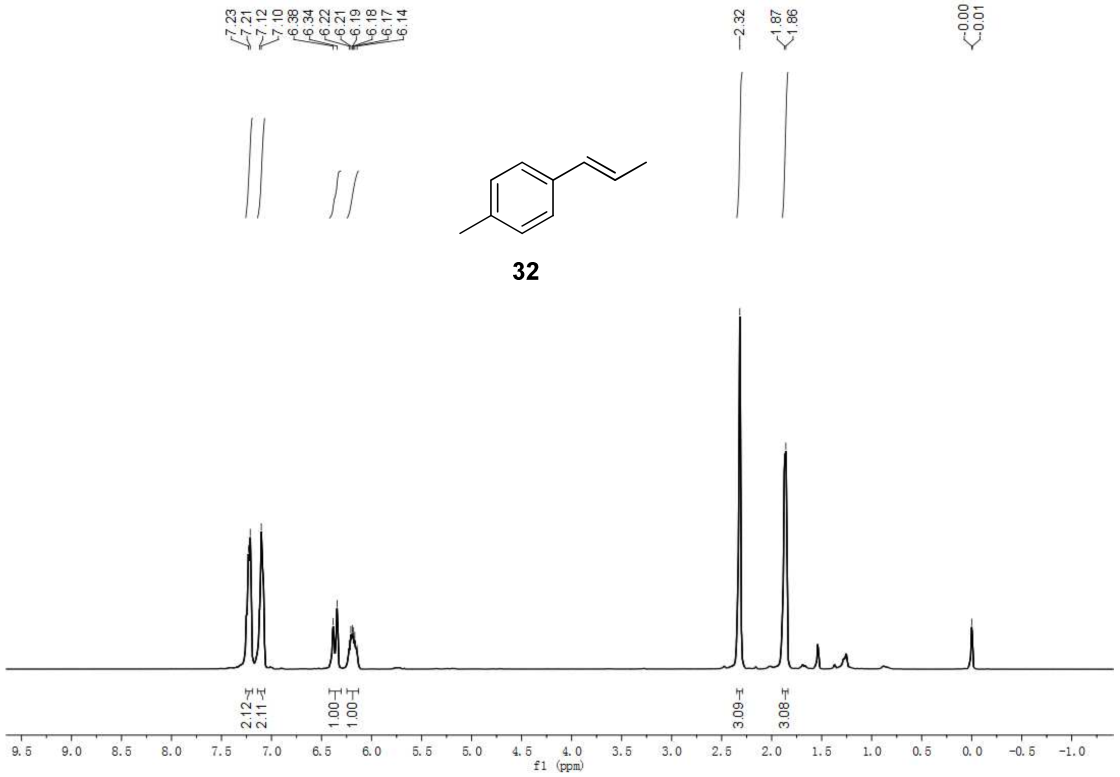
7.23
7.21
7.12
7.10
6.38
6.34
6.22
6.21
6.19
6.18
6.17
6.14
2.32
1.87
1.86
0.00
0.01
32
2.12
2.11
1.00
1.00
3.09
3.08
9.5 9.0 8.5 8.0 7.5 7.0 6.5 6.0 5.5 5.0 4.5 4.0 3.5 3.0 2.5 2.0 1.5 1.0 0.5 0.0 -0.5 -1.0
f1 (ppm)

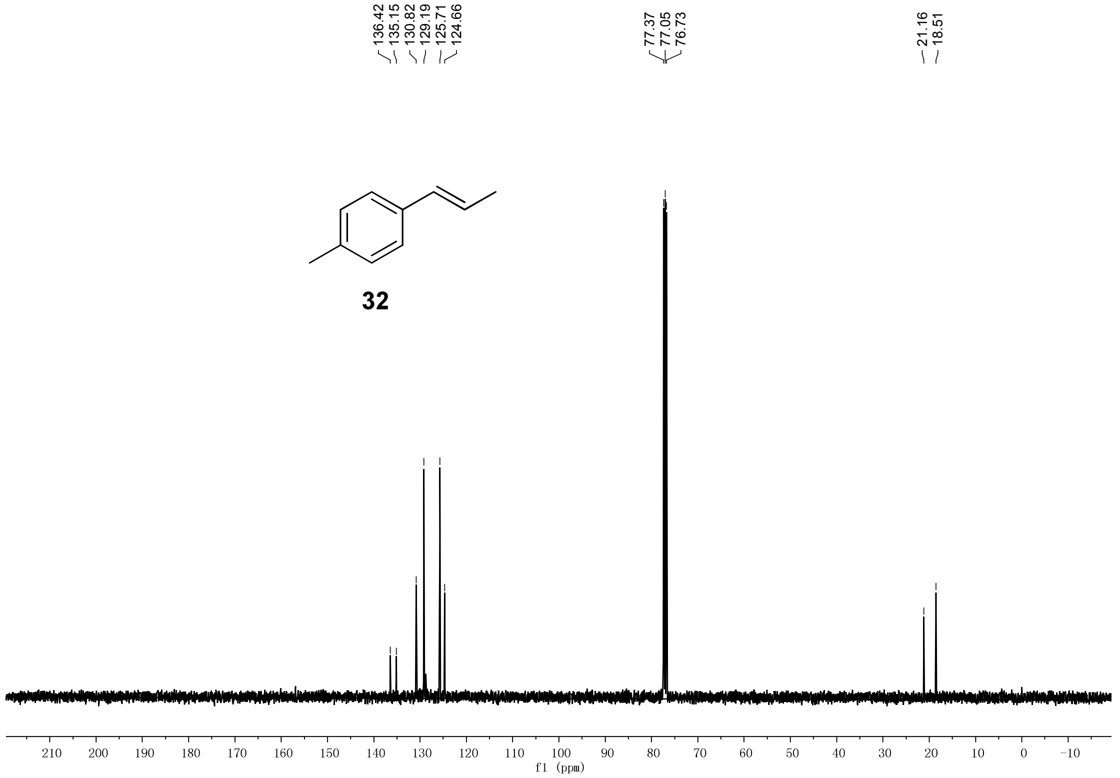
136.42
135.15
130.82
129.19
125.71
124.66
77.37
77.05
76.73
21.16
18.51
32
210 200 190 180 170 160 150 140 130 120 110 100 90 80 70 60 50 40 30 20 10 0 -10
f1 (ppm)

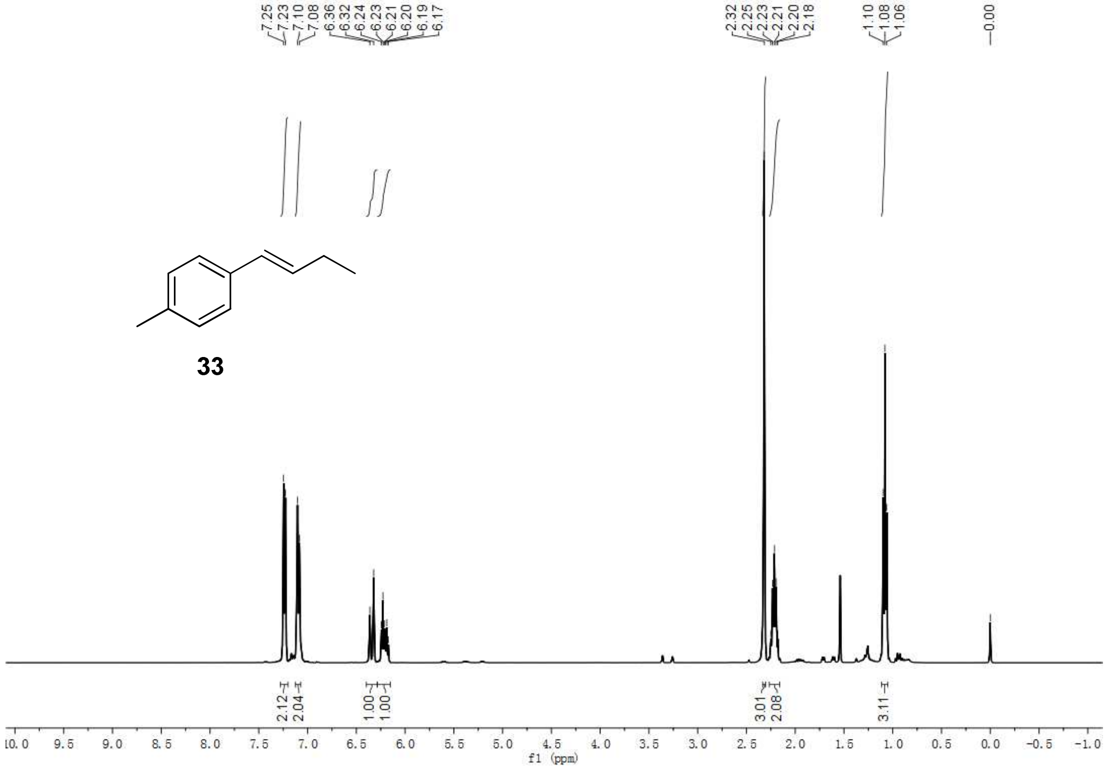
7.25
7.23
7.10
7.08
6.36
6.32
6.24
6.23
6.21
6.20
6.19
6.17
2.32
2.25
2.23
2.21
2.20
2.18
1.10
1.08
1.06
0.00
33
2.12
2.04
1.00
1.00
3.01
2.08
3.11
f1 (ppm)

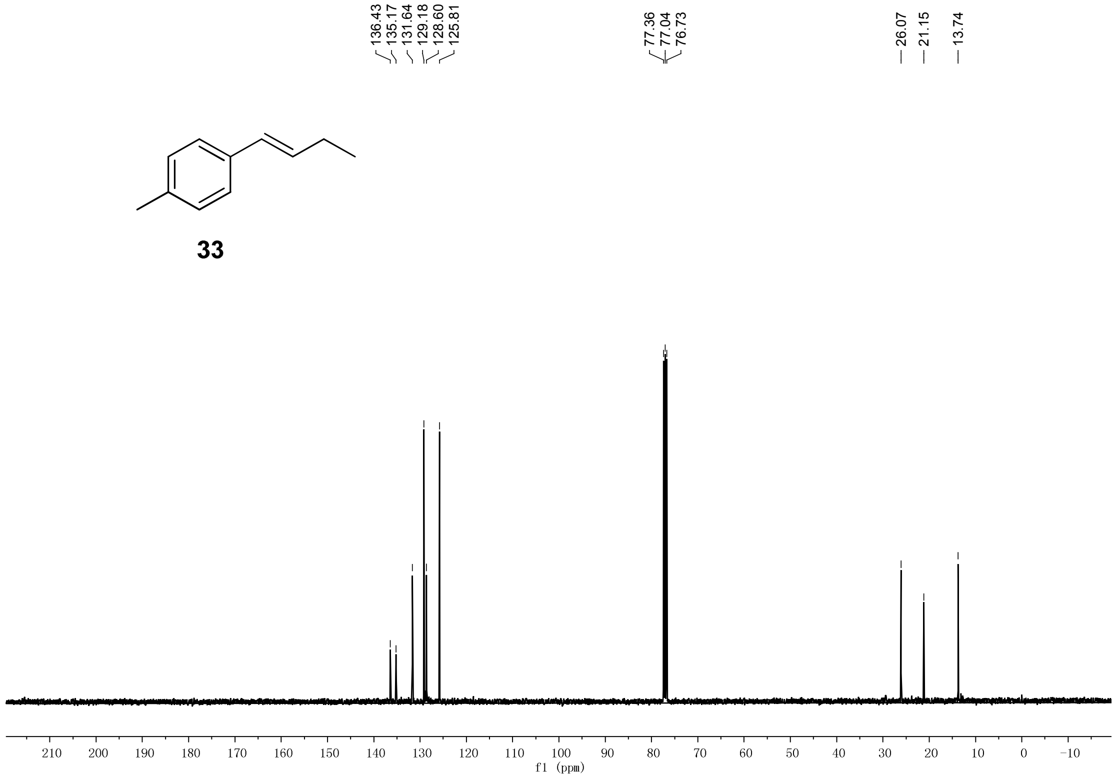
136.43
135.17
131.64
129.18
128.60
125.81
77.36
77.04
76.73
26.07
21.15
13.74
33
f1 (ppm)

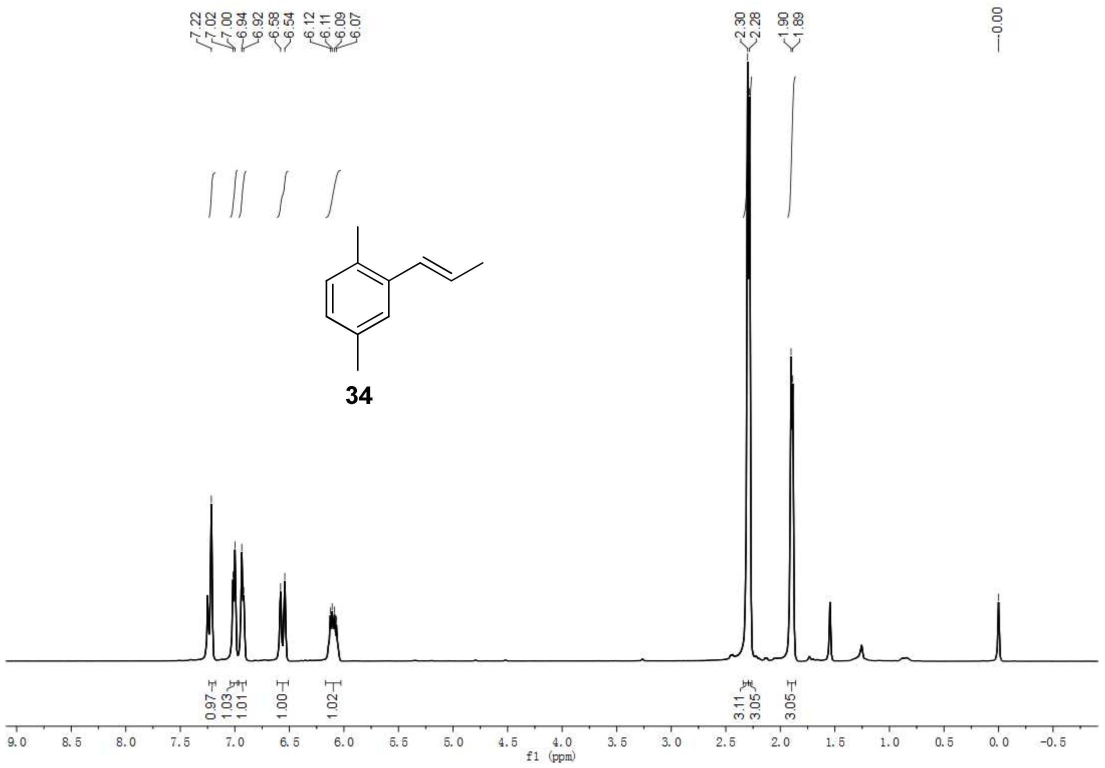

7.22
7.02
7.00
6.94
6.92
6.58
6.54
6.12
6.11
6.09
6.07
2.30
2.28
1.90
1.89
0.00
34
0.97
1.03
1.01
1.00
1.02
3.11
3.05
3.05
9.0 8.5 8.0 7.5 7.0 6.5 6.0 5.5 5.0 4.5 4.0 3.5 3.0 2.5 2.0 1.5 1.0 0.5 0.0 -0.5
f1 (ppm)

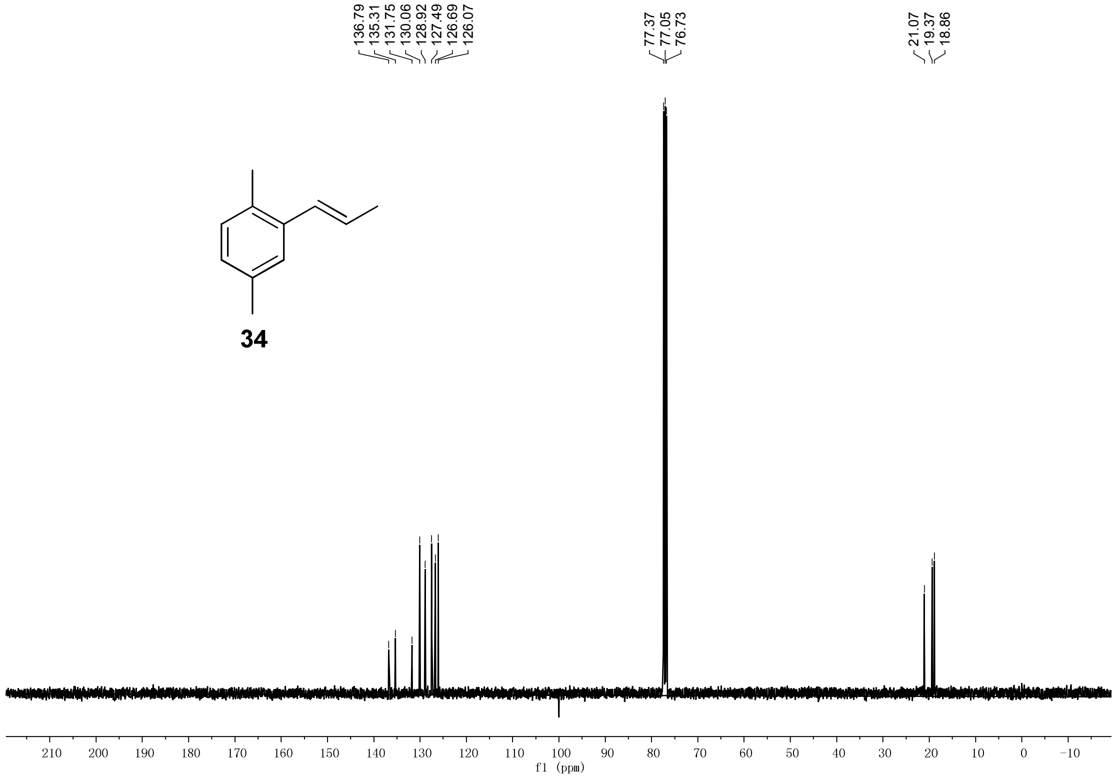

136.79
135.31
131.75
130.06
128.92
127.49
126.69
126.07
77.37
77.05
76.73
21.07
19.37
18.86
34
210 200 190 180 170 160 150 140 130 120 110 100 90 80 70 60 50 40 30 20 10 0 -10
f1 (ppm)

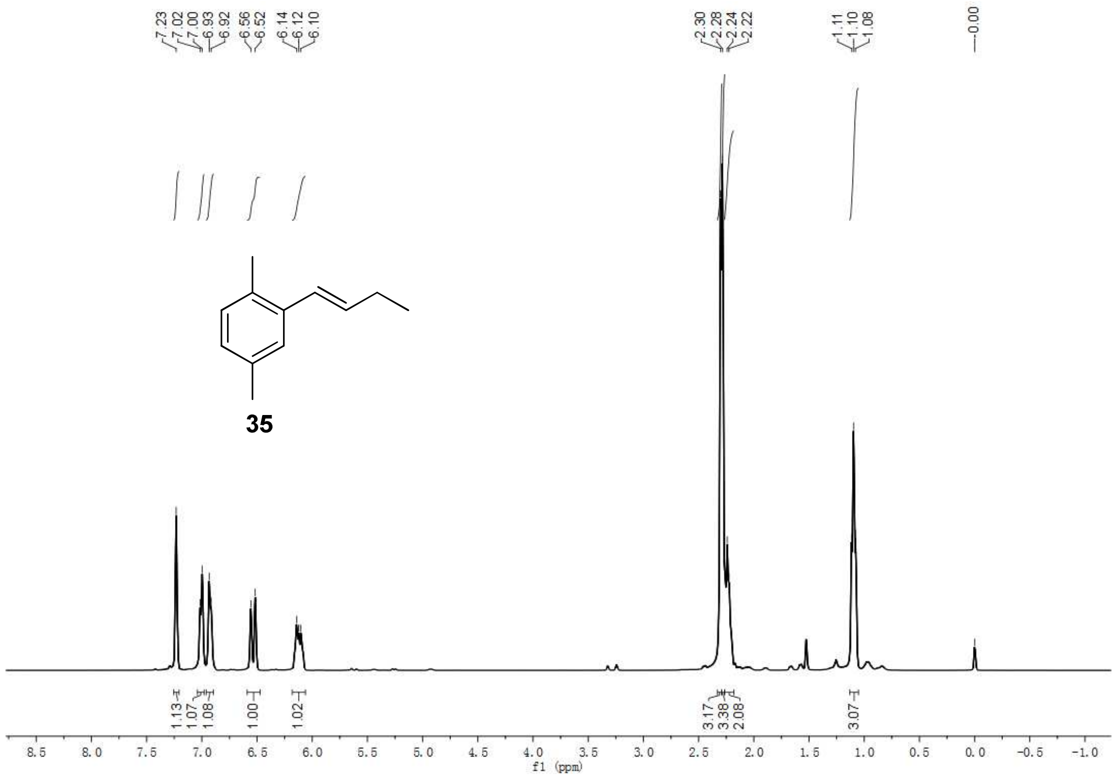
7.23 7.02 7.00 6.93 6.92 6.56 6.52 6.14 6.12 6.10
2.30 2.28 2.24 2.22
1.11 1.10 1.08
0.00
35
1.13 1.07 1.08 1.00 1.02
3.17 3.38 2.08
3.07
f1 (ppm)

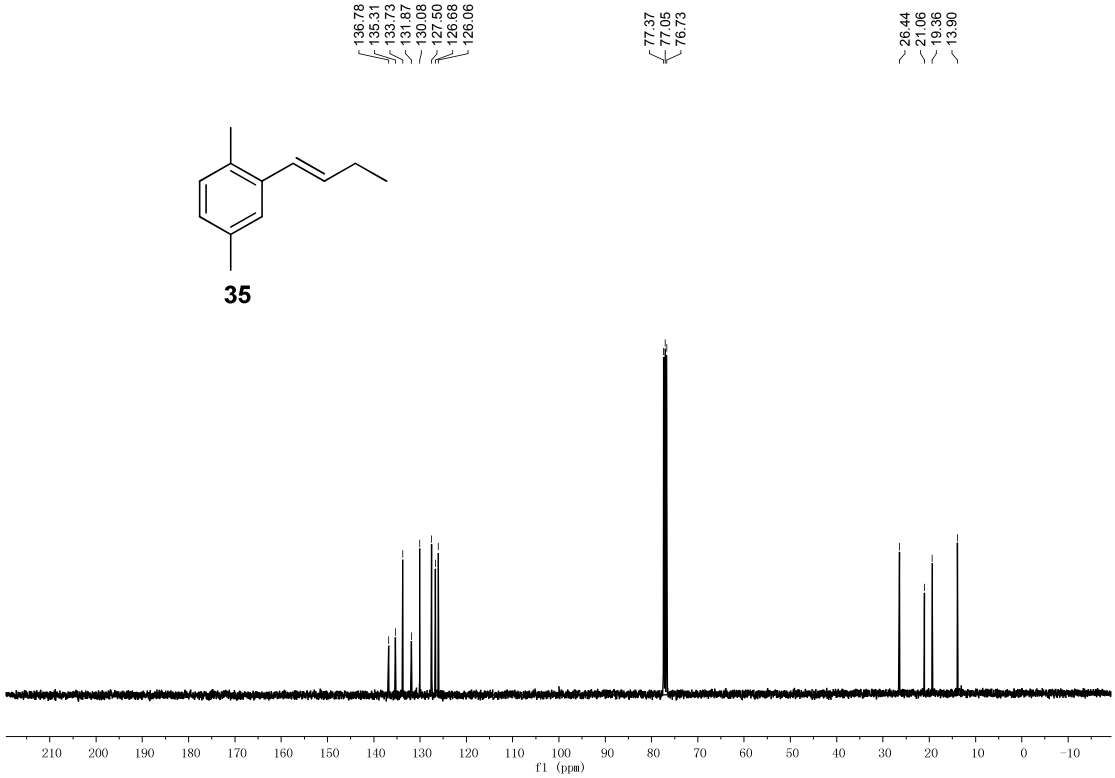
136.78 135.31 133.73 131.87 130.08 127.50 126.68 126.06
77.37 77.05 76.73
26.44 21.06 19.36 13.90
35
f1 (ppm)

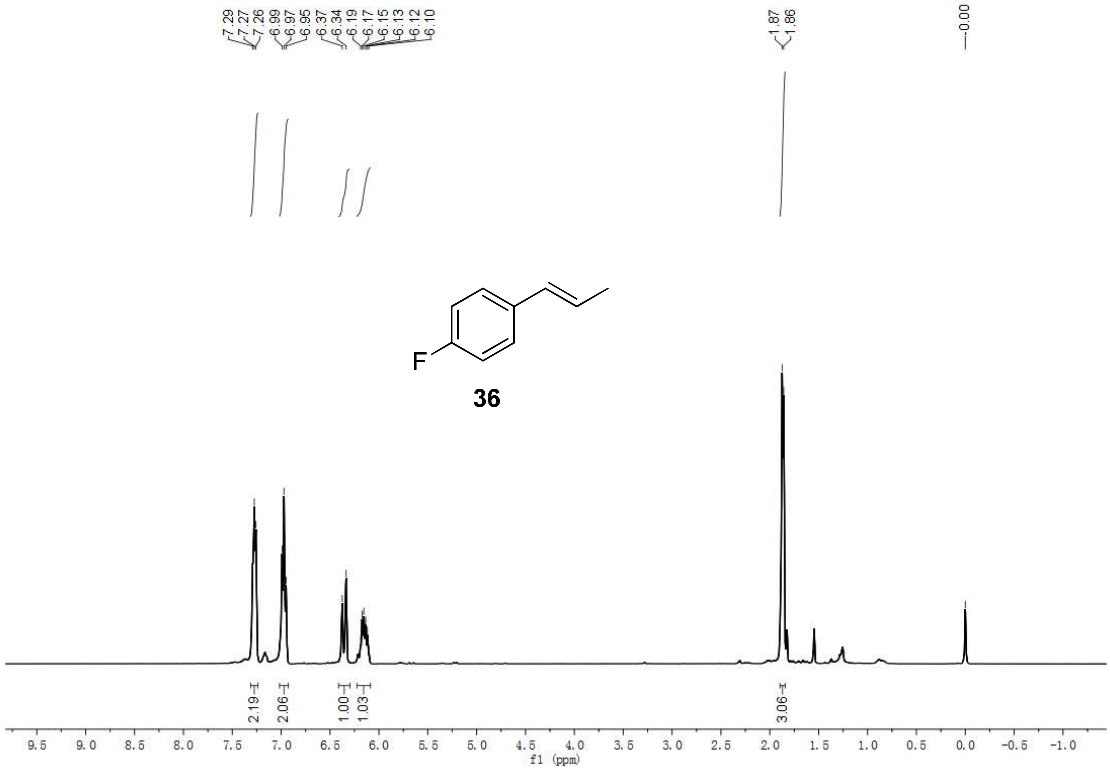
7.29
7.27
7.26
6.99
6.97
6.95
6.37
6.34
6.19
6.17
6.15
6.13
6.12
6.10
1.87
1.86
0.00
F
36
2.19
2.06
1.00
1.03
3.06
9.5 9.0 8.5 8.0 7.5 7.0 6.5 6.0 5.5 5.0 4.5 4.0 3.5 3.0 2.5 2.0 1.5 1.0 0.5 0.0 -0.5 -1.0
f1 (ppm)

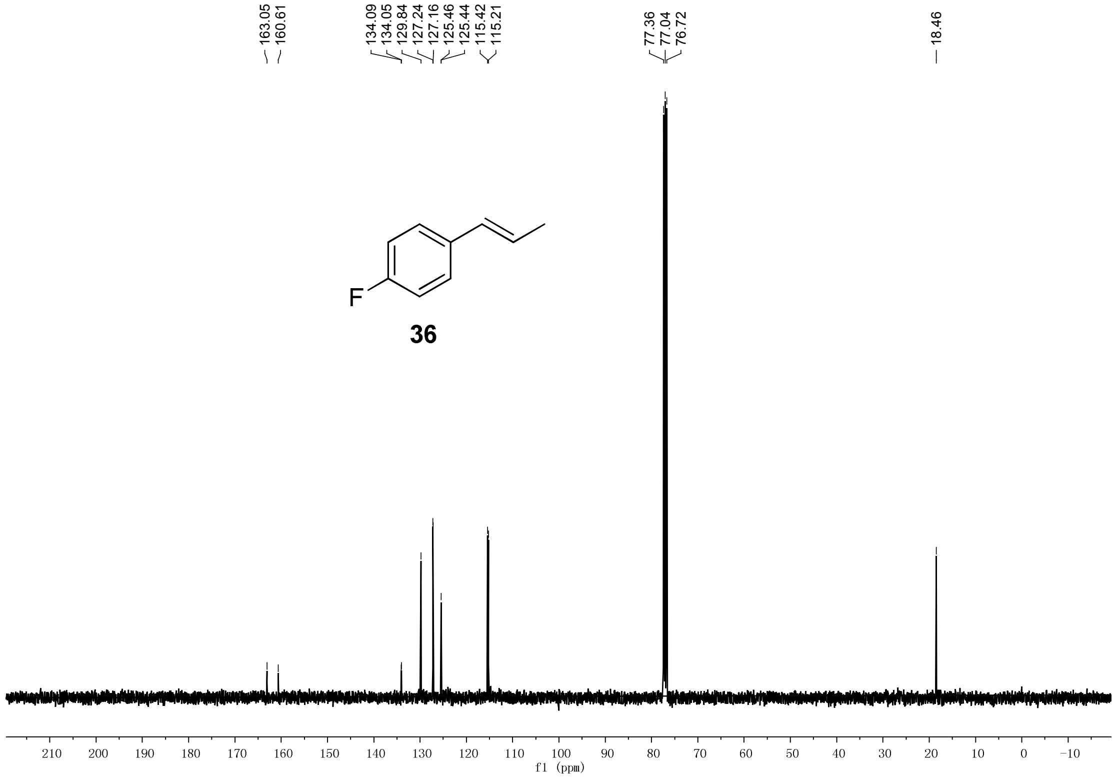
163.05
160.61
134.09
134.05
129.84
127.24
127.16
125.46
125.44
115.42
115.21
77.36
77.04
76.72
18.46
F
36
210 200 190 180 170 160 150 140 130 120 110 100 90 80 70 60 50 40 30 20 10 0 -10
f1 (ppm)

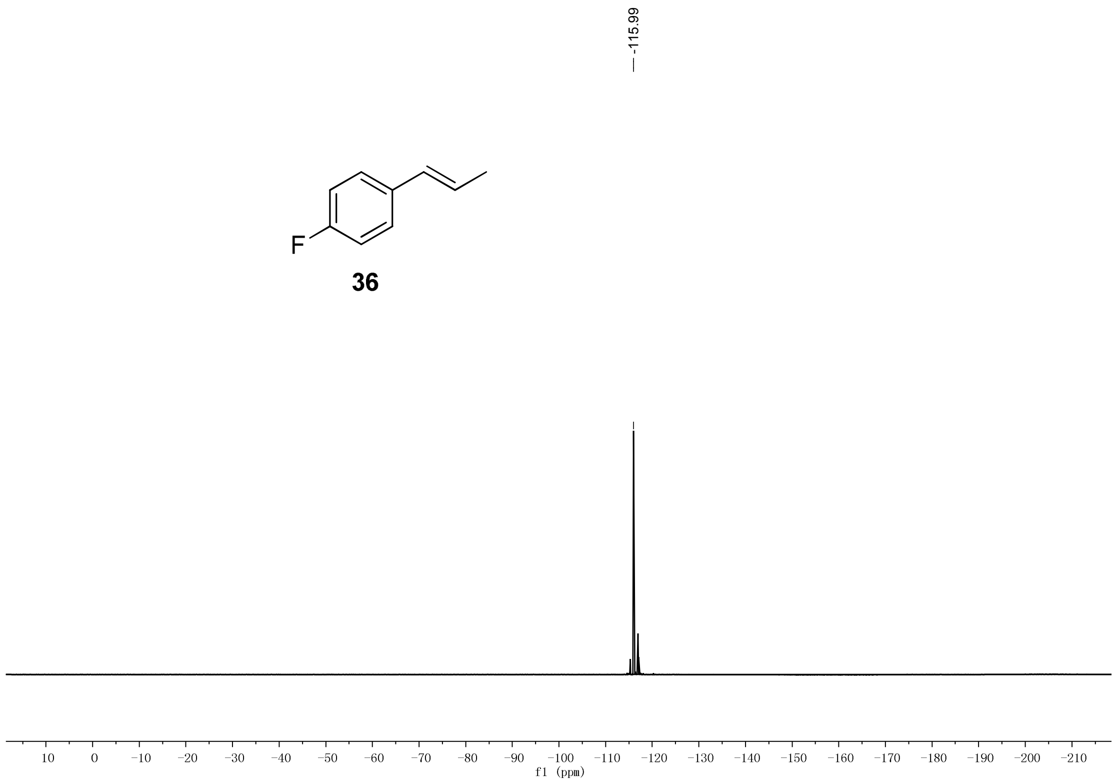
-115.99
F
36
10 0 −10 −20 −30 −40 −50 −60 −70 −80 −90 −100 −110 −120 −130 −140 −150 −160 −170 −180 −190 −200 −210
f1 (ppm)

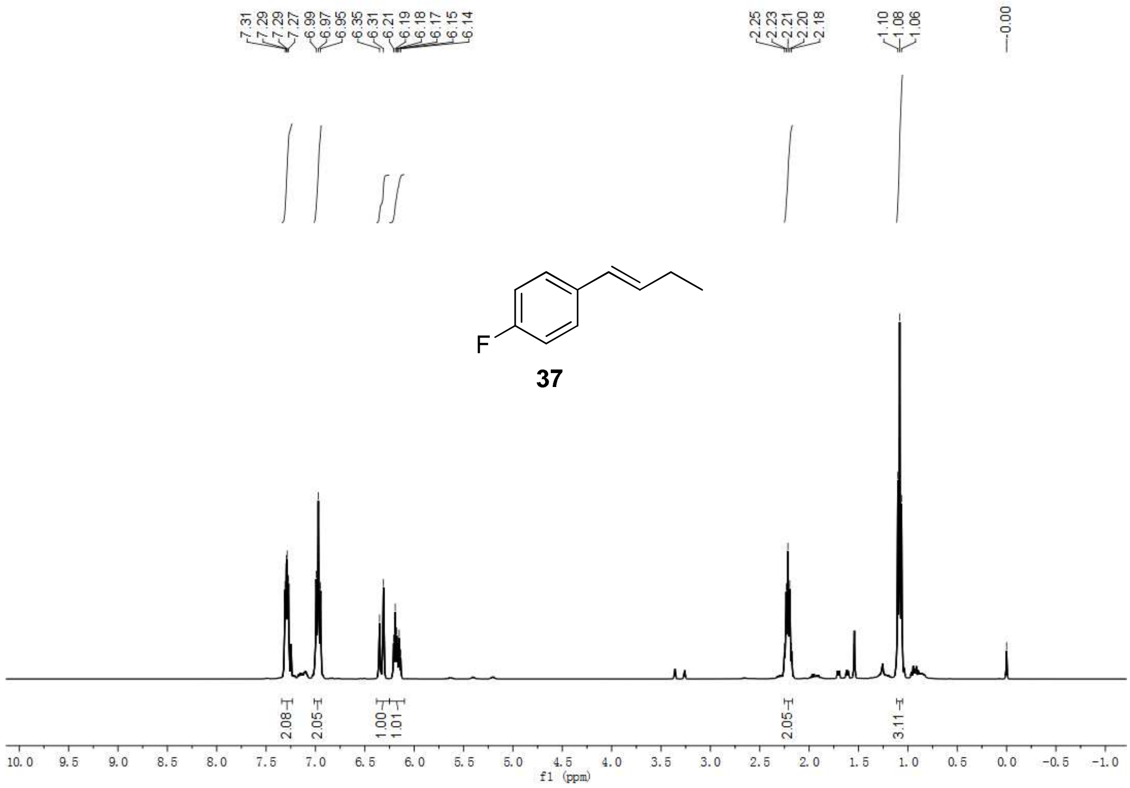
7.31 7.29 7.29 7.27 6.99 6.97 6.95 6.35 6.31 6.21 6.19 6.18 6.17 6.15 6.14
2.25 2.23 2.21 2.20 2.18
1.10 1.08 1.06
0.00
F
37
2.08 2.05 1.00 1.01 2.05 3.11
10.0 9.5 9.0 8.5 8.0 7.5 7.0 6.5 6.0 5.5 5.0 4.5 4.0 3.5 3.0 2.5 2.0 1.5 1.0 0.5 0.0 −0.5 −1.0
f1 (ppm)

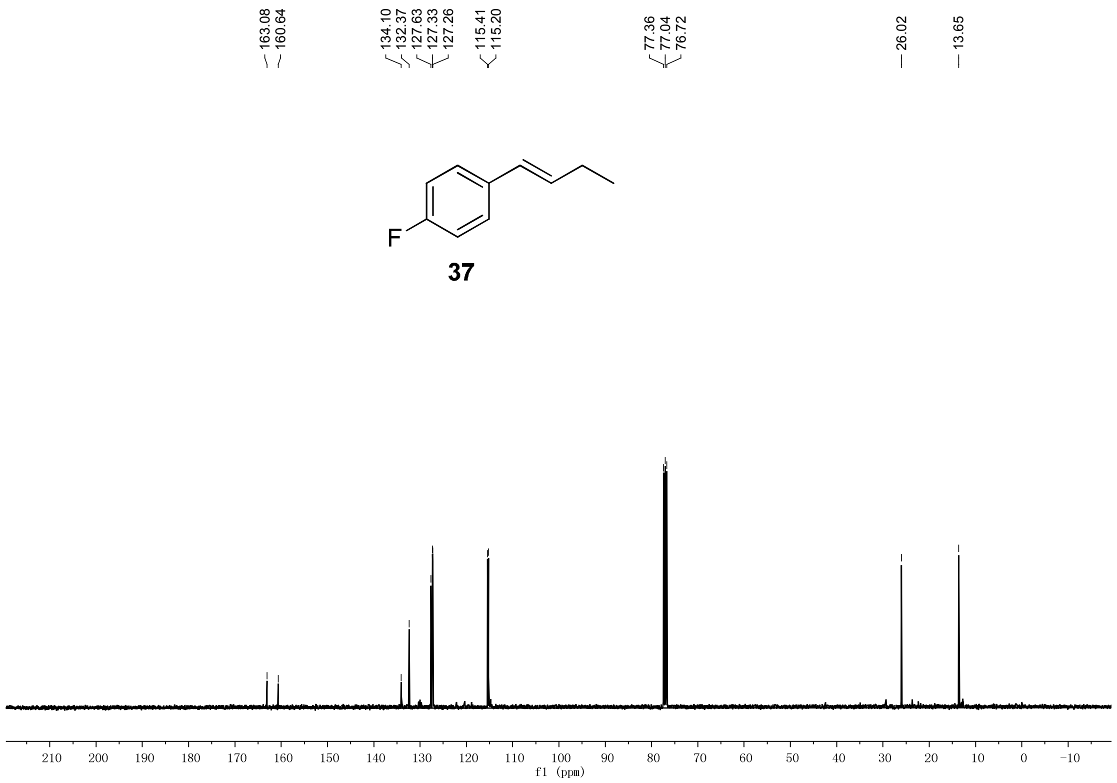
163.08
160.64
134.10
132.37
127.63
127.33
127.26
115.41
115.20
77.36
77.04
76.72
26.02
13.65
F
37
210 200 190 180 170 160 150 140 130 120 110 100 90 80 70 60 50 40 30 20 10 0 -10
f1 (ppm)

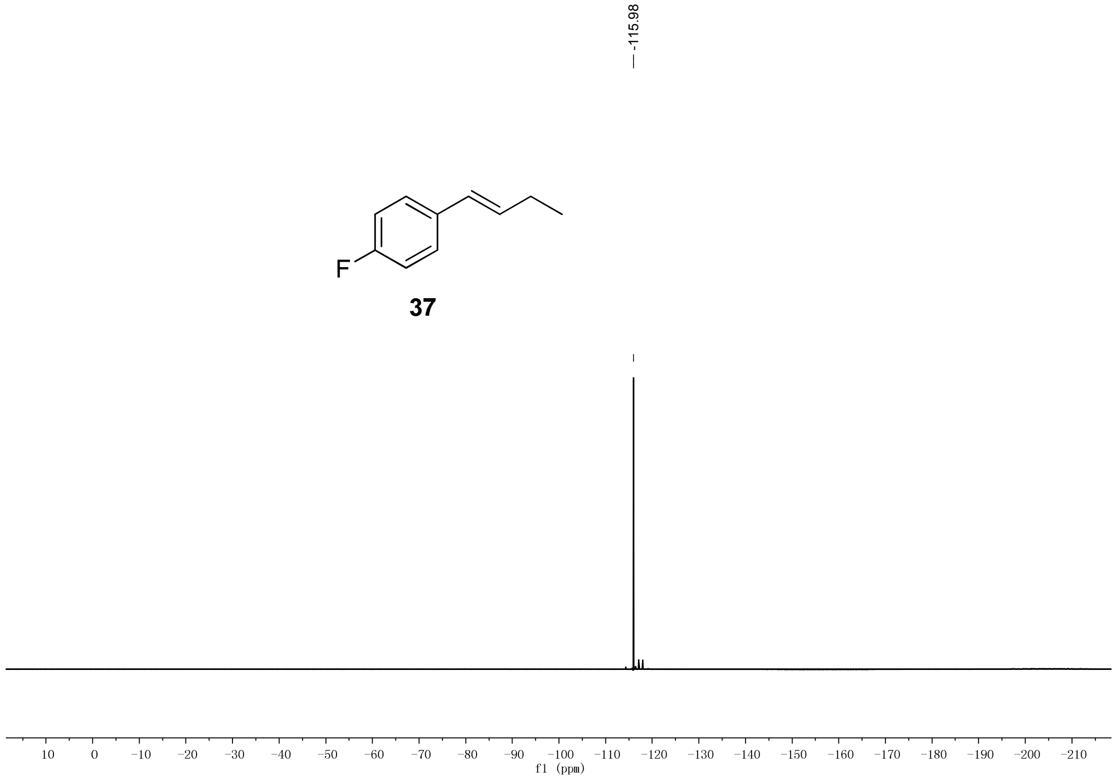
-115.98
F
37
10 0 -10 -20 -30 -40 -50 -60 -70 -80 -90 -100 -110 -120 -130 -140 -150 -160 -170 -180 -190 -200 -210
f1 (ppm)

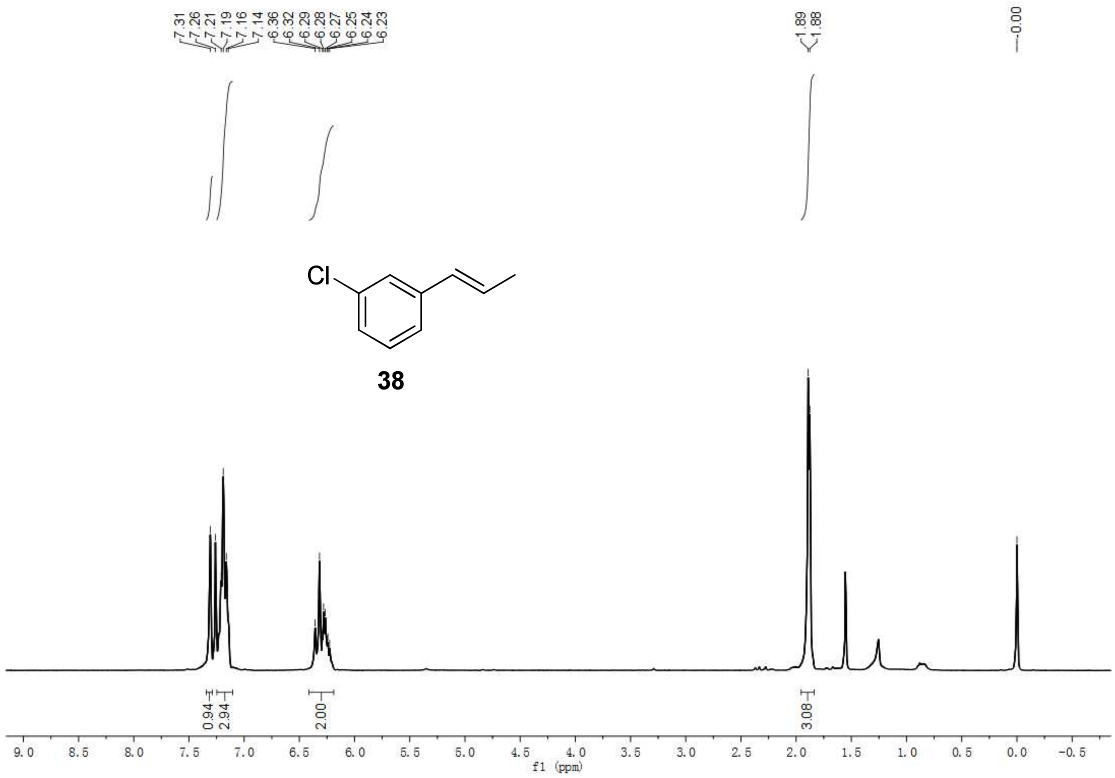
7.31
7.26
7.21
7.19
7.16
7.14
6.36
6.32
6.29
6.28
6.27
6.25
6.24
6.23
1.89
1.88
0.00
Cl
38
0.94
2.94
2.00
3.08
9.0 8.5 8.0 7.5 7.0 6.5 6.0 5.5 5.0 4.5 4.0 3.5 3.0 2.5 2.0 1.5 1.0 0.5 0.0 -0.5
f1 (ppm)

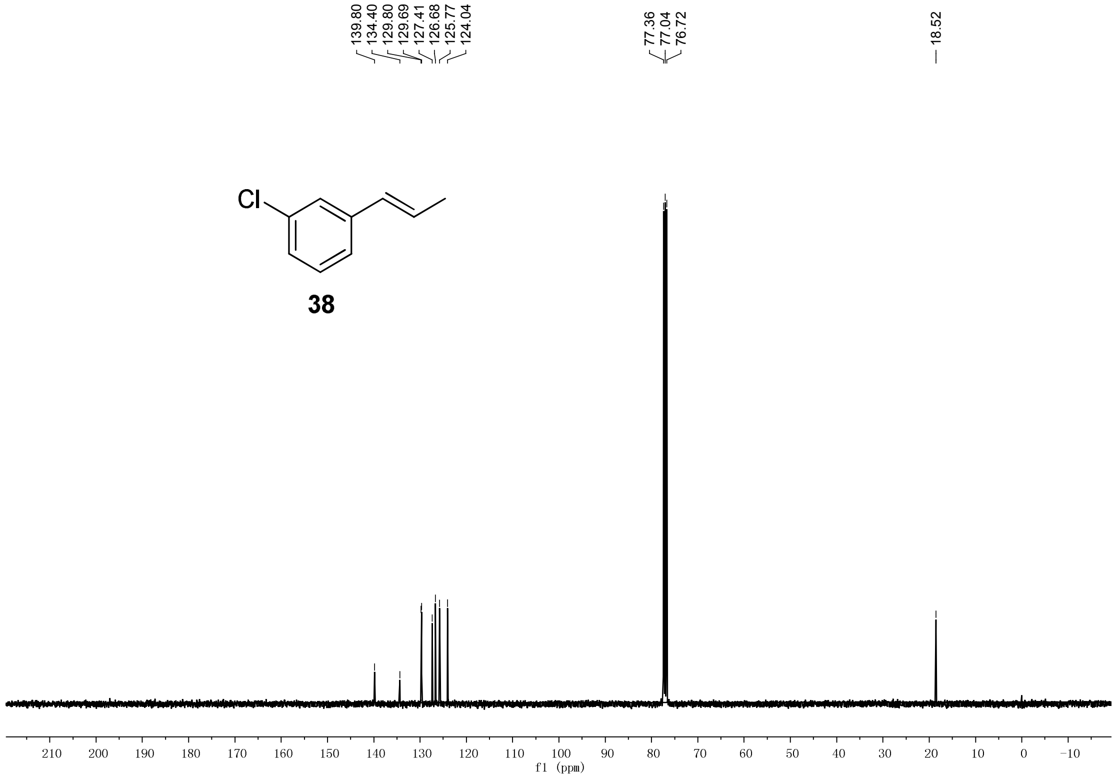
139.80
134.40
129.80
129.69
127.41
126.68
125.77
124.04
77.36
77.04
76.72
18.52
Cl
38
210 200 190 180 170 160 150 140 130 120 110 100 90 80 70 60 50 40 30 20 10 0 -10
f1 (ppm)

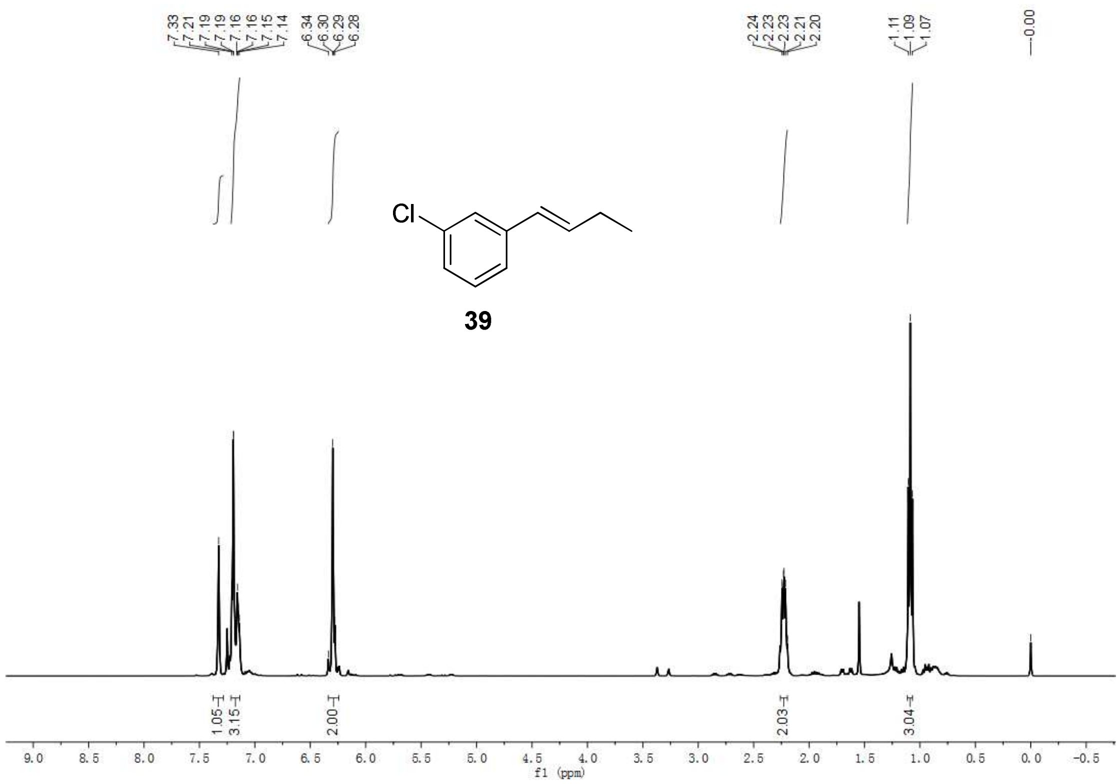
7.33
7.21
7.19
7.19
7.16
7.16
7.15
7.14
6.34
6.30
6.29
6.28
2.24
2.23
2.23
2.21
2.20
1.11
1.09
1.07
0.00
Cl
39
1.05
3.15
2.00
2.03
3.04
f1 (ppm)

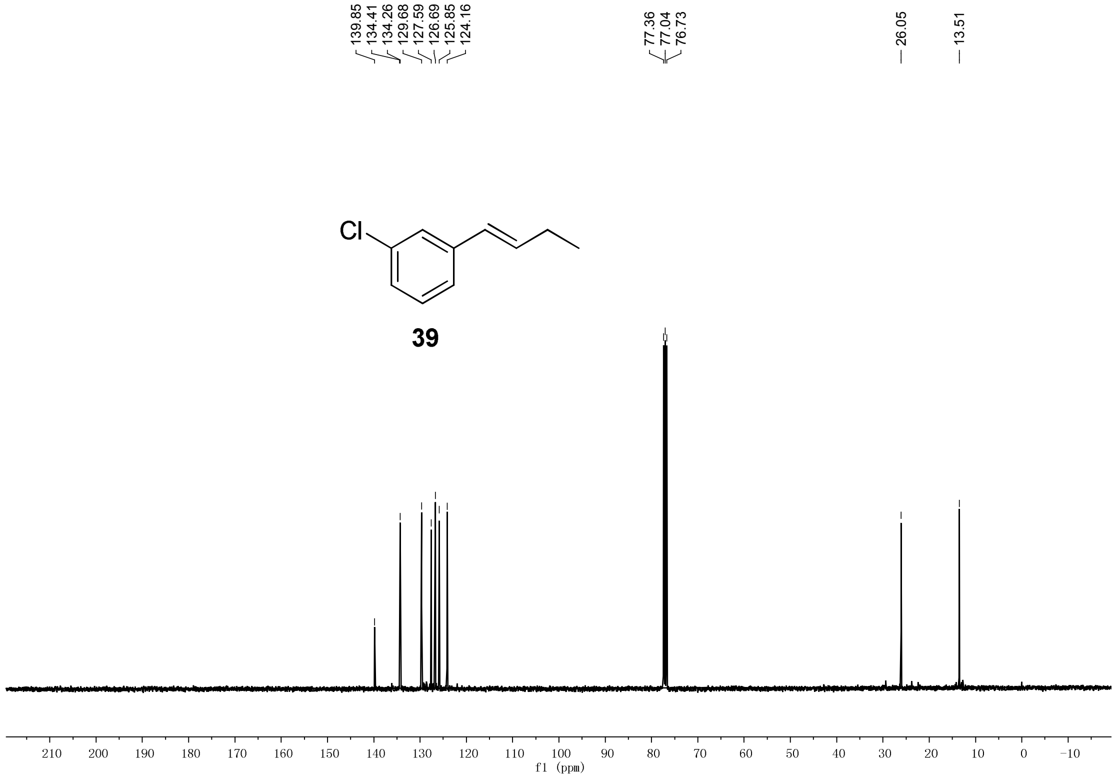
139.85
134.41
134.26
129.68
127.59
126.69
125.85
124.16
77.36
77.04
76.73
26.05
13.51
Cl
39
f1 (ppm)

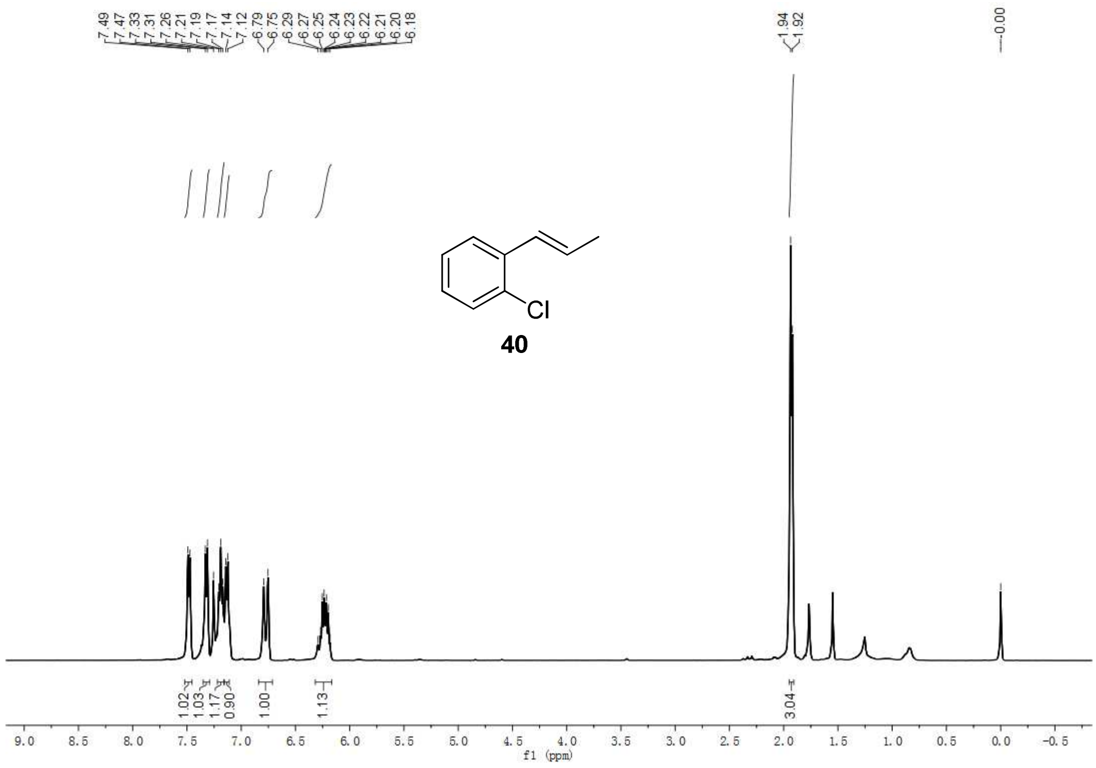

7.49
7.47
7.33
7.31
7.26
7.21
7.19
7.17
7.14
7.12
6.79
6.75
6.29
6.27
6.25
6.24
6.23
6.22
6.21
6.20
6.18
1.94
1.92
0.00
Cl
1.02
1.03
1.17
0.90
1.00
1.13
3.04
9.0 8.5 8.0 7.5 7.0 6.5 6.0 5.5 5.0 4.5 4.0 3.5 3.0 2.5 2.0 1.5 1.0 0.5 0.0 -0.5
f1 (ppm)

40

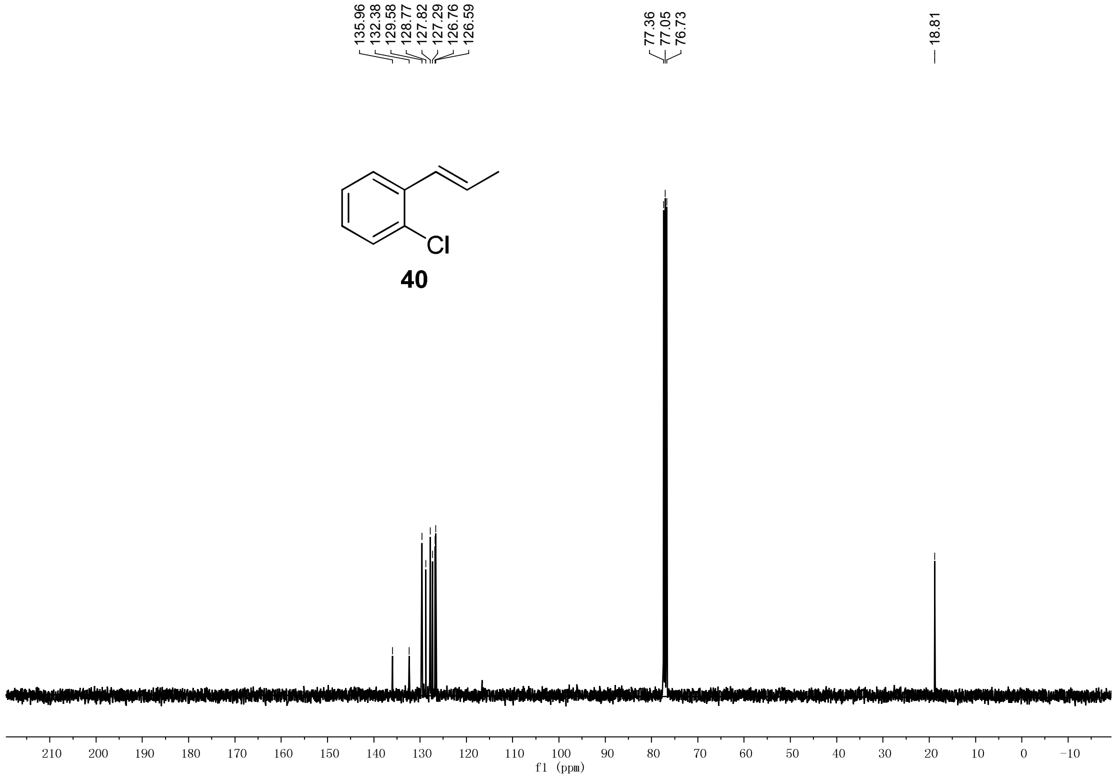

135.96
132.38
129.58
128.77
127.82
127.29
126.76
126.59
77.36
77.05
76.73
18.81
Cl
210 200 190 180 170 160 150 140 130 120 110 100 90 80 70 60 50 40 30 20 10 0 -10
f1 (ppm)

40

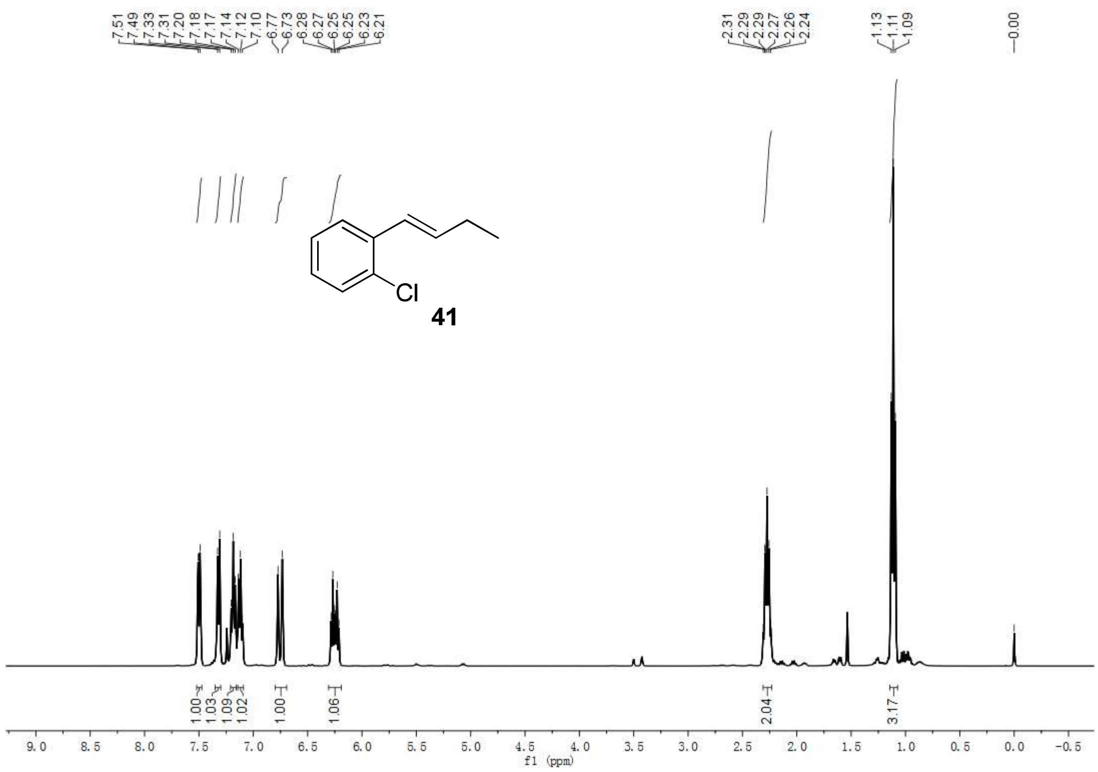
Cl
41
f1 (ppm)

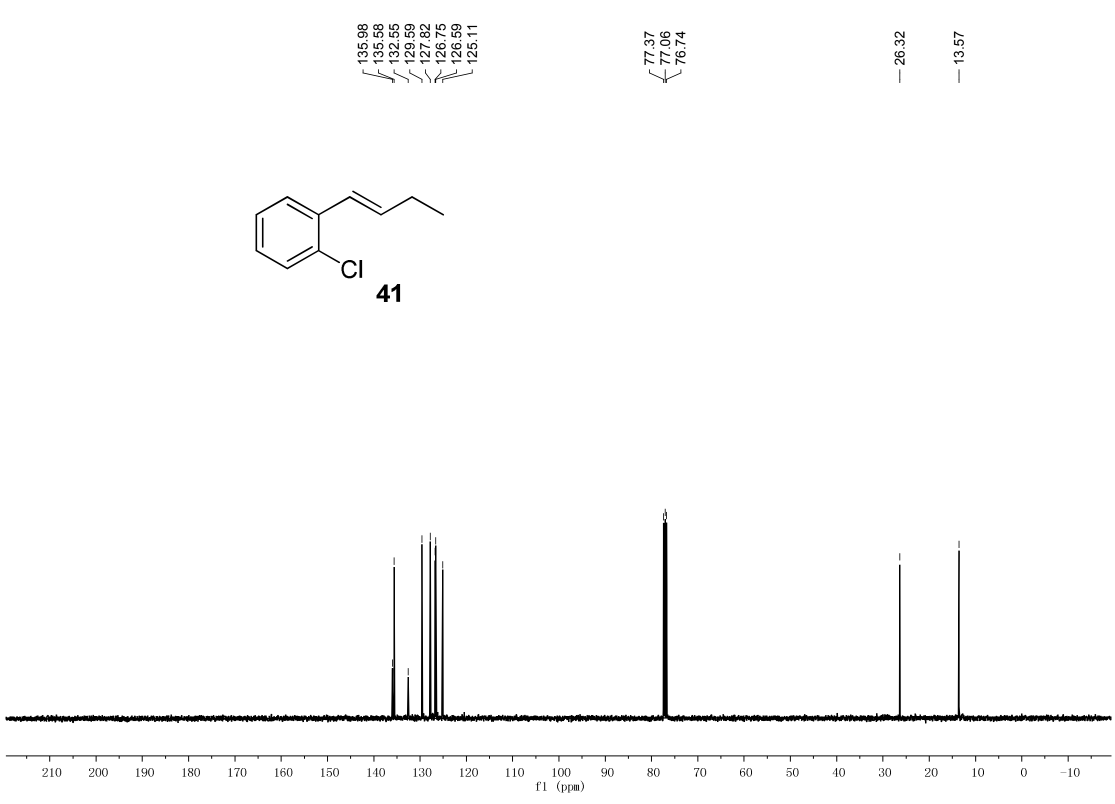
Cl
41
f1 (ppm)

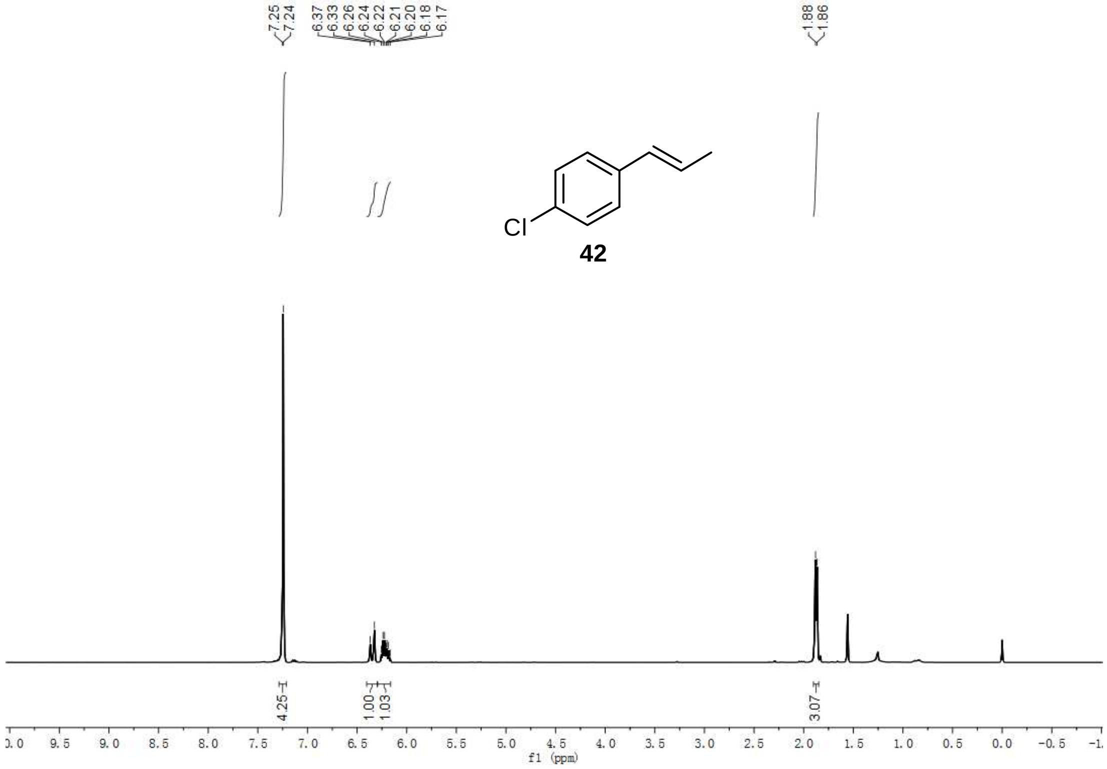
7.25
7.24
6.37
6.33
6.26
6.24
6.22
6.21
6.20
6.18
6.17
1.88
1.86
Cl
42
4.25
1.00
1.03
3.07
f1 (ppm)

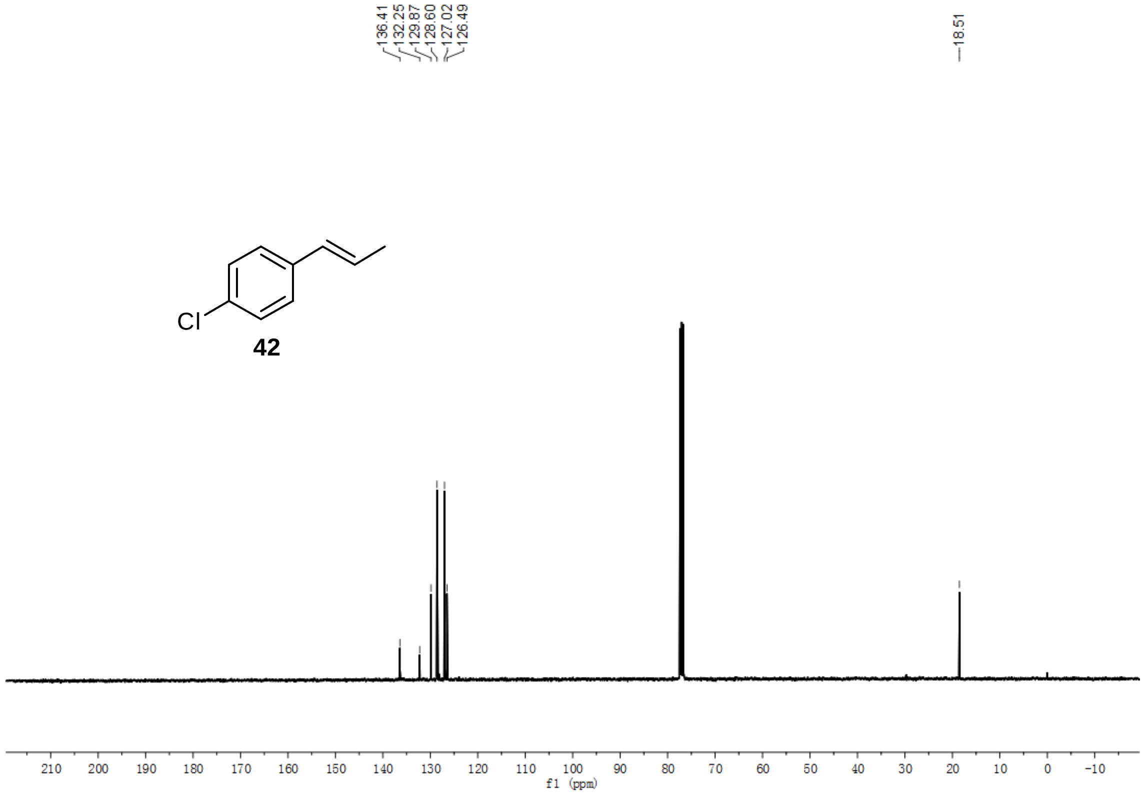
136.41
132.25
129.87
128.60
127.02
126.49
18.51
Cl
42
f1 (ppm)

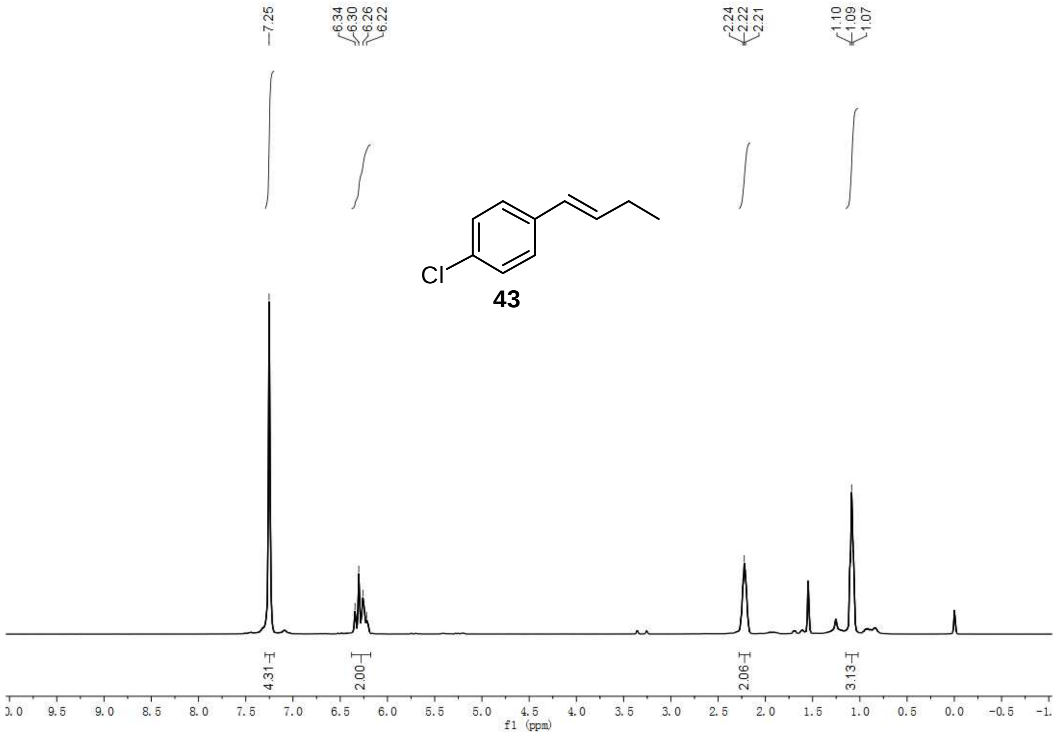
7.25
6.34
6.30
6.26
6.22
2.24
2.22
2.21
1.10
1.09
1.07
Cl
43
4.31
2.00
2.06
3.13
f1 (ppm)

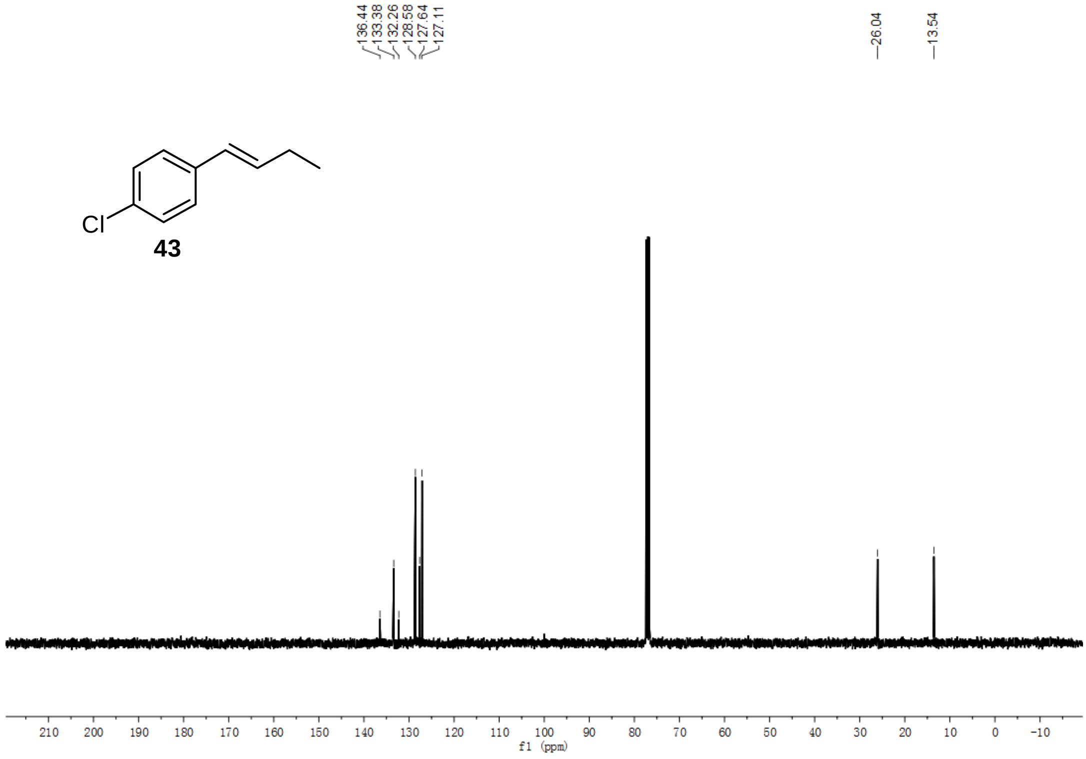
136.44
133.38
132.26
128.58
127.64
127.11
26.04
13.54
Cl
43
f1 (ppm)

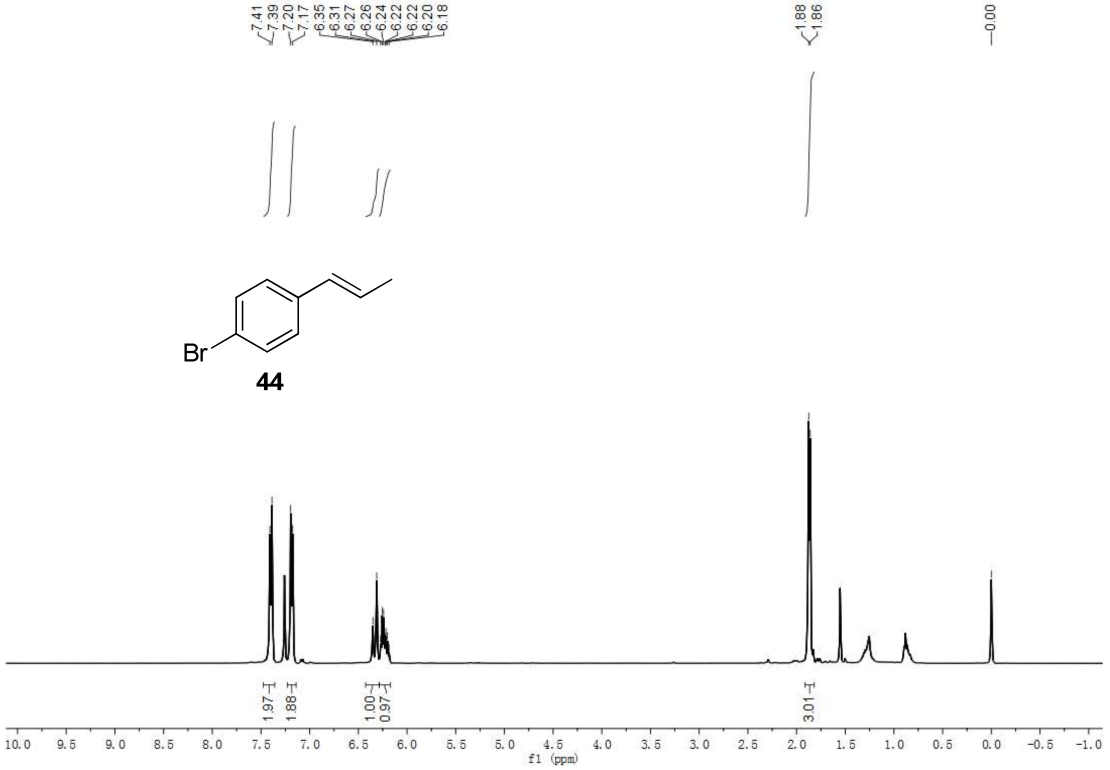
7.41
7.39
7.20
7.17
6.35
6.31
6.27
6.26
6.24
6.22
6.22
6.20
6.18
1.88
1.86
0.00
Br
44
1.97
1.88
1.00
0.97
3.01
10.0 9.5 9.0 8.5 8.0 7.5 7.0 6.5 6.0 5.5 5.0 4.5 4.0 3.5 3.0 2.5 2.0 1.5 1.0 0.5 0.0 -0.5 -1.0
f1 (ppm)

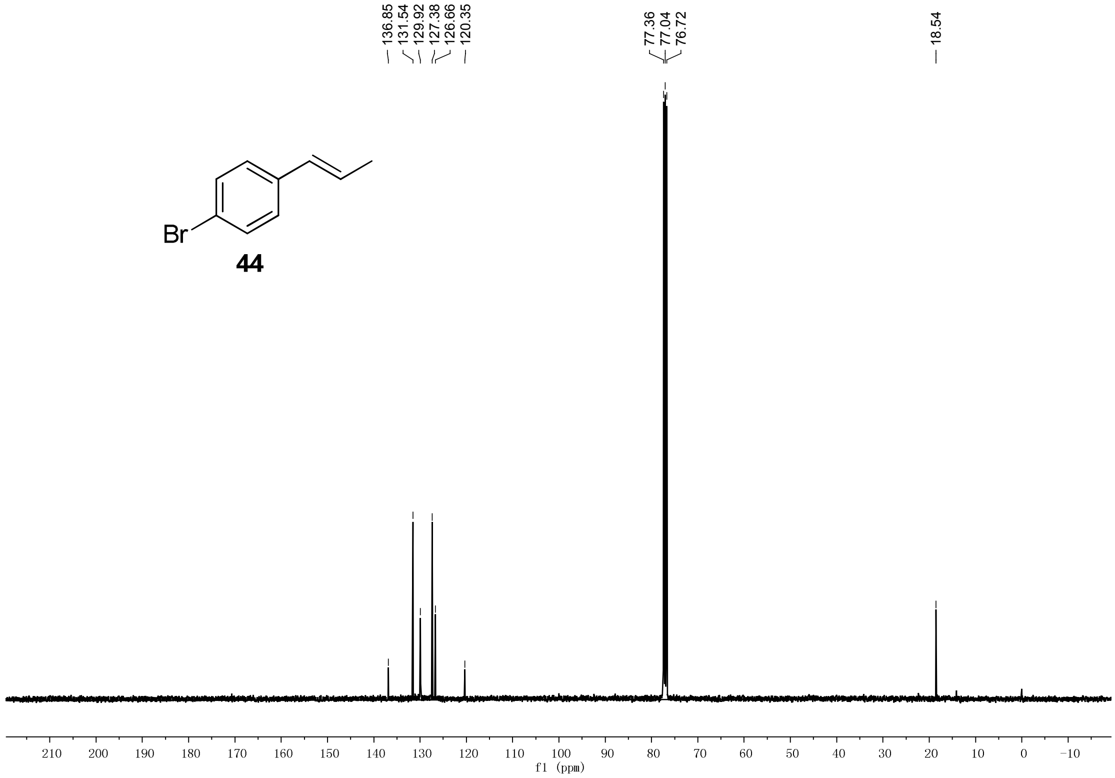
136.85
131.54
129.92
127.38
126.66
120.35
77.36
77.04
76.72
18.54
Br
44
210 200 190 180 170 160 150 140 130 120 110 100 90 80 70 60 50 40 30 20 10 0 -10
f1 (ppm)

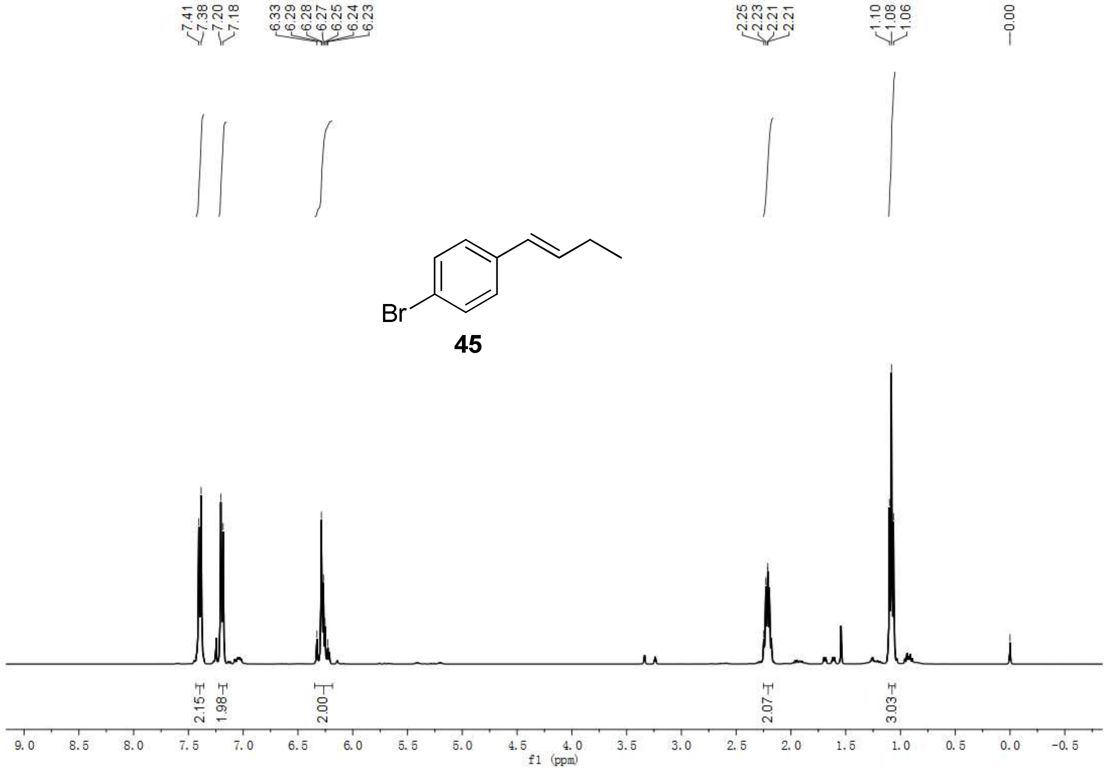
7.41
7.38
7.20
7.18
6.33
6.29
6.28
6.27
6.25
6.24
6.23
2.25
2.23
2.21
2.21
1.10
1.08
1.06
0.00
Br
2.15
1.98
2.00
2.07
3.03
f1 (ppm)

45

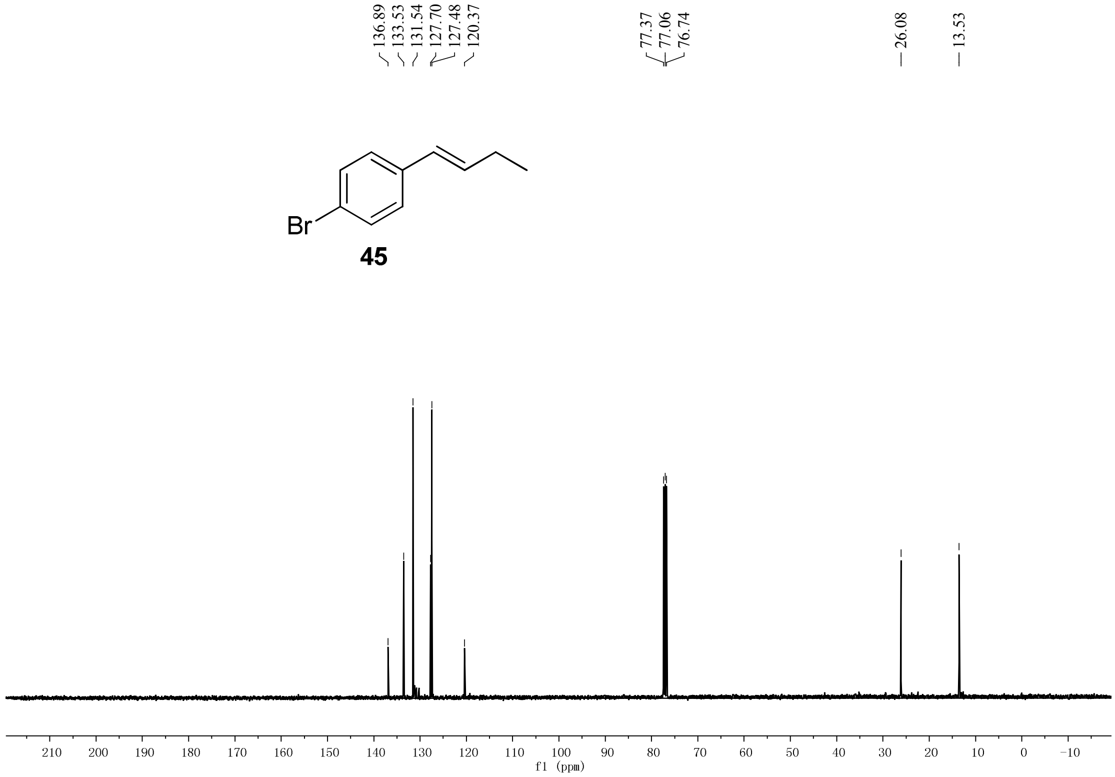
136.89
133.53
131.54
127.70
127.48
120.37
77.37
77.06
76.74
26.08
13.53
Br
f1 (ppm)

45

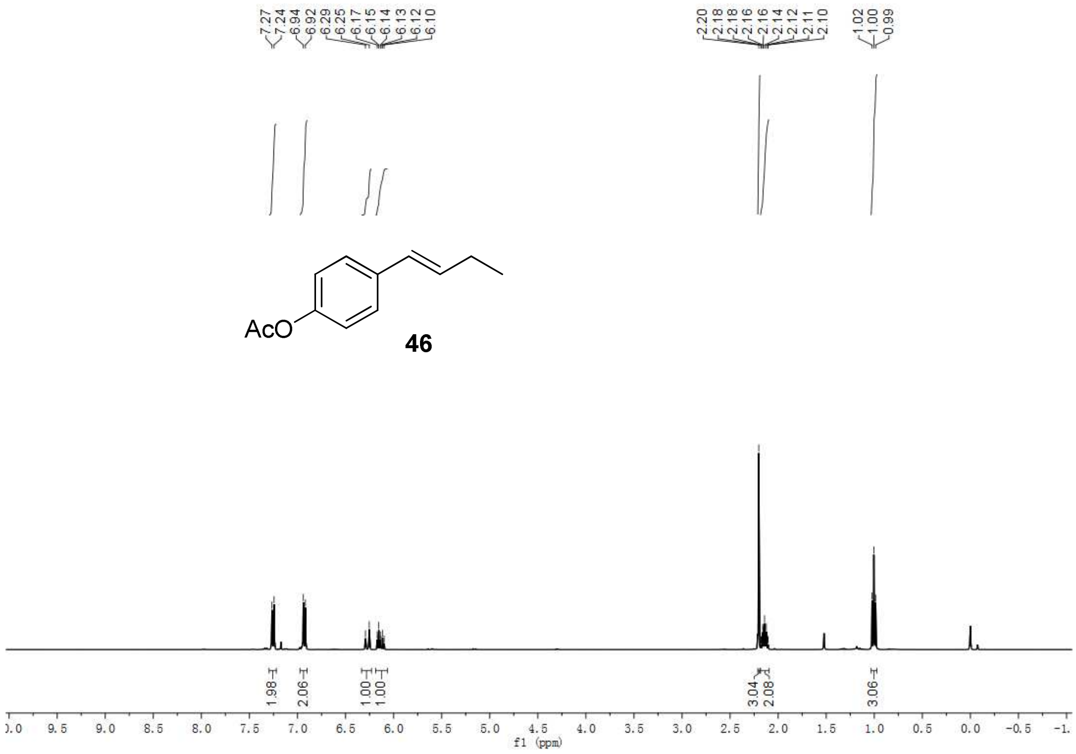
AcO
46
f1 (ppm)

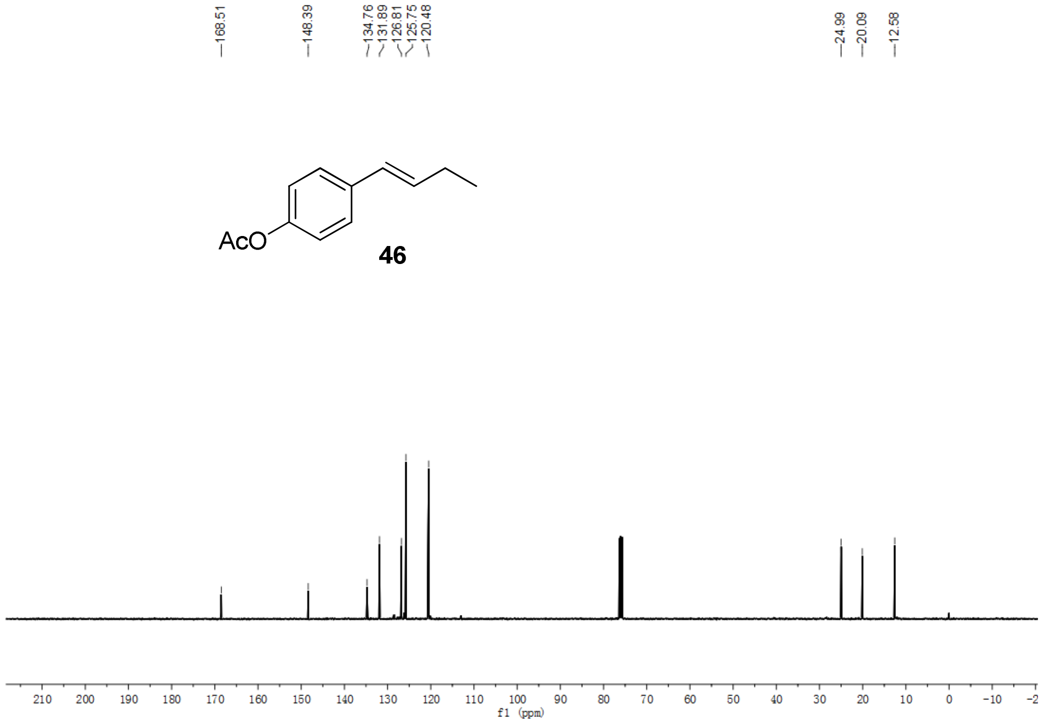
AcO
46
f1 (ppm)

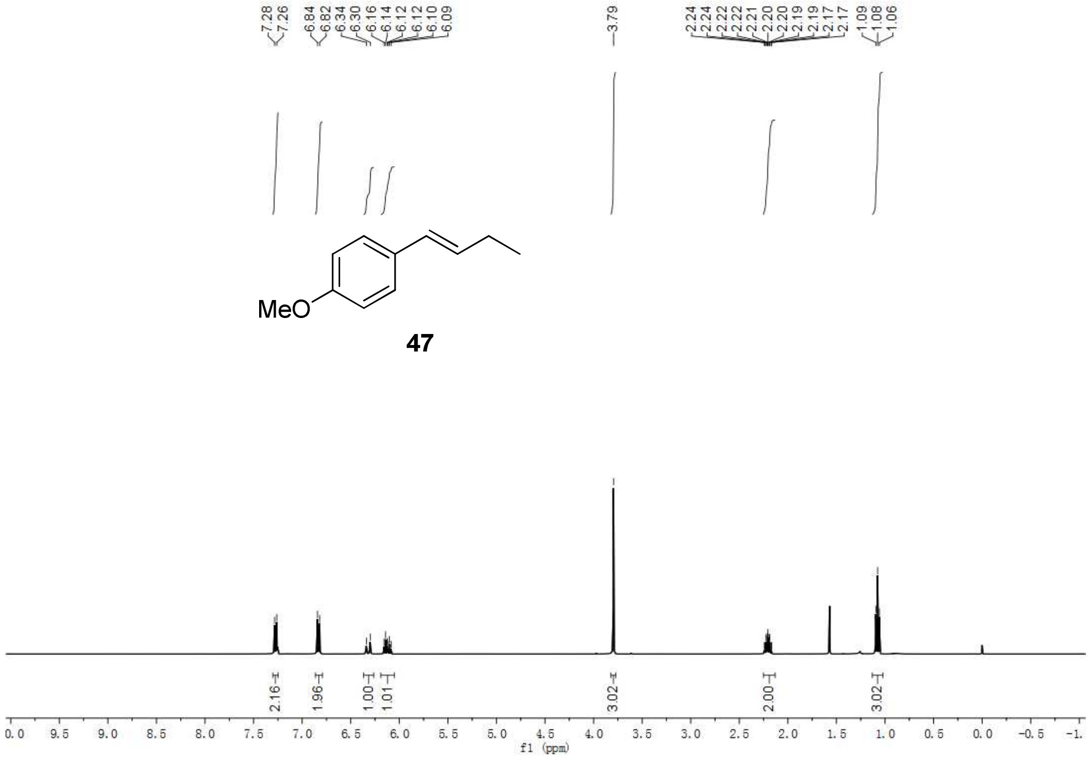
7.28
7.26
6.84
6.82
6.34
6.30
6.16
6.14
6.12
6.12
6.10
6.09
3.79
2.24
2.24
2.22
2.22
2.21
2.20
2.20
2.19
2.17
2.17
1.09
1.08
1.06
MeO
47
2.16
1.96
1.00
1.01
3.02
2.00
3.02
f1 (ppm)

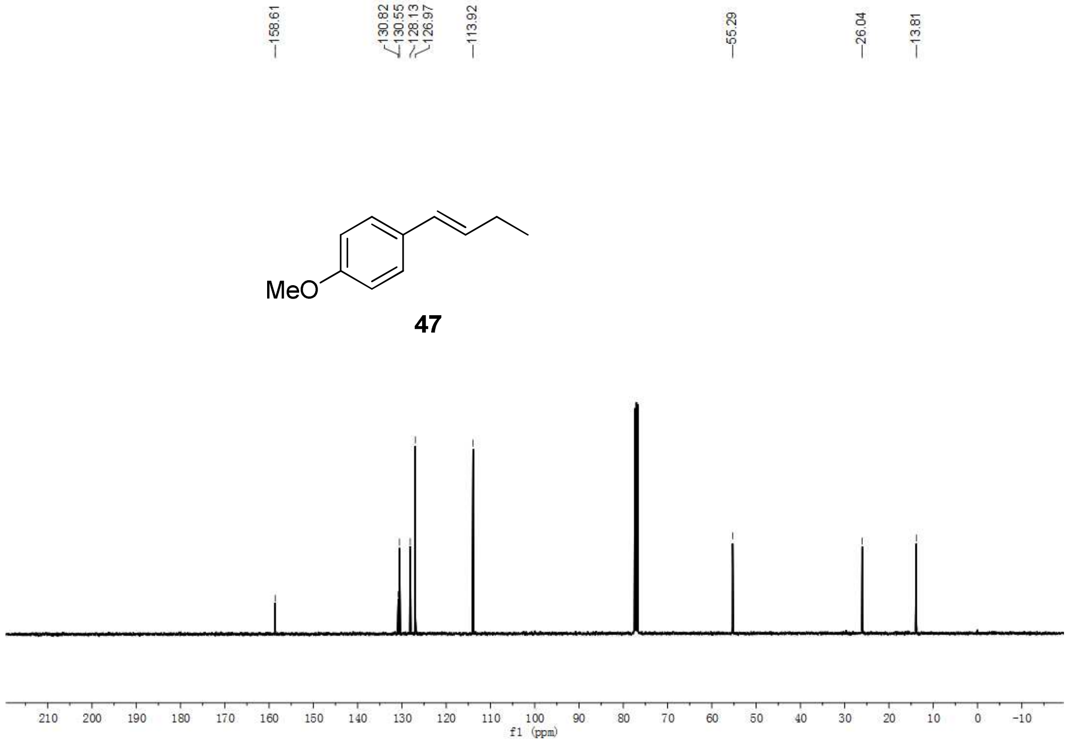
158.61
130.82
130.55
128.13
126.97
113.92
55.29
26.04
13.81
MeO
47
f1 (ppm)

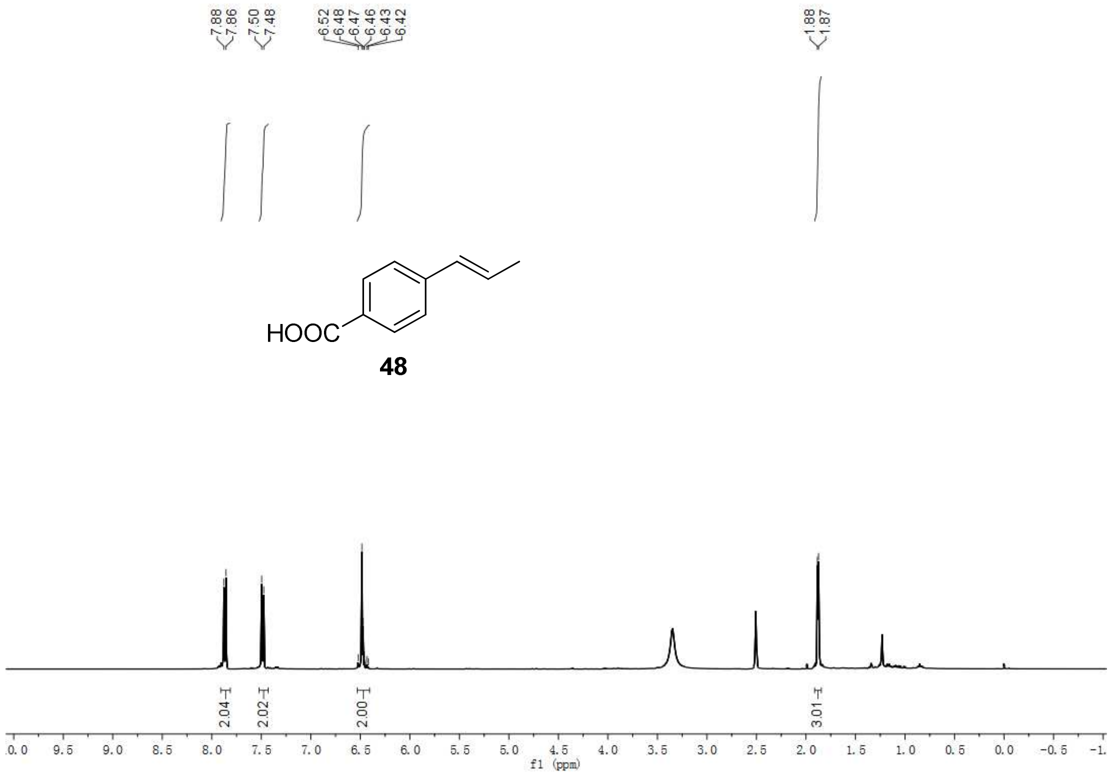
7.88
7.86
7.50
7.48
6.52
6.48
6.47
6.46
6.43
6.42
1.88
1.87
HOOC
48
2.04
2.02
2.00
3.01
f1 (ppm)

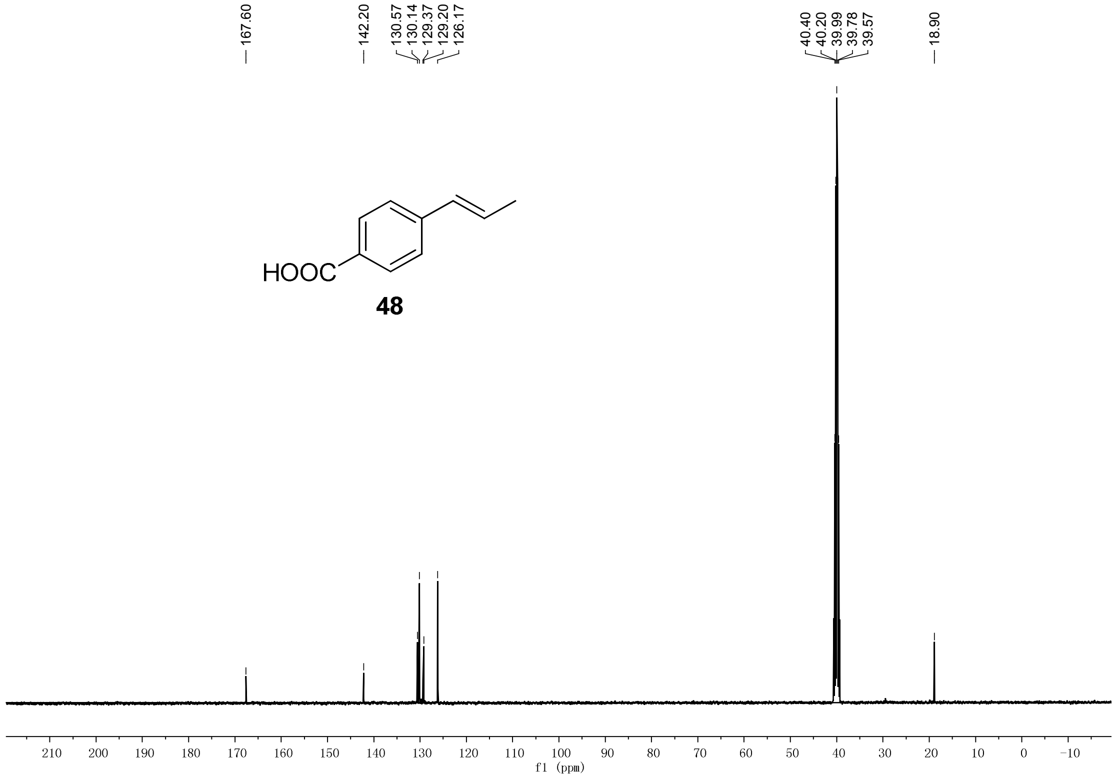
167.60
142.20
130.57
130.14
129.37
129.20
126.17
40.40
40.20
39.99
39.78
39.57
18.90
HOOC
48
f1 (ppm)

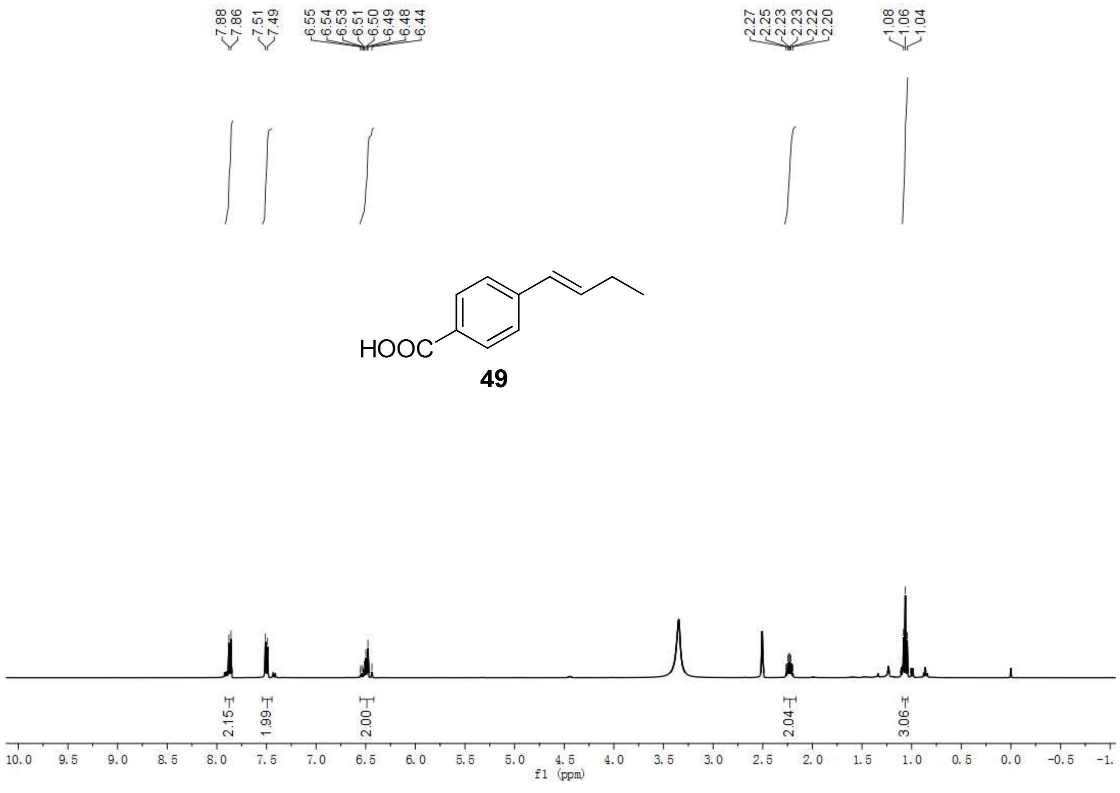
7.88
7.86
7.51
7.49
6.55
6.54
6.53
6.51
6.50
6.49
6.48
6.44
2.27
2.25
2.23
2.23
2.22
2.20
1.08
1.06
1.04
HOOC
49
2.15
1.99
2.00
2.04
3.06
10.0 9.5 9.0 8.5 8.0 7.5 7.0 6.5 6.0 5.5 5.0 4.5 4.0 3.5 3.0 2.5 2.0 1.5 1.0 0.5 0.0 -0.5
f1 (ppm)

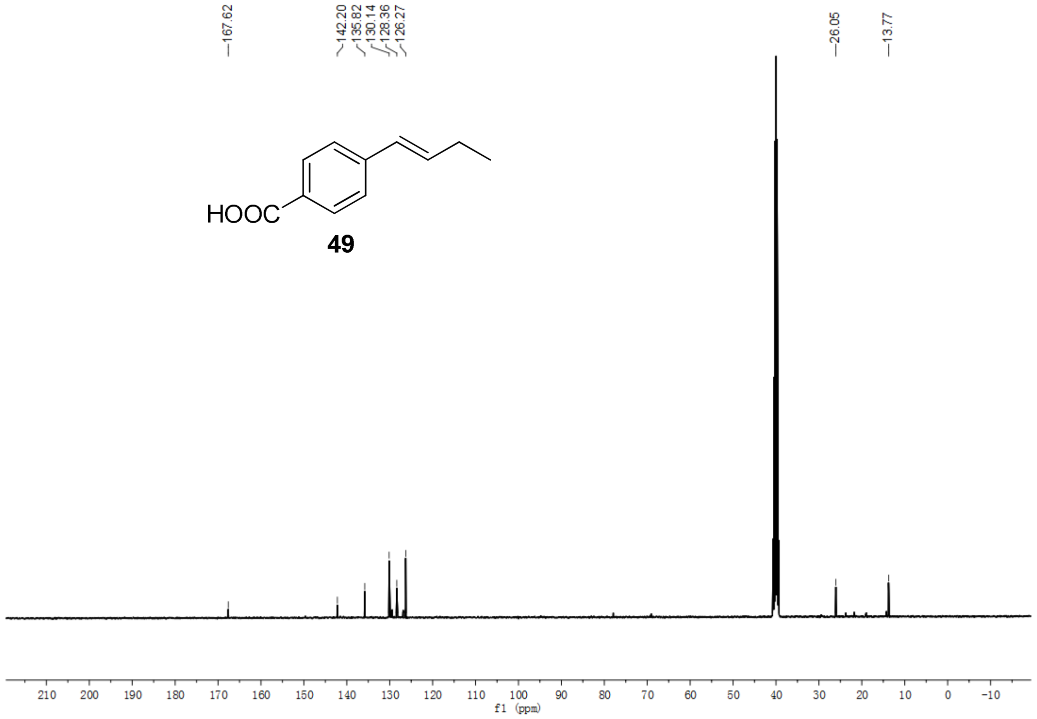
167.62
142.20
135.82
130.14
128.36
126.27
26.05
13.77
HOOC
49
210 200 190 180 170 160 150 140 130 120 110 100 90 80 70 60 50 40 30 20 10 0 -10
f1 (ppm)

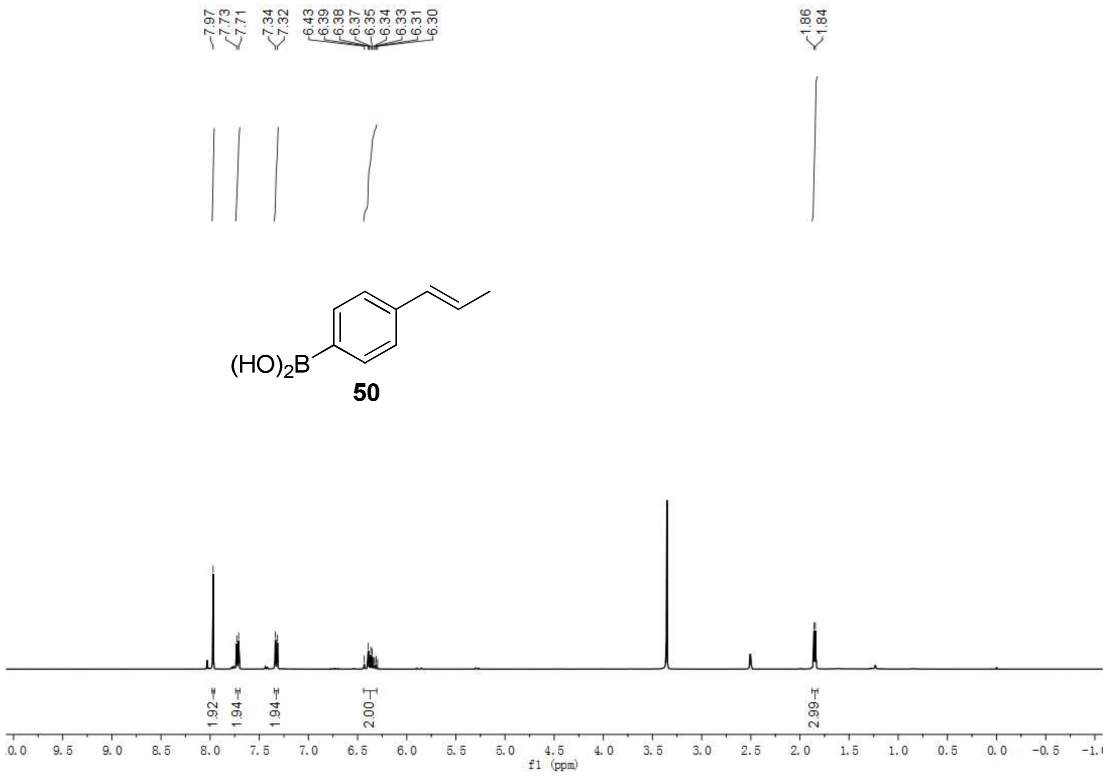
7.97
7.73
7.71
7.34
7.32
6.43
6.39
6.38
6.37
6.35
6.34
6.33
6.31
6.30
1.86
1.84
(HO)2B
50
1.92
1.94
1.94
2.00
2.99
f1 (ppm)

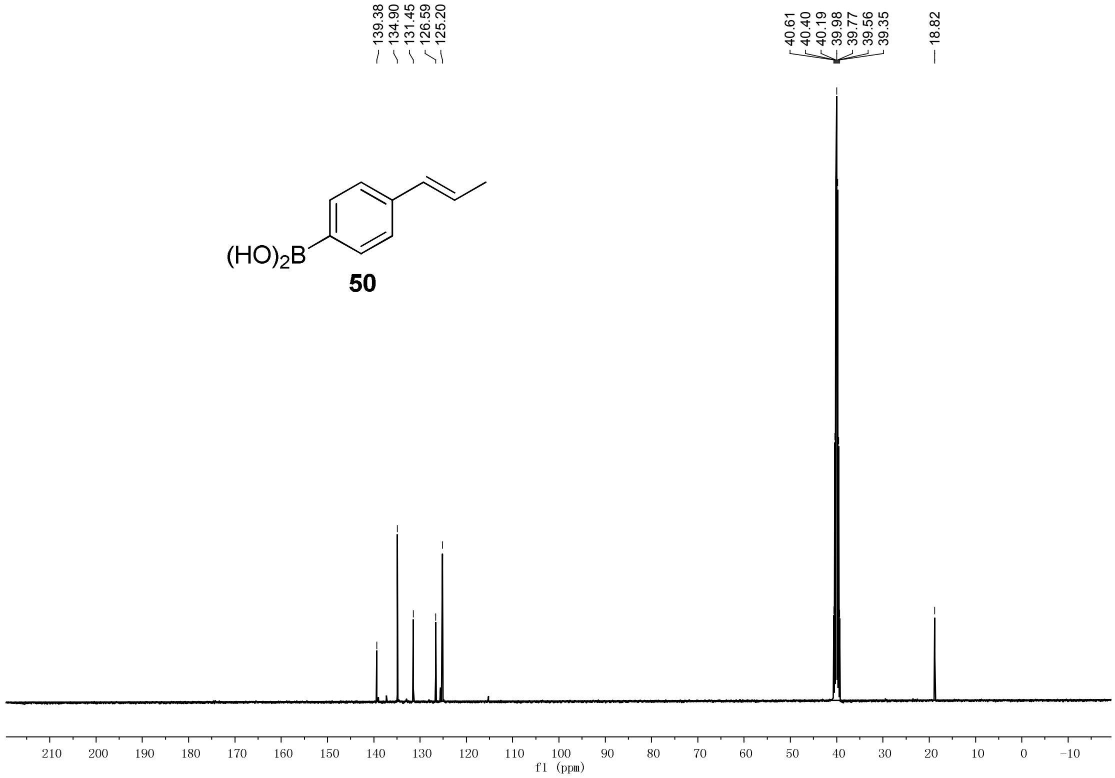
139.38
134.90
131.45
126.59
125.20
40.61
40.40
40.19
39.98
39.77
39.56
39.35
18.82
(HO)2B
50
f1 (ppm)

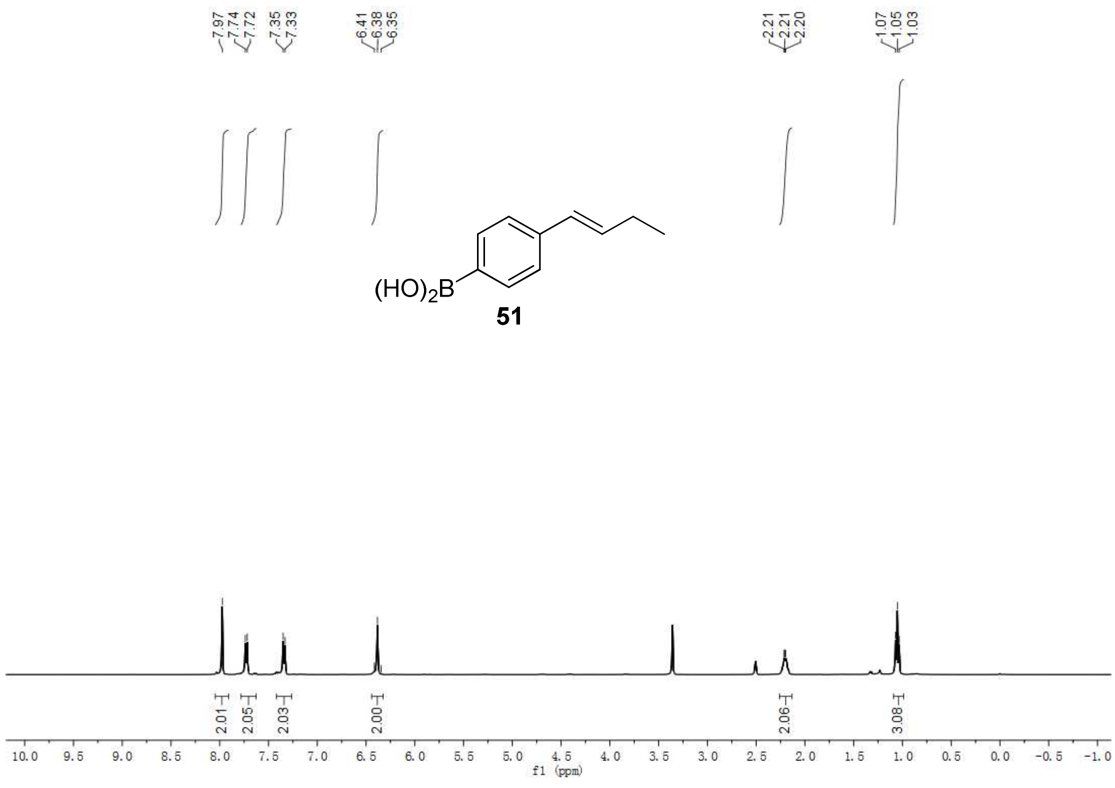

7.97
7.74
7.72
7.35
7.33
6.41
6.38
6.35
2.21
2.21
2.20
1.07
1.05
1.03
(HO)2B
51
2.01
2.05
2.03
2.00
2.06
3.08
f1 (ppm)

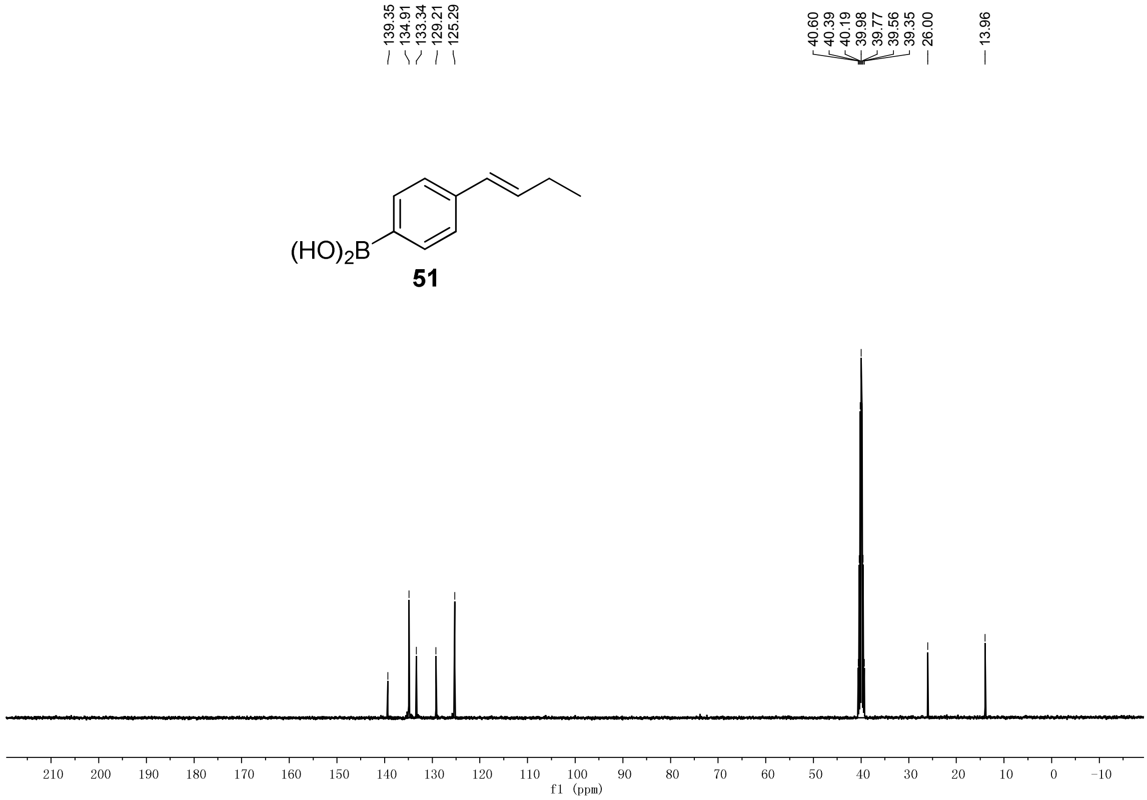

139.35
134.91
133.34
129.21
125.29
40.60
40.39
40.19
39.98
39.77
39.56
39.35
26.00
13.96
(HO)2B
51
f1 (ppm)

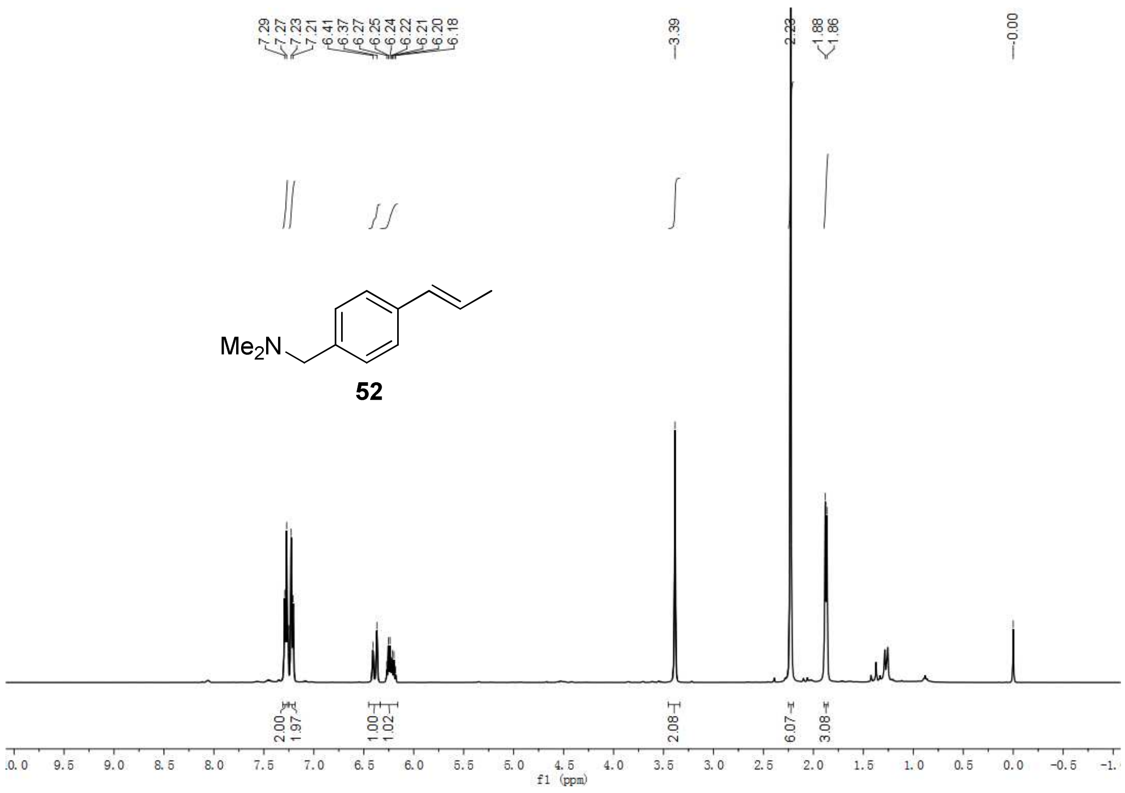
7.29
7.27
7.23
7.21
6.41
6.37
6.27
6.25
6.24
6.22
6.21
6.20
6.18
3.39
2.23
1.88
1.86
0.00
Me2N
52
2.00
1.97
1.00
1.02
2.08
6.07
3.08
f1 (ppm)

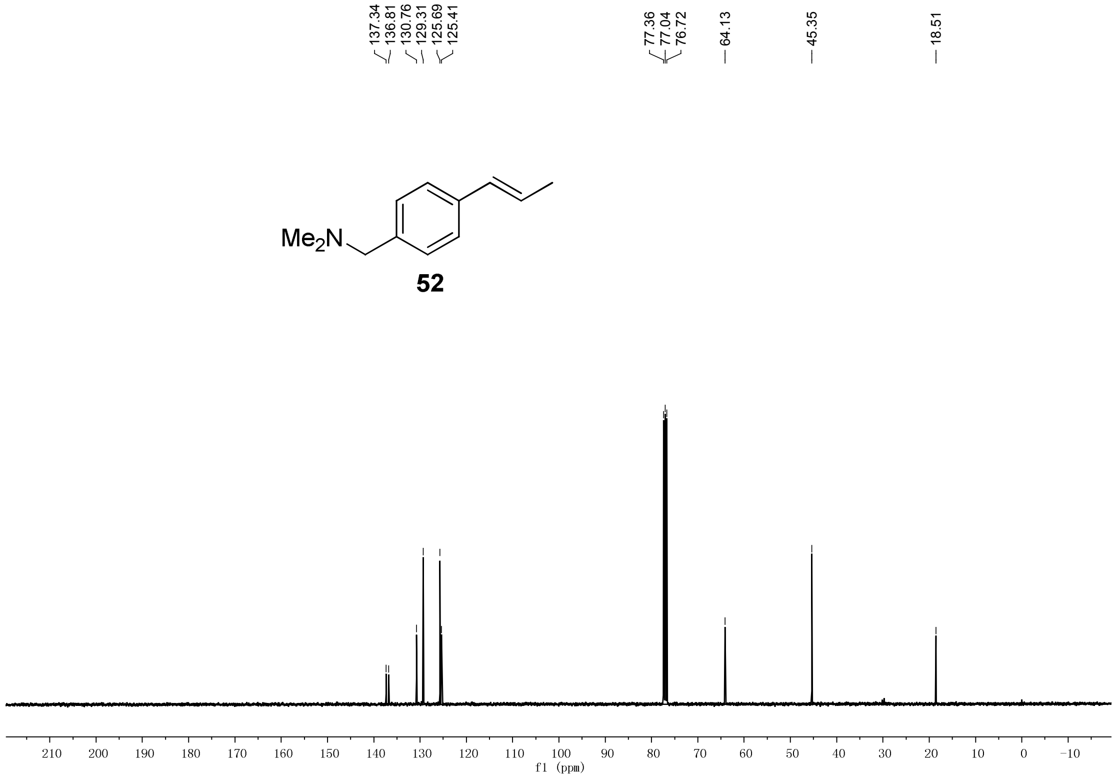
137.34
136.81
130.76
129.31
125.69
125.41
77.36
77.04
76.72
64.13
45.35
18.51
Me2N
52
f1 (ppm)

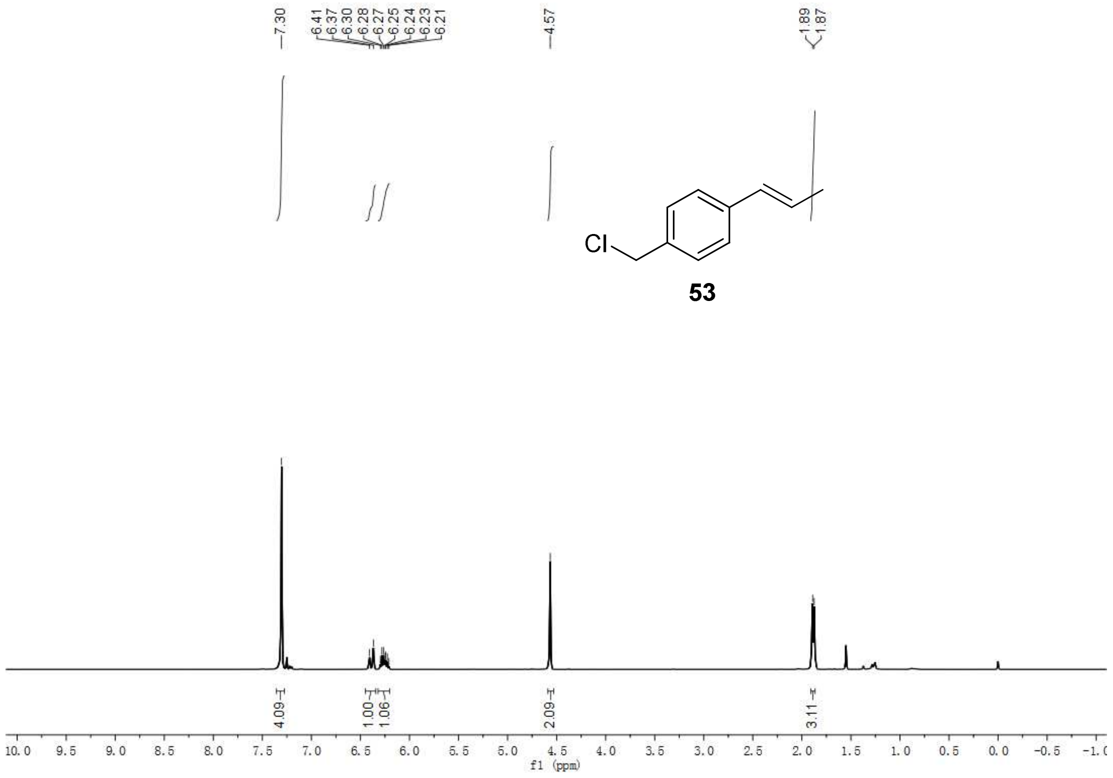
7.30
6.41
6.37
6.30
6.28
6.27
6.25
6.24
6.23
6.21
4.57
1.89
1.87
Cl
53
4.09
1.00
1.06
2.09
3.11
f1 (ppm)

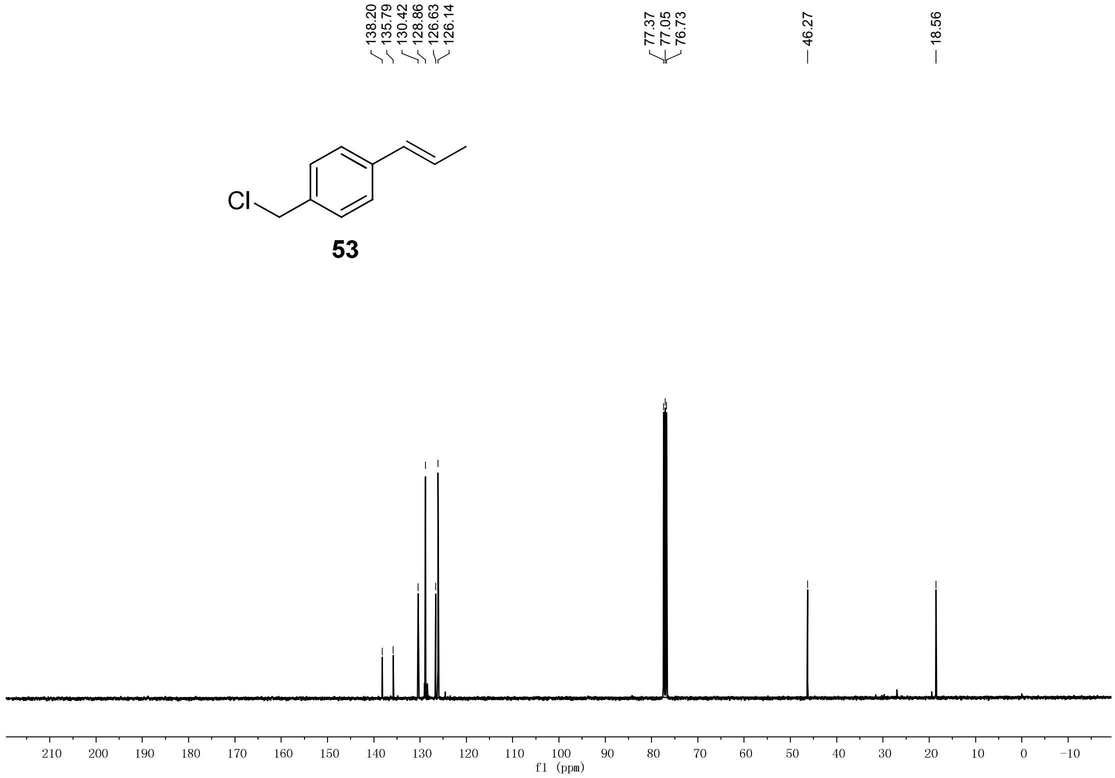
138.20
135.79
130.42
128.86
126.63
126.14
77.37
77.05
76.73
46.27
18.56
Cl
53
f1 (ppm)

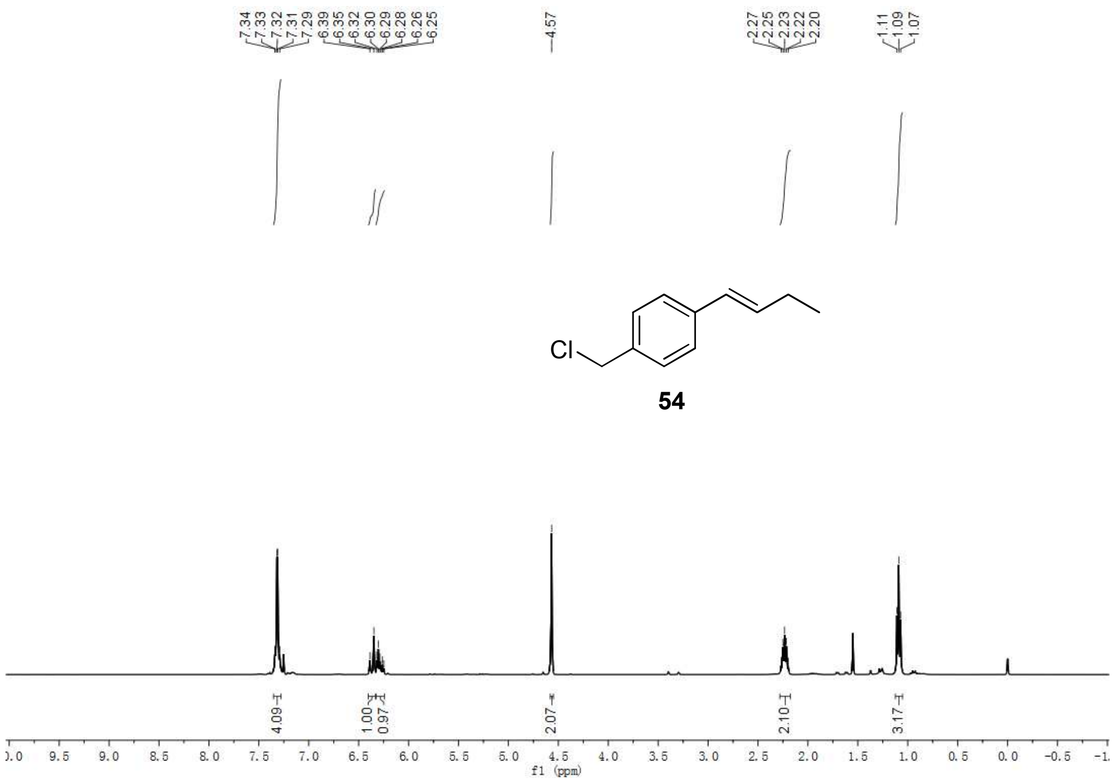
7.34
7.33
7.32
7.31
7.29
6.39
6.35
6.32
6.30
6.29
6.28
6.26
6.25
4.57
2.27
2.25
2.23
2.22
2.20
1.11
1.09
1.07
Cl
54
4.09
1.00
0.97
2.07
2.10
3.17
f1 (ppm)

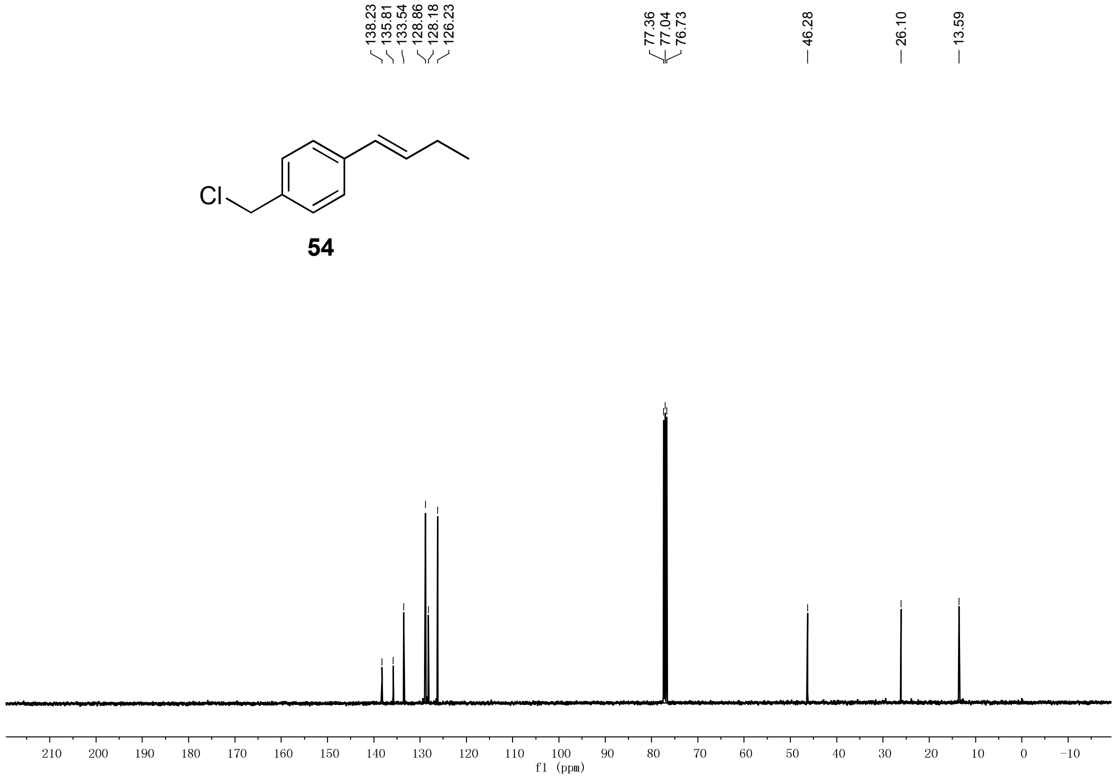
138.23
135.81
133.54
128.86
128.18
126.23
77.36
77.04
76.73
46.28
26.10
13.59
Cl
54
f1 (ppm)

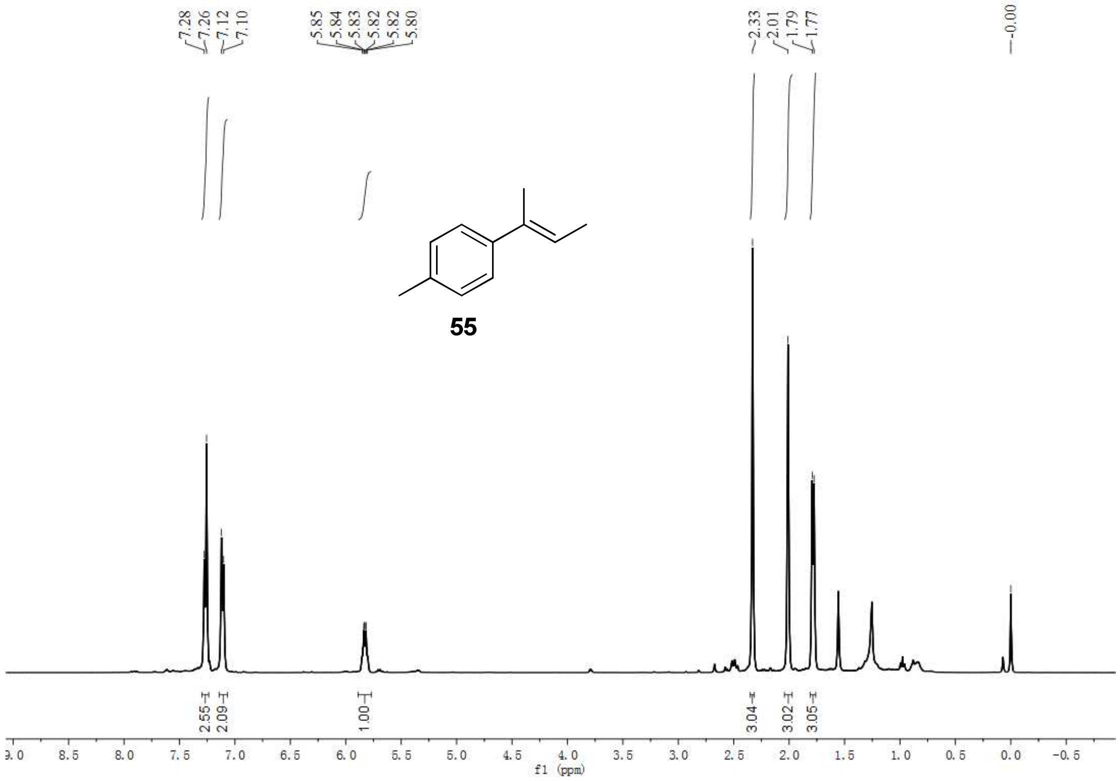
7.28
7.26
7.12
7.10
5.85
5.84
5.83
5.82
5.82
5.80
2.33
2.01
1.79
1.77
-0.00
55
2.55
2.09
1.00
3.04
3.02
3.05
f1 (ppm)

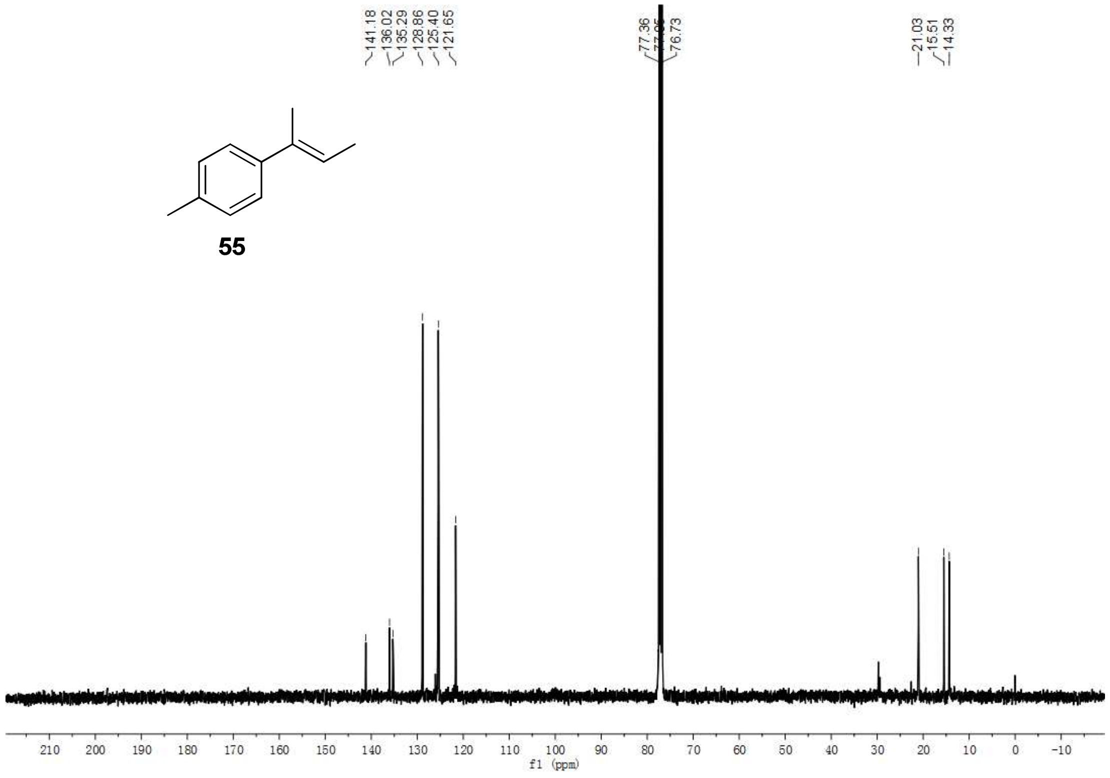
141.18
136.02
135.29
128.86
125.40
121.65
77.36
76.73
21.03
15.51
14.33
55
f1 (ppm)

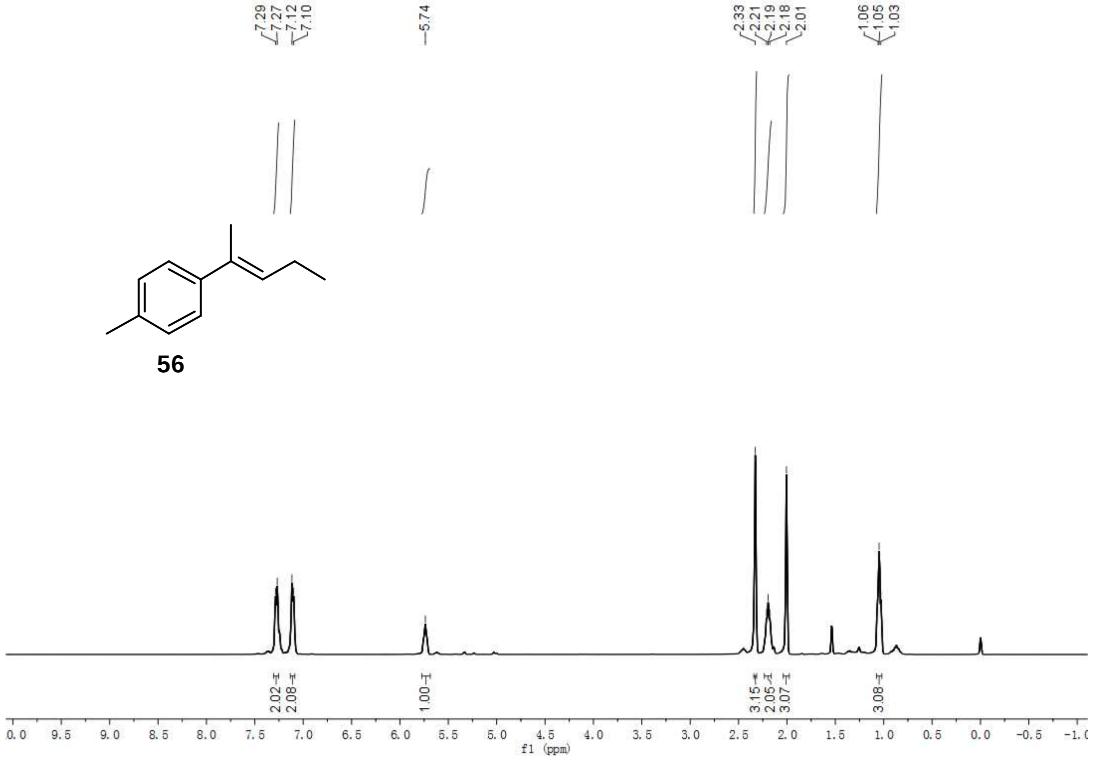
7.29
7.27
7.12
7.10
5.74
2.33
2.21
2.19
2.18
2.01
1.06
1.05
1.03
56
2.02
2.08
1.00
3.15
2.05
3.07
3.08
f1 (ppm)

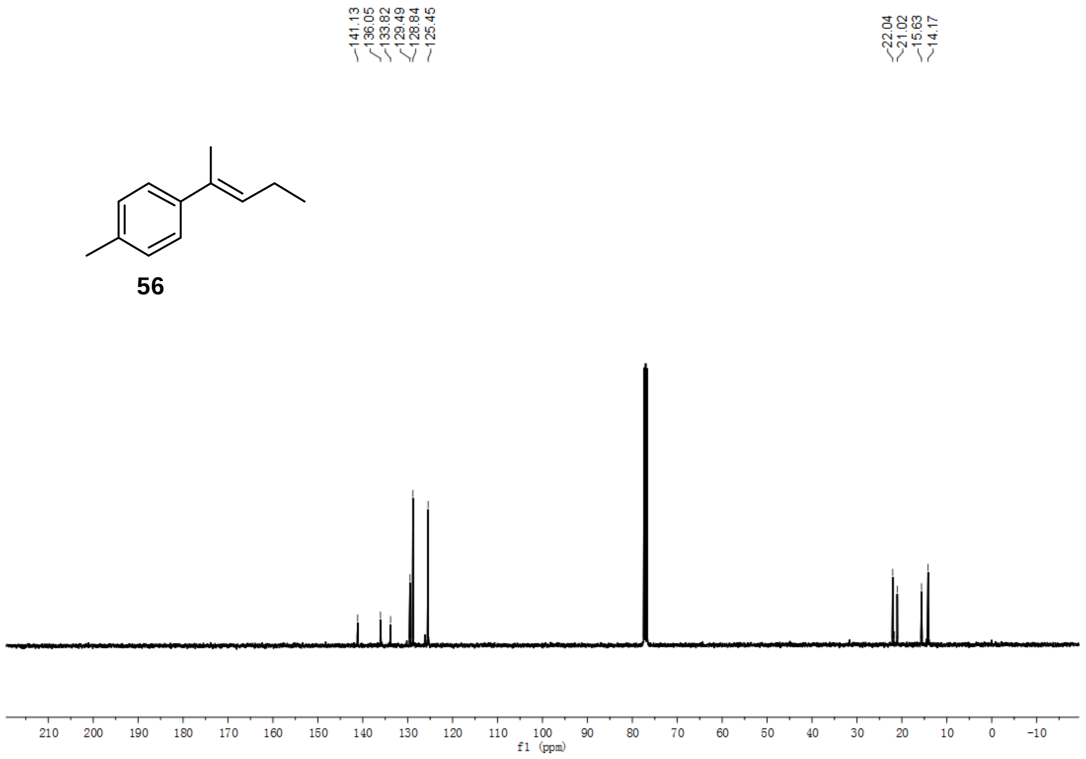
141.13
136.05
133.82
129.49
128.84
125.45
22.04
21.02
15.63
14.17
56
f1 (ppm)

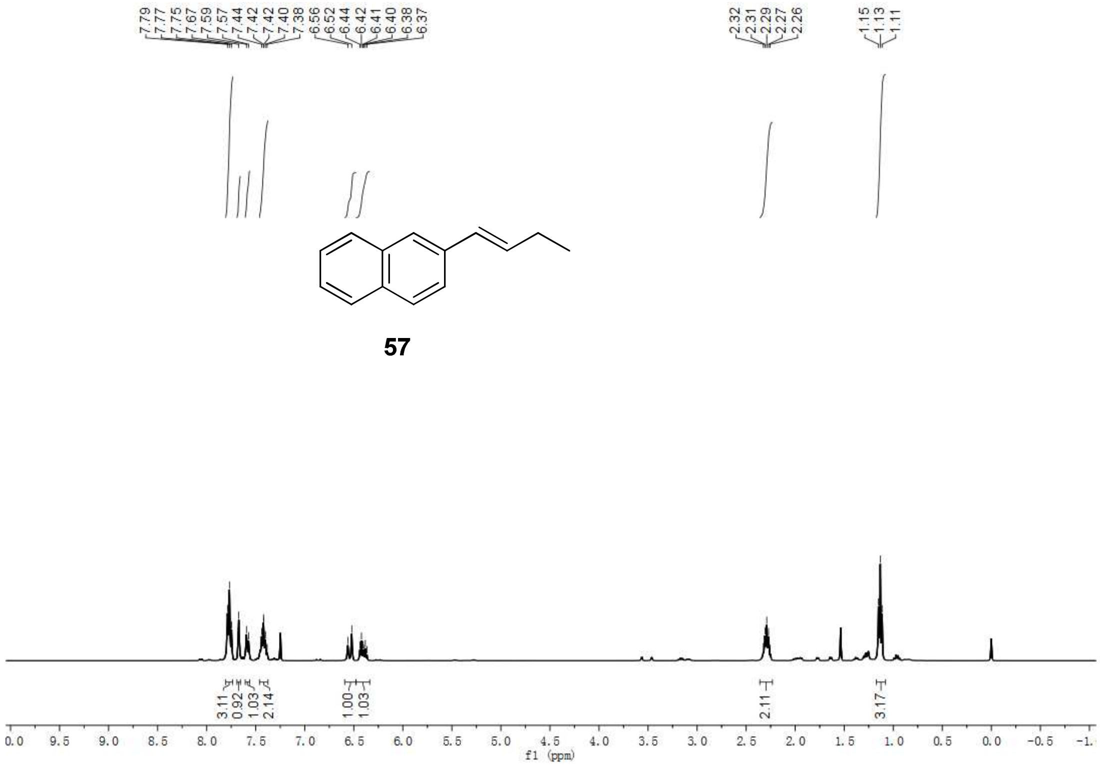
7.79
7.77
7.75
7.67
7.59
7.57
7.44
7.42
7.42
7.40
7.38
6.56
6.52
6.44
6.42
6.41
6.40
6.38
6.37
2.32
2.31
2.29
2.27
2.26
1.15
1.13
1.11
57
3.11
0.92
1.03
2.14
1.00
1.03
2.11
3.17
f1 (ppm)

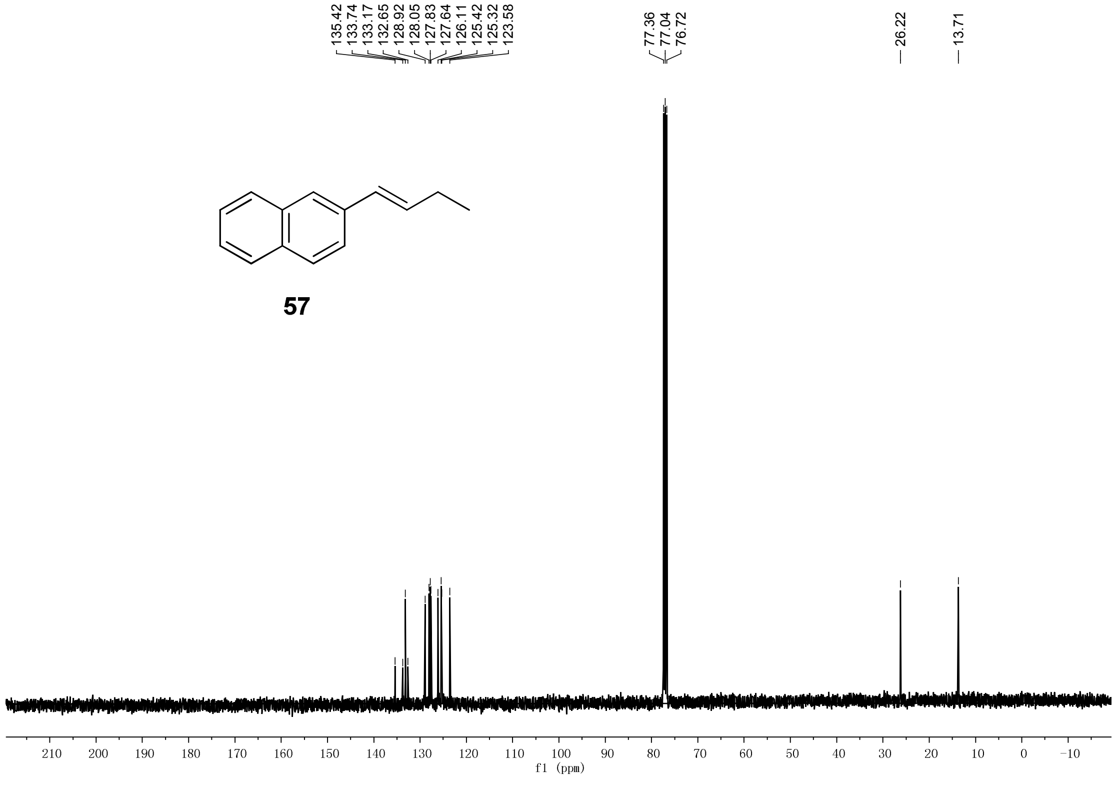
135.42
133.74
133.17
132.65
128.92
128.05
127.83
127.64
126.11
125.42
125.32
123.58
77.36
77.04
76.72
26.22
13.71
57
f1 (ppm)

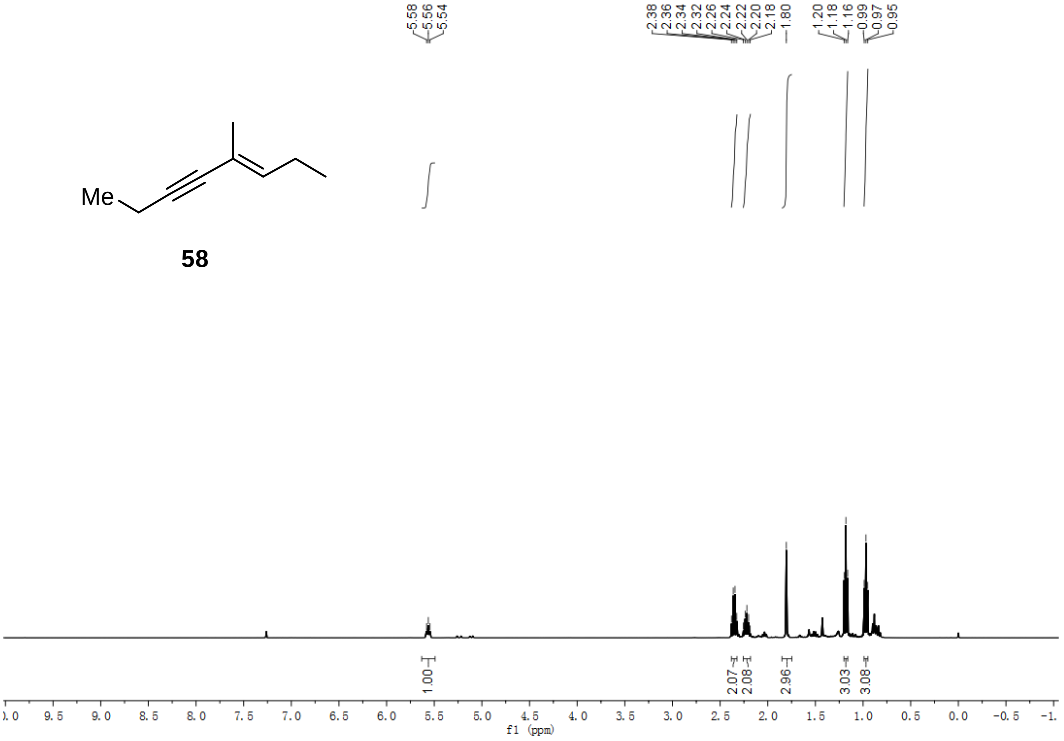
Me
5.58
5.56
5.54
2.38
2.36
2.34
2.32
2.26
2.24
2.22
2.20
2.18
1.80
1.20
1.18
1.16
0.99
0.97
0.95
1.00
2.07
2.08
2.96
3.03
3.08
f1 (ppm)

58

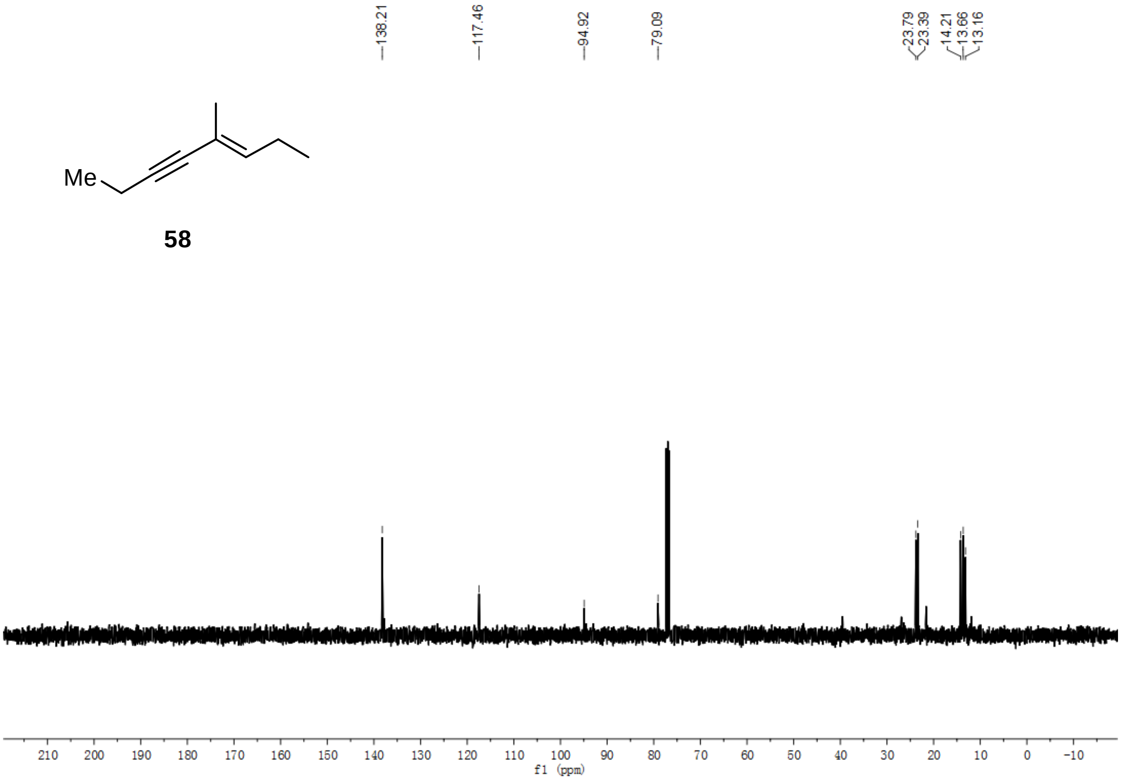
Me
138.21
117.46
94.92
79.09
23.79
23.39
14.21
13.66
13.16
f1 (ppm)

58

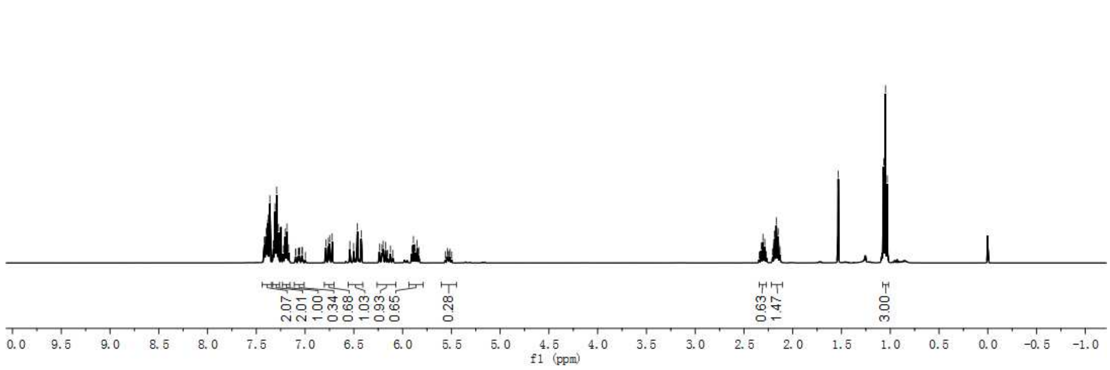

7.42 7.40 7.38 7.36 7.33 7.32 7.31 7.29 7.27 7.22 7.20 7.19 7.17 7.10 7.07 7.06 7.03 7.03 6.79 6.76 6.75 6.72 6.54 6.50 6.46 6.42 6.24 6.21 6.20 6.17 6.13 6.10 5.91 5.89 5.87 5.85 5.83 5.54 5.51 2.34 2.32 2.31 2.29 2.27 2.21 2.19 2.17 2.15 2.13 1.53 1.07 1.05 1.03
2.07 2.01 1.00 0.34 0.68 1.03 0.93 0.65 0.28 0.63 1.47 3.00
f1 (ppm)

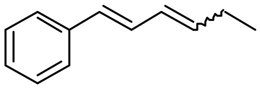

59

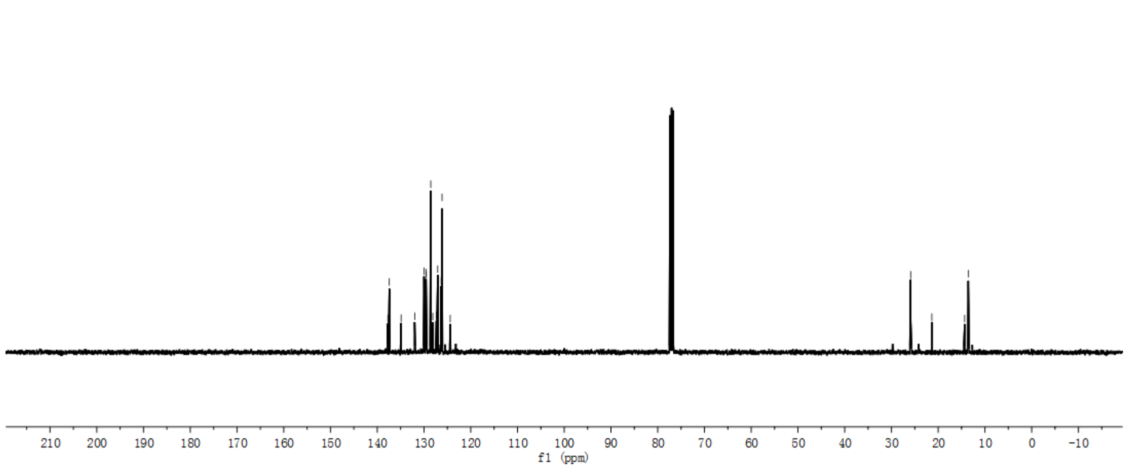

137.72 137.42 134.89 131.98 129.99 129.57 129.50 128.59 128.55 128.11 127.34 127.07 126.33 126.14 124.39
25.89 21.36 14.34 13.55
f1 (ppm)

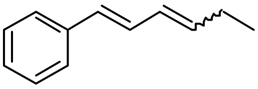

59

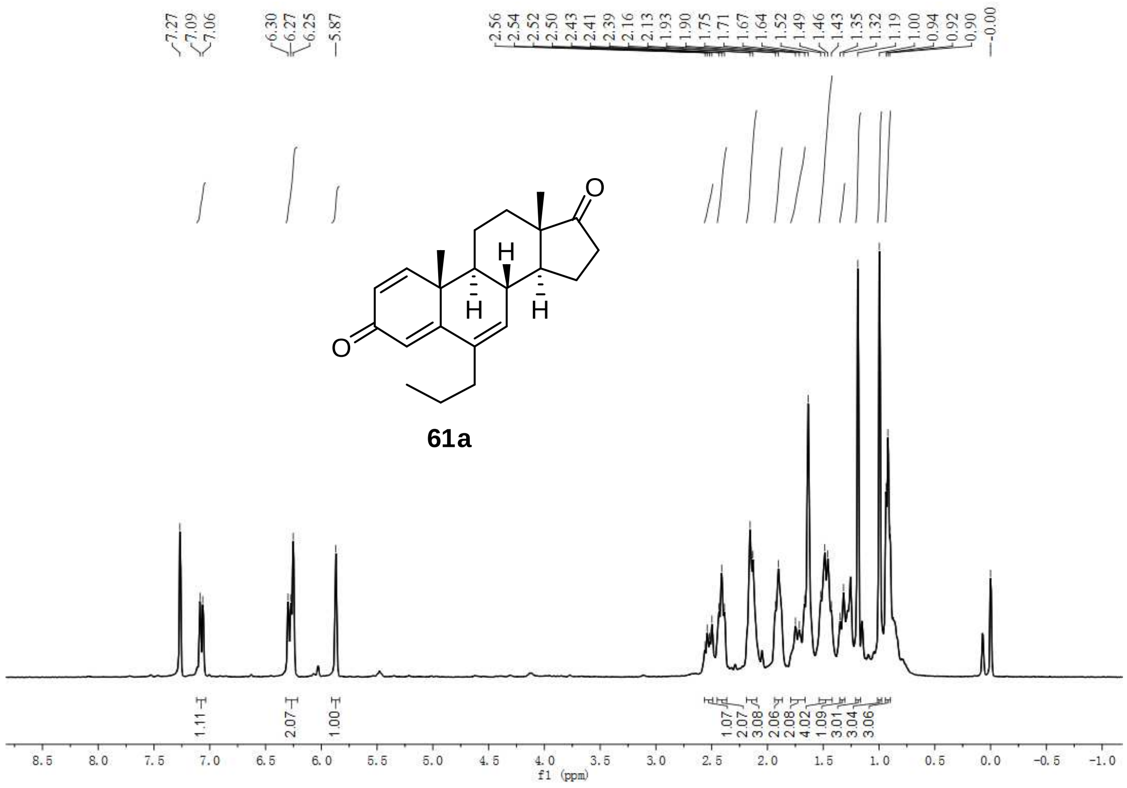
61a
f1 (ppm)

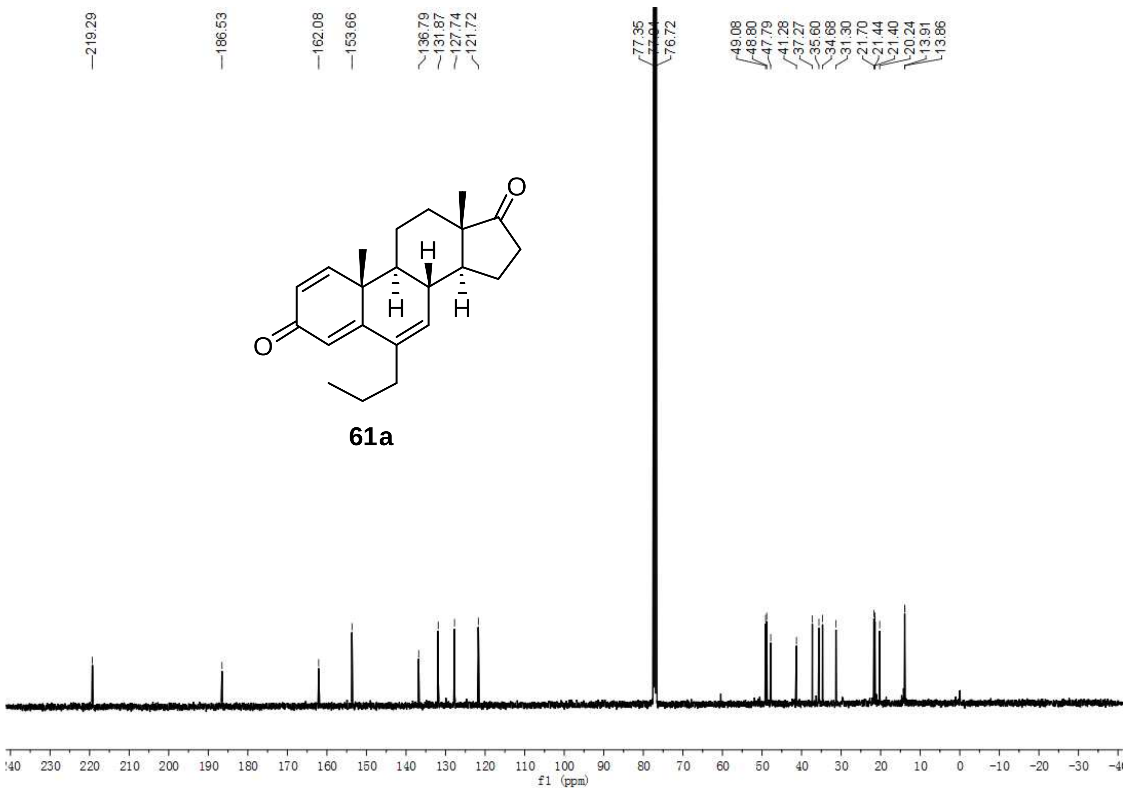
61a
f1 (ppm)

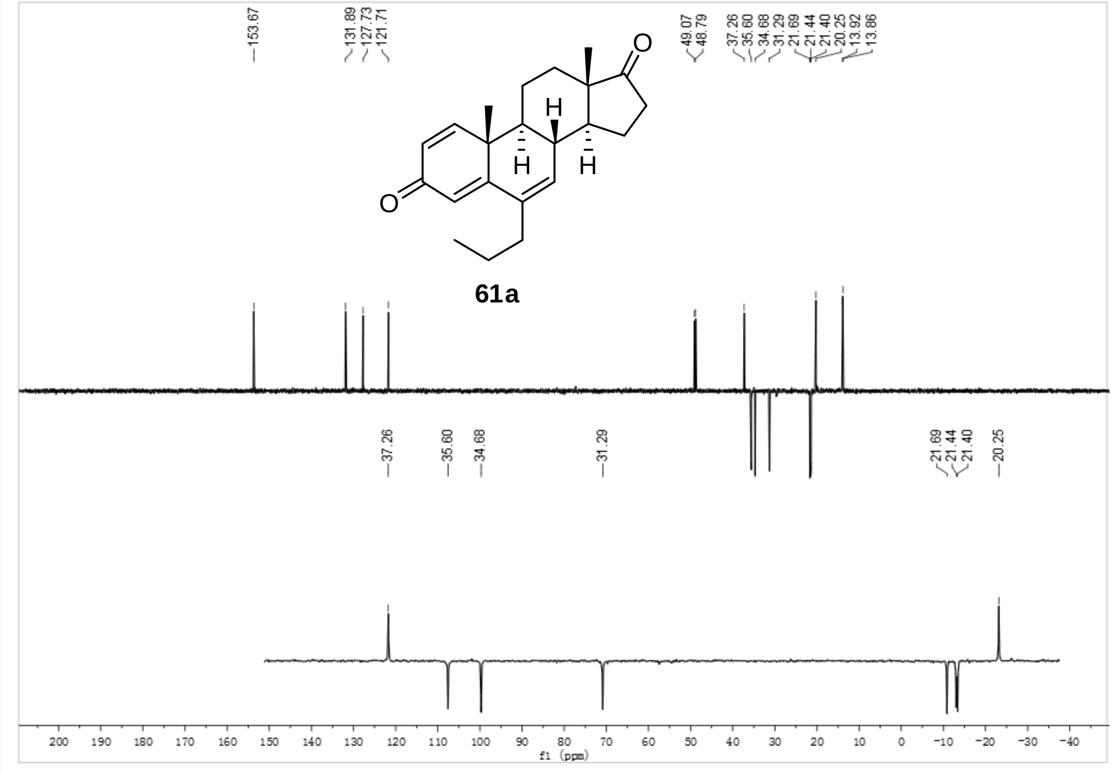

153.67
131.89
127.73
121.71
49.07
48.79
37.26
35.60
34.68
31.29
21.69
21.44
21.40
20.25
13.92
13.86
61a
37.26
35.60
34.68
31.29
21.69
21.44
21.40
20.25
f1 (ppm)

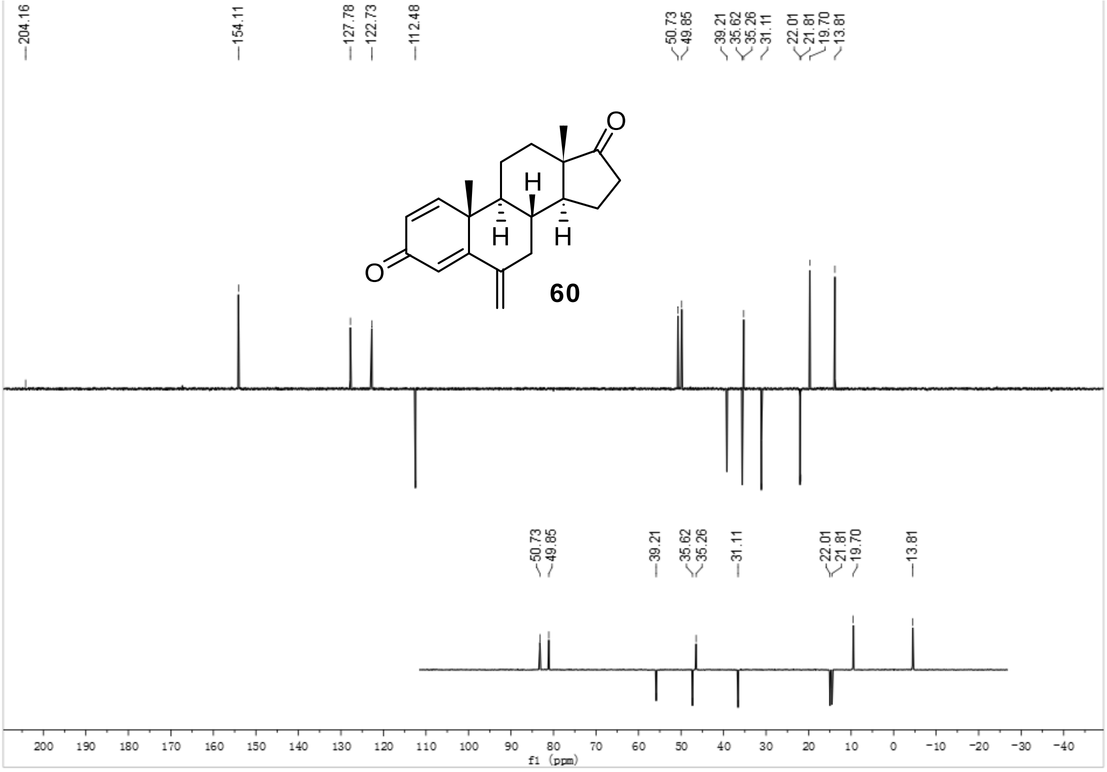

204.16
154.11
127.78
122.73
112.48
50.73
49.85
39.21
35.62
35.26
31.11
22.01
21.81
19.70
13.81
60
50.73
49.85
39.21
35.62
35.26
31.11
22.01
21.81
19.70
13.81
f1 (ppm)

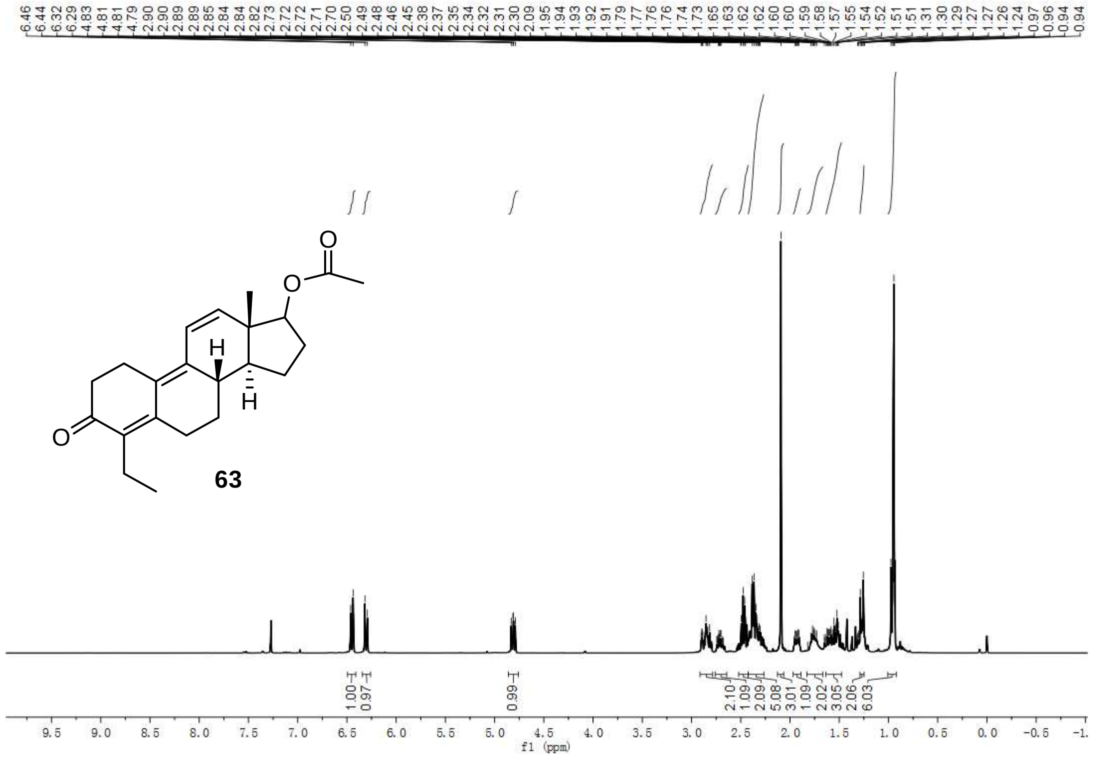

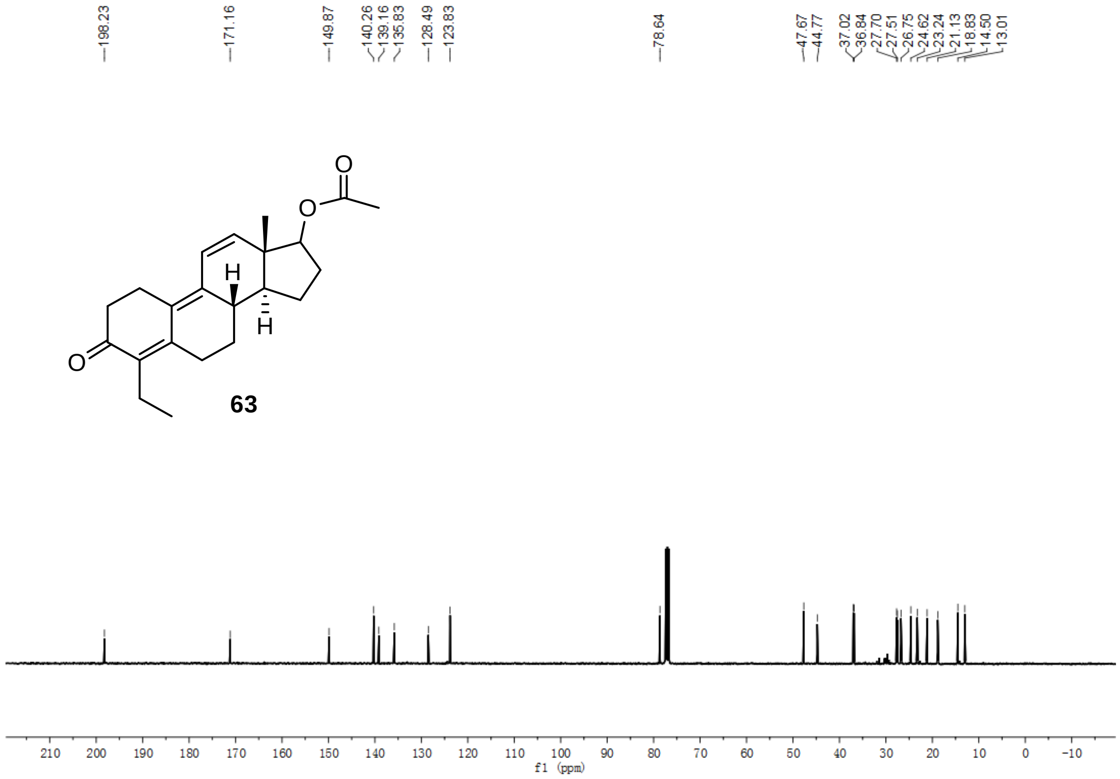

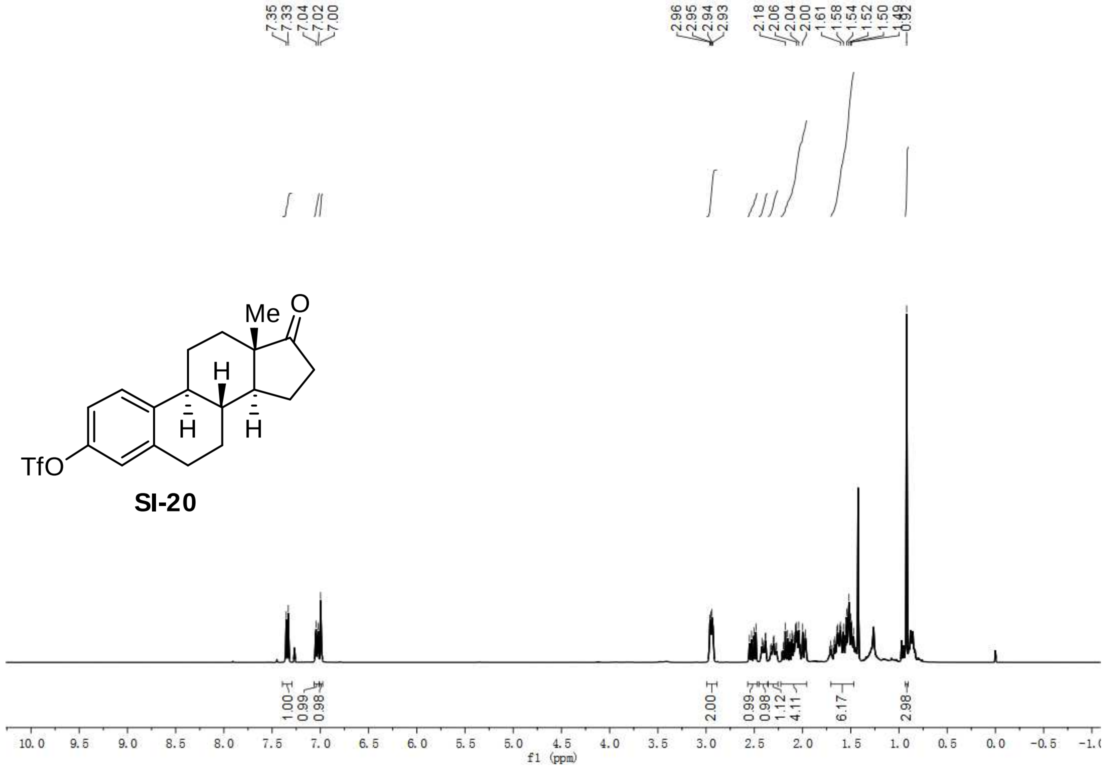
7.35
7.33
7.04
7.02
7.00
2.96
2.95
2.94
2.93
2.18
2.06
2.04
2.00
1.61
1.58
1.54
1.52
1.50
1.49
0.92
Me
O
H
H
H
TfO
SI-20
1.00
0.99
0.98
2.00
0.99
0.98
1.12
4.11
6.17
2.98
f1 (ppm)

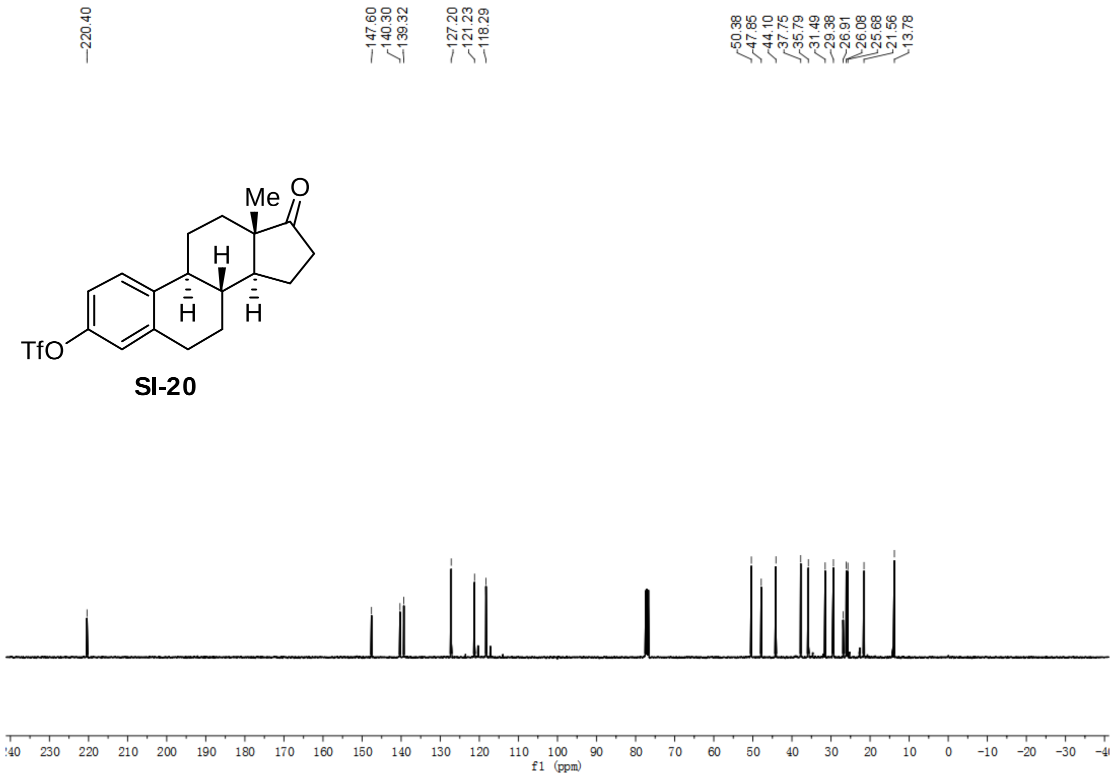
220.40
147.60
140.30
139.32
127.20
121.23
118.29
50.38
47.85
44.10
37.75
35.79
31.49
29.38
26.91
26.08
25.68
21.56
13.78
Me
O
H
H
H
TfO
SI-20
f1 (ppm)

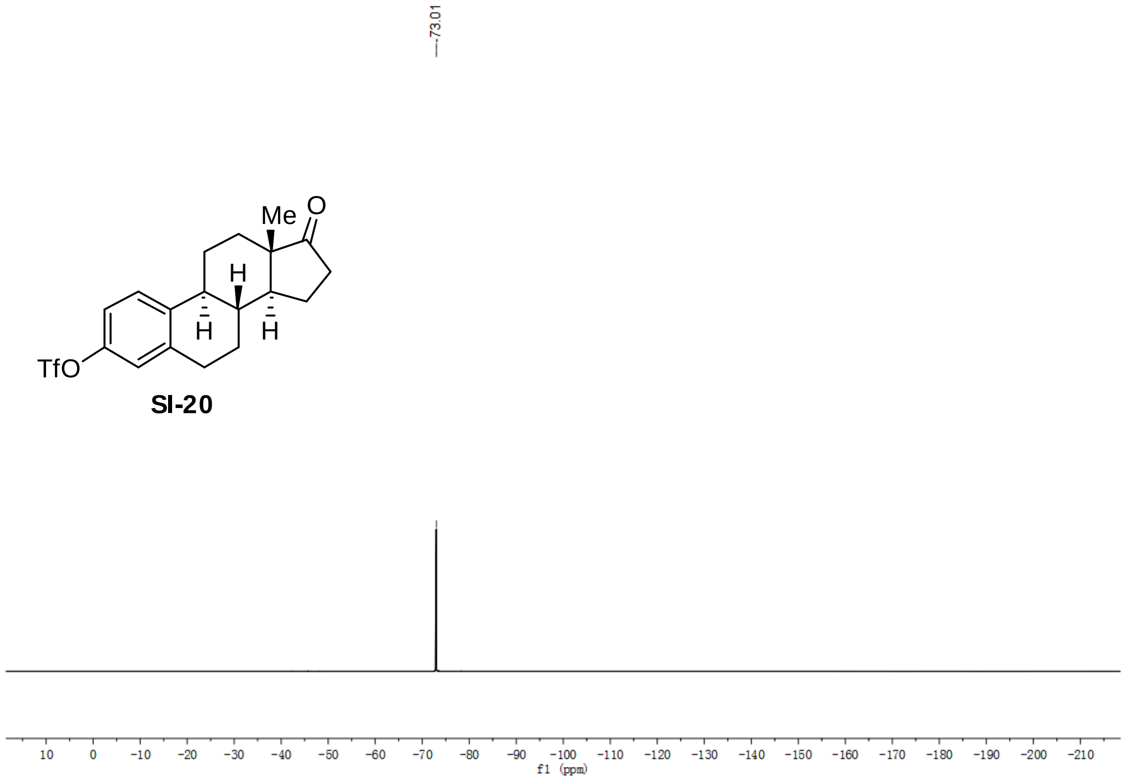
-73.01
TfO
Me
O
H
H
H
10 0 −10 −20 −30 −40 −50 −60 −70 −80 −90 −100 −110 −120 −130 −140 −150 −160 −170 −180 −190 −200 −210
f1 (ppm)

SI-20

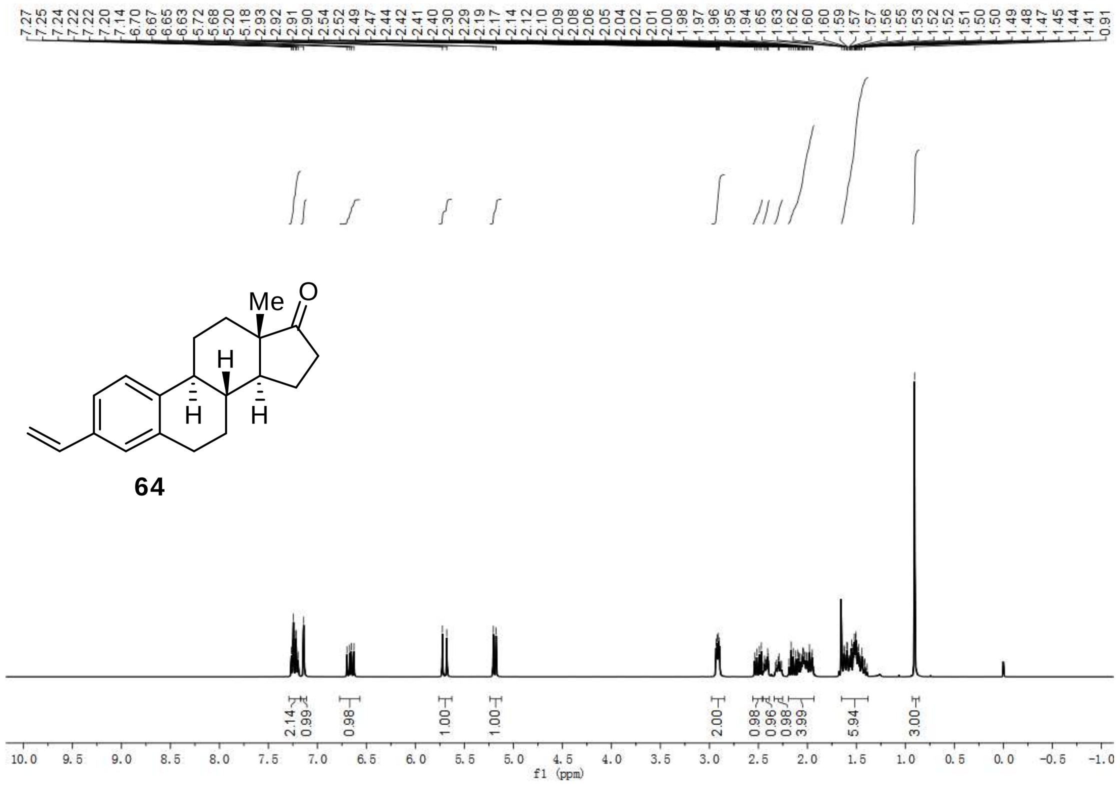
7.27 7.25 7.24 7.22 7.22 7.20 7.14 6.70 6.67 6.65 6.63 5.72 5.68 5.20 5.18 2.93 2.92 2.91 2.90 2.54 2.52 2.49 2.47 2.44 2.42 2.41 2.40 2.30 2.29 2.19 2.17 2.14 2.12 2.10 2.09 2.08 2.06 2.05 2.04 2.02 2.01 2.00 1.98 1.97 1.96 1.95 1.94 1.65 1.63 1.62 1.60 1.60 1.59 1.57 1.57 1.56 1.55 1.53 1.52 1.52 1.51 1.50 1.49 1.48 1.47 1.45 1.44 1.41 0.91
Me
O
H
H
H
64
2.14 0.99 0.98 1.00 1.00 2.00 0.98 0.96 0.98 3.99 5.94 3.00
10.0 9.5 9.0 8.5 8.0 7.5 7.0 6.5 6.0 5.5 5.0 4.5 4.0 3.5 3.0 2.5 2.0 1.5 1.0 0.5 0.0 -0.5 -1.0
f1 (ppm)

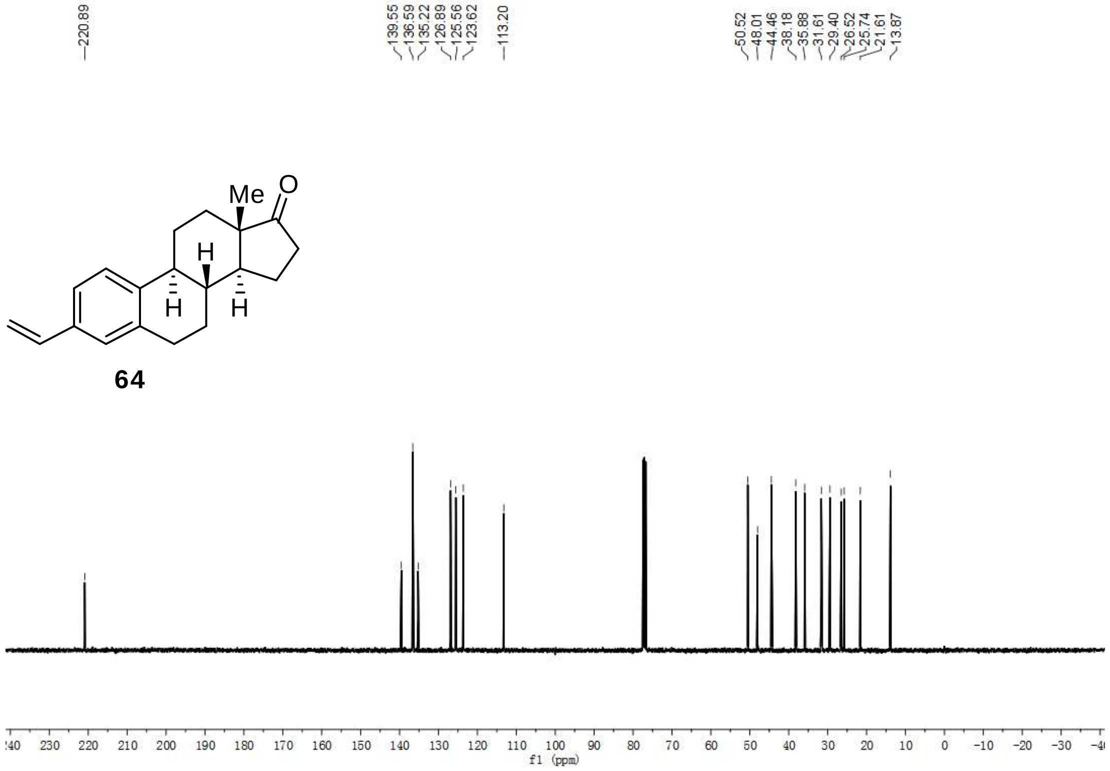
220.89
139.55 136.59 135.22 126.89 125.56 123.62 113.20
50.52 48.01 44.46 38.18 35.88 31.61 29.40 26.52 25.74 21.61 13.87
Me
O
H
H
H
64
240 230 220 210 200 190 180 170 160 150 140 130 120 110 100 90 80 70 60 50 40 30 20 10 0 -10 -20 -30 -40
f1 (ppm)

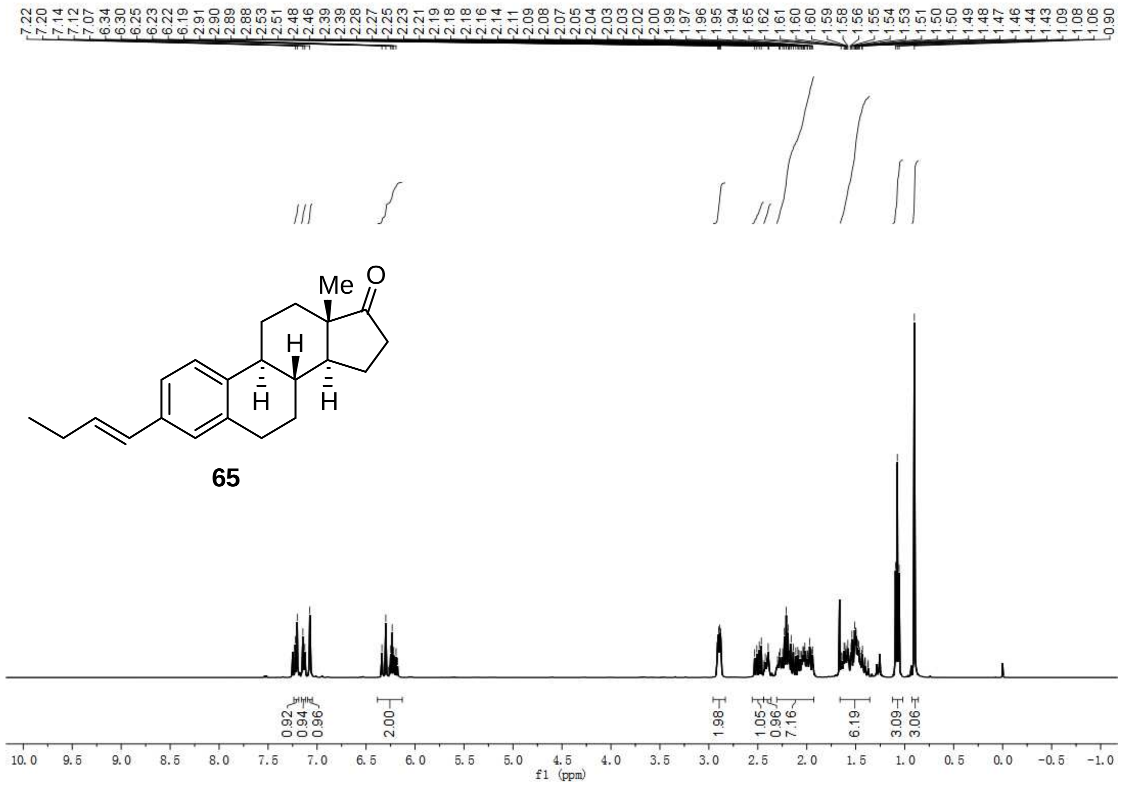
7.22 7.20 7.14 7.12 7.07 6.34 6.30 6.25 6.23 6.22 6.19 2.91 2.90 2.89 2.88 2.53 2.51 2.48 2.46 2.39 2.39 2.28 2.27 2.25 2.23 2.21 2.19 2.18 2.16 2.14 2.11 2.09 2.08 2.07 2.05 2.04 2.03 2.03 2.02 2.00 1.99 1.97 1.96 1.95 1.94 1.65 1.62 1.61 1.60 1.60 1.59 1.58 1.56 1.55 1.54 1.53 1.51 1.50 1.50 1.49 1.48 1.47 1.46 1.44 1.43 1.09 1.08 1.06 0.90
Me
O
H
H
H
65
0.92
0.94
0.96
2.00
1.98
1.05
0.96
7.16
6.19
3.09
3.06
f1 (ppm)

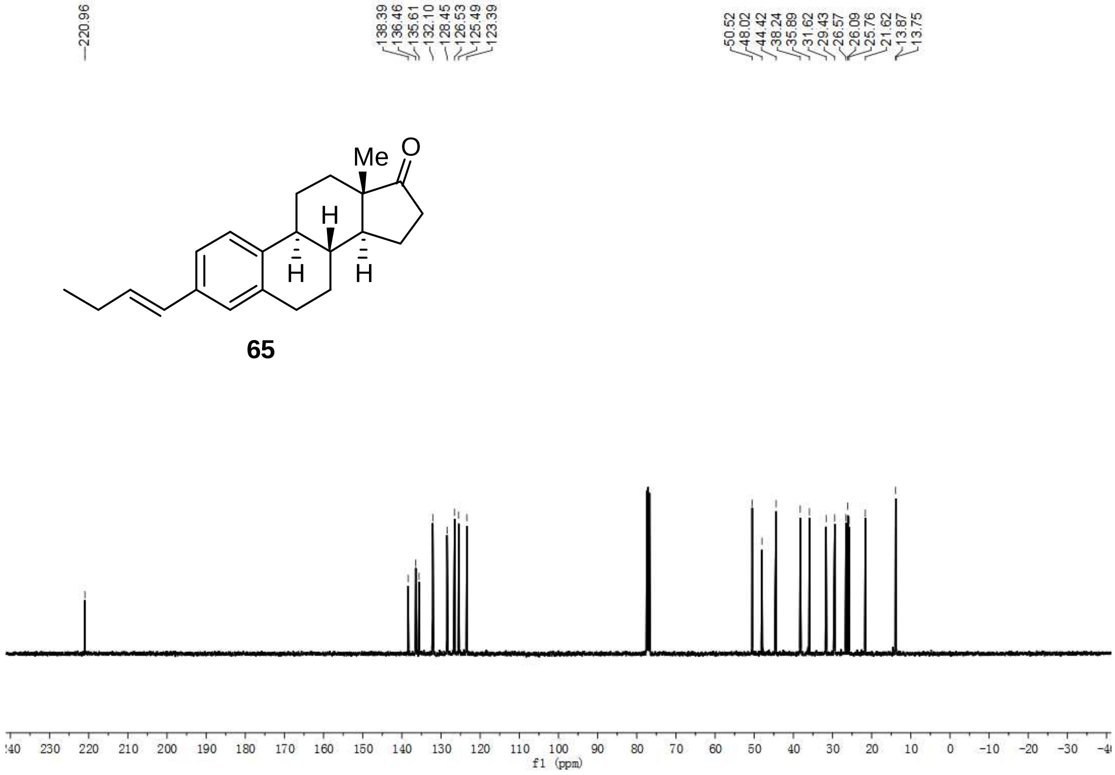
220.96
138.39 136.46 135.61 132.10 128.45 126.53 125.49 123.39
50.52 48.02 44.42 38.24 35.89 31.62 29.43 26.57 26.09 25.76 21.62 13.87 13.75
Me
O
H
H
H
65
f1 (ppm)

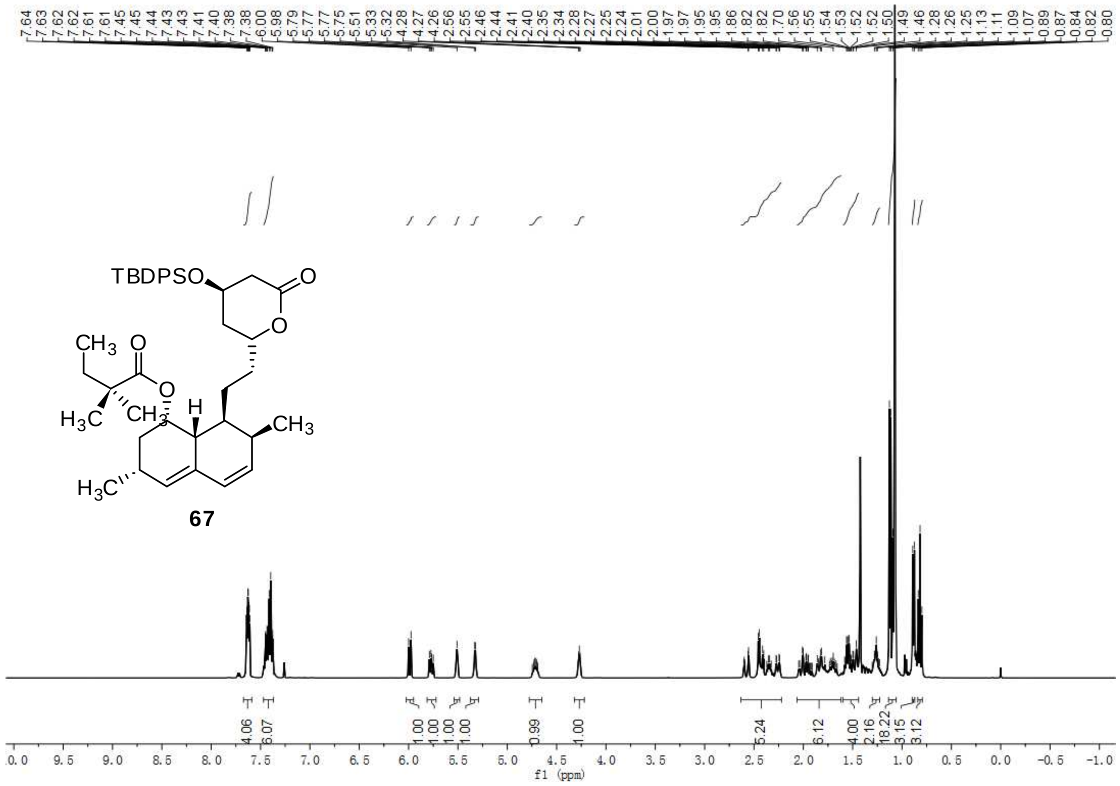
7.64 7.63 7.62 7.61 7.45 7.45 7.44 7.43 7.43 7.41 7.40 7.38 7.38 6.00 5.98 5.79 5.77 5.75 5.51 5.33 5.32 4.28 4.27 4.26 2.56 2.55 2.46 2.44 2.41 2.40 2.35 2.34 2.28 2.27 2.25 2.24 2.21 2.01 2.00 1.97 1.95 1.95 1.86 1.82 1.82 1.70 1.56 1.55 1.54 1.53 1.52 1.52 1.50 1.49 1.46 1.28 1.26 1.25 1.13 1.11 1.09 1.07 0.89 0.87 0.84 0.82 0.80
TBDPSO
O
O
CH3
O
O
H
H3C
CH3
CH3
H3C
67
4.06 6.07 1.00 1.00 1.00 1.00 0.99 1.00 5.24 6.12 4.00 2.16 18.22 3.15 3.12
f1 (ppm)

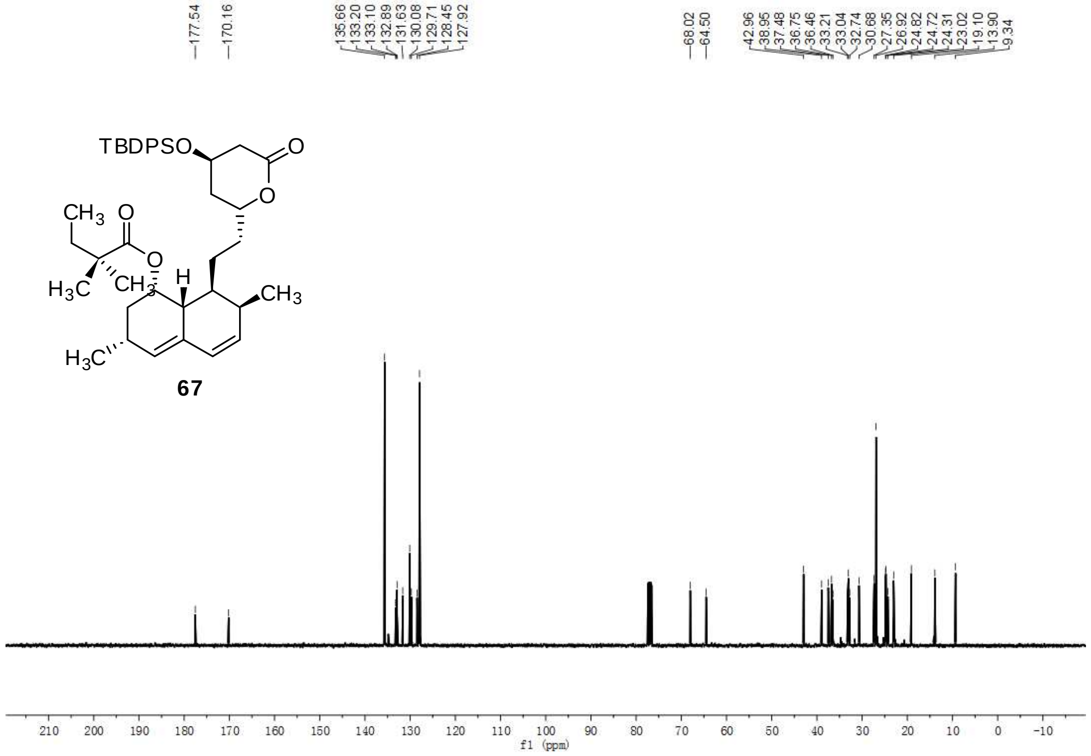
177.54 170.16
135.66 133.20 132.89 131.63 130.08 129.71 128.45 127.92
68.02 64.50
42.96 38.95 37.48 36.75 36.46 33.21 33.04 32.74 30.68 27.35 26.92 24.82 24.72 24.31 23.02 19.10 13.90 9.34
TBDPSO
O
O
CH3
O
O
H
H3C
CH3
CH3
H3C
67
f1 (ppm)

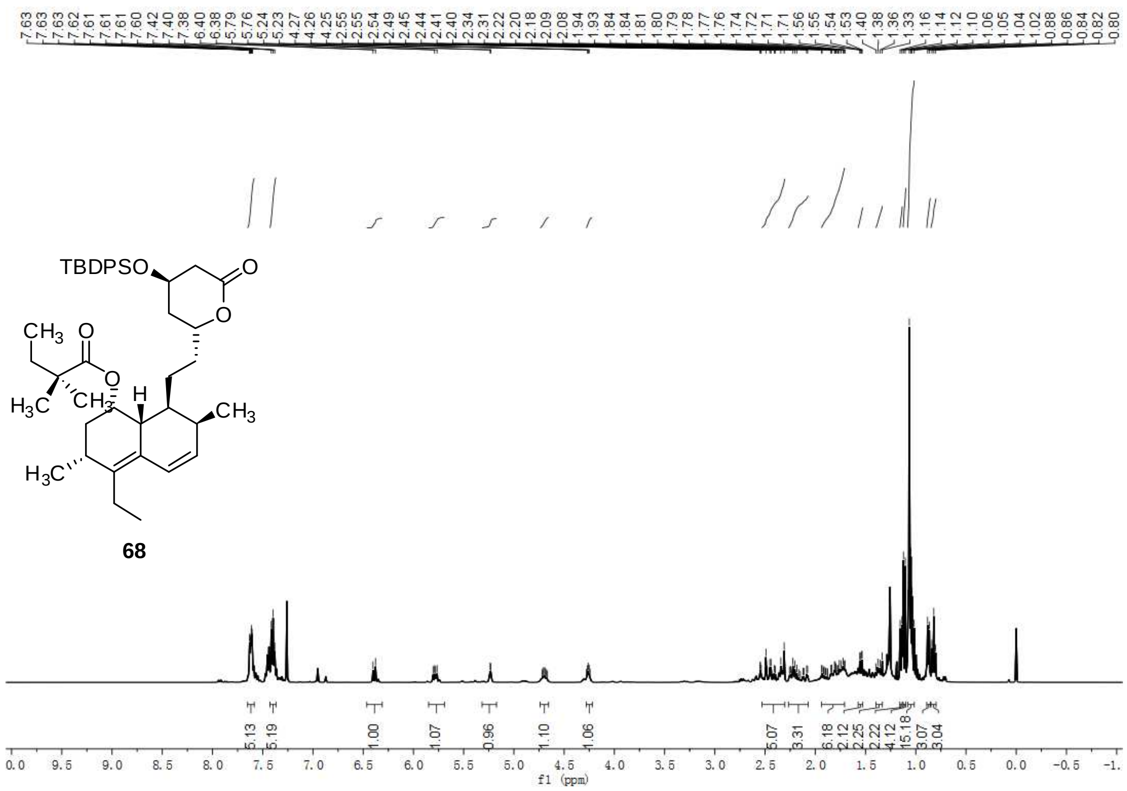
7.63 7.63 7.62 7.61 7.61 7.60 7.42 7.40 7.38 6.40 6.38 5.79 5.76 5.24 5.23 4.27 4.26 4.25 2.55 2.54 2.49 2.45 2.44 2.41 2.40 2.34 2.31 2.22 2.20 2.18 2.09 2.08 1.94 1.93 1.84 1.84 1.81 1.80 1.79 1.78 1.77 1.76 1.74 1.72 1.71 1.71 1.56 1.55 1.54 1.53 1.40 1.38 1.36 1.33 1.16 1.14 1.12 1.10 1.06 1.05 1.04 1.02 0.88 0.86 0.84 0.82 0.80
TBDPSO
O
O
CH3
O
O
H
CH3
H3C
CH3
H3C
5.13 5.19 1.00 1.07 0.96 1.10 1.06 5.07 3.31 6.18 2.12 2.25 2.22 4.12 15.18 3.07 3.04
f1 (ppm)

68

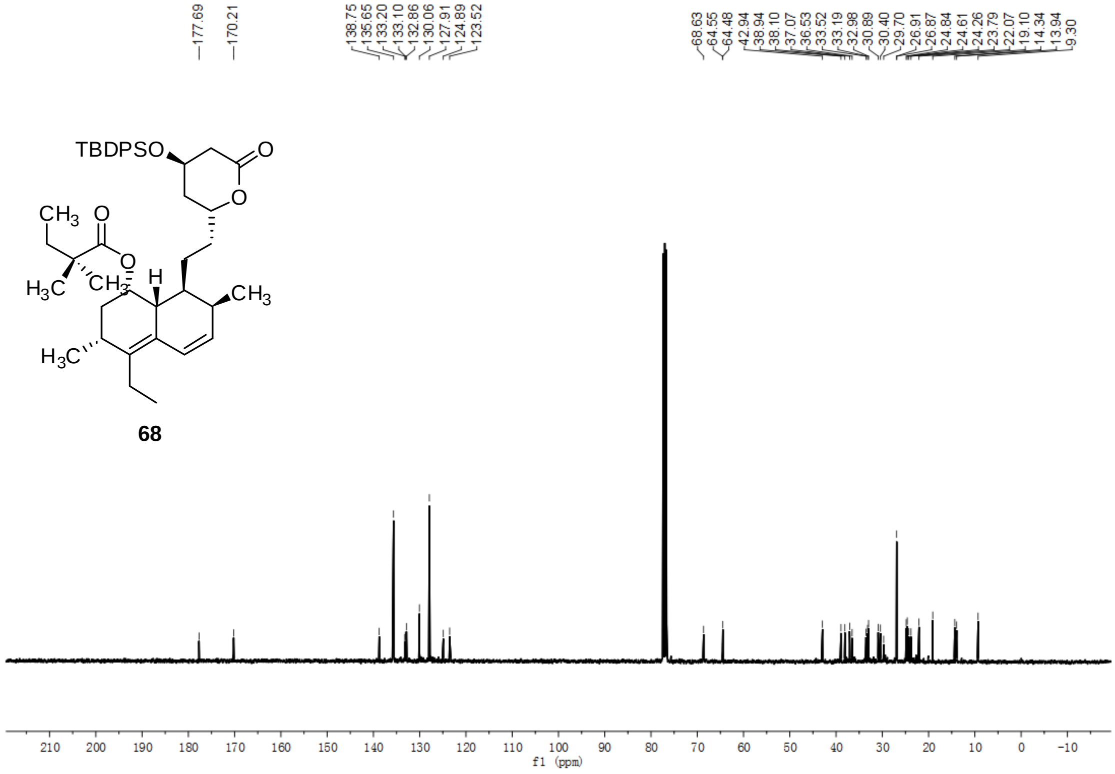
177.69 170.21
138.75 135.65 133.20 133.10 132.86 130.06 127.91 124.89 123.52
68.63 64.55 64.48 42.94 38.94 38.10 37.07 36.53 33.52 33.19 32.98 30.89 30.40 29.70 26.91 26.87 24.84 24.61 24.26 23.79 22.07 19.10 14.34 13.94 9.30
TBDPSO
O
O
CH3
O
O
H
CH3
H3C
CH3
H3C
f1 (ppm)

68

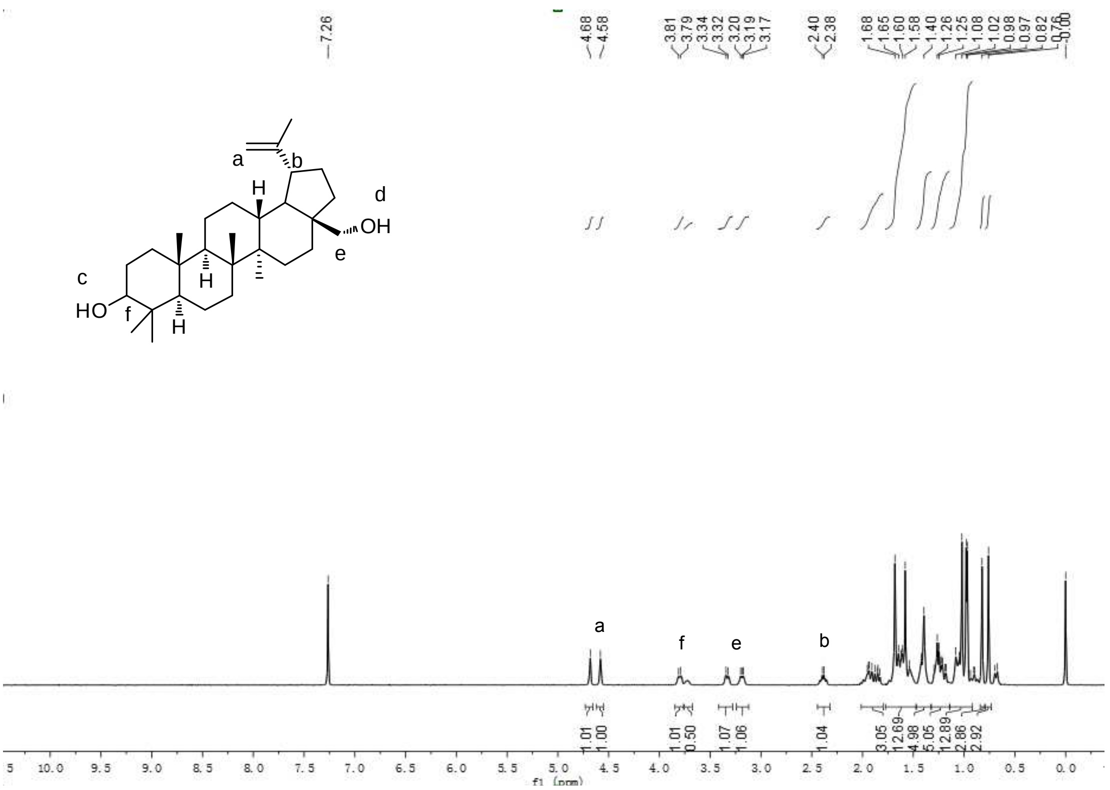

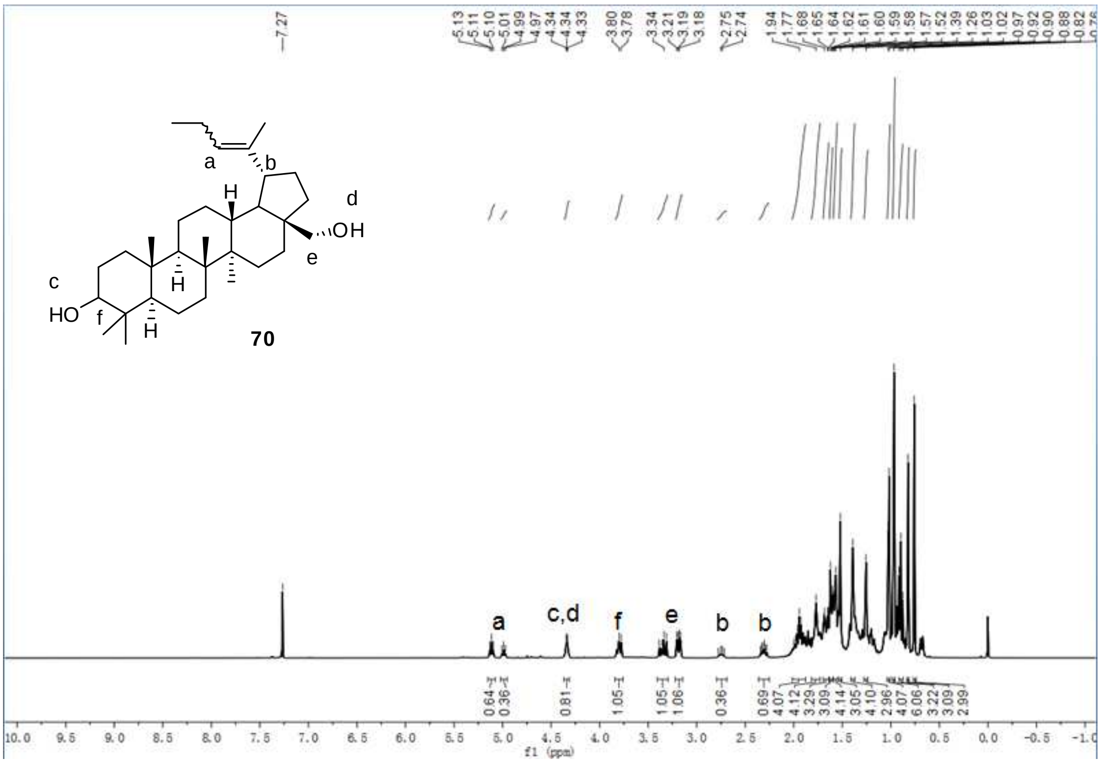

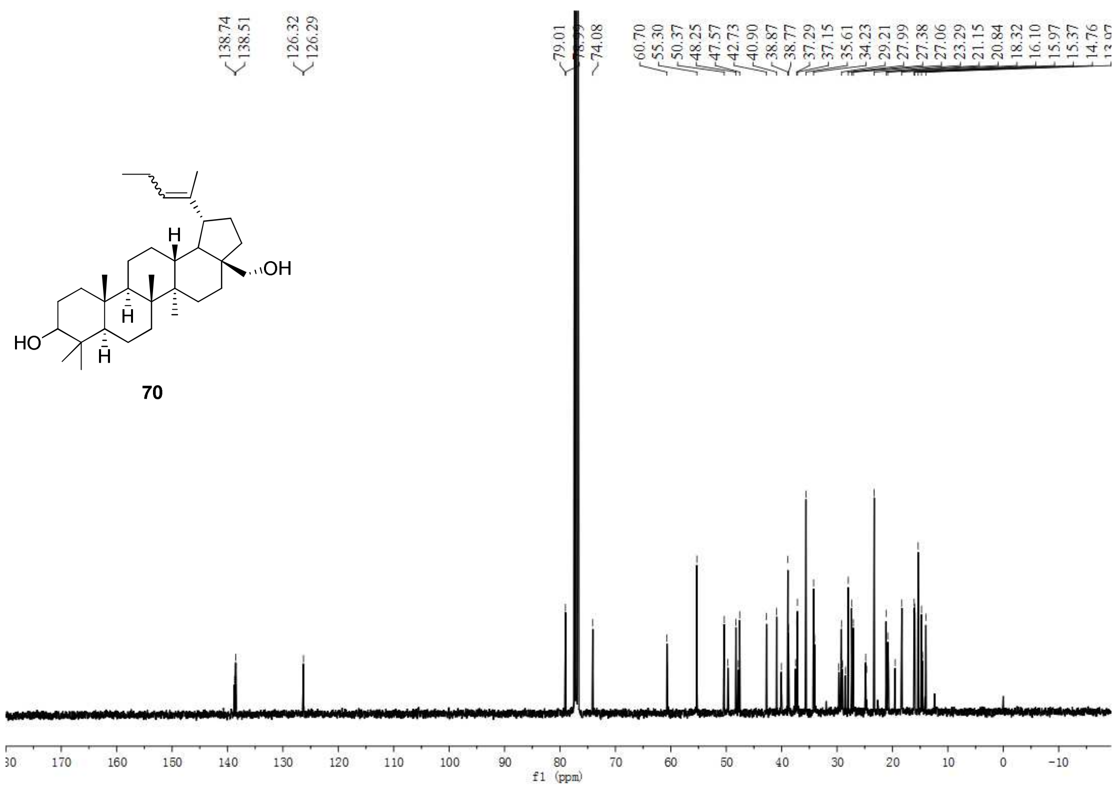
138.74
138.51
126.32
126.29
79.01
74.08
60.70
55.30
50.37
48.25
47.57
42.73
40.90
38.87
38.77
37.29
37.15
35.61
34.23
29.21
27.99
27.38
27.06
23.29
21.15
20.84
18.32
16.10
15.97
15.37
14.76
HO
H
H
H
OH
70
f1 (ppm)

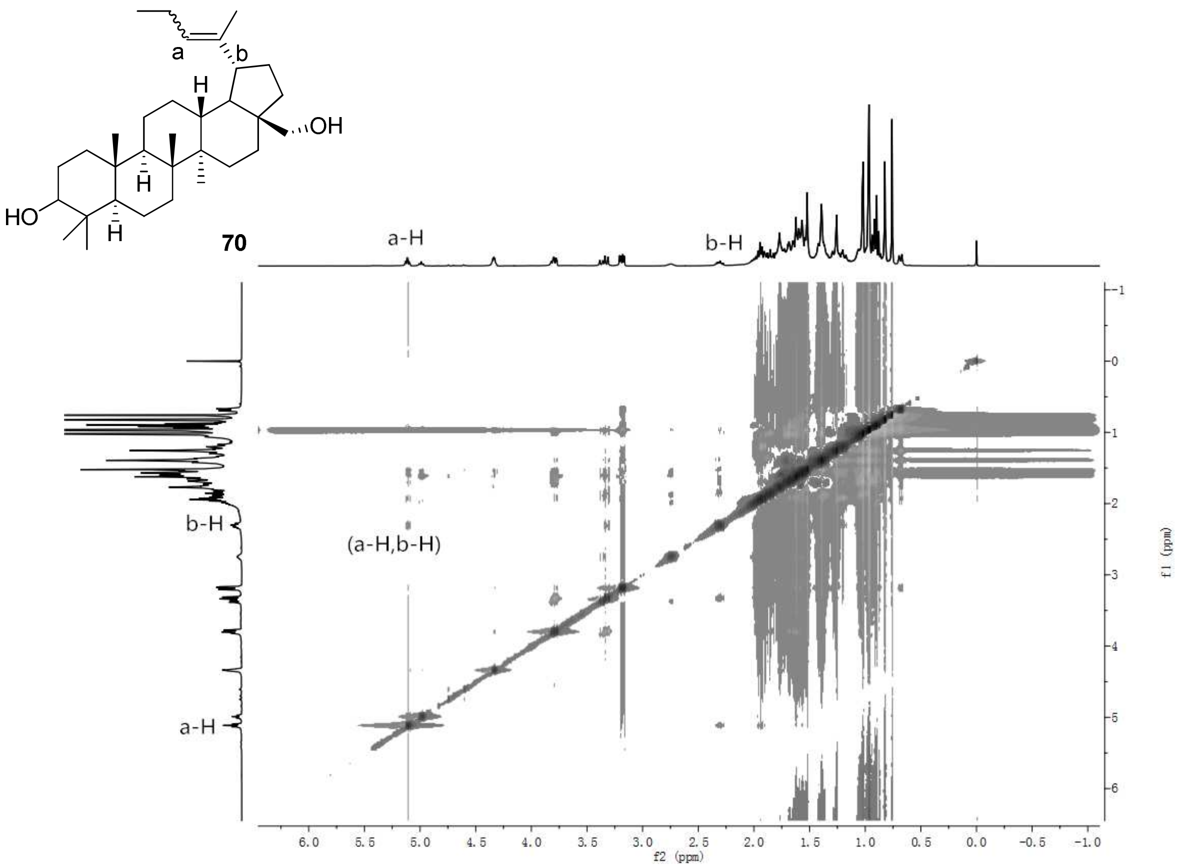
a
b
H
OH
H
HO
H
70
a-H
b-H
(a-H,b-H)
f2 (ppm)
f1 (ppm)

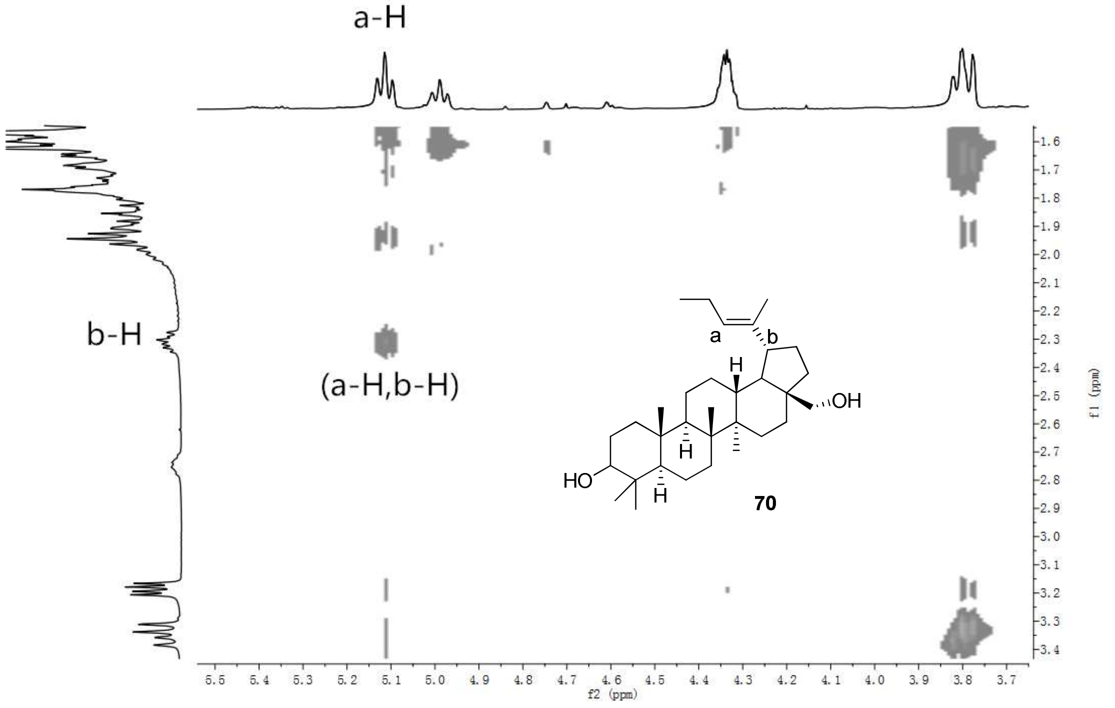
a-H
b-H
(a-H,b-H)
a
b
HO
OH
70
f2 (ppm)
f1 (ppm)

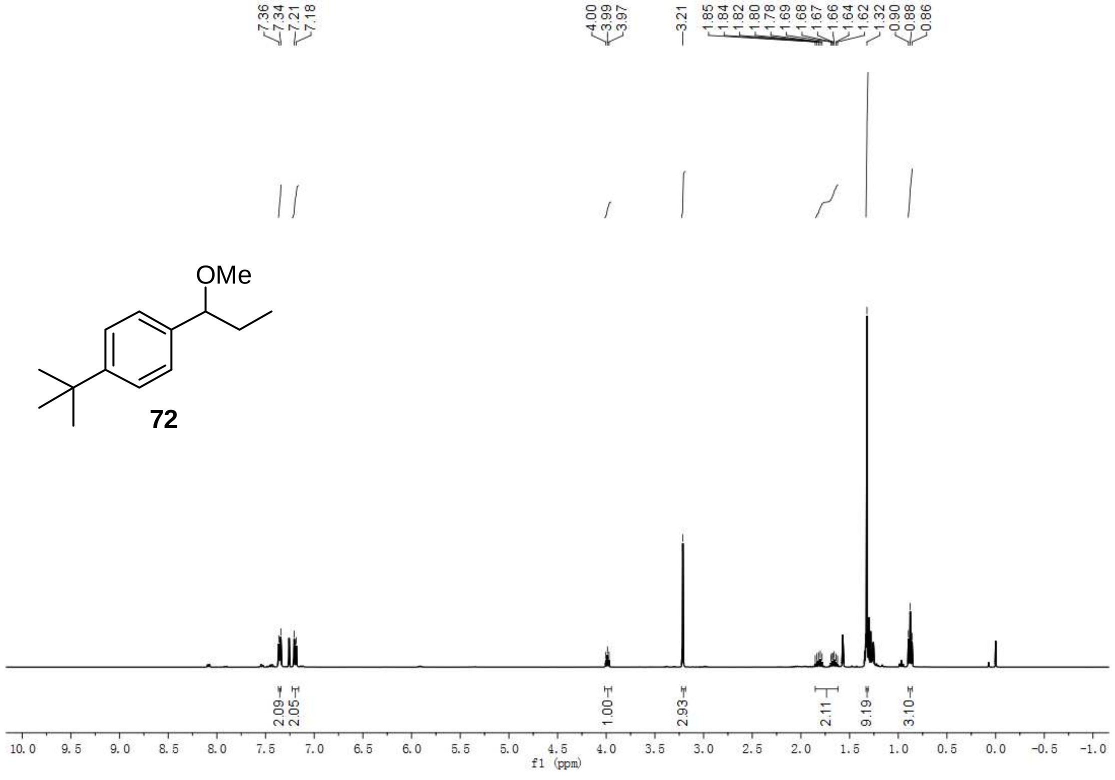
OMe
72
f1 (ppm)

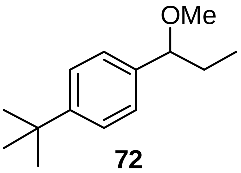

72

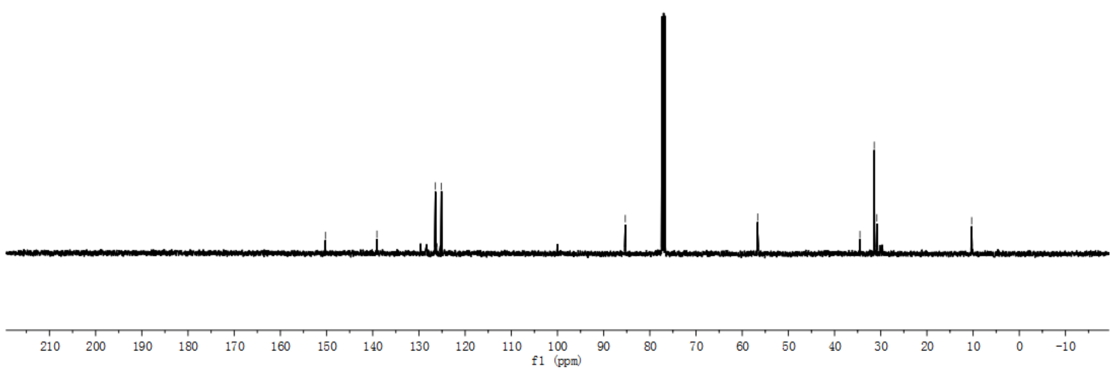

Supplement: Supplementary file 1 [file SC-008-C6SC04274K-s001.pdf]
